# Tunable TriPcides suppress virulence factor secretion during *Staphylococcus aureus* infection and kill dormant cells

**DOI:** 10.1126/sciadv.aec9100

**Published:** 2026-05-06

**Authors:** Hasan Tükenmez, Taylor M. Nye, Pardeep Singh, Aaron Mychack, Mari Bonde, Suzanne Hickerson, Chloe L. P. Obernuefemann, Jerome S. Pinkner, Shaochun Zhu, Souvik Sarkar, Jaideep B. Bharate, Ingeborg van der Lingen, Anh Quoc Ntuyenm, V. U. Bhaskara Rao, Anders E. G. Lindgren, Hanna Klein, Zongsen Zou, Karen W. Dodson, Suzanne Walker, Andre Mateus, Jörgen Johansson, Michael G. Caparon, Fredrik Almqvist, Scott J. Hultgren

**Affiliations:** ^1^ Nordic BioConsult AB, SE-91332, Holmsund, Sweden.; ^2^ QureTech Bio AB, SE-90736, Umeå, Sweden.; ^3^ Department of Molecular Microbiology and Center for Women’s Infectious Disease Research, Washington University School of Medicine, St. Louis, MO 63110, USA.; ^4^ Department of Chemistry, Umeå University, SE-90187 Umeå, Sweden.; ^5^ Department of Microbiology, Harvard Medical School, Boston, MA 02115, USA.; ^6^ Department of Molecular Biology, Umeå University, SE-90187 Umeå, Sweden.; ^7^ The Laboratory for Molecular Infection Medicine Sweden (MIMS), SE-90187, Umeå, Sweden.; ^8^ Umeå Centre for Microbial Research, UCMR, Umeå University, SE-90187 Umeå, Sweden.

## Abstract

Antimicrobial resistance (AMR) in common bacterial pathogens, including methicillin-resistant *Staphylococcus aureus* (MRSA), is an increasingly dire public health threat, with MRSA accounting for up to 90% of *S. aureus* infections. To expand the treatment arsenal against MRSA infections, we developed a class of tunable three-dimensional tricyclic 2-pyridones, termed TriPcides, that can kill MRSA resistant to last-resort antibiotics and eliminate MRSA persister cells. No preexisting resistance was detected across hundreds of clinical isolates, and continuous exposure of MRSA to TriPcides did not elicit detectable resistance. Treatment with TriPcides causes a rapid decrease in membrane integrity and increased levels of reactive oxygen species. Last, TriPcides effectively reduce secretion of important virulence factors and result in reduced ulcer size and healing time in *S. aureus* murine skin and soft tissue infections but do not reduce bacterial burden.

## Teaser

TriPcides offer a promising strategy against MRSA infections by targeting both dormant bacteria and their virulence machinery.

## INTRODUCTION

Antimicrobial-resistant (AMR) bacterial infections represent a growing public health crisis, with 1.27 million deaths caused by AMR infections and 4.95 million AMR-associated deaths in 2019, surpassing the number of deaths from HIV/AIDS and malaria combined. Without substantial public health interventions and the development of new classes of antibiotics, the number of deaths due to antibiotic resistance could grow to 10 million deaths per year by 2050, with a projected cost of $100 trillion to the global economy (*1*). This is of particular importance to those who, due to immune deficiencies, chronic disease, cancer, etc., are prone to frequent infections. From an antibiotic development perspective, few new classes of clinically approved antibiotics have been developed since the 1980s, and resistance has already emerged to many of these new classes (*2*–*5*). Approximately 80% of antibiotics in use today are derived from natural sources. Because bacteria have been exposed to these compounds over long evolutionary periods, they are more likely to develop resistance mechanisms against them (*6*). Thus, there is an urgent need to develop new classes of synthetic antibiotics without preexisting resistance to combat the rise of AMR infections.

One of the leading types of AMR bacterial infections are skin and soft tissue infections (SSTIs), which account for 14 million out-patient visits and 900,000 inpatient hospital visits per year in the United States alone (*7*, *8*). Complicated SSTIs, which include more severe SSTIs in both healthy patients and those with comorbidities, can be particularly difficult to treat and may require surgical debridement or amputation. The leading cause of SSTIs in North America is the Gram-positive pathogen *Staphylococcus aureus* (*9*). Infections caused by methicillin-sensitive *S. aureus* (MSSA) are typically treated with first-generation cephalosporins or penicillinase-resistant penicillins (oxacillin and nafcillin) (*10*), whereas SSTIs caused by methicillin-resistant *S. aureus* (MRSA) are commonly treated with vancomycin. Vancomycin-intermediate *S. aureus* infections or issues arising from patient tolerance/safety of vancomycin necessitate the use of other last resort antibiotics, such as daptomycin and linezolid. Use of these antibiotics is also limited due to costs and adverse reactions, as well as antibiotic stewardship considerations, necessitating the development of new classes of cost-effective antibiotics with less severe side effects that can be used to treat SSTIs caused by MRSA and other highly resistant Gram-positive bacteria.

We previously developed bicyclic GmPcides, a class of synthetic antibiotics that share a thiazolo ring-fused 2-pyridone scaffold with fine-tunable properties that are broadly efficacious at killing Gram-positive pathogens, including MRSA, and erythromycin-resistant *Streptococcus pyogenes*, as well as vancomycin-resistant enterococcus (VRE), multidrug-resistant *Streptococcus pneumoniae*, and clindamycin-resistant *Streptococcus agalactiae* (*11*, *12*). Despite the demonstrated efficacy of lead bicyclic compounds, resistances quickly emerged (within a few bacterial passages) in several Gram-positive bacteria, including VRE and *Listeria monocytogenes* (*12*, *13*). Repeated exposure over these passages likely facilitated the selection of preexisting resistant variants. Isolation of genomic DNA and subsequent whole genome sequencing revealed that most PS757-resistant isolates (a lead bicyclic compound) in VRE had acquired mutations in the regulatory region of the *lmrB* efflux pump, presumably increasing tolerance through efflux of the compound outside of the cell (*13*).

Here, we describe the design of novel tricyclic GmPcides (TriPcides) based on a sp^3^-rich thiazolo-2-pyridone scaffold. Like GmPcides, TriPcides effectively kill a wide range of Gram-positive pathogens of concern, but they are not sensitive to the efflux-based mechanisms that confer resistance to GmPcides. Further, we could not select mutants resistant to TriPcides by continually increasing exposure to the compounds, as has been done previously for GmPcides. Moreover, no preexisting resistance to TriPcides was detected across 121 clinical MRSA and MSSA isolates as well as 109 enterococcal isolates tested. We also found that TriPcides kill exponential and nondividing stationary phase MRSA as well as persister cells, resulting in rapid membrane disruption across all these cell types. Transposon sequencing (Tn-seq) experiments revealed that treatment with sublethal concentrations of TriPcides results in a fitness disadvantage for insertions in cellular processes involved in adenosine triphosphate (ATP) generation and reactive oxygen species (ROS) stress response, and we confirmed that ATP and ROS levels were increased in TriPcide-treated cells, providing insight into the effects observed in otherwise dormant cell states. Last, we show that TriPcides markedly reduce ulcer size and healing time in murine SSTI in the absence of a reduction of viable staphylococcal cells, likely through a TriPcide-dependent decrease in secreted staphylococcal virulence factors. Thus, TriPcides represent a novel tunable antibiotic capable of attenuating MRSA infection and targeting clinically relevant and difficult-to-kill dormant cell states.

## RESULTS

### Design and development of TriPcide antibiotics without detectable resistance

**Fig. 1.**
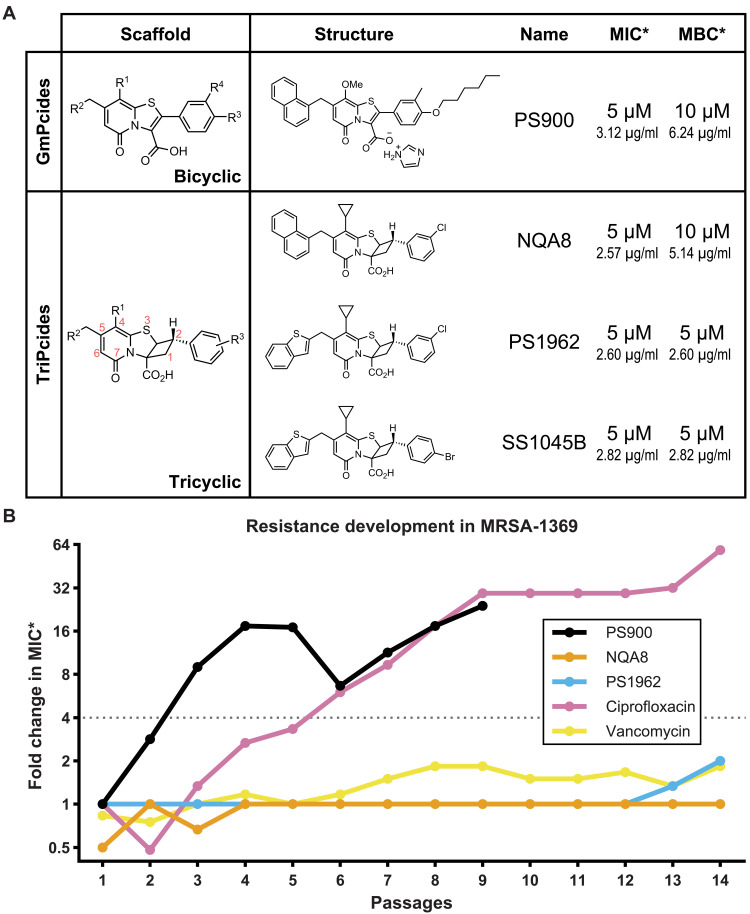
MRSA-1369 did not develop resistance toward the new generation TriPcides. (**A**) Structures of the GmPcide and TriPcide scaffolds, PS900, NQA8, PS1962, and SS1045B with their corresponding MICs and MBCs for MRSA-1369. (**B**) Resistance development in MRSA-1369 by continued exposure to GmPcides, TriPcides, or antibiotics. A fourfold increase (dotted line) in MIC is indicative of bacterial resistance. The results are shown as means from biological triplicates. *See Materials and Methods.

PS757 is one of the most potent bicyclic GmPcides developed to date. It has a high degree of sp^2^ bonds that results in a flat scaffold and is substituted with a hydrocarbon tail that increases killing activity (Fig. 1A) (*13*). We hypothesized that both these structural features (flat scaffold and a hydrocarbon tail) render them susceptible to efflux-mediated resistance mechanisms (*13*). Taking advantage of evidence that the likelihood of delivering a compound as a drug correlates with increased complexity (*14*), we designed a three-dimensional scaffold to decrease their susceptibility to efflux. We envisaged that annulation of the bicyclic thiazolo-2-pyridone scaffold with a substituted cyclobutyl ring (Fig. 1A; see Materials and Methods) would keep the essential structural elements like a substituted phenyl group at C2 (numbering is highlighted in Fig. 1A) in their positions while simultaneously increasing the complexity and number of sp^3^ carbons. The [2 + 2] photocycloaddition reaction is a commonly used approach to preparing cyclobutane ring–containing molecules. Such reactions can be performed under either ultraviolet light or visible light in the presence of a photocatalyst. However, there are not many examples of [2 + 2] photocycloaddition reactions that can be done under visible light without a photocatalyst. To the best of our knowledge, [2 + 2] photocycloaddition reactions of thiazolo-2-pyridones have not been explored previously. To this end, we developed a photocatalyst-free visible light–mediated, regio- and diastereoselective [2 + 2] cycloaddition reaction of thiazolo-2-pyridones with styrenes (see Materials and Methods). The developed methodology is very robust and has a wide substrate scope, and this transformation can be performed at a late stage in the synthetic sequence.

An initial set of three sp^3^-rich tricyclic GmPcides (IL262, IL305, and NQA8; table S1) was synthesized by keeping 1-naphthyl at position C5, cyclopropyl or methoxy at C4, and a substituted phenyl at C2. These compounds were screened for antibacterial activity against MRSA using minimal inhibitory concentration (MIC) and minimal bactericidal concentration (MBC), as well as for selected properties including in vitro hemolysis and HeLa cell cytotoxicity. Compound NQA8 (5 μM MIC) bearing a cyclopropyl ring at C4 and 3-chlorophenyl at C2 showed potent anti-MRSA activity compared to IL305 (>25 μM MIC) and IL262 (10 μM MIC), indicating that antibacterial activity is influenced by substituents at positions C2 and C4. The corresponding endo diastereomers IL263 (>25 μM MIC) and NQA9 (25 μM MIC) were found to be less potent compared to IL262 (10 μM MIC) and NQA8 (5 μM MIC), further confirming our hypothesis that the phenyl group at position C2 needs to occupy a spatial arrangement similar to that in bicyclic GmPcides. To further improve the potency and aforementioned selected properties, we next focused on structure-activity relationships (SAR) with respect to substituents at position C5 by introducing various aryls/heteroaryls (PS1961, PS1962, PS1963, PS1965, and PS1970; table S1). Of these, compound PS1962, which contains a 2-benzothiophene group at C5, exhibited similar MIC (5 μM) but better MBC (from 10 μM to 5 μM) values compared to NQA8 (table S1), and both exhibited MIC and MBC values below or similar to those of the lead bicyclic GmPcide PS757 (table S1). Moreover, both tricyclic compounds demonstrated low hemolytic activity (<1% at 100 μM) and no significant cytotoxicity at concentrations up to 4 to 8× MIC (table S1).

In a continuous exposure assay (CEA; see Materials and Methods), several mutants resistant to bicyclic GmPcide PS900 (PS757 with an imidazole salt to improve solubility) in a MRSA-1369 strain were isolated, which included mutations that mapped to the FarR fatty acid efflux pump system, much like the efflux mutants that were observed for PS900-resistant *Enterococcus faecalis* and *L. monocytogenes* mutants (Fig. 1B and table S2). However, all the PS900-resistant strains were sensitive to treatment with TriPcide PS1962, with MICs increasing no more than twofold (5 to 10 μM) relative to wild type (table S2). Further, we were unable to isolate resistant mutants to TriPcide PS1962 or NQA8 using the CEA assay up to 14 passages (Fig. 1B). Despite these advances, PS1962 was found to have low kinetic solubility (2.32 μM) which limited its further use for in vitro and in vivo assays. To improve kinetic solubility, we focused on modifying the substituents at position C2. Thus, a series of analogs was synthesized by introducing aryl and heteroaryl groups. While the incorporation of heteroaryls such as pyridazine, pyrazine, and pyridine at C2 abolished antibacterial activity, substituted phenyl groups such as 4-bromo and 4-trifluoromethyl phenyl retained activity (table S3). Compound SS1045B having a 4-bromo phenyl group at position C2 was found to exhibit superior kinetic solubility (64.64 μM) while maintaining low hemolytic activity and low cytotoxicity in both HeLa and HepG2 cells and retaining MIC and MBC values similar to PS1962. Thus, SS1045B was selected to test activity against a broad panel of Gram-positive clinical isolates. All clinical isolates tested, including 121 *S. aureus* (53% MRSA and 47% MSSA) and 109 enterococcal species (65% VRE and 35% vancomycin-sensitive enterococcus), were sensitive to SS1045B (fig. S1), with MIC90 values ranging between 2 and 4 μg/ml (3.5 to 7μM). An exception was a single VRE isolate with an MIC of 8 μg/ml (fig. S1). Furthermore, we could not observe any resistance mutants emerging upon multiple screenings on agar plates. TriPcides were also effective against all other Gram-positive bacteria tested, including *Bacillus subtilis*, *S. pyogenes*, and *S. pneumoniae* (fig. S2). These results highlight that TriPcides are highly effective against clinical MDR isolates.

### TriPcides cause membrane disruption and kill MRSA exponential, stationary, and persister cells

**Fig. 2.**
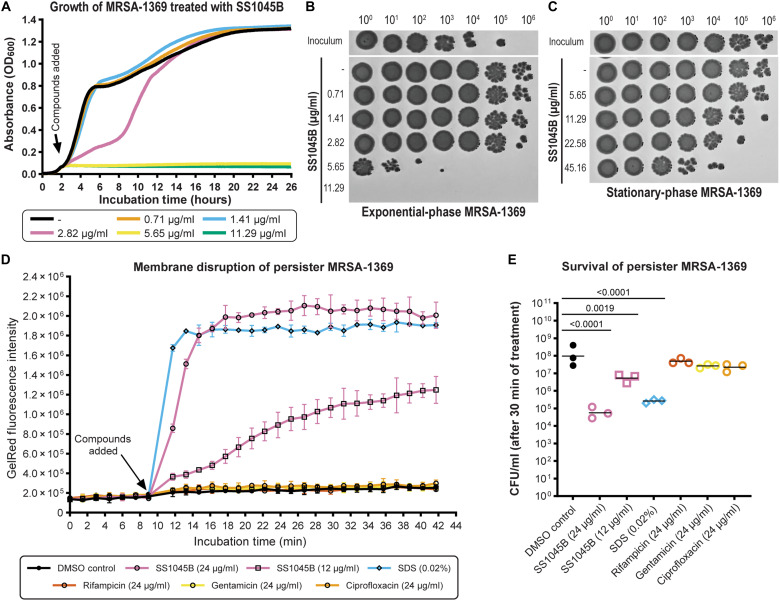
SS1045B causes membrane disruption and kills exponential, stationary, and persister MRSA-1369 cells. (**A**) Growth of exponential-phase MRSA-1369 cultures in BHI medium in the presence of SS1045B (0.71 to 11.29 μg/ml; 1.25 to 20 μM). MRSA-1369 cultures were started in the absence of SS1045B at 37°C. At the 2-hour time point, indicated concentrations of SS1045B were added to each culture, and the growth (OD_600_) was monitored for 24 hours at 37°C. (**B**) Spot titer assay of exponential-phase MRSA-1369 cultures at t2 (inoculum; before treatment) and t26 hours (posttreatment). (**C**) Spot titer assay of stationary phase MRSA-1369 cultures at t0 (inoculum; before treatment) and t24 hours (posttreatment). [(A) to (C)] Represented by one of the biological replicates, see fig. S3 for other biological replicates. (**D**) Monitoring membrane integrity of ciprofloxacin-persister MRSA-1369 cells. Membrane disruption was determined by monitoring the uptake of the membrane-impermeable fluorescent dye GelRed in the presence of DMSO (vehicle control), 0.02% SDS, SS1045B (12 to 24 μg/ml; 21.3 to 42.5 μM; 4 to 8× MIC), rifampicin (24 μg/ml; MIC, <0.006 μg/ml), gentamicin (24 μg/ml; MIC, <1.5 μg/ml), or ciprofloxacin (24 μg/ml; MIC, <0.38 μg/ml) for 30 min at 37°C in 1× PBS. The results are shown as means from biological triplicates, and error bars indicate the SD. (**E**) Determination of CFUs at the end of 30-min membrane integrity monitoring of ciprofloxacin-persister MRSA-1369 cells from (D). CFUs were determined by spot titer assay. Statistical analysis by ordinary one-way analysis of variance (ANOVA) test with Tukey’s comparison.

To better understand how TriPcides kill MRSA, growth curves and colony-forming unit (CFU) measurements were performed on exponential-phase cells treated with increasing concentrations of SS1045B. At concentrations more than 2.82 μg/ml (5 μM) SS1045B, no increase in optical density (OD) was observed over the 24-hour treatment period (Fig. 2A and Fig. S3, A and B), which was associated with up to a 3 log_10_ reduction in recovered CFUs relative to the initial inoculum (1.2 × 10^7^ cells/ml) (Fig. 2B and fig. S3, C and D). A GelRed fluorescence membrane integrity assay revealed that treatment with SS1045B resulted in a rapid compromise of the bacterial membrane. In this assay (see Materials and Methods), GelRed fluoresces only when bound to DNA, which can only happen in the presence of membrane perturbation to allow the membrane-impermeable GelRed to gain access to the cell cytoplasm. Membrane disruption assays were performed in phosphate-buffered saline (PBS) to minimize interference from growth media and to de-energize cells, which enhances detection of membrane permeability as described in previous studies (*15*–*17*). Comparison of untreated to treated exponential-phase cells demonstrated that significant GelRed fluorescence was observed within 5-min exposure to SS1045B (fig. S4A). While most existing antibiotics are primarily active against actively dividing cells, we previously showed that bicyclic GmPcides were able to kill nondividing stationary-phase enterococcal cells (*12*). When stationary-phase MRSA cells were treated with the tricyclic SS1045B, the nondividing cells also rapidly lost membrane integrity (fig. S4B) with a concomitant 1 to 3 log_10_ decrease in recoverable CFUs following 24-hour treatment with SS1045B (11.29 to 45.16 μg/ml; 20 to 80 μM) relative to untreated cells (Fig. 2C and Fig. S3, E and F).

Given the effectiveness of SS1045B in killing nondividing stationary phase cells, we assayed its activity on *S. aureus* persister cells. Nondividing persister cells are a notable clinical problem because they can evade antibiotic treatment through reduced metabolic activity (*18*). MRSA persister cells were selected by an established method (*17*) via treatment with ciprofloxacin (24 μg/ml; MIC, <0.38 μg/ml). Subsequent challenge with SS1045B (12 to 24 μg/ml; 21.3 to 42.5 μM) resulted in near immediate membrane disruption, as determined using the GelRed fluorescence assay (Fig. 2D) at levels similar to the detergent SDS (0.02%). In contrast, membrane disruption was not observed following treatment of persister cells with ciprofloxacin (24 μg/ml), gentamicin (MIC, <1.5 μg/ml), or rifampicin (MIC, <0.006 μg/ml), far above the MICs of these compounds (Fig. 2D). Thirty-minute treatment with SS1045B resulted in >3 log_10_ reduction in the number of recoverable CFUs from persister cell cultures, whereas there was no significant reduction that was observed following treatment with rifampicin, gentamicin, or ciprofloxacin (Fig. 2E). Similar results were obtained using a gentamicin-based method to generate persister cells (*19*), with SS1045B treatment producing identical kinetics of membrane disruption, as well as a 3 log_10_ reduction in recoverable CFUs after 30-min treatment (fig. S5).

### TriPcides kill MRSA strains with varied daptomycin sensitivities

In addition to persister cells, the multidrug resistance profile of MRSA provides treatment challenges, often necessitating the use of “last resort” antibiotics such as daptomycin. Daptomycin is also known to cause rapid membrane disruption, but it requires Ca^2+^ ions (typically supplemented from CaCl_2_) to bind bacterial membranes and exert its bactericidal effect (*20*). We tested the effectiveness of TriPcides against five clinical MRSA isolates with a range of daptomycin sensitivities. Daptomycin MICs of these strains ranged from ~6.7 to 21.7 μg/ml when supplemented with CaCl_2_ (50 μg/ml) which were all above the clinical breakpoint (1 μg/ml) for daptomycin (table S4) (*21*). In the absence of CaCl_2_, daptomycin MICs increased substantially ranging from 42.7 to 128 μg/ml (table S4). TriPcides PS1962 and SS1045B exhibited MICs between 3.12 and 5.65 μg/ml (6 and 10 μM) for all five isolates independent of supplementation with CaCl_2_ (table S4), demonstrating that TriPcides are effective against these strains and suggesting that, although treatment with TriPcides or daptomycin results in membrane disruption, the mechanism of action differs between the antibiotics. To determine whether TriPcides enhance or interfere with the activity of front-line anti-MRSA antibiotics, we performed checkerboard assays combining SS1045B with either daptomycin [in brain heart infusion (BHI) medium supplemented with CaCl_2_ (50 μg/ml)] or linezolid (in BHI medium). Across tested concentrations, the fractional inhibitory concentration index (FICI) values ranged from 1.83 to 2.00, indicating indifferent interactions. These data suggest that SS1045B neither synergizes nor antagonizes with these front-line MRSA antibiotics in vitro (table S5).

### TriPcide treatment affects membrane integrity and increases ATP and ROS levels

As traditional approaches to understanding the mechanism of action were unavailable given our inability to generate TriPcide-resistant strains, we chose to screen a pooled transposon library to obtain genetic information that could reveal the mechanism of action. As previously published, this pooled library in MRSA strain USA300 contains loss-of-function transposon mutants as well as mutants that increase expression of genes downstream of insertions (*22*, *23*). The transposon libraries were grown in the presence of sublethal concentrations of PS1962 (1.04 μg/ml, 2 μM) and then harvested (OD_600_, 2.0) for genomic DNA extraction, library preparation, and sequencing.

**Fig. 3.**
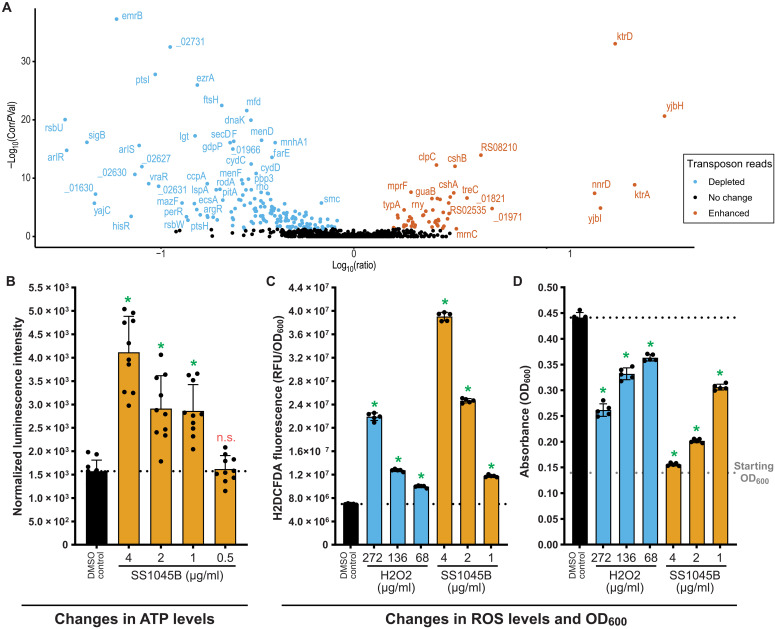
TriPcides cause increased ROS and ATP levels in MRSA-1369. (**A**) Transposon insertion mutants represented in the library following treatment relative to the vehicle control. Log_10_ ratio of insertions in the experimental condition relative to the control (*x* axis) and corrected *P* values (*y* axis) of transposon insertions in indicated genes in the PS1962 (1.04 μg/ml, 2 μM)–treated group relative to DMSO control. Genes that significantly differ in insertions relative to the control are indicated in blue (ratio < 0.5, corrected (corr.) *P* value < 0.05) and red (ratio > 2, corr. *P* value < 0.05). (**B**) Changes in ATP levels in exponential-phase MRSA-1369. The MRSA-1369 cells were treated with DMSO (vehicle control) or SS1045B (0.5 to 4 μg/ml; 0.9 to 7 μM) for 30 min, and then the ATP levels were determined using a BacTiter-Glo Microbial Cell Viability Assay kit from Promega. Bar graphs are represented as the mean and SD derived from 10 independent biological replicates. (**C**) Changes in ROS levels in exponential-phase MRSA-1369 cells incubated in BHI medium supplemented with 10 μM H2DCFDA fluorescence probe in the presence of DMSO (vehicle control), H2O2 (68 to 272 μg/ml, 2 to 8 mM), or SS1045B (1 to 4 μg/ml; 1.8 to 7 μM) at 37°C for 1 hour. ROS levels were determined by H2DCFDA fluorescence intensity normalized with corresponding OD_600_ values. (**D**) OD_600_ values of the MRSA-1369 samples shown in (C). Bar graphs in (C) and (D) are represented as means and SD from five biological replicates. Statistical analysis by ordinary one-way ANOVA test with Tukey’s comparison. Samples marked with (*) showed a statistically significant difference compared to the DMSO control (*P* ≤ 0.0001), whereas samples marked with (n.s.) were not significantly different from the DMSO control (*P* = 0.9996). RFU, relative fluorescence unit.

We identified 155 genes wherein reads due to transposon insertion were depleted in the PS1962 group relative to the vehicle control, suggesting that the gene products conferred a fitness advantage upon TriPcide treatment. Conversely, for 52 genes, transposon reads were increased, suggesting that loss of function conferred the fitness advantage (Fig. 3A and data S1). Consistent with our previous results, pathway analysis of genes depleted for insertions showed that many were involved or related to the cell membrane [fold-enrichment (FE) > 2; enrichment false discovery rate (FDR) = <1 × 10^−6^], including the top pathways for plasma membrane (75 of 620 genes, FDR = 1.73 × 10^−12^), membrane (79 of 686 genes, FDR = 1.73 × 10^−12^), and cell periphery (75 of 655 genes, FDR = 1.60 × 10^−11^). Previously published data from the same Tn-seq library treated with daptomycin (0.5 μg/ml) revealed 44 genes depleted for insertions, and the plasma membrane (23 of 620 genes, FDR = 1.78 × 10^−4^) and membrane (24 of 686 genes, FDR = 1.78 × 10^−4^) pathways were also among the top three hits, consistent with both drugs affecting the cell membrane (for complete gene pathway information, see data S1) (*23*).

The pathways with the greatest fold enrichment for genes depleted in transposon insertions upon PS1962 treatment were menaquinone biosynthesis (FE = 19.3, FDR = 1.38 × 10^−6^), biosynthesis of the menaquinone precursor chorismate (FE = 16.5, FDR = 7.72 × 10^−6^), and several pathways related to ion transport and oxidative phosphorylation (FE > 6, enrichment FDR <1 × 10^−5^) (data S1). Menaquinones have important functions as electron carriers in the electron transport chain of Gram-positive bacteria during oxidative phosphorylation and function as redox sensing molecules. Given the importance of menaquinone synthesis in the response to TriPcides, as well as ion transport and oxidative phosphorylation, we tested the effect of sublethal concentrations of SS1045B on cellular levels of ATP. We observed increased levels of ATP (1.8- to 2.6-fold) in MRSA cells treated with as little as 1 μg/ml (1.8 μM) of SS1045B after 30 min of treatment (Fig. 3B). We reasoned that the increased ATP levels may result in increased ROS as by-products of cellular respiration. Consistently, we observed that among the genes most depleted for transposon insertions in the PS1962-treated condition were *sigB* and its regulators (*rsbUW*), which is known to mediate the response to oxidative and antibiotic stressors, as well as the oxidative stress regulator *perR* (Fig. 3A). We measured ROS levels in MRSA cells treated with sublethal concentrations of SS1045B and found that at concentrations greater than 2 μg/ml (3.5 μM), SS1045B shows greater ROS activity than treatment with hydrogen peroxide (272.08 μg/ml; 8 mM) (3.5- to 5.6-fold compared to 3.1-fold), which is known to result in the formation of hydroxyl radicals via the Fenton reaction, after 60 min of treatment (Fig. 3, C and D). Thus, the Tn-seq–based approach revealed that genes involved in membrane synthesis, ATP generation, and redox stress response are all important to surviving sublethal TriPcide treatment.

### Sublethal concentrations of SS1045B decrease secretion of known *S. aureus* virulence factors and protect HeLa cells treated with *S. aureus* supernatants

**Fig. 4.**
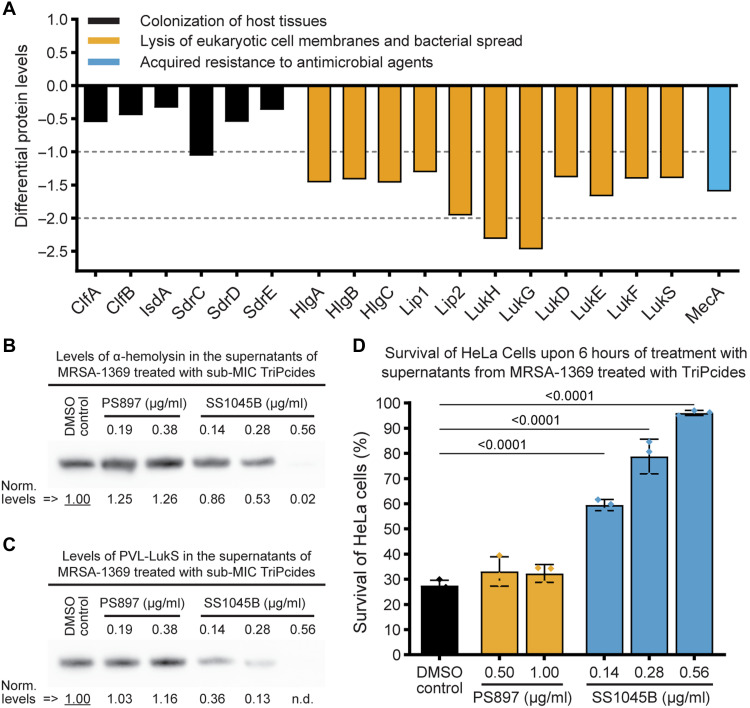
Treatment with sub-MIC SS1045B results in reduced secretion of virulence factors in MRSA-1369. (**A**) Differential protein levels of known virulence and host evasion factors in the supernatant of MRSA-1369 upon 18 hours of incubation in chemically defined medium supplemented with sub-MIC (0.28 μg/ml, 0.5 μM) SS1045B (in comparison to DMSO vehicle control). (**B**) Levels of α-hemolysin and (**C**) PVL-LukS found in the supernatants of MRSA-1369 upon 18 hours of incubation in chemically defined medium supplemented with DMSO (vehicle control), inactive compound PS897 (0.19 to 0.38 μg/ml, 0.5 to 1 μM), or sub-MIC SS1045B (0.14 to 0.56 μg/ml, 0.25 to 1 μM). Composite images (Fig. 4, B and C) are derived from single gels edited to remove unrelated lanes. (**D**) Survival of HeLa cells upon 6 hours of treatment with filtered supernatants of MRSA-1369 cultures at the end of incubation in chemically defined medium supplemented with DMSO (vehicle control), inactive PS897 (0.19 to 0.38 μg/ml, 0.5 to 1 μM), or sub-MIC SS1045B (0.14 to 0.56 μg/ml, 0.25 to 1 μM) for 18 hours. Bar graphs are represented as means from biological triplicates, and error bars indicate the SD. Statistical analysis by ordinary one-way ANOVA test with Tukey’s comparison. n.d., not determined.

Outside of *sigB* and its regulators, both genes in the two-component ArlRS system were among the most transposon-depleted genes, with ratios of 0.032 and 0.077, for relative insertions upon TriPcide treatment compared to the vehicle control (Fig. 3A). The ArlRS system regulates several key secreted virulence factors, including extracellular matrix proteins, adhesins, immune modulators, and toxins. Thus, to determine whether secreted virulence factors were affected by TriPcide treatment, mass spectrometry–based secretomics was performed on supernatants of MRSA-1369 cells treated with sublethal concentrations of SS1045B (0.28 μg/ml, 0.5 μM) or the vehicle [dimethyl sulfoxide (DMSO)] control (fig. S6, A and B; see Materials and Methods). Of the 494 secreted proteins detected, 112 showed a greater than twofold change when treated with SS1045B compared to the DMSO control, most of which (80) were down-regulated (fig. S6C). Notably, many of the factors with the most decreased abundance were those involved in the colonization of host tissues (ClfAB, IsdA, and SdrCDE) or the lysis of eukaryotic cell membranes and bacterial spread, including leukotoxins (LukGH and LukDE), gamma-hemolysin (HglAB), Panton-Valentine leukocidin (PVL) toxin (LukF-PV and LukS-PV), and α-hemolysin (Hla) (Fig. 4A). As both PVL and α-hemolysin have been shown to be important contributors in the murine SSTI model, we further tested the levels of these secreted factors via immunoblots of supernatants from OD-normalized cells treated with subinhibitory (0.14, 0.28, and 0.56 μg/ml; 0.25, 0.5, and 1 μM) concentrations of SS1045B. We observed a marked decrease in detectable levels of α-hemolysin and LukS-PV in the supernatants of SS1045B-treated cells at concentrations as low 0.14 μg/ml (0.25 μM) SS1045B, with little to no detectable protein in the presence of 0.56 μg/ml (1 μM) SS1045B (Fig. 4, B and C). There was no inhibition of virulence factor secretion observed with inactive compound PS897 at concentrations of 0.19 to 0.38 μg/ml (0.5 to 1 μM).

To test whether the SS1045B-dependent decrease in secreted MRSA virulence and host evasion factors affected host cells, we incubated HeLa cells for 6 hours with cell-free filtered supernatants harvested from MRSA cultures that were treated with the vehicle control (DMSO) or a sublethal concentration of SS1045B (0.56 μg/ml, 1 μM). Treatment with the vehicle control supernatants resulted in a marked decrease in HeLa cell viability, as determined by resazurin assay (see Materials and Methods), with less than 20 to 30% of cells surviving exposure (Fig. 4D). In contrast, HeLa cells challenged with TriPcide-derived culture supernatant showed a significant and dose-dependent increase in the survival rate as compared to the vehicle-derived supernatant. At a concentration of 0.56 μg/ml (1 μM), SS1045B greater than 95% of HeLa cells remained viable (Fig. 4D). Supernatants from PS897-treated cultures (0.19 to 0.38 μg/ml; 0.5 to 1 μM) showed similar results to the DMSO (vehicle) control. These results demonstrate that treatment of MRSA with sublethal concentrations of SS1045B markedly decreases deleterious effects on host cell viability, likely through the decreased expression of *S. aureus* secreted virulence factors.

### TriPcide SS1045B reduces ulcer size and promotes healing in murine SSTI with *S. aureus*

**Fig. 5.**
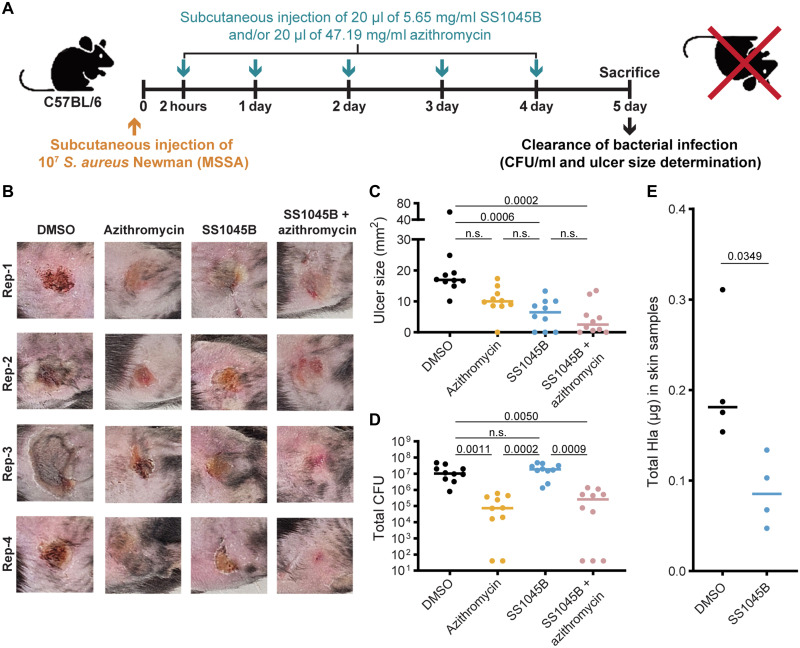
Treatment with SS1045B greatly reduces ulcer size in *S. aureus* murine SSTI model. (**A**) Timeline of the 5-day infection and treatment protocol using SS1045B and/or azithromycin to treat *S. aureus* Newman in mice. (**B** and **C**) Azithromycin, SS1045B, and combo (SS1045B + azithromycin) treatments reduced ulcer formation on day 5. (**D**) Azithromycin and combo treatments attenuated bacterial burden on day 5 but not the treatment with SS1045B. Ten mice were used for each treatment condition, and the differences between groups were tested for significance using nonparametric Kruskal-Wallis test with Dunn’s comparison. (**E**) Detection of Hla toxin in control (DMSO) and SS1045B-treated ulcer tissue homogenates. The amount of Hla from frozen tissue homogenates was measured using rabbit anti-Hla polyclonal antibody and quantified against a standard curve generated from recombinant Hla. Four mice were used for each treatment condition, and the statistical analysis was performed by unpaired *t* test with Welch’s correction. Mouse cartoon in Fig. 5A was created in BioRender. Nye, T. (2026) https://BioRender.com/rfkmr95.

Having established that subinhibitory treatment with SS1045B decreased secreted virulence factors, we next sought to establish its efficacy for treatment of a model of SSTI. In this model, infection of 1 × 10^7^ CFU of *S. aureus* Newman into the flank of a C57BL/6 mouse results in the generation of a local draining ulcer that increases in size to reach a maximum area at day 5 postinfection. Mice were infected and then received a 20-μl subcutaneous injection adjacent to the site of infection with (i) SS1045B (5.65 mg/ml stock solution in 100% DMSO), (ii) vehicle control (100% DMSO), (iii) standard-of-care antibiotic azithromycin (47.19 mg/ml stock solution in 100% DMSO), or (iv) a combination of SS1045B and azithromycin (Fig. 5A). Injections were at 2 hours postinfection and then every 24 hours over the course of 5 days. The mice tolerated the subcutaneous injections well, with no observable signs of distress or adverse effects throughout the treatment period. At the time of peak ulcer formation (day 5), the size of each ulcer was recorded, and the mice were euthanized for enumeration of ulcer CFUs. Compared to vehicle-treated mice, those treated with either SS1045B or azithromycin showed a reduction in ulcer size by ~40 and 39%, respectively. However, SS1045B was not effective in reducing bacterial burdens, showing no significant change relative to the vehicle control, whereas azithromycin achieved approximately 2 log_10_ reduction (Fig. 5, B and D). The combination of SS1045B with azithromycin resulted in significantly decreased ulcer size (~85% compared to vehicle control), with an azithromycin-like reduction in bacterial burdens (Fig. 5, B to D). Reasoning that the reduction in ulcer size may be the result of a decrease in secreted virulence factors in vivo, we performed an enzyme-linked immunosorbent assay (ELISA) assay on tissue homogenates from TriPcide-treated and untreated ulcers to determine the total amount of α-hemolysin in ulcers. Levels of α-hemolysin (Hla) in tissue have previously been shown to correlate with the degree of tissue damage in the murine SSTI model (*24*). Consistent with the reduction of Hla levels observed upon challenge with a subinhibitory concentration of TriPcides in vitro (see above), Hla levels in treated ulcers were significantly decreased (~53%, *P* = 0.0349) from the levels detected in vehicle alone–treated ulcers (Fig. 5E). These data suggest that TriPcide-induced reductions in secreted virulence factor expression contribute to the reduction of tissue damage observed in the murine model.

## DISCUSSION

To address the dire need for therapeutic strategies to combat AMR infections, we introduce a tricyclic synthetic scaffold (TriPcides) that has yet to be explored in drug discovery. The synthetic methodology to prepare tricyclic analogs is very robust, no expensive photocatalyst is needed, and the methodology accommodates a wide substrate scope. This transformation is scalable and can be performed at a late stage in the synthetic sequence. As a result, the substitution pattern around the scaffold can be efficiently modified to fine-tune the properties of TriPcides. In addition to retaining and/or improving upon the desirable properties of bicyclic GmPcides, including bactericidal activity against a range of Gram-positive pathogens in the low micromolar range, TriPcides are active against bacterial strains resistant to bicyclic GmPcides and active against 121 clinical MRSA and MSSA isolates, 109 enterococcal isolates, as well as 5 MRSA isolates that are resistant to the last resort antibiotic daptomycin.

Antibiotic tolerance, unlike resistance, allows bacteria to survive lethal antibiotic concentrations without altering the MIC often due to reduced growth rate and metabolic activity. Consequently, many antibiotics lose efficacy against dormant bacterial populations, such as those in the stationary phase. Here, we show that TriPcides effectively kill both exponential and stationary phase MRSA cells, suggesting that they overcome dormancy-associated antibiotic tolerance. We additionally demonstrate that TriPcides rapidly kill MRSA persister cells, which are a small subpopulation of dormant cells in a bacterial population that can survive high exposure to antibiotics. Many of the existing antibiotics that have been shown to be effective at killing persister Gram-positive cells have targets within the cell wall or membrane; NH125, nisin, and HT61 all cause membrane disruption (*15*, *19*, *25*, *26*), and lysostaphin hydrolyzes peptidoglycan in the cell wall (*19*). Consistent with these observations, we found that TriPcides cause rapid membrane disruption. Furthermore, when we treated a MRSA Tn-seq library with sublethal concentrations of PS1962, we found that many of the genes with decreased transposon insertions, or those that had a fitness defect upon treatment with TriPcide, were involved in pathways related to the cell membrane.

Our Tn-seq results also revealed that enriched pathways for genes depleted in transposon insertions included menaquinone biosynthesis, oxidative phosphorylation, and ion transport (Fig. 3A and data S1). Given that menaquinones have important functions as electron carriers in the electron oxidative phosphorylation and that oxidative phosphorylation and ion transport are critical to aerobic respiration, these data suggest that increased cellular respiration may be required to survive sublethal TriPcide treatment, which could effectively sensitize otherwise dormant cells to antibiotic activity. Of note, outside of membrane-targeting antibiotics, antibiotic PK150 has been shown to kill persister cells and also targets the menaquinone pathway (*17*). Together, these results suggest that TriPcides affect two of the main targets, cell membrane and menaquinone biosynthesis, that are known to kill persister cells.

In addition to the ability of TriPcides to kill dormant cell states, we also found that treatment with sublethal concentrations of SS1045B greatly reduced MRSA secreted virulence factors. Among the decreased secreted virulence factors were those involved in the lysis of eukaryotic cell membranes and bacterial spread, including HlgABC, Lip1 and Lip2, and LukDE and LukFS, and those involved in the colonization of host tissue, including ClfA, ClfB, IsdA, and SdrCDE. Supporting this observation, HeLa cells treated with supernatants from MRSA cells exposed to sublethal TriPcide showed a significantly higher survival rate compared to those treated with vehicle control supernatants. This indicates that TriPcide treatment at sublethal concentrations reduces the cytotoxic potential of MRSA by suppressing the secretion of virulence factors. Notably, previous-generation GmPcides have also been shown to inhibit virulence factors in *S. pyogenes* and *L. monocytogenes*, supporting the broader antivirulence potential of this compound class (*11*, *13*, *27*, *28*).

When TriPcides were applied to a murine SSTI, we observed ulcer healing comparable to the azithromycin-treated mice, despite observing no significant reduction in *S. aureus* ulcer CFUs with TriPcide treatment and significant reduction of bacterial burden in azithromycin-treated ulcers (Fig. 5). The healing in the absence of reduction in bacterial load could be explained by TriPcides’ effects on secreted virulence factors (e.g., α-hemolysin), which may lead to decreased bacterial colonization and spread in addition to less host cell lysis. This may represent a promising therapeutic avenue, as there could be less selective pressure against TriPcides that reduce the virulence capabilities but do not kill bacterial cells. More frequent dosing of TriPcides may be required to increase bactericidal activity in vivo, and future pharmacokinetic studies will be necessary to determine appropriate dosing regimens. In addition, TriPcides could be used in combination with other bactericidal antibiotics to target both the secreted virulence factors, reducing bacterial spread and promoting ulcer healing, and reduce the bacterial burdens at the site of infection. We note that the previous generation of GmPcides exhibited synergistic killing when combined with several standard-of-care antibiotics with various cellular targets (*12*). We are also actively generating libraries of TriPcide analogs that have higher antibacterial activity as candidates to improve killing activity in vivo*.*

The synthetic methodologies developed herein for synthesis of TriPcides allow for large-scale synthesis without expensive catalysts and for robust and efficient modifications around the scaffold. Given their robust antibacterial activities against actively dividing and nondividing cells, as well as the lack of detectable resistance among MDR Gram-positive pathogens, TriPcides have strong therapeutic potential for translation to the clinical setting.

## MATERIALS AND METHODS

### Experimental procedure for [2 + 2] photo-cycloaddition reaction

Thiazolo-2-pyridone of general structure 1 (0.25 mmol) was charged into a glass vial and added 2.5 ml of dichloromethane, followed by the addition of styrene (4 eq). The reaction mixture was irradiated under purple light-emitting diode at 395 nm after degassing with nitrogen for 3 to 5 min. After completion of reaction as indicated by thin-layer chromatography, the solvent was removed under reduced pressure, and the crude product was purified by column chromatography using Biotage isolera (25-g cartridge; heptane/EtOAc, 0 to 100%).

### General procedure for the hydrolysis: Synthesis of TriPcides

Methyl esters of general structure 2 or 3 (0.24 mmol) was charged into a round-bottom flask and added tetrahydrofuran (2 ml). Lithium hydroxide (1 M, aqueous 0.97 mmol) was added to the reaction mixture and stirred at room temperature for 16 hours. The reaction mixture was neutralized with 1 M HCl and extracted with ethyl acetate (15 ml × 2). Combined organic layers were dried over anhydrous Na_2_SO_4_ and evaporated under reduced pressure to yield the crude product. The residue was redissolved in DMSO (1 ml) and purified with preparative high-performance liquid chromatography (HPLC; H_2_O/acetonitrile + 0.75% HCOOH, 20 to 100% in 45 min, 100% in 10 min). The pure product was diluted with water (10 ml) and freeze-dried.

The synthesized compounds were characterized using nuclear magnetic resonance, infrared, and mass spectrometry. The analytical data are included in the Supplementary Materials (figs. S7 to S34) (*29*, *30*).

### Bacterial strains and growth conditions

Unless otherwise specified, all bacterial cultures were inoculated in BHI medium (BD and Company) or on BHI agar plates at 37°C. Overnight MRSA-1369 cultures were started by suspension of individual colonies from BHI agar into BHI medium followed by shaking incubation at 37°C. These cultures were then back-diluted (1000-fold) in BHI medium to achieve the exponential-phase and stationary-phase MRSA-1369 cultures. The exponential-phase MRSA-1369 cultures were collected at the end of 3 hours of shaking incubation at 37°C, and stationary-phase MRSA-1369 cultures were collected at the end of 18 hours of shaking incubation at 37°C. Ciprofloxacin-persister MRSA-1369 cells were prepared as previously described in (*17*). Briefly, late-stationary phase (24-hour-old) MRSA-1369 cultures were treated with ciprofloxacin (50 μg/ml; 100× MIC) for an additional 24 hours of shaking at 37°C. Gentamicin-persister MRSA-1369 cells were prepared as previously described in (*19*). Briefly, late-stationary phase (24-hour-old) MRSA-1369 cultures were treated with ciprofloxacin (24 μg/ml; 200× MIC) for an additional 4 hours of shaking at 37°C. Other growth conditions and concentrations of the drugs are described in the main text or below. Bacterial strains used in this study are listed in table S6 (*31*–*37*).

### Determination of MIC and MBC

MIC and MBC assays were performed as previously described (*12*). Briefly, MIC and MBC were determined following the Clinical and Laboratory Standards Institute (CLSI) method in BHI medium unless otherwise indicated (*38*). Accordingly, the MIC was defined as the lowest concentration of a compound that prevented growth, measured by OD_600_ after values were normalized to the DMSO (vehicle) control, using a 20% cutoff relative to the control. The MBC was determined as the concentration that gave a ≥3 log_10_ decrease in CFUs relative to the inoculum. MIC and MBC values for experimental compounds were rejected if the MIC and MBC values of quality strains tested against a standardized panel of antibiotics were outside of CLSI determined ranges.

### Continuous exposure assay

CEA used in this study was adapted from (*16*). Overnight MRSA-1369 cultures were diluted 100-fold in round-bottom clear 96-well plates containing serial dilutions of bicyclic GmPcide (PS900), tricyclic TriPcides (NQA8 and PS1962), or antibiotics (ciprofloxacin and vancomycin) and then incubated at 37°C shaking for 24 hours. The growth was determined by OD_600_ measurements at the end of the 24 hours of incubation. For each treatment, an aliquot was taken from the highest concentration of treatment that allowed ≥70% growth compared to untreated control, diluted 100-fold, and passaged into a new plate containing the same corresponding treatment (three biological replicates for each treatment condition). This process was repeated every day for 14 days (passages). GmPcide, TriPcide, and antibiotic concentrations were adjusted for each passage as the samples developed resistance, and experiments were terminated once the samples became resistant to 32 to 64× the MIC of MRSA-1369.

### Isolation and identification of PS900-resistant MRSA-1369 mutants

PS900-resistant MRSA-1369 mutants were selected at the end of the CEA, as described above. These mutants were reisolated as a single colony on BHI agar plate, and their resistance to PS900 was confirmed with a standard MIC/MBC assay as described above. Overnight cultures of MRSA-1369 and PS900-resistant isolates HT110, HT114, and HT115 were grown in BHI medium shaking at 37°C. The cells were harvested via centrifugation, and pellets were stored at −80°C. DNA libraries from these samples were prepared and sequenced as previously described in (*12*). Sequencing reads were deposited to the National Center for Biotechnology Information Sequence Read Archive (SRA) database and can be accessed under BioProject ID PRJNA1268509.

### Cytotoxicity assay

HeLa 229 cells [CCL-2.1; American Type Culture Collection (ATCC)] were maintained and passaged at 37°C, 5% CO_2_, in RPMI 1640 medium (52400-025, Gibco) supplemented with 10% fetal bovine serum (FBS) (F7524, Sigma-Aldrich). HepG2 cells (HB-8065; ATCC) were maintained and passaged at 37°C, 5% CO_2_ in Dulbecco’s modified Eagle’s medium–high glucose (D6429, Sigma-Aldrich) supplemented with 10% FBS (F7524, Sigma-Aldrich) media. For the neutral red (NR) cytotoxicity assay, HeLa and HepG2 cells were seeded in 96-well plates and left to adhere overnight. For screening of TriPcide cytotoxicity, HeLa cells were thereafter treated in triplicate with 10, 20, 40, and 80 μM compound. To determine median inhibitory concentration, cytotoxicity of selected TriPcides was examined on both HeLa and HepG2 cells using a 10-point dose-response curve ranging from 100 to 7.5 μM using 1.33× dilution steps in duplicate. The TriPcides were first dissolved in 100% DMSO (Sigma-Aldrich) and then further diluted in complete media before they were added to the wells in 96-well plates. After 48 hours of treatment, the cells were washed with Dulbecco’s PBS (DPBS) (CaCl_2_ and MgCl_2_) (Gibco) and thereafter incubated with complete media containing NR (Sigma-Aldrich) for 3 hours. The cells were subsequently washed with DPBS (CaCl_2_ and MgCl_2_), the dye was extracted with NR desorb (1% glacial acetic acid, 50% ethanol, and 49% H_2_O), and absorbance was measured at 540 nm. Averages and SDs of samples were calculated, and the percentage of viable cells compared to the DMSO control sample was determined.

### Hemolysis assay

Red blood cells (RBCs) for the hemolysis assay were prepared from heparinized blood samples from three healthy individuals (~10 ml each). These blood samples were spun down at 3000*g* for 10 min at room temperature. Serum from each sample was discarded, RBCs were washed thrice with equal volume of Tyrode buffer (130 mM NaCl, 4 mM KCl, 2.8 mM NaOAc, 1 mM MgCl_2_, 10 mM glucose, 1 mM CaCl_2_, and 10 mM HEPES, pH adjusted to 7.4 with NaOH). The RBCs in Tyrode buffer were pooled together and labeled as 100% RBCs. A total of 10 mM GmPcide stocks in DMSO were diluted to 200 μM concentration in Tyrode buffer and then mixed with 100% RBCs, resulting in 100 μM final GmPcide concentration in 96-well plates (200-μl final volume). The plates were incubated at 37°C shaking (at 250 rpm) for 45 min and then spun down at 2653*g* for 10 min at room temperature. Eighty microliters of supernatants was transferred into new 96-well plates, and the levels of hemolysis were determined by absorbance measurements at 540 nm. Absorbance values of each GmPcide treatment minus the average absorbance value from negative controls (DMSO only) were normalized to average absorbance value from positive controls (2% Triton X). The final hemolysis activity for each GmPcide was determined as the median value of five independent replicates.

### Kinetic solubility

Kinetic solubility of PS1962, SS1040B, and SS1045B compounds was determined in 100 mM potassium phosphate buffer solution (pH 7.4) containing 1% DMSO. The samples were incubated at 37°C for 24 hours of shaking at 1000 rpm. The samples were then rapidly filtered through a 0.45 μM polytetrafluoroethylene (PTFE) membrane filter into amber glass autosampler vials, and the concentration of dissolved compounds was quantified by HPLC analysis (37°C).

### Determination of MIC distribution versus clinical isolates

MIC and MIC90 values were determined according to the Clinical Laboratory Standards Institute guidelines using recent European clinical isolates of *S. aureus* (121 isolates) and enterococcal species (109 isolates) as previously described (*39*). These experiments were conducted as part of the Enable-2 Consortium.

### Growth curves and killing of exponential-phase cultures

The stationary-phase MRSA-1369 cell cultures (4.6 to 5.1 OD_600_ in BHI medium) were diluted to 0.01 OD_600_ in BHI medium and aliquoted into the wells of 96-well plates (190 μl culture per well). The samples were incubated in the absence of vehicle control or SS1045B for 2 hours of shaking at 37°C. Then, 10 μl of vehicle control (DMSO) or SS1045B (1.25 to 20 μM final concentration) was added to corresponding wells. The samples were then incubated shaking at 37°C for 24 hours. The growth of the samples was monitored by OD_600_ measurements every 5 min in a SpectraMax iD3 plate reader. At t2 (before treatment) and t26 hours (posttreatment), samples were diluted and spotted on BHI agar plates followed by overnight incubation at 37°C to determine CFUs.

### Killing of stationary phase cultures

The stationary-phase MRSA-1369 cell cultures (5.2 to 5.5 OD_600_ in BHI medium) were aliquoted into the wells of 96-well plates (192-μl cultures per well), and 8 μl of vehicle control (DMSO) or SS1045B (10 to 80 μM final concentration) was added to corresponding wells. The samples were then incubated shaking at 37°C for 24 hours. At t0 (before treatment) and t24 hours (posttreatment), samples were diluted and spotted on BHI agar plates followed by overnight incubation at 37°C to determine CFUs.

### GelRed membrane disruption assays

The exponential-phase, stationary-phase, or persister MRSA-1369 cultures (as described above) were washed twice with 1× PBS and adjusted to OD_600_ 0.8 in 1× PBS buffer supplemented with 4× membrane-impermeable GelRed (diluted from 10,000× original stock) and aliquoted into a clear flat-bottom black 96-well plate (Brand, 781971). Background fluorescence from the samples was monitored for 8 to 9 min at 300-nm excitation and 595-nm emission using a SpectraMax iD3 plate reader. Then, equal volumes of 2× concentration of vehicle control, SDS, GmPcides, TriPcides, or antibiotics were added to corresponding wells, and membrane disruption was monitored for 30 min via fluorescence measurements at 300-nm excitation and 595-nm emission. In the beginning and end of the treatments, samples were diluted and spotted on BHI agar plates followed by overnight incubation at 37°C to determine CFUs.

### Transposon sequencing of USA300 treated with PS1962

An aliquot of transposon library of *S. aureus* strain USA300-TCH1516 (*23*) was thawed on ice, then diluted in 10 ml of BHI medium to OD_600_ = 5, and incubated at 30°C with shaking at 250 rpm for 1 hour. The number of colony-forming units per milliliter was determined to be 8 × 10^8^ by plating dilutions of the library on BHI agar and counting colonies after incubating at 30°C overnight. In a 250-ml Erlenmeyer flask, the library was diluted 1:1000 into 62.5 ml (5 × 10^7^ CFUs, ~200× coverage with respect to total possible TA insertion sites) of BHI either with DMSO [final, 0.02% (v/v)] alone or with PS1962 dissolved in DMSO at a subinhibitory concentration of 2.0 μM. The cultures were incubated at 30°C with shaking at 250 rpm. Growth was monitored until OD_600_ for each culture reached ~2.0, at which point cells were collected by centrifugation at 3.22 k × *g* for 10 min in a swinging bucket rotor. Pellets were frozen in liquid nitrogen. Genomic DNA was extracted and processed to capture transposon junctions as described previously (*22*), with the following modifications.

Previously, the HiSeq 2500 Sequencing System was used for next-generation sequencing for transposon sequencing analysis (*23*). Because of the discontinuation of reagents sold necessary for utilization of this technology, experimental procedures were updated to accommodate commissioned Illumina platforms. The polymerase chain reaction (PCR) primers used previously (Tm199 and LIB_PCR_3), which amplify the final PCR product while incorporating Illumina flow cell adaptors, were modified to enable efficient binding to commissioned flow cells. Tm199 and LIB_PCR_3 were replaced with TM1178 (P5_UniversalAdaptor_AM; 5′-AATGATACGGCGACCACCGAGATCTACACTCTTTCCCTACACGACGCTCTTCCGATCT-3′) and TM1154 (P7_Adaptor_AM; 5′-CAAGCAGAAGACGGCATACGAGATAGACCACGCGTGCCATAAC-3′). In addition to this change, the library adaptors, which include the indexing barcode, were modified. The previously named “LIB_AdaptT/B_long” were replaced with the adaptors listed in Table 1.

**Table 1.** Oligonucleotides used as the library adapter.

Sample	Name	Sequence*
DMSO treated	ADAPT_1A	TTCCCTACACGACGCTCTTCCGATCT**GTCGGTAA**NN
	ADAPT_1B	**TTACCGAC**AGATCGGAAGAGCGTCGTGTAGGGAA
2.0 μM PS1962	ADAPT_9A	TTCCCTACACGACGCTCTTCCGATCT**TAGTTGCG**tcNN
	ADAPT_9B	ga**CGCAACTA**AGATCGGAAGAGCGTCGTGTAGGGAA

* Bold represents the indexing sequence. NN represents randomized sequence for MmeI ligation. Noncapitalized nucleotides are additional bases added following the index, implementing phase amplicon sequencing (*22*).

Next-generation sequencing was performed with a P1 XLEAP-SBS kit using the NextSeq 1000 Sequencing System (Illumina). Transposon sequencing data analysis was performed as described (*23*). Sequencing reads were mapped to TA dinucleotide transposon insertion sites in the USA300-TCH1516 genome. The number of mapped reads at each site was compared for each PS1962 treatment concentration to the DMSO alone control library. Genes that were depleted or enriched under each condition were identified by Mann-Whitney *U* tests (data S1), with *P* values corrected using the Benjamini-Hochberg procedure (data S1) (*40*, *41*). Sequencing data are available under BioProject: PRJNA1266636. Scripts can be acquired from GitHub (https://github.com/SuzanneWalkerLab/5SATnSeq).

To perform downstream analysis, USA300 TCH1516 locus tags were matched with the equivalent from reference strain NCTC8325 (data S1). Gene names were assigned according to the NCTC8325 locus tags published on AureoWiki (*42*). Pathway analysis was performed using ShinyGO 0.82 with the *S. aureus subsp. aureus* NCTC8325 database with default settings (*43*). For the comparison with daptomycin, pathway analysis was performed on the dataset generated from treated libraries (0.5 mg/ml) in MHB2 media (*23*) using NCTC8325 matched locus tags.

### Determination of changes in ROS levels

Changes in ROS levels in the exponential-phase MRSA-1369 cells were determined by using cell-permeant 2′,7′-dichlorodihydrofluorescein diacetate (H2DCFDA) fluorescence probe adapted from (*44*). The exponential-phase MRSA-1369 cells were diluted to 0.4 OD_600_ in BHI medium supplemented with 10 μM H2DCFDA (Sigma-Aldrich, D6883) in the presence of vehicle control (DMSO), H2O2 (2 to 8 mM), or SS1045B (1 to 4 μg/ml) in a clear flat-bottom black 96-well plate (Brand, 781971) followed by shaking incubation at 37°C for 1 hour. At the end of incubation, H2DCFDA fluorescence measurements at 485-nm excitation and 535-nm emission were obtained using a SpectraMax iD3 plate reader, and the fluorescence levels were normalized to OD_600_ measurements to determine the changes in the ROS levels.

### Determination of changes in ATP levels

Changes in ATP levels in exponential-phase MRSA-1369 cells in the presence of vehicle control (DMSO) or SS1045B (0.5 to 4 μg/ml) were determined by using a BacTiter-Glo Microbial Cell Viability Assay kit from Promega (catalog no. G8230) according to the manufacturer’s guidelines. The exponential-phase MRSA-1369 cells were diluted to 0.1 OD_600_ in BHI medium in the presence of vehicle control (DMSO) or varying concentrations of SS1045B in a clear flat-bottom black 96-well plate (Brand, 781971) followed by incubation in the dark for 5 min. The ATP levels were then determined by luminescence measurements at 560 nm using a SpectraMax iD3 plate reader.

### Secretomic sample preparation

MRSA-1369 strain was cultivated for 18 hours in chemically defined medium containing amino acids, salts, and glucose, as previously described (*45*) in the presence of sub-MIC (0.5 μM) compounds. The proteins in 10 ml of filtered supernatants from these samples were precipitated with TCA, washed with 80% acetone, and then suspended with 100 μl of 2% SDS followed by boiling for 10 min. Samples (5 μg) were then digested into peptides using a modified SP3 protocol (*46*, *47*). Briefly, SpeedBeads magnetic carboxylate modified particles (Sigma-Aldrich, beads A hydrophylic, catalog no. GE45152105050250; beads B hydrophobic, catalog no. GE65152105050250) were combined with ratio 1:1 (v/v) and washed using liquid chromatography–mass spectrometry (LC-MS) water four times. Then, the beads were mixed with each sample in binding buffer (50% ethanol and 2.5% formic acid in final) and incubated with shaking at 500 rpm for 15 min at room temperature. Then, they were transferred into a filter plate (0.22 μm, Sigma-Aldrich, part no: MSGVN2210). Unbound fractions were removed by centrifugation at 1000*g*. Beads were retained on the filter and washed with 70% ethanol four times. Trypsin was mixed with digestion solution [100 mM HEPES (pH 7.5), 5 mM chloroacetamide, and 1.2 mM tris(2-carboxyethyl)phosphine (TCEP)] and added to each sample (0.2-μg trypsin) on the plate. Samples were digested overnight at room temperature with shaking at 500 rpm. Flowthrough containing peptides was collected with centrifugation at 1000*g*. Ten microliters of 2% DMSO was added to beads for eluting any remaining peptides and pooled with the previous eluates. Eluates were dried and reconstituted by 10 μl of H_2_O (LC-MS grade). TMTpro (Thermo Fisher Scientific, A44522) was used for labeling each sample according to the manufacturer’s protocol. Then, all samples were pooled and desalted in an Oasis hydrophilic-lipophilic balance (HLB) plate (Waters, 186001828BA) and dried in a SpeedVac concentrator.

### Liquid chromatography–tandem mass spectrometry

Dried peptide samples were dissolved with 20 μl of 0.1% formic acid in water. Three microliters of peptides from each sample was injected into the MS using the Vanquish Neo (Thermo Fisher Scientific). The trapping column was PEPMAP NEO C18 (5-μm particle size, 300 μm by 5 mm; Thermo Fisher Scientific). Analytical column was nanoEase M/Z HSS C18 T3 (100 Å, 1.8-μm particle size, 75 μm by 250 mm; Waters). Total gradient length was 90 min for separation, and elution started with 98% mobile phase A (water and 0.1% formic acid) and 2% B (80% acetonitrile and 0.1% formic acid), then B rose to 5% over 0.5 min, and then to 9.5% in 1 min. After that, it rose to 28% over 70 min and then to 42% in 7.5 min. After that, B increased to 80% in 0.1 min and held for 4 min, lastly to 2% in 0.5 min; last, column equilibration was performed.

Data acquisition on Exploris 480 (Thermo Fisher Scientific) was carried out using a data-dependent method. Survey scans covering the mass range of 375 to 1500 were acquired at a resolution of 60,000, RF lens of 40%, and normalized automatic gain control (AGC) of 300%. Maximum cycling time of 2 s was used to control the number of precursors for tandem MS/MS (MS2) analysis. Charge states included two to six charges. Dynamic exclusion was set to exclude the previously selected precursors for 35 s. MS2 scans were acquired at a resolution of 45,000 [at mass/charge ratio (*m/z*) 200], with normalized AGC target value of 200%. The isolation window was 0.7 *m/z*. HCD fragmentation was induced with a normalized collision energy of 32. Isotopes were excluded for MS2 analysis.

Raw data were converted into mzML files by msConvert. Then, they were searched against the genome of *S. aureus* (CP055225) UniProt FASTA using FragPipe (version 18), and quantification was achieved using TMT16 workflow. Proteins identified from contaminants and decoys were removed. R (version 4.2.2) was used for statistical analysis and volcano plots. Data was normalized using vsn package (*48*). Protein differential expressions were evaluated using the limma package.

### Western blotting

MRSA-1369 strain was cultivated for 18 hours in chemically defined medium (*45*) with DMSO (vehicle control) or sub-MIC SS1045B (0.25 to 1.0 μM). The proteins in 10 ml of filtered supernatants from these samples were precipitated with TCA, washed with 80% acetone, and then suspended with 100 μl of 2% SDS followed by boiling for 10 min. As follows, 10 μl of boiled protein samples was mixed with 90 μl of TCA-Laemmli loading buffer and stored at −20°C.

NuPAGE SDS–polyacrylamide gel electrophoresis gel and blotting system was used according to the manufacturer’s instructions (Invitrogen) together with Immobilon Membrane FL polyvinylidene difluoride membrane (Merck Millipore). The Precision Plus Protein Kaleidoscope Prestained protein Standard (Bio-Rad) was used as a molecular mass marker. Immunoblots were probed with either the primary antibody anti–α-hemolysin antibody (8B7) N-terminal (AB190467-1001, Abcam) followed by goat anti-mouse immunoglobulin G (IgG) H&L [horseradish peroxidase (HRP)] (AB205710-1001, Abcam) or the primary antibody anti-PVL LukS antibody (AB190473-1001, Abcam) followed by goat anti-rabbit IgG H&L (HRP) (AB205718-1001, Abcam). Bound antibodies were detected with SuperSignal West Dura Extended Duration Substrate (Thermo Fisher Scientific).

### Killing of HeLa cells with MRSA-1369 supernatants

HeLa cells were seeded in the 96-well plate (1.8 × 10^4^ cells per well; 100-μl volume) in RPMI 1460 medium supplemented with 10% FBS and incubated overnight at 37°C with 5% CO_2_. The supernatants from MRSA-1369 cultures in chemically defined medium (*45*) supplemented with vehicle control (DMSO) or SS1045B (sub-MIC: 0.25 to 1.00 μM) were collected and filtered at the end of 18-hour shaking incubation at 37°C, and 80 μl of filtered supernatants was added to corresponding wells containing seeded HeLa cells in 100 μl of RPMI 1460 medium supplemented with 10% FBS followed by incubation at 37°C with 5% CO_2_ for 6 hours. To determine the viability of the HeLa cells, 20 μl of 400 mM resazurin was added into each well followed by additional incubation at 37°C with 5% CO_2_ for 2 to 3 hours. The viability of HeLa cells was then determined by conversion of resazurin to resorufin (fluorescent at 555-nm excitation and 585-nm emission) using a SpectraMax iD3 plate reader. Viability results were presented as percentage viability relative to untreated control samples.

### Subcutaneous mouse infection and GmPcide treatment

Subcutaneous murine infection was conducted as previously described (*11*, *49*) with the following modifications for staphylococci: To prepare the inoculum, an overnight culture of *S. aureus* Newman in trypticase soy broth (TSB) was diluted 1:100 with fresh TSB and grown at 37°C with shaking to an OD_600_ of 0.5. Staphylococci were harvested by centrifugation, washed once with an equal volume of PBS, and then suspended in PBS to density of 1 × 10^7^ CFU in 50 μl, which was confirmed by enumeration of CFUs by serial dilution and plating on tryptic soy agar. Using 8-week-old female C57BL/6 mice (Charles River NIC Grantee), 50 μl was injected subcutaneously into the shaved left thigh of each mouse. Treatments consisted of a 20-μl subcutaneous injection adjacent to the site of infection of (i) vehicle (DMSO), (ii) azithromycin (7.19 mg/ml stock solution; Sigma-Aldrich), (iii) GmPcide SS1045B (10 mM stock solution in DMSO), or (iv) a mixture of azithromycin and SS1045B (in DMSO) at 2 hours postinfection and again on days 1, 2, 3, and 4. On day 5, mice were euthanized, and ulcer sizes were quantified by digital photography and ImageJ software (*50*). Ulcers were resected and homogenized for determination of CFUs as described (*50*). As is standard, total CFUs recovered from each lesion are reported, since ulcer area and CFUs recovered are not necessarily correlated (*11*, *51*, *52*). Differences in ulcer size and recovered CFUs were tested for significance using a nonparametric Kruskal-Wallis test with Dunn’s comparison with *P* < 0.05 considered significant. All animal experimentation in this study was conducted following the National Institutes of Health guidelines for housing and care of laboratory animals and performed in accordance with institutional regulations after pertinent review and approval by the Animal Studies Committee at Washington University School of Medicine (protocol #22-0307).

### Hla detection in tissue samples

Hla detection in tissue samples was conducted as previously described (*53*–*55*) with the following modifications: Frozen, homogenized ulcer tissue samples were thawed and diluted to protein (1 mg/ml) in PBS plus a protease inhibitor (Roche). The concentration of Hla present in each sample was determined by plating 100 μg of sample onto a Maxisorp microtiter plate (Thermo Fisher Scientific) coated with monoclonal capture antibody 7B8 at 1 μg/ml, a rabbit polyclonal anti-Hla detection antibody (1:2500), and HRP-conjugated goat anti-rabbit IgG (1:4000, Cell Signaling). The amount of HLA (micrograms) present per ulcer was calculated by taking the concentration of Hla in the sample determined by an ELISA and multiplying it by the dilution factor of the sample diluted at 1 mg/ml as previously described (*53*). Differences in Hla concentration were tested for significance using an unpaired *t* test with Welch’s correction.

### Assessment of antimicrobial synergy

Synergy between antimicrobial agents was evaluated using the checkerboard assay, in which two compounds were tested across intersecting twofold dilution gradients in a 96-well plate (*56*). The interaction between the antimicrobial compounds was classified as synergistic, antagonistic, or indifferent based on the FICI. For each concentration of a compound, the FIC value was calculated as the MIC in combination divided by the MIC when tested alone. The FICI was derived by summing the individual FICs. Interactions were defined as antagonistic when FICI > 2, indifferent when 0.5 < FICI ≤ 4, and synergistic when FICI ≤ 0.5. Control conditions included single compound gradients and untreated cultures.
