# Supplementary material for: Tunable TriPcides suppress virulence factor secretion during Staphylococcus aureus infection and kill dormant cells
Source: Sci Adv. 2026 May 6;12(19):eaec9100. doi: 10.1126/sciadv.aec9100 (PMC13148309; doi:10.1126/sciadv.aec9100)
Supplement: Supplementary file 1 — Figs. S1 to S36 Tables S1 to S6 Legend for data S1 [file sciadv.aec9100_sm.pdf]

Science Advances
AAAS

Supplementary Materials for

Tunable TriPcides suppress virulence factor secretion during *Staphylococcus aureus* infection and kill dormant cells

Hasan Tükenmez *et al.*

Corresponding author: Michael G. Caparon, caparon@wustl.edu; Fredrik Almqvist, fredrik.almqvist@umu.se; Scott J. Hultgren, hultgren@wustl.edu

Sci. Adv. **12**, eaec9100 (2026)
DOI: 10.1126/sciadv.aec9100

The PDF file includes:

Figs. S1 to S36
Tables S1 to S6
Legend for data S1

Other Supplementary Material for this manuscript includes the following:

Data S1

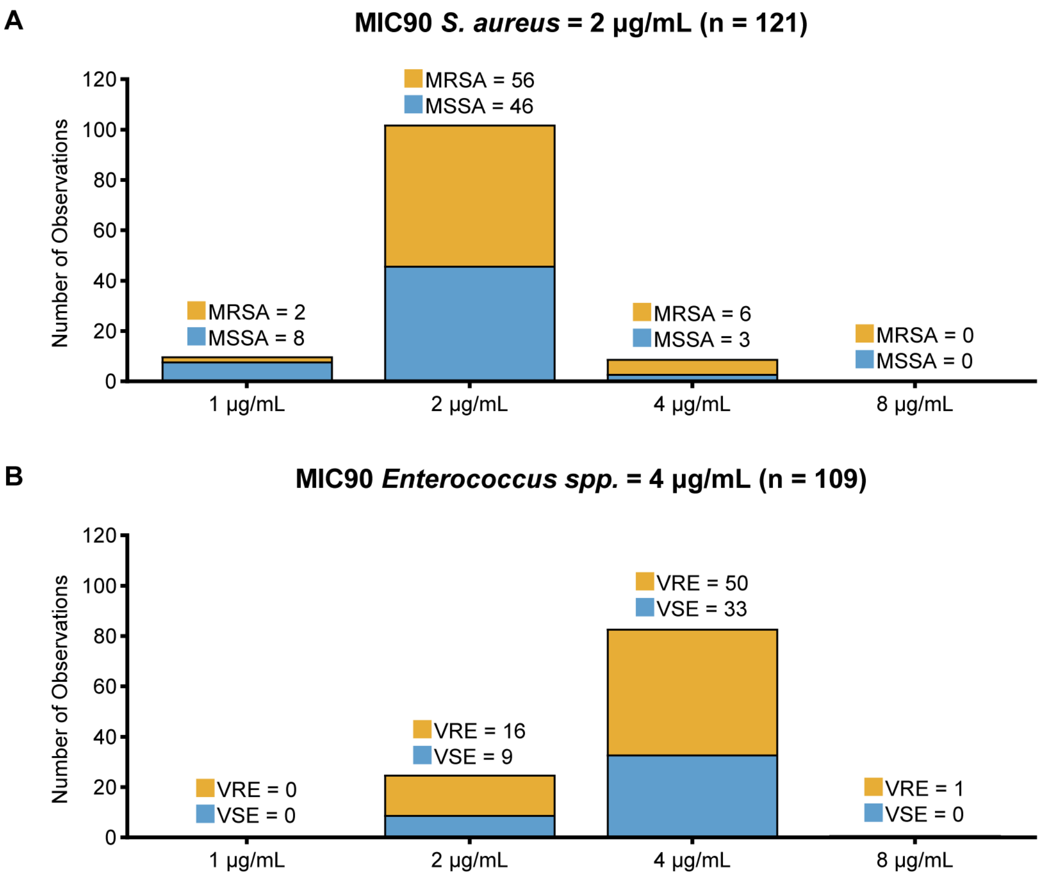

Fig. S1. No signs of pre-existing reduced susceptibility to SS1045B in a set of recent clinical isolates. MIC90 for SS1045B against clinical isolates of *S. aureus* **(A)** and enterococcal species **(B)** with different resistance profiles (MRSA: methicillin-resistant *Staphylococcus aureus*; MSSA: methicillin-resistant *Staphylococcus aureus*; VRE: vancomycin-resistant enterococci; VSE: vancomycin-sensitive enterococci).

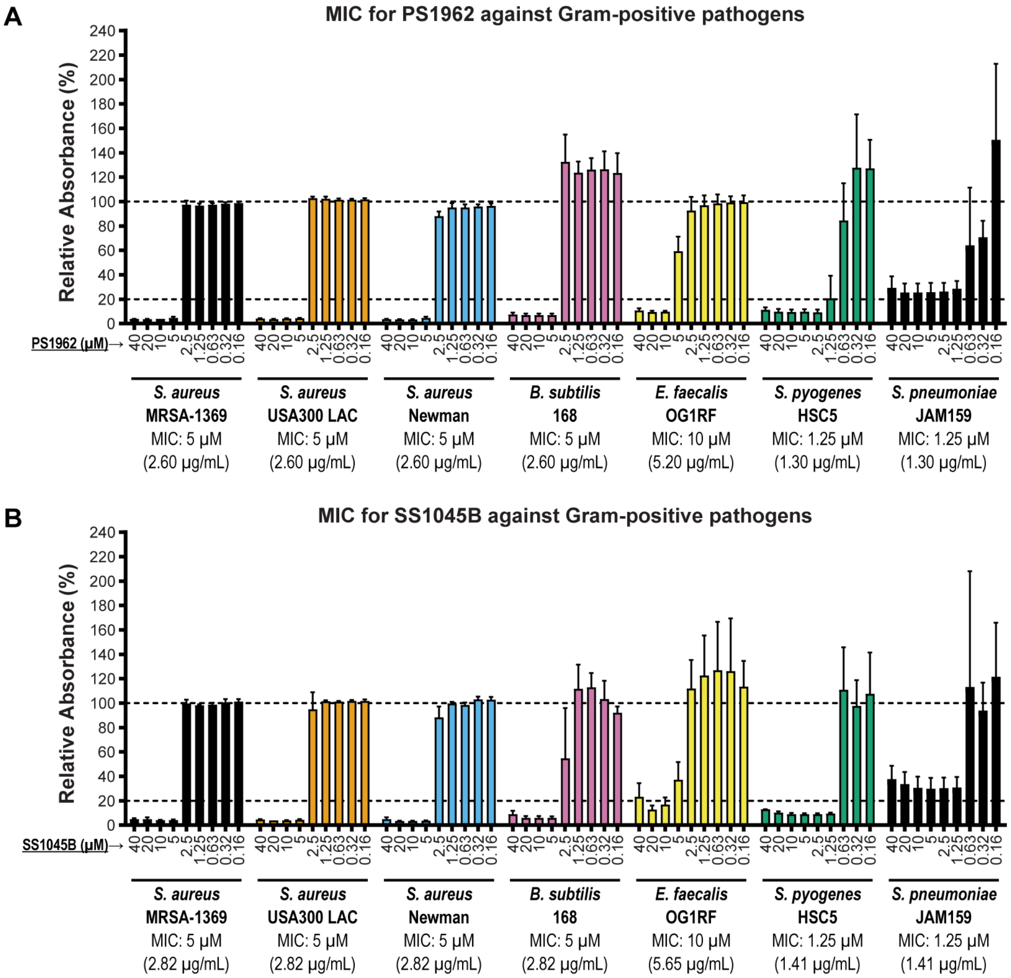

Fig. S2. PS1962 and SS1045B are effective against a variety of Gram-positive pathogens. MICs for PS900 **(A)** and PS1962 **(B)** were determined by OD_{600} measurements at the end of 24-hour shaking incubation at 37°C in BHI-medium in presence of varying concentrations (0.16-40 µM) PS900 or PS1962. OD_{600} values from each treatment were normalized to the mean OD_{600} of the DMSO (vehicle) controls. The normalized data are presented as relative absorbance (%). Bar graphs are represented as means from triplicates and error bars indicate the standard deviation.

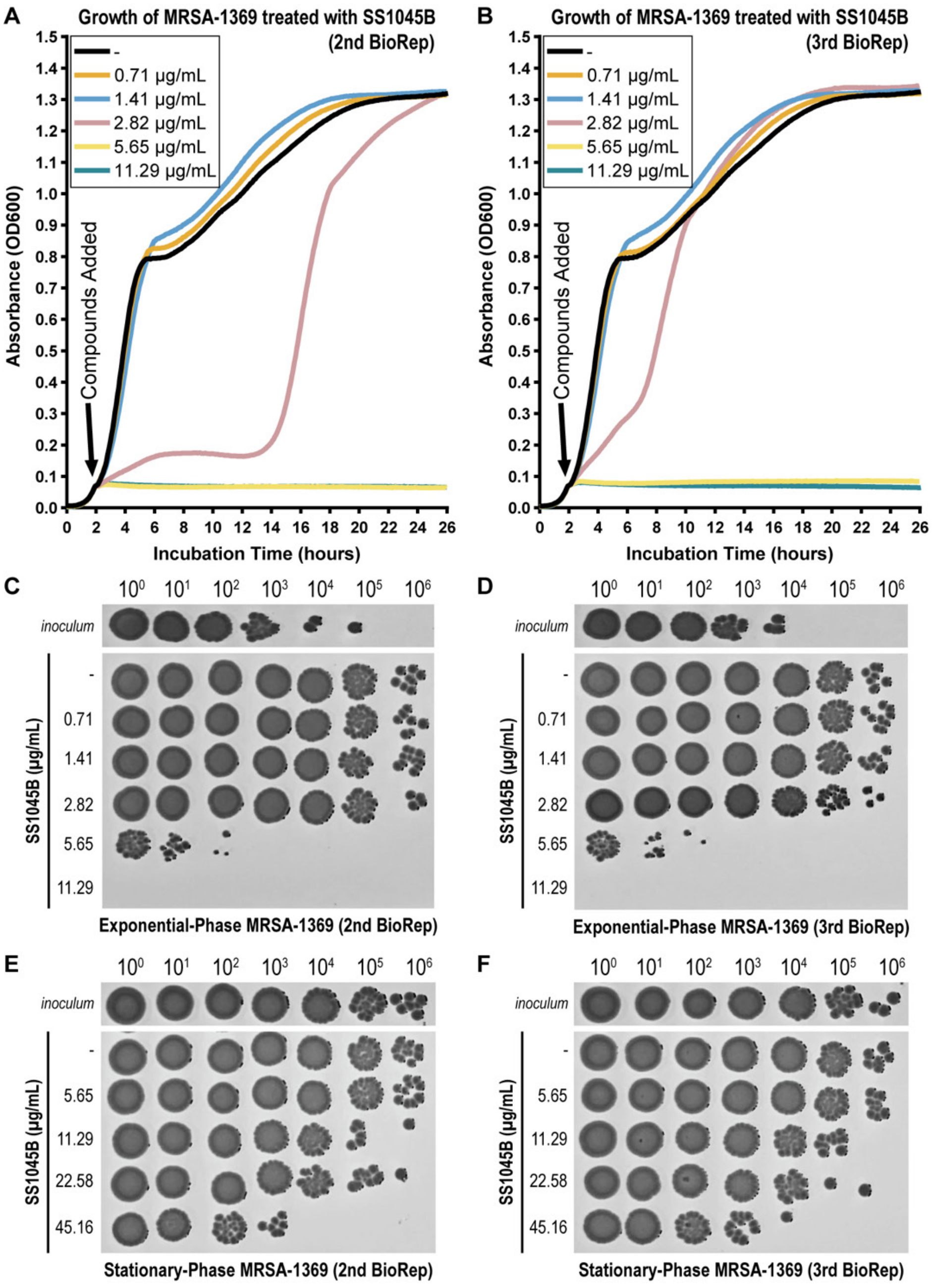

Fig. S3. SS1045B kills exponential and stationary MRSA-1369 cells. (A-B) Growth of exponential phase MRSA-1369 cultures in BHI-medium in presence of 0.71 to 11.29 µg/mL (1.25 to 20 µM) of SS1045B. MRSA-1369 cultures were started in the absence of SS1045B at 37°C. At 2 h time point, corresponding concentrations of SS1045B were added to each culture and the growth (OD_{600}) was monitored for 24 hours at 37°C. **(C-D)** Spot titer assay of exponential phase MRSA-1369 cultures at t2 (inoculum; prior to treatment) and t26 hours (post-treatment). **(E-F)** Spot titer assay of stationary phase MRSA-1369 cultures at t0 (inoculum; prior to treatment) and t24 hours (post-treatment).

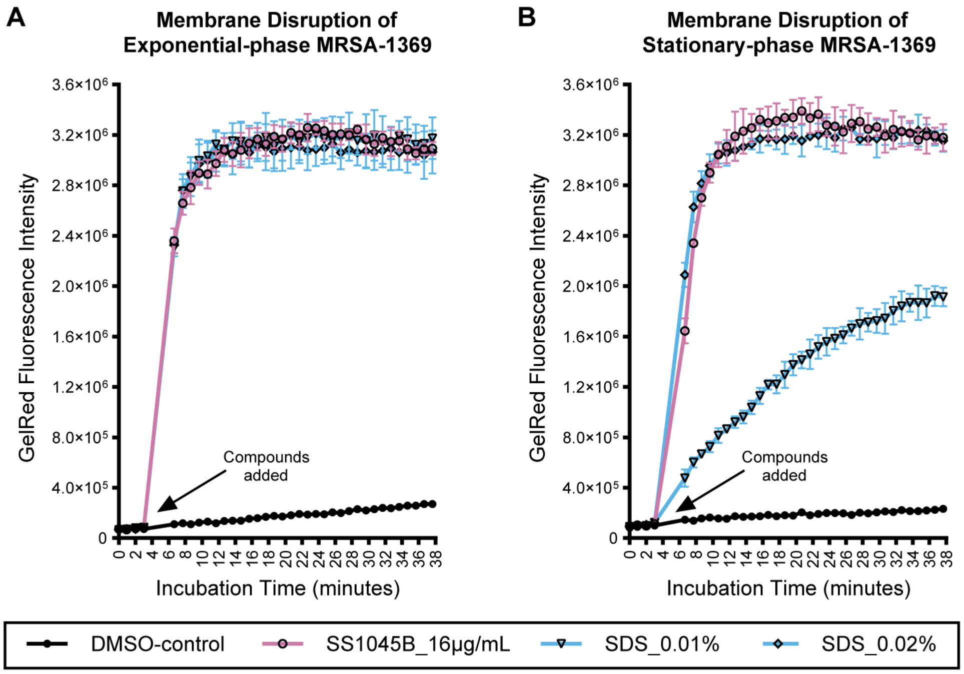

Fig. S4. SS1045B causes membrane disruption in exponential and stationary MRSA-1369. Membrane disruption in exponential **(A)** and stationary **(B)** MRSA-1369 cells was determined by monitoring the uptake of the membrane-impermeable fluorescent dye GelRed in presence of DMSO (vehicle control), 0.01-0.02% SDS or 16 µg/mL (28 µM) SS1045B for 30 minutes at 37°C in 1x PBS. The results are shown as means from triplicates and error bars indicate the standard deviation.

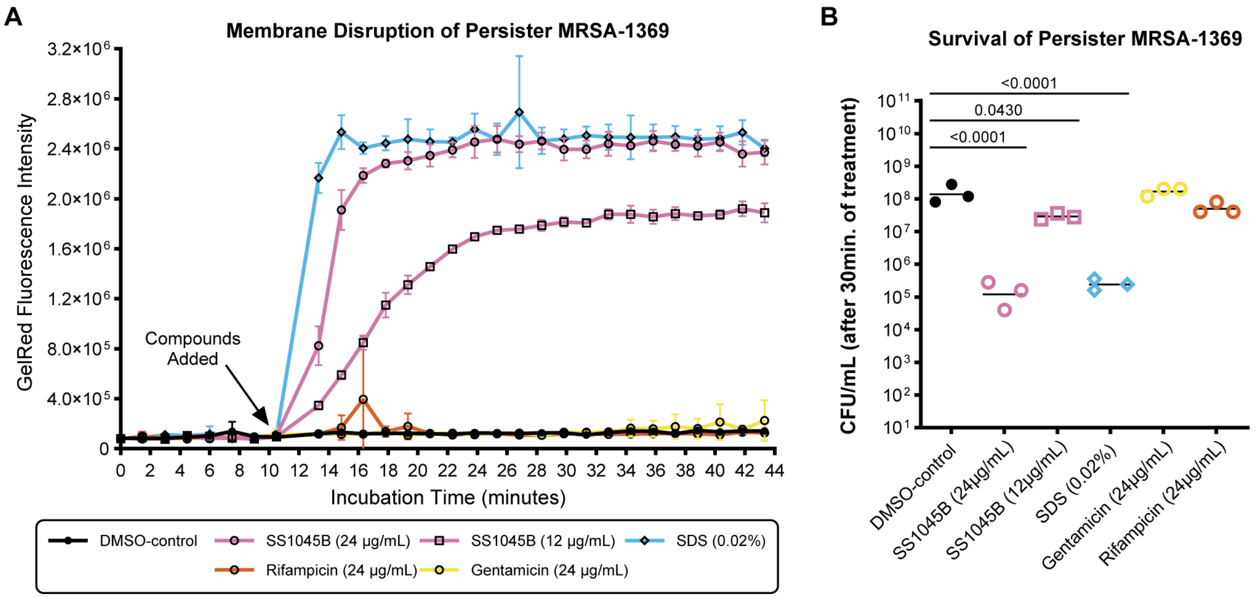

Fig. S5. SS1045B causes membrane disruption and kills gentamicin-persister MRSA-1369 cells. (A) Monitoring membrane integrity of gentamicin-persister MRSA-1369 cells. Membrane disruption was determined by monitoring the uptake of the membrane-impermeable fluorescent dye GelRed in presence of DMSO (vehicle control), 0.02% SDS, 12-24 µg/mL (21.3-42.5 µM; 4-8x MIC) SS1045B, 24 µg/mL rifampicin (MIC <0.006 µg/mL) or 24 µg/mL gentamicin (MIC <1.5 µg/mL) for 30 minutes at 37°C in 1x PBS. The results are shown as means from triplicates and error bars indicate the standard deviation. (**B**) Determination of CFUs at the end of 30-minute membrane integrity monitoring of gentamicin-persister MRSA-1369 cells from Fig. S7A. CFUs were determined by spot titer assay. Statistical analysis by ordinary one-way ANOVA with Tukey's comparison.

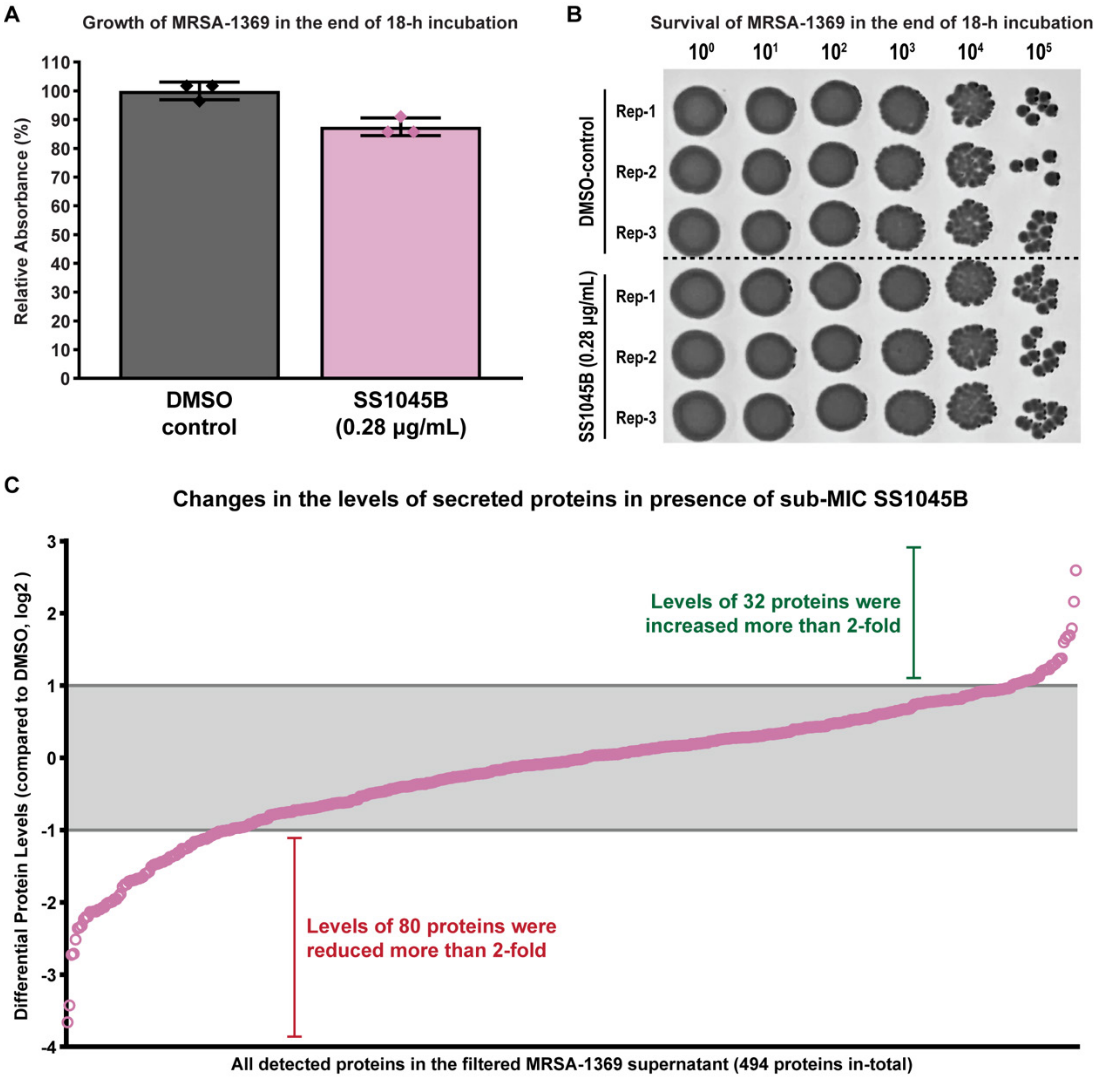

Fig. S6. Summary of mass spectrometry (secretomics) on the supernatants of MRSA-1369 cells treated with sublethal concentration of SS1045B. (A) Final OD_{600} values of MRSA-1369 at the end of 18-hour shaking incubation in chemically defined medium in presence of DMSO (vehicle-control) or SS1045B (0.28 µg/mL, 0.5 µM, sublethal). Bar graphs are represented as means from triplicates and error bars indicate the standard deviation. **(B)** Spot titer assay of MRSA-1369 cultures at the end of 18-hour shaking incubation in chemically defined medium in presence of DMSO (vehicle-control) or SS1045B (0.28 µg/mL, 0.5 µM, sublethal). **(C)** Differential levels of secreted proteins (494 in-total) were detected in the supernatants of MRSA-1369 at the end of 18-hour of shaking incubation in chemically defined medium in presence of DMSO (vehicle-control) or SS1045B (0.28 µg/mL, 0.5 µM, sublethal). Amongst all detected proteins, 80 were more than 2-fold downregulated and 32 were more than 2-fold upregulated upon treatment with 0.28 µg/mL (0.5 µM) SS1045B compared to DMSO (vehicle-control).

Table S1. Properties of new generation GmPcides. MIC/MBC in MRSA-1369, hemolytic activity at 100 µM concentration and cytotoxicity towards HeLa cells were determined as described in Materials and Methods.

**Name**	**Structure**	**MIC MRSA**	**MBC MRSA**	**Hemolysis (100 µM)**	**Cytotoxicity in HeLa cells (% viable cells) at**			
					**10 µM**	**20 µM**	**40 µM**	**80 µM**
**PS897**	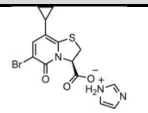	>25 µM	>25 µM	n.d.	111%	115%	110%	110%
**PS757**	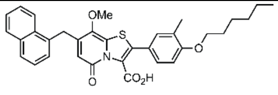	**5 µM**	**10 µM**	**0.57%**	**104%**	**94%**	**99%**	**7%**
**IL305**	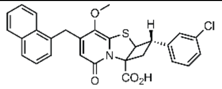	>25 µM	>25 µM	0.68%	91%	82%	65%	25%
**IL262**	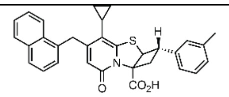	10 µM	10 µM	1.37%	91%	84%	67%	9%
**IL263**	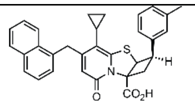	>25 µM	>25 µM	0.56%	99%	92%	96%	51%
**NQA8**	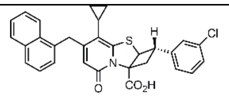	**5 µM**	**10 µM**	**0.77%**	**96%**	**86%**	**69%**	**10%**
**NQA9**	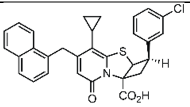	25 µM	25 µM	0.74%	98%	90%	90%	55%
**PS1961**	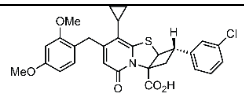	>25 µM	>25 µM	1.29%	96%	94%	73%	30%
**PS1962**	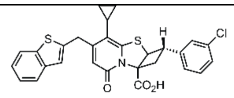	**5 µM**	**5 µM**	**0.61%**	**106%**	**107%**	**93%**	**47%**
**PS1963**	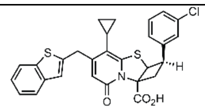	>25 µM	>25 µM	0.67%	102%	107%	104%	94%
**PS1965**	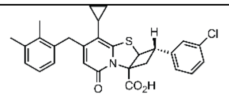	10 µM	10 µM	2.60%	105%	103%	83%	30%
**PS1970**	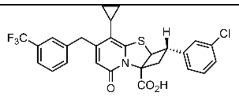	25 µM	>25 µM	2.78%	102%	105%	89%	34%

Table S2. Identification of MRSA-1369 mutants that are resistant to PS900 and their corresponding MIC for PS900 and PS1962. These mutants were obtained at the continuous exposure assay (see Fig. S1).

Strains	Mutations in coding regions	PS900 MIC (old generation)	PS1962 MIC (new generation)
**MRSA-1369 (wild type)**	none	5 µM<br>3.12 µg/ml	5 µM<br>2.60 µg/ml
**HT110**	rny (A427V)<br>comFA (D95G)<br>farR (V115I)	>80 µM<br>>49.90 µg/ml	10 µM<br>5.20 µg/ml
**HT114**	cspA (G14E)<br>ktrB (W264*)	20 µM<br>12.48 µg/ml	10 µM<br>5.20 µg/ml
**HT115**	ugtP (AATT ins)<br>farR (V115I)<br>farR (R96I)	>80 µM<br>>49.90 µg/ml	10 µM<br>5.20 µg/ml

Table S3. Properties of PS1962 analogues. MIC/MBC in MRSA-1369, hemolytic activity at 100 µM concentration, kinetic solubility and cytotoxicity (IC50) in HeLa and HepG2 cells were determined as described in Materials and Methods.

Name	Structure	MIC MRSA	MBC MRSA	Hemolysis (100 µM)	Kinetic Solubility	Cytotoxicity (IC50)	
						in HeLa cells	in HepG2 cells
**PS1962**	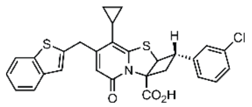	**5 µM**	**5 µM**	**0.61%**	**2.32 µM**	**75.5 µM**	**118.3 µM**
**SS1040B**	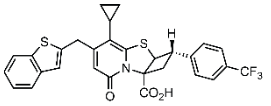	**5 µM**	**5 µM**	**0.73%**	**4.52 µM**	**54.2 µM**	**87.0 µM**
**SS1045B**	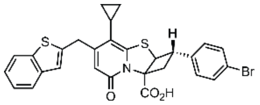	**5 µM**	**5 µM**	**0.64%**	**64.64 µM**	**61.9 µM**	**90.9 µM**
**SS1022B**	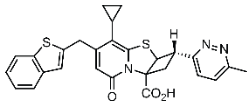	>25 µM	>25 µM	n.d.	n.d.	n.d.	n.d.
**PS2840**	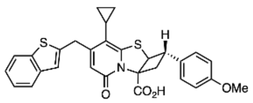	10 µM	25 µM	n.d.	n.d.	n.d.	n.d.
**SS1238B**	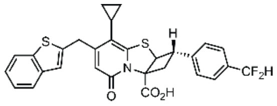	10 µM	25 µM	n.d.	n.d.	n.d.	n.d.
**SS998**	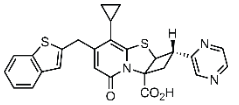	>25 µM	>25 µM	n.d.	n.d.	n.d.	n.d.
**SS991B**	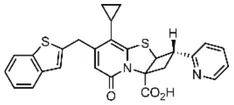	>25 µM	>25 µM	n.d.	n.d.	n.d.	n.d.

Table S4. TriPcides (PS1962 and SS1045B) are effective against daptomycin-resistant MRSA strains. MICs for daptomycin, PS1962 and SS1045 were determined by OD_{600} measurements at the end of 24-hour shaking incubation at 37°C in BHI-medium with or without 50 µg/mL $CaCl_2$ supplement in presence of varying concentrations of daptomycin (2-128 µg/mL), PS1962 (0.78 -50 µM) or SS1045B (0.78-50 µM). MICs are represented as means from triplicates.

**Strains**	**Daptomycin MIC (µg/mL)**		**PS1962 MIC (µg/mL)**		**SS1045B MIC (µg/mL)**	
	**- CaCl2**	**+ $CaCl_2$**	**- CaCl2**	**+ $CaCl_2$**	**- CaCl2**	**+ $CaCl_2$**
**MRSA-1369**	42.7	6.7	3.28	3.28	3.56	3.56
**Dapto-1**	128	16	3.28	3.28	2.94	3.56
**Dapto-2**	128	21.3	3.28	3.28	3.56	3.56
**Dapto-3**	64	16	3.28	3.28	3.56	3.56
**Dapto-4**	128	16	3.28	5.41	3.56	5.87
**Dapto-5**	85.3	16	3.28	5.41	3.56	4.69

Table S5. Checkerboard assay to determine front-line anti-MRSA antibiotic interactions with SS1045B. MICs for SS1045B-daptomycin checkerboard assay were determined by OD_{600} measurements at the end of 24-hour shaking incubation at 37°C in BHI-medium with 50 µg/mL $CaCl_2$ supplement in presence of varying concentrations of daptomycin (0.25-128 µg/mL) and/or SS1045B (0.39-25 µM). MICs for SS1045B-linezolid checkerboard assay were determined by OD_{600} measurements at the end of 24-hour shaking incubation at 37°C in BHI-medium in presence of varying concentrations of linezolid (0.024-25 µg/mL) and/or SS1045B (0.39-25 µM). MICs and FICs are represented as means from triplicates.

Strain	SS1045B MIC Standard	SS1045B MIC Combo	Antibiotic	Antibiotic MIC Standard	Antibiotic MIC Combo	SS1045B FIC	Antibiotic FIC	FIC Index	Interaction
MRSA-1369	3.1 µM	3.1 µM	daptomycin	10.7 µg/mL	10.7 µg/mL	1.00	1.00	2.00	indifferent
Newman	3.1 µM	2.1 µM	daptomycin	8 µg/mL	9.3 µg/mL	0.67	1.17	1.83	indifferent
MRSA-1369	3.1 µM	3.1 µM	linezolid	1.6 µg/mL	1.6 µg/mL	1.00	1.00	2.00	indifferent
Newman	3.1 µM	2.1 µM	linezolid	1.6 µg/mL	1.0 µg/mL	0.67	0.67	1.33	indifferent

Table S6. List of strains used in this study

Species	Strain	Description	Resistance	Reference (PMID)
Staphylococcus aureus	MRSA-1369	Urine isolate	Methicillin	(*31*)
Staphylococcus aureus	USA300 LAC	Clinical isolate		(*32*)
Staphylococcus aureus	Newman	Clinical isolate		(*33*)*
Staphylococcus aureus	Dapto-1	Clinical isolate	Daptomycin	(*34*)**
Staphylococcus aureus	Dapto-2	Clinical isolate	Daptomycin	(*34*)**
Staphylococcus aureus	Dapto-3	Clinical isolate	Daptomycin	(*34*)**
Staphylococcus aureus	Dapto-4	Clinical isolate	Daptomycin	(*34*)**
Staphylococcus aureus	Dapto-5	Clinical isolate	Daptomycin	(*34*)**
Enterococcus faecalis	OG1RF	Laboratory strain	Rif, Fusidic acid	(*35*)
Bacillus subtilis	168	Laboratory strain		(36)
Streptococcus pyogenes	HSC5	Laboratory strain		(*37*)
Streptococcus pneumoniae	JAM159	Clinical isolate		***

* Gift of Julianne Bubeck-Wardenburg, Washington University School of Medicine

** Gift of Melanie Yarbrough, Washington University School of Medicine

*** Gift of Celeste Morley, Washington University School of Medicine

Details on synthesis of the compounds and supporting analytic data

General remarks

Unless stated, all reagents and solvents were used as received from commercial suppliers. All reactions were carried out under an inert atmosphere with dry solvents under anhydrous conditions, unless otherwise indicated. Microwave reactions were performed in sealed vessels using a Biotage® Initiator microwave synthesizer; temperatures were monitored by an internal IR probe. TLC was performed on purchased aluminium backed silica gel plates (median pore size 60 Å, fluorescent indicator 254 nm) and detected with UV light at 254 and 366 nm. Flash column chromatography was performed using silica gel (0.063–0.200 mesh). Automated flash column chromatography was performed using a Biotage® Isolera One system and purchased pre-packed silica gel cartridges (Biotage® SNAP Cartridge, KP-Sil). Optical rotation was measured with a Perkin Elmer polarimeter 343 at 25 °C and 589 nm. ^{1}H-, ^{13}C- and ^{19}F-NMR spectra were recorded, depending on instrument availability, on a Bruker Avance III 400 MHz spectrometer with a BBO-F/H Smartprobe^{TM}, a Bruker Avance III HD 600 MHz spectrometer with a CP BBO-H/F, 5 mm cryoprobe, or a Bruker Avance III HD 850 MHz spectrometer with a CP TCI HCN, 5 mm cryoprobe, at 298 K, unless other temperature is given. All spectrometers were operated by Topspin 3.5.7. Resonances are given in ppm relative to TMS, and calibrated to solvent residual signals ($CDCl_3$: δ_H = 7.26 ppm; δ_C = 77.16 ppm. $(CD_3)_2SO$: δ_H = 2.50 ppm; δ_C = 39.51 ppm. CD_3OD δ_H = 3.31 ppm; δ_C = 49.00 ppm). The following abbreviations are used to indicate splitting patterns: s = singlet; d = doublet; dd = double doublet; t = triplet; m = multiplet; bs = broad singlet. LC-MS was conducted on a Micromass ZQ mass spectrometer with ES^{+} and ES^{-} ionization. HRMS was performed on a mass spectrometer with ESI-TOF (ES^{+}).

Reaction Set up for [2+2] photocycloaddition:

The 2+2 photocycloaddition reactions were conducted in borosilicate glass vials (WICOM 20mm Rollrandflasche, klar, 10 mL, 46.0mm(H) x 22.5mm (AD), fuer Agilent Headspace GC, Carlo-Erba Headspce, flacher boerdelrand, WIC 43110) sealed with microwave lids. The reaction mixture was irradiated at a distance of 2-4 cm from LED. as shown in **Fig. S7**. The 395 nm LEDs were bought from Mouser Electronics, Sweden (https://www.mouser.se/ProductDetail/ams-OSRAM/LZ4-40UB00-00U5?qs=VFUlUigmnHIR8XDToflSOw%3D%3D, accessed on 2025-06-09).

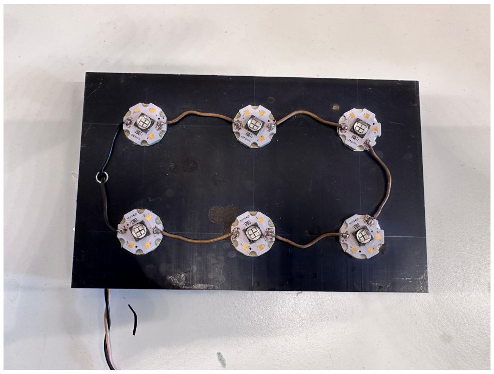

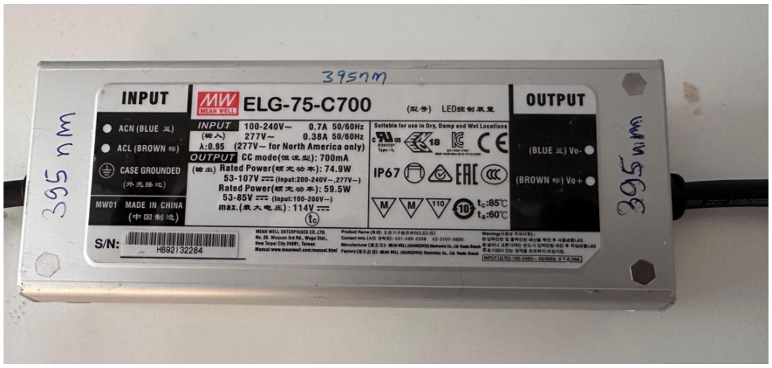

Fig. S7. 395 nm LED and power supply

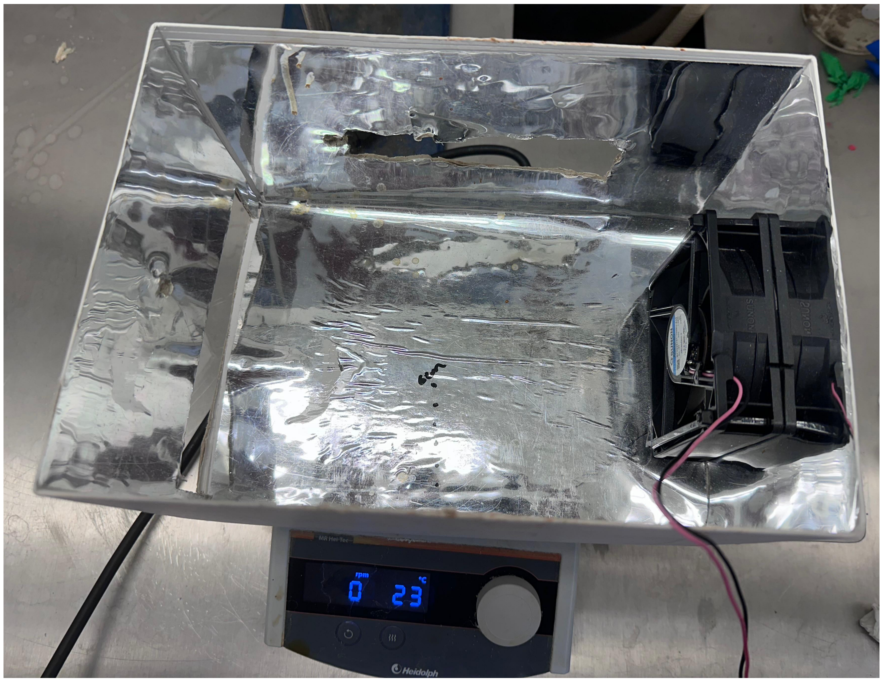

Fig. S8. In house made cooling box equipped with a fan

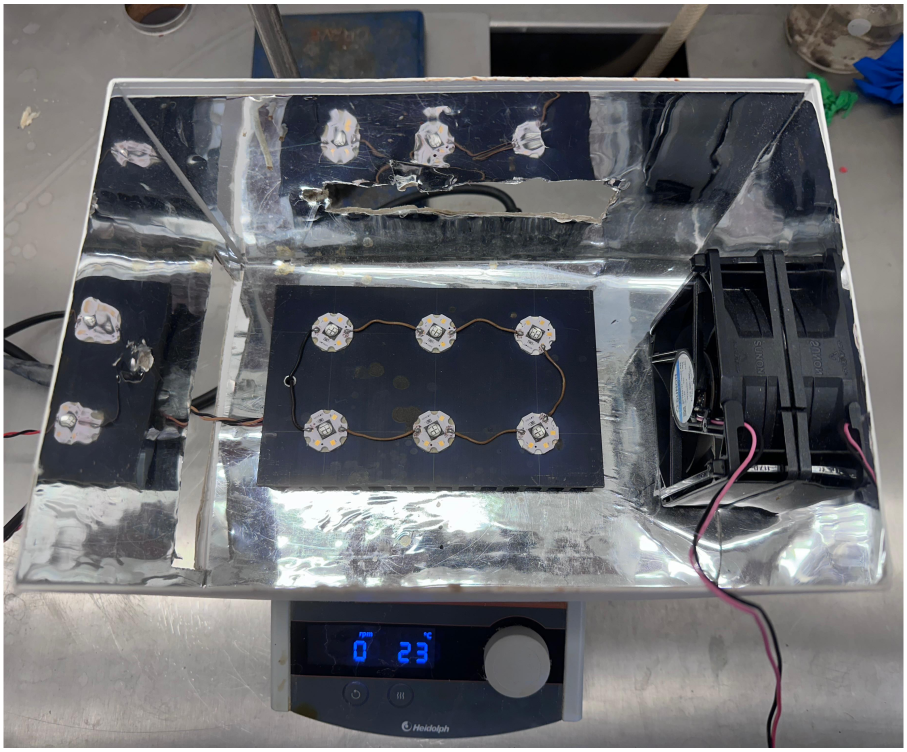

Fig. S9. Cooling box with 395 nm LED on a stirring plate

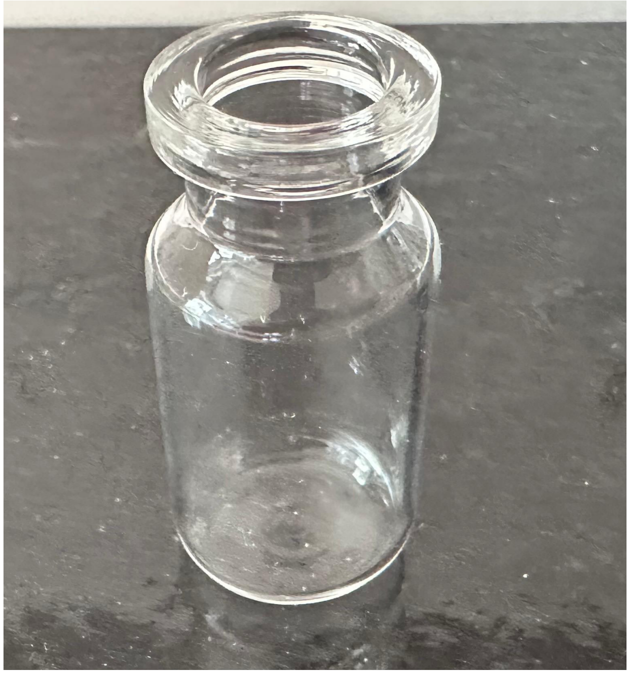

Fig. S10. Reaction vial

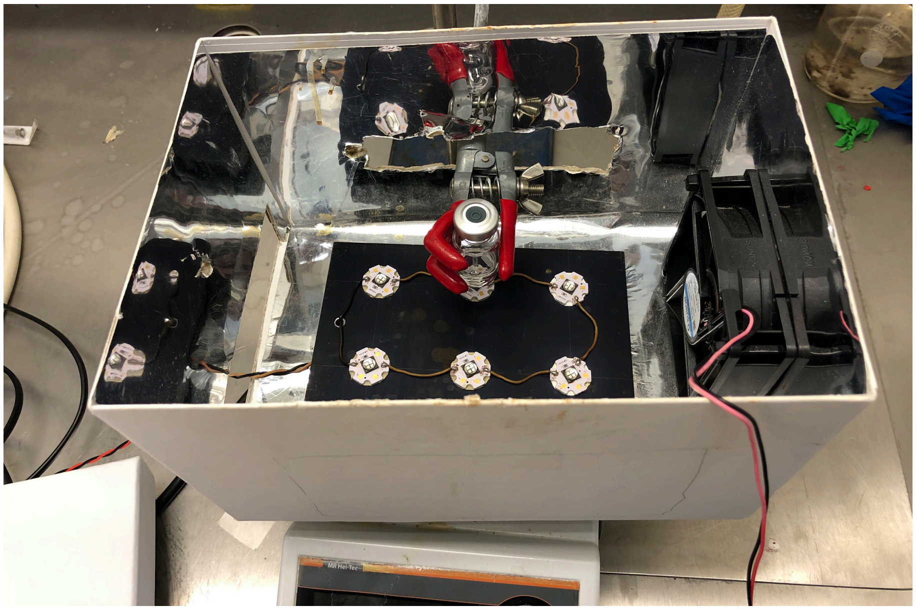

Fig. S11. Reaction vial placed over 395 nm LED

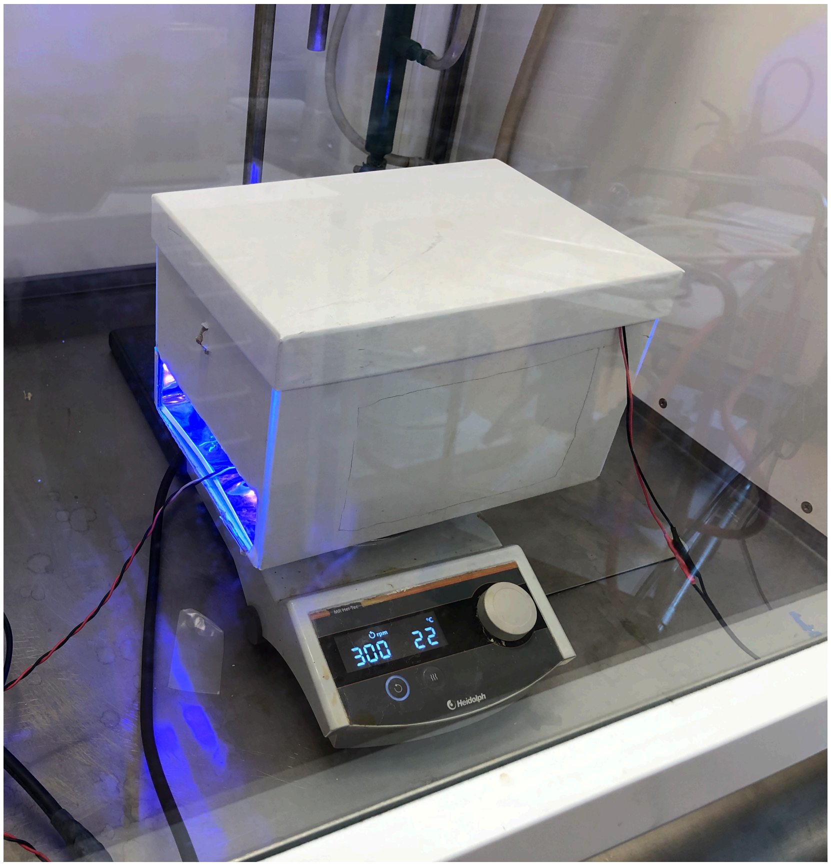

Fig. S12. Irradiation of reaction mixture with 395 nm LED under cooling with a fan.

Synthesis of intermediates

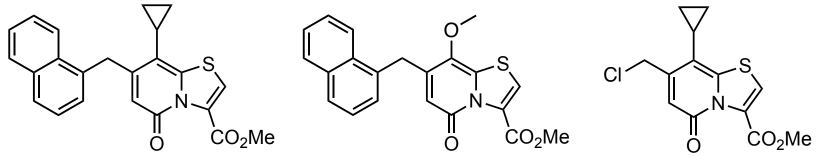

1a **1b** **1c**

Intermediates **1a**, **1b** and **1c** were prepared according to the reported procedures (*29*, *30*).

General procedure for the preparation of intermediates 1d-g

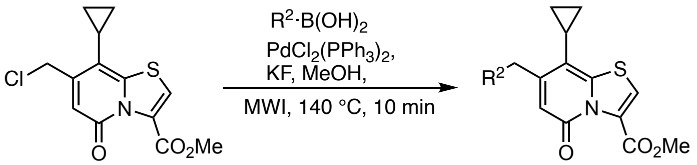

1c **1d-g**

Fig. S13. General procedure for the preparation of intermediates 1d-g

Microwave vial was charged with thiazolo-2-pyridone **1c** (0.40 mmol), added MeOH (3 mL) and degassed with nitrogen gas for 10 minutes. Added boronic acid (0.80 mmol), KF (0.60 mmol) and $PdCl_2(PPh_3)_2$ (0.04 mmol) The reaction mixture was again degassed with nitrogen gas for 5 minutes. Microwave vial was sealed and reaction mixture heated under MWI at 140 °C for 12 minutes. Diluted with dichloromethane (15 mL) and washed with brine (10 mL). Aqueous layer was extracted with DCM (15 mL). Organic layers were combined, dried over anhydrous Na_2SO_4 and evaporated to yield crude product which was purified by flash column chromatography (Biotage 100 g column) eluting with 0-100% ethylacetate in heptane.

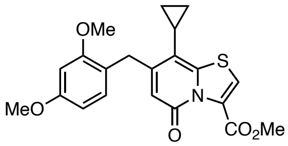

Methyl 8-cyclopropyl-7-(2,4-dimethoxybenzyl)-5-oxo-5*H*-thiazolo[3,2-*a*]pyridine-3-carboxylate (1d). Prepared by following the general procedure**,** purified by automated flash column chromatography (25 g SNAP Cartridge) eluting with 0–100% ethyl acetate in heptane. 120 mg of **1c** was converted to 138 mg (85%) of **1d**, isolated as a light yellow solid. IR ($CHCl_3$, cm^{-1}): ν 3002, 2951, 1740, 1655, 1507, 1470, 1434, 1255, 1209, 1038, 748. ^1H NMR (600 MHz, $CDCl_3$) δ 7.04 (s, 1H), 6.93 (d, *J* = 8.3 Hz, 1H), 6.45 (d, *J* = 2.4 Hz, 1H), 6.41 (dd, *J* = 8.3, 2.5 Hz, 1H), 6.03 (s, 1H), 4.01 (s, 2H), 3.94 (s, 3H), 3.79 (s, 3H), 3.75 (s, 3H), 1.75 – 1.70 (m, 1H), 1.04 – 1.01 (m, 2H), 0.73 – 0.70 (m, 2H). ^{13}C NMR (151 MHz, $CDCl_3$) δ 161.4, 160.0, 159.3, 158.3, 154.9, 146.8, 131.5, 130.9, 119.2, 113.7, 112.7, 111.3, 104.2, 98.7, 55.5, 55.4, 53.4, 32.7, 11.0, 7.9. HRMS (ESI) calcd $(M+H)^+$ $C_{21}H_{22}NO_5S^+$ 400,1213 observed 400.1195.

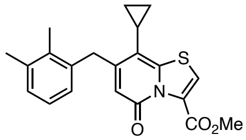

Methyl 8-cyclopropyl-7-(2,3-dimethylbenzyl)-5-oxo-5*H*-thiazolo[3,2-*a*]pyridine-3-carboxylate (1e). Prepared by following the general procedure**,** purified by automated flash column chromatography (25 g SNAP Cartridge) eluting with 0–100% ethyl acetate in heptane. 120 mg of **1c** was converted to 135 mg (91%) of **1e**, isolated as a light yellow solid. IR ($CHCl_3$, cm^{-1}): ν 3004, 1741, 1655, 1563, 1473, 1433, 1246, 1224, 1123, 1038, 750. ^1H NMR (600 MHz, $CDCl_3$) δ 7.07 (d, *J* = 8.2 Hz, 1H), 7.06 (s, 2H), 7.03 (t, *J* = 7.5 Hz, 1H), 6.89 (d, *J* = 6.3 Hz, 1H), 5.86 (s, 1H), 4.10 (s, 2H), 3.93 (s, 3H), 2.29 (s, 3H), 2.09 (s, 3H), 1.79 – 1.75 (m, 1H), 1.08 – 1.04 (m, 2H), 0.75 – 0.71 (m, 2H). ^{13}C NMR (151 MHz, $CDCl_3$) δ 161.3, 159.3, 154.4, 147.0, 137.3, 136.2, 135.1, 131.6, 128.9, 128.0, 125.8, 113.8, 112.4, 111.2, 53.4, 37.4, 20.7, 15.5, 10.9, 7.9. HRMS (ESI) calcd $(M+Na)^+$ $C_{21}H_{21}NNaO_3S^+$ 390,1134 observed 390.1153.

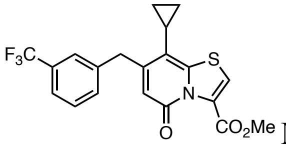

Methyl 8-cyclopropyl-5-oxo-7-(3-(trifluoromethyl)benzyl)-5*H*-thiazolo[3,2-*a*]pyridine-3-carboxylate (1f). Prepared by following the general procedure**,** purified by automated flash column chromatography (25 g SNAP Cartridge) eluting with 0–100% ethyl acetate in heptane. 120 mg of **1c** was converted to 130 mg (79%) of **1f**, isolated as a light yellow solid. IR ($CHCl_3$, cm^{-1}): ν 3089, 3005, 1741, 1657, 1564, 1471, 1434, 1248, 1229, 1124, 1039, 758. ^1H NMR (600 MHz, $CDCl_3$) δ 7.49 (d, *J* = 7.8 Hz, 1H), 7.46 (s, 1H), 7.41 (t, *J* = 7.7 Hz, 1H), 7.35 (d, *J* = 7.8 Hz, 1H), 7.09 (s, 1H), 6.15 (s, 1H), 4.16 (s, 2H), 3.96 (s, 3H), 1.57 – 1.52 (m, 1H), 1.05 – 1.01 (m, 2H), 0.71 – 0.67 (m, 2H). ^{13}C NMR (151 MHz, $CDCl_3$) δ 161.1, 159.0,

152.9, 148.0, 139.4, 132.4, 131.7, 131.2(q, *J* = 31.7 Hz) 129.2, 125.8 (q, *J* = 3.9 Hz), 125.0 (q, *J* = 273.3 Hz), 123.75 (q, *J* = 3.3 Hz), 114.2, 112.3, 112.1, 53.5, 39.1, 11.0, 8.2 (2C). ^{19}F NMR (565 MHz, $CDCl_3$) δ -62.54. HRMS (ESI) calcd $(M+Na)^+$ $C_{20}H_{16}F_3NNaO_3S^+$ 430,0695 observed 430.0718.

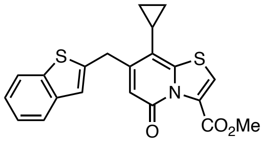

Methyl 7-(benzo[*b*]thiophen-2-ylmethyl)-8-cyclopropyl-5-oxo-5*H*-thiazolo[3,2-*a*]pyridine-3-carboxylate (1g). Prepared by following the general procedure**,** purified by automated flash column chromatography (50 g SNAP Cartridge) eluting with 0–100% ethyl acetate in heptane. 520 mg of **1c** was converted to 522 mg (75%) of **1g**, isolated as a light yellow solid. IR ($CHCl_3$, cm^{-1}): ν 3583, 1738, 1655, 1563, 1469, 1433, 1247, 1229, 1125, 1038, 748. ^{1}H NMR (400 MHz, $CDCl_3$) δ 7.74 (dd, *J* = 7.9, 1.2 Hz, 1H), 7.66 (dd, *J* = 7.2, 1.7 Hz, 1H), 7.34 – 7.24 (m, 2H), 7.09 (s, 1H), 7.01 (s, 1H), 6.36 (s, 1H), 4.34 (s, 2H), 3.97 (s, 3H), 1.74 – 1.67 (m, 1H), 1.13 – 0.98 (m, 2H), 0.77 – 0.61 (m, 2H). ^{13}C NMR (100 MHz, $CDCl_3$) δ 161.1, 159.1, 152.2, 148.0, 142.0, 139.9, 139.7, 131.7, 124.4, 124.0, 123.2, 122.5, 122.2, 114.2, 112.3, 111.8, 53.5, 34.4, 10.9, 8.0. HRMS (ESI) calcd $(M+Na)^+$ $C_{21}H_{17}NNaO_3S_2^+$ 418,0542 observed 418.0536.

Design of TriPcides

The previously reported bicyclic GmPcides have a high degree of sp^2 bonds (unsaturated double bonds) resulting in a fairly flat scaffold. It's well documented that molecules with a flat appearance more easily aggregate, resulting in lower solubility. In addition, the more active bicyclic GmPcides were substituted with a hydrophobic carbon chain, resulting in a fatty acid-like molecule. This in turn led to the target of efflux-mediated resistance across multiple bacterial genera. Thus, to enhance pharmaco kinetic (PK) properties and reduce efflux susceptibility, a new sp^3-rich carbon scaffold was designed by incorporating a substituted cyclobutyl ring, forming a novel tricyclic scaffold (Fig. S14). This new scaffold modification would keep the essential structural elements in their position while simultaneously increase the number of sp^3 carbons and thus result in a more three dimensional and complex molecule.

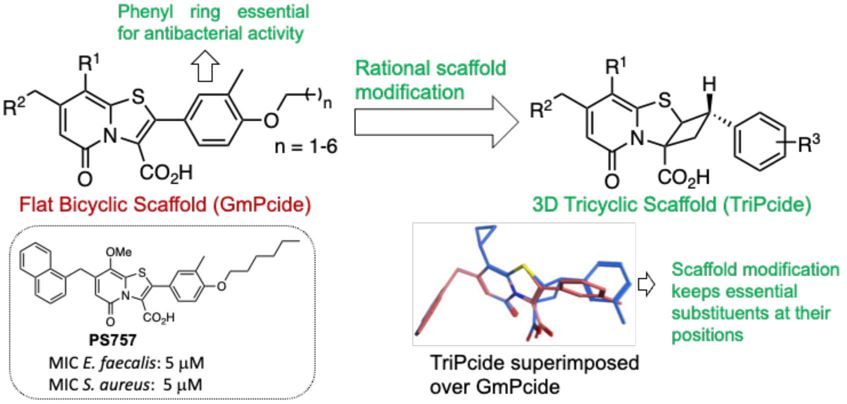

Fig. S14. PS757, a flat bicyclic GmPcide; and rationally designed novel 3D TriPcide scaffold to overcome the problem of resistance.

To create this new scaffold, we developed a light catalyzed, regio- and stereoselective [2+2] cycloaddition. The synthetic methodology to prepare new tricyclic analogues is very robust, no expensive photocatalyst is needed, has a wide substrate scope and this transformation is scalable and can be performed at a late stage in the synthetic sequence.

General Procedure for the synthesis of 2a-n and 3a-n

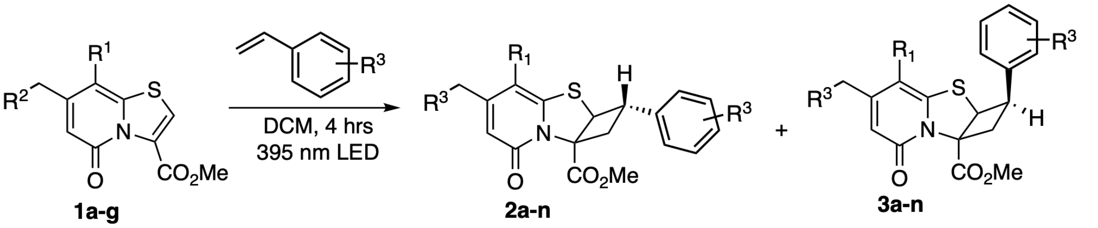

Fig. S15. General Procedure for the synthesis of 2a-n and 3a-n

Thiazolo-2-Pyridone of general structure **1** (0.25 mmol) was charged into a glass vial and added 2.5 mL of DCM, followed by the addition of styrene (4 eq). The reaction mixture was irradiated under purple LED at 395 nm after degassing with nitrogen for 3-5 minutes. After completion of reaction as indicated by TLC, the solvent was removed under reduced pressure and the crude product was purified by column chromatography using Biotage isolera (25 g cartridge, heptane/EtOAc 0-100%)

Synthesis of compound 2a and 3a

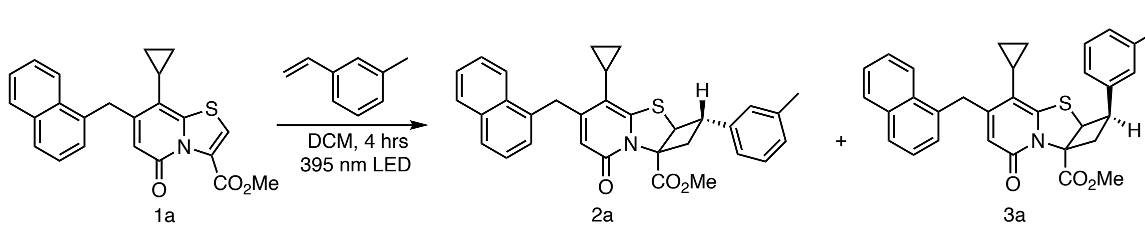

Fig. S16. Synthesis of compound 2a and 3a

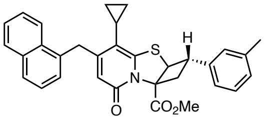

Methyl (2*S*)-4-cyclopropyl-5-(naphthalen-1-ylmethyl)-7-oxo-2-(*m*-tolyl)-2,2a-dihydro-7*H*-cyclobuta[4,5]thiazolo[3,2-*a*]pyridine-8a(1*H*)-carboxylate (2a). Prepared by following the general procedure, purified by automated flash column chromatography (25 g SNAP Cartridge) eluting with 0–100% ethyl acetate in heptane. 100 mg of **1** was converted to 96 mg (73%) of **2a**, isolated as a white solid. IR ($CHCl_3$, cm^{-1}): ν 3007, 1743, 1652, 1577, 1489, 1432, 1304, 1218, 1116, 1032, 753. ^{1}H NMR (600 MHz, $CDCl_3$) δ 8.02 – 7.96 (m, 2H), 7.92 (d, J = 8.2 Hz, 1H), 7.63 – 7.61 (m, 2H), 7.56 (t, J = 7.6 Hz, 1H), 7.45 – 7.37 (m, 3H), 7.28 – 7.22 (m, 3H), 5.88 (s, 1H), 4.71 – 4.49 (q, J = 17.1 Hz, 2H), 4.31 (d, J = 7.6 Hz, 1H), 3.90 (s, 3H), 3.82 (dd, J = 9.8, 7.6 Hz, 1H), 3.56 (dd, J = 13.3, 9.9 Hz, 1H), 3.09 (dd, J = 13.3, 9.5 Hz, 1H), 2.50 (s, 3H), 1.87 – 1.83 (m, 1H), 1.16 – 1.08 (m, 2H), 0.99 – 0.92 (m, 2H). ^{13}C NMR (151 MHz, $CDCl_3$) δ 168.6, 161.1, 157.4, 149.5, 140.7, 138.7, 134.1, 134.0, 132.1, 129.0, 128.9, 128.3, 127.8, 127.5, 126.4, 125.7, 123.9, 123.8, 115.9, 114.0, 73.8, 53.3, 52.6, 48.0, 36.4, 35.3, 21.5, 11.4, 8.1, 8.0. HRMS (ESI) calcd $(M+H)^+$ $C_{32}H_{30}NO_3S^+$ 508,1941 observed 508.1923.

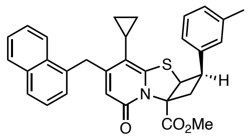

Methyl (2*R*)-4-cyclopropyl-5-(naphthalen-1-ylmethyl)-7-oxo-2-(*m*-tolyl)-2,2a-dihydro-7*H*-cyclobuta[4,5]thiazolo[3,2-*a*]pyridine-8a(1*H*)-carboxylate (3a). Prepared by following the general procedure, purified by automated flash column chromatography (25 g SNAP Cartridge) eluting with 0–100% ethyl acetate in heptane. 100 mg of **1** was converted to 23 mg (17%) of **3a**, isolated as a white solid. IR ($CHCl_3$, cm^{-1}): ν 3005, 1744, 1653, 1578, 1488, 1432, 1303, 1223, 1105, 1031, 783, 734. 1H NMR (600 MHz, CDCl3) δ 7.92 – 7.85 (m, 1H), 7.79 (t, *J* = 7.8 Hz, 2H), 7.51 – 7.47 (m, 2H), 7.45 – 7.41 (m, 1H), 7.26 (d, *J* = 5.4 Hz, 2H), 7.20 (t, *J* = 7.6 Hz, 1H), 7.09 (d, *J* = 7.5 Hz, 1H), 6.80 (d, *J* = 7.6 Hz, 1H), 6.77 (s, 1H), 5.76 (s, 1H), 4.69 (d, *J* = 8.4 Hz, 1H), 4.43 (q, *J* = 16.4 Hz, 2H), 4.09 (dd, *J* = 13.8, 6.4 Hz, 1H), 3.80 (s, 3H), 3.74 – 3.67 (m, 1H), 2.89 (dd, *J* = 13.8, 6.4 Hz, 1H), 2.32 (s, 3H), 1.46 – 1.41 (m, 1H), 0.88 – 0.81 (m, 1H), 0.74 – 0.79 (m, 1H), 0.54 – 0.50 (m, 1H), 0.31 – 0.26 (m, 1H). ^{13}C NMR (151 MHz, $CDCl_3$) δ 169.0, 160.5, 157.4, 150.2, 138.0, 137.9, 134.1, 134.1, 132.1, 129.0, 128.8, 128.2, 128.0, 127.8, 127.6, 126.3, 125.8, 125.6, 125.4, 123.8, 115.6, 113.1, 74.3, 53.4, 50.6, 41.6, 36.2, 33.3, 21.6, 10.9, 8.8, 7.4. HRMS (ESI) calcd $(M+H)^+$ $C_{32}H_{30}NO_3S^+$ 508,1941 observed 508.1935.

Synthesis of compound 2b and 3b

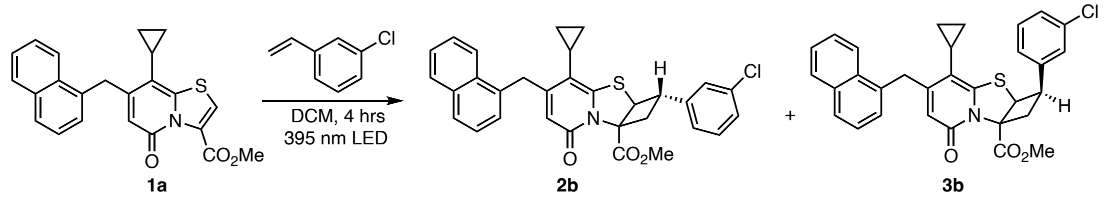

Fig. S17. Synthesis of compound 2b and 3b

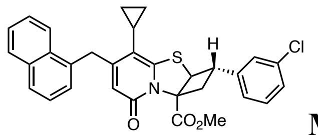

Methyl (2*S*)-2-(3-chlorophenyl)-4-cyclopropyl-5-(naphthalen-1-ylmethyl)-7-oxo-2,2a-dihydro-7*H*-cyclobuta[4,5]thiazolo[3,2-*a*]pyridine-8a(1*H*)-carboxylate (2b). Prepared by following the general procedure, purified by automated flash column chromatography (25 g SNAP Cartridge) eluting with 0–100% ethyl acetate in heptane. 100 mg of **1** was converted to 98 mg (72%) of **2b**, isolated as a white solid. IR ($CHCl_3$, cm^{-1}): ν 3006, 1743, 1652, 1575, 1490, 1434, 1330, 1226, 1117, 1033, 754. ^1H NMR (600 MHz, $CDCl_3$) δ 7.90 – 7.87 (m, 1H), 7.86 – 7.82 (m, 1H), 7.80 (d, *J* = 8.3 Hz, 1H), 7.51 – 7.48 (m, 2H), 7.44 (dd, *J* = 8.3, 7.0 Hz, 1H), 7.33 – 7.27 (m, 4H), 7.23 (d, *J* = 7.5 Hz, 1H), 5.75 (s, 1H), 4.50 (q, *J* = 17.3 Hz, 2H), 4.17 (d, *J* = 7.3 Hz, 1H), 3.78 (s, 3H), 3.74 – 3.69 (dd, *J* = 9.2, 7.4 Hz,1H), 3.42 (dd, *J* = 13.2, 10.0 Hz, 1H), 2.99 (dd, *J* = 13.3, 9.6 Hz, 1H), 1.75 – 1.71 (m, 1H), 1.05 – 0.96 (m, 2H), 0.87 – 0.80 (m, 2H). ^{13}C NMR (151 MHz, $CDCl_3$) δ 168.4, 161.0, 157.5, 149.1, 142.8, 134.8, 134.1, 134.0, 132.1, 130.3, 129.0, 127.9, 127.8 (2C), 127.0, 126.4, 125.9, 125.7, 124.9, 123.9, 116.0, 114.1, 73.7, 53.4, 52.4, 47.6, 36.4, 35.1, 11.4, 8.1, 8.0. HRMS (ESI-TOF) m/z: $[M+H]^+$ Calcd for $C_{31}H_{27}ClNO_3S^+$ 528,1395; observed 528.1368.

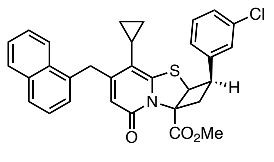

Methyl (2*R*)-2-(3-chlorophenyl)-4-cyclopropyl-5-(naphthalen-1-ylmethyl)-7-oxo-2,2a-dihydro-7*H*-cyclobuta[4,5]thiazolo[3,2-*a*]pyridine-8a(1*H*)-carboxylate (3b). Prepared by following the general procedure, purified by automated flash column chromatography (25 g SNAP Cartridge) eluting with 0–100% ethyl acetate in heptane. 100 mg of **1** was converted to 21 mg (15%) of **3b**, isolated as a white solid. IR ($CHCl_3$, cm^{-1}): ν 3005, 1743, 1650, 1573, 1488, 1437, 1304, 1221, 1102, 1032, 753.^{1}H NMR (400 MHz, $CDCl_3$) δ 7.90 – 7.86 (m, 1H), 7.83 – 7.78 (m, 2H), 7.51 – 7.46 (m, 2H), 7.43 (dd, *J* = 8.2, 7.0 Hz, 1H), 7.29 – 7.26 (m, 2H), 7.26 – 7.22 (m, 1H), 6.92 – 6.89 (m, 2H), 5.74 (s, 1H), 4.69 (dt, *J* = 8.5, 1.3 Hz, 1H), 4.45 (q, *J* = 17.4 Hz, 2H), 4.12 – 4.05 (m, 1H), 3.79 (s, 3H), 3.75 – 3.68 (m, 1H), 2.84 (ddd, *J* = 14.0, 6.1, 1.2 Hz, 1H), 1.49 – 1.42 (m, 1H), 0.90 – 0.83 (m, 1H), 0.76 – 0.79 (m, 1H), 0.62 – 0.56 (m, 1H), 0.31 – 0.25 (m, 1H). ^{13}C NMR (100 MHz, $CDCl_3$) δ 168.8, 160.5, 157.7, 149.4, 140.3, 134.4, 134.1, 134.0, 132.1, 129.6, 128.9, 128.1, 127.8 (2C), 127.5, 127.00, 126.3, 125.8, 125.6, 123.9, 115.8, 113.3, 74.3, 53.4, 50.3, 41.7, 36.3, 33.1, 10.9, 8.8, 7.3. HRMS (ESI-TOF) m/z: $[M+H]^+$ Calcd for $C_{31}H_{27}ClNO_3S^+$ 528,1395; observed 528.1365.

Synthesis of compound 2c and 3c

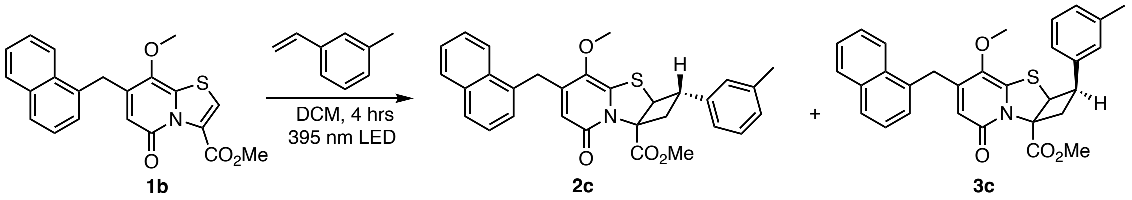

Fig. S18. Synthesis of compound 2c and 3c

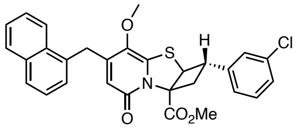

Methyl (2*S*)-2-(3-chlorophenyl)-4-methoxy-5-(naphthalen-1-ylmethyl)-7-oxo-2,2a-dihydro-7*H*-cyclobuta[4,5]thiazolo[3,2-*a*]pyridine-8a(1*H*)-carboxylate (2c). Prepared by following the general procedure, purified by automated flash column chromatography (25 g SNAP Cartridge) eluting with 0–100% ethyl acetate in heptane. 96 mg of **2** was converted to 28 mg (21%) of **2c**, isolated as a white solid. IR ($CHCl_3$, cm^{-1}): ν 3005, 2953, 1743, 1658, 1578, 1496, 1436, 1339, 1226, 1116, 1042, 780 735. ^{1}H NMR (400 MHz, $CDCl_3$) δ 7.92 – 7.87 (m, 2H), 7.81 (d, *J* = 8.2 Hz, 1H), 7.52 – 7.48 (m, 2H), 7.47 – 7.43 (m, 1H), 7.38 (d, *J* = 5.7 Hz, 1H), 7.34 – 7.28 (m, 3H), 7.23 – 7.20 (m, 1H), 5.75 (s, 1H), 4.38 (q, 16.7 Hz, 2H), 4.26 (d, *J* = 7.7 Hz, 1H), 3.81 (s, 3H), 3.79 (s, 3H), 3.77 – 3.71 (m, 1H), 3.40 (dd, *J* = 13.2, 10.0 Hz, 1H), 3.00 (dd, *J* = 13.3, 9.6 Hz, 1H). ^{13}C NMR (100 MHz, $CDCl_3$) δ 168.1, 160.0, 152.3, 142.4, 140.7, 137.2, 134.9, 134.1, 133.3, 132.0, 130.3, 129.0, 128.1, 127.9, 127.0, 126.5, 125.9, 125.6, 124.9, 123.9, 115.9, 74.3, 61.1, 53.5, 53.4, 47.6, 34.8, 32.9. HRMS (ESI-TOF) m/z: $[M+H]^+$ Calcd for $C_{29}H_{25}ClNO_4S^+$ 518,1187; observed 518.1160.

Synthesis of compound 2d and 3d

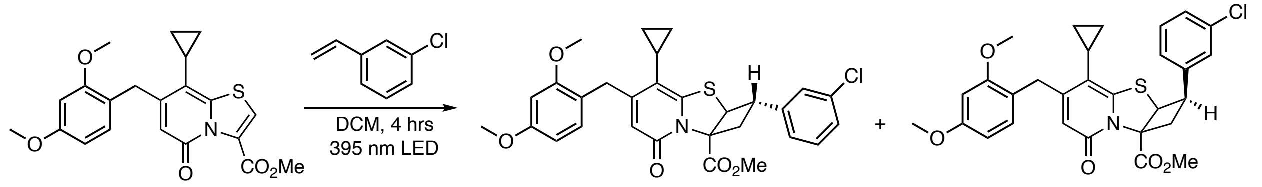

1d 2d (Crude: 4:1 dr) 3d

Fig. S19. Synthesis of compound 2d and 3d

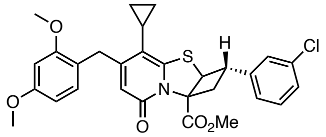

Methyl (2*S*)-2-(3-chlorophenyl)-4-cyclopropyl-5-(2,4-dimethoxybenzyl)-7-oxo-2,2a-dihydro-7*H*-cyclobuta[4,5]thiazolo[3,2-*a*]pyridine-8a(1*H*)-carboxylate (2d). Prepared by following the general procedure, purified by automated flash column chromatography (25 g SNAP Cartridge) eluting with 0–100% ethyl acetate in heptane. 100 mg of **1d** was converted to 70 mg (52%) of **3d**, isolated as a white solid. IR ($CHCl_3$, cm^{-1}): ν 3004, 2955, 2245, 1743, 1652, 1575, 1490, 1432, 1332, 1209, 1120, 1036, 730. ^1H NMR (600 MHz, $CDCl_3$) δ 7.32 – 7.29 (m, 2H), 7.28 – 7.25 (m, 2H), 7.23 – 7.21 (m, 1H), 6.99 (d, *J* = 8.2 Hz, 1H), 6.48 (d, *J* = 2.4 Hz, 1H), 6.45 (dd, *J* = 8.2, 2.4 Hz, 1H), 5.87 (s, 1H), 4.14 (d, *J* = 7.3 Hz, 1H), 3.96 (q, *J* = 16.7 Hz, 2H), 3.81 (s, 3H), 3.79 (s, 3H), 3.78 (s, 3H), 3.68 (td, *J* = 9.8, 7.3 Hz, 1H), 3.42 (dd, *J* = 13.1, 10.0 Hz, 1H), 3.02 – 2.97 (m, 1H), 1.65 – 1.61 (m, 1H), 1.01 – 0.90 (m, 2H), 0.79 – 0.72 (m, 2H). ^{13}C NMR (151 MHz, $CDCl_3$) δ 168.6, 161.2, 160.1, 158.4, 148.5, 142.9, 134.8, 131.2, 130.3, 127.7, 127.0, 124.9, 118.8, 115.3, 114.5, 104.3, 98.8, 73.6, 55.5, 55.5, 53.4, 52.4, 47.6, 35.1, 32.7, 11.4, 8.0, 8.01. HRMS (ESI-TOF) m/z: $[M+H]^+$ Calcd for $C_{29}H_{29}ClNO_5S^+$ 538,1449; observed 538.1431.

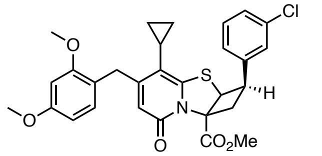

Methyl (2*R*)-2-(3-chlorophenyl)-4-cyclopropyl-5-(2,4-dimethoxybenzyl)-7-oxo-2,2a-dihydro-7*H*-cyclobuta[4,5]thiazolo[3,2-*a*]pyridine-8a(1*H*)-carboxylate (3d). Prepared by following the general procedure, purified by automated flash column chromatography (25 g SNAP Cartridge) eluting with 0–100% ethyl acetate in heptane. 100 mg of **1d** was converted to 12 mg (9%) of **3d**, isolated as a white solid. IR ($CHCl_3$, cm^{-1}): ν 3002, 2954, 1743, 1652, 1576, 1488, 1424, 1333, 1209, 1104, 1037, 730. 1H NMR (600 MHz, CDCl3) δ 7.24 – 7.19 (m, 2H), 6.95 (d, *J* = 8.3 Hz, 1H), 6.91 – 6.87 (m, 2H), 6.47 (d, *J* = 2.5 Hz, 1H), 6.44 (dd, *J* = 8.3, 2.5 Hz, 1H), 5.87 (s, 1H), 4.67 (d, *J* = 8.5 Hz, 1H), 4.08 (td, *J* = 9.6, 6.3 Hz, 1H), 3.90 (q, *J* = 16.6 Hz, 2H), 3.81 (s, 3H), 3.81 (s, 3H), 3.77 (s, 3H), 3.75 – 3.70 (m, 1H), 2.84 (dd, *J* = 13.9, 6.3 Hz, 1H), 1.38 – 1.33 (m, 1H), 0.85 – 0.80 (m, 1H), 0.69 – 0.66 (m, 1H), 0.54 – 0.50 (m, 1H), 0.24 – 0.22 (m, 1H). ^{13}C NMR (151 MHz, $CDCl_3$) δ 168.9, 160.7, 160.0, 158.6, 158.4, 148.9, 140.4, 134.4, 131.1, 129.5, 128.2, 127.4, 126.8, 118.8, 115.1, 113.7, 104.3, 98.7, 74.1, 55.5, 55.4, 53.4, 50.3, 41.6, 33.4, 32.6, 10.9, 8.7, 7.3. HRMS (ESI-TOF) m/z: $[M+H]^+$ Calcd for $C_{29}H_{29}ClNO_5S^+$ 538,1449; observed 538.1397.

Synthesis of compound 2e and 3e

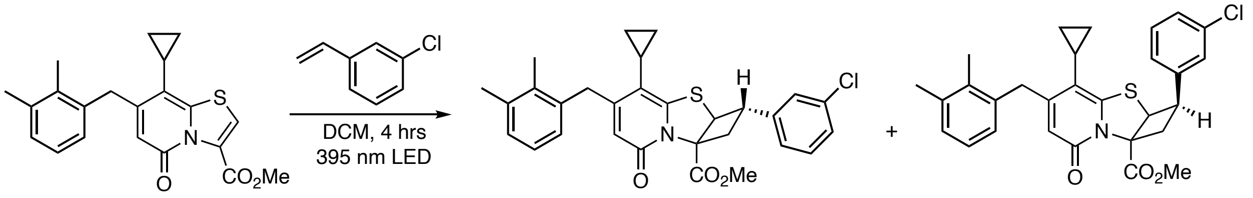

Fig. S20. Synthesis of compound 2e and 3e

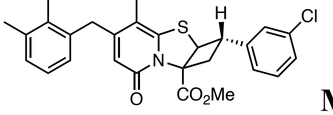

Methyl (2*S*)-2-(3-chlorophenyl)-4-cyclopropyl-5-(2,3-dimethylbenzyl)-7-oxo-2,2a-dihydro-7*H*-cyclobuta[4,5]thiazolo[3,2-*a*]pyridine-8a(1*H*)-carboxylate (2e). Prepared by following the general procedure, purified by automated flash column chromatography (25 g SNAP Cartridge) eluting with 0–100% ethyl acetate in heptane. 120 mg of **1e** was converted to 103 mg (62%) of **2e**, isolated as a white solid. IR ($CHCl_3$, cm^{-1}): ν 3583, 3384, 3005, 1743, 1652, 1575, 1490, 1433, 1306, 1226, 1117, 1032, 732. 1H NMR (600 MHz, CDC13) δ 7.33 – 7.30 (m, 2H), 7.29 – 7.26 (m, 2H), 7.24 – 7.22 (m, 1H), 7.09 – 7.05 (m, 2H), 6.95 (d, *J* = 7.2 Hz, 1H), 5.71 (s, 1H), 4.16 (d, *J* = 7.3 Hz, 1H), 4.03 (q, *J* = 18.6 Hz, 2H), 3.79 (s, 3H), 3.69 (td, *J* = 9.8, 7.4 Hz, 1H), 3.43 (dd, *J* = 13.2, 10.0 Hz, 1H), 2.99 (ddd, *J* = 13.3, 9.6, 1.1 Hz, 1H), 2.31 (s, 3H), 2.12 (s, 3H), 1.70 – 1.66 (m, 1H), 1.05 – 0.96 (m, 2H), 0.80 – 0.75 (m, 2H).^{13}C NMR (151 MHz, $CDCl_3$) δ 168.5, 161.2, 157.8, 148.8, 142.8, 137.4, 136.0, 135.3, 134.8, 130.3, 129.0, 128.3, 127.8, 127.0, 125.8, 124.9, 115.3, 114.1, 73.6, 53.4, 52.4, 47.6, 37.5, 35.1, 20.8, 15.6, 11.3, 8.1, 7.9. HRMS (ESI-TOF) m/z: $[M+H]^+$ Calcd for $C_{29}H_{29}ClNO_3S^+$ 506,1551; observed 506.1526. (Done)

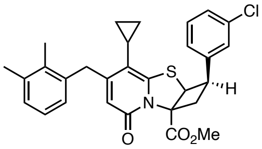

Methyl (2*R*)-2-(3-chlorophenyl)-4-cyclopropyl-5-(2,3-dimethylbenzyl)-7-oxo-2,2a-dihydro-7*H*-cyclobuta[4,5]thiazolo[3,2-*a*]pyridine-8a(1*H*)-carboxylate (3e). Prepared by following the general procedure, purified by automated flash column chromatography (25 g SNAP Cartridge) eluting with 0–100% ethyl acetate in heptane. 120 mg of **1e** was converted to 17 mg (10%) of **3e**, isolated as a white solid. IR ($CHCl_3$, cm^{-1}): ν 3583, 3384, 3004, 1743, 1650, 1573, 1489, 1434, 1305, 1219, 1103, 1031, 752. 1H NMR (600 MHz, CDC13) δ 7.26 – 7.20 (m, 2H), 7.09 – 7.04 (m, 2H), 6.92 (dd, *J* = 7.1, 2.0 Hz, 1H), 6.90 – 6.88 (m, 2H), 5.70 (s, 1H), 4.68 (d, *J* = 8.5 Hz, 1H), 4.08 (td, *J* = 9.6, 6.1 Hz, 1H), 4.01 (q, *J* = 18.0 Hz, 2H), 3.81 (s, 3H), 3.73 (ddd, *J* = 14.0, 9.9, 1.6 Hz, 1H), 2.85 (ddd, *J* = 14.0, 6.0, 1.1 Hz, 1H), 2.30 (s, 3H), 2.10 (s, 3H), 1.41 – 1.39 (m, 1H), 0.89 – 0.84 (m, 1H), 0.75 – 0.70 (m, 1H), 0.55 – 0.50 (m, 1H), 0.25 – 0.21 (m, 1H).^{13}C NMR (151 MHz, $CDCl_3$) δ 168.9, 160.7, 158.0, 149.1, 140.3, 137.3, 136.0, 135.3, 134.4, 129.5, 128.9, 128.3, 128.1, 127.5, 126.9, 125.8, 115.0, 113.3, 74.3, 53.4, 50.2, 41.7, 37.5, 33.1, 20.8, 15.6, 10.8, 8.8, 7.3 . HRMS (ESI-TOF) m/z: $[M+H]^+$ Calcd for $C_{29}H_{29}ClNO_3S^+$ 506,1551; observed 506.1489.

Synthesis of compound 2f and 3f

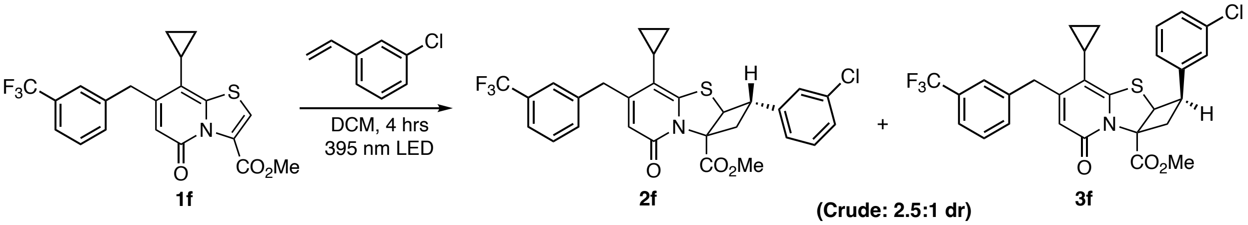

Fig. S21. Synthesis of compound 2f and 3f

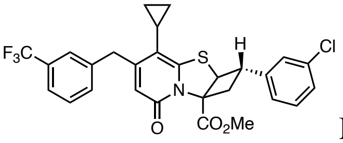

Methyl (2*S*)-2-(3-chlorophenyl)-4-cyclopropyl-7-oxo-5-(3-(trifluoromethyl)benzyl)-2,2a-dihydro-7*H*-cyclobuta[4,5]thiazolo[3,2-*a*]pyridine-8a(1*H*)-carboxylate (2f). Prepared by following the general procedure**,** purified by automated flash column chromatography (25 g SNAP Cartridge) eluting with 0–100% ethyl acetate in heptane. 133 mg of **1f** was converted to 57 mg (32%) of **2f**, isolated as a white solid. IR ($CHCl_3$, cm^{-1}): ν 2955, 1744, 1656, 1577, 1485, 1434, 1304, 1228, 1124, 1033, 764, 732. **^{1}H** NMR (600 MHz, $CDCl_3$) δ 7.53 (d, *J* = 7.6 Hz, 1H), 7.48 (s, 1H), 7.45 (t, *J* = 7.7 Hz, 1H), 7.41 (d, *J* = 7.7 Hz, 1H), 7.33 – 7.29 (m, 2H), 7.28 – 7.27 (m, 1H), 7.23 – 7.20 (m, 1H), 5.97 (s, 1H), 4.16 (d, *J* = 7.4 Hz, 1H), 4.10 (q, *J* = 16.2 Hz, 2H), 3.80 (s, 3H), 3.72 – 3.67 (m, 1H), 3.44 (dd, *J* = 13.2, 9.9 Hz, 1H), 3.02 (ddd, *J* = 13.3, 9.6, 1.1 Hz, 1H), 1.50 – 1.46 (m, 1H), 1.01 – 0.91 (m, 2H), 0.77 – 0.71 (m, 2H). 13C NMR (151 MHz, CDCl3) δ 168.4, 160.9, 156.6, 150.0, 142.7, 139.0, 134.8, 132.8, 131.2 (q, *J* = 32.5 Hz), 130.3, 129.3, 127.8, 127.0, 126.1 (q, *J* = 3.9 Hz), 125.0 (q, *J* = 272.1 Hz), 124.9, 123.8 (q, *J* = 3.9 Hz), 116.2, 113.9, 73.8, 53.5, 52.4, 47.6, 39.1, 35.1, 11.4, 8.3, 8.2. ^{19}F NMR (565 MHz, $CDCl_3$) δ -62.53. HRMS (ESI-TOF) m/z: $[M+H]^+$ Calcd for $C_{28}H_{24}ClF_3NO_3S^+$ 546,1112; observed 546.1086.

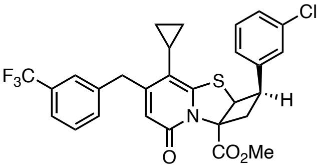

Methyl (2*R*)-2-(3-chlorophenyl)-4-cyclopropyl-7-oxo-5-(3-(trifluoromethyl)benzyl)-2,2a-dihydro-7*H*-cyclobuta[4,5]thiazolo[3,2-*a*]pyridine-8a(1*H*)-carboxylate (3f). Prepared by following the general procedure**,** purified by automated flash column chromatography (25 g SNAP Cartridge) eluting with 0–100% ethyl acetate in heptane. 133 mg of **1f** was converted to 17 mg (9%) of **3f**, isolated as a white solid. IR ($CHCl_3$, cm^{-1}): ν 3007, 1744, 1652, 1574, 1485, 1435, 1304, 1218, 1126, 1034, 756. ^{1}H NMR (600 MHz, $CDCl_3$) δ 7.51 (d, *J* = 7.6 Hz, 1H), 7.47 – 7.43 (m, 2H), 7.37 (d, *J* = 7.6 Hz, 1H), 7.22 (d, *J* = 7.9 Hz, 1H), 7.19 (t, *J* = 7.6 Hz, 1H), 6.88 (d, *J* = 9.4 Hz, 2H), 5.97 (s, 1H), 4.69 (d, *J* = 8.4 Hz, 1H), 4.09 (q, *J* = 7.1 Hz, 1H), 4.03 (q, *J* = 16.2 Hz, 2H), 3.82 (s, 3H), 3.72 (dd, *J* = 13.8, 5.5 Hz 1H), 2.87 (dd, *J* = 14.1, 5.8 Hz, 1H), 0.85 – 0.82 (m, 1H), 0.72 – 0.68 (m, 1H), 0.50 0.46 – (m, 1H), 0.22 – 0.17 (m, 1H). ^{13}C NMR (151 MHz, $CDCl_3$) δ 168.7, 160.5, 156.8, 150.4, 140.2, 139.0, 134.4, 132.7, 131.25 (q, *J* = 30.7 Hz), 129.6, 129.2, 128.1, 127.5, 126.8, 126.1 (q, *J* = 3.8 Hz), 125.0 (q, *J* = 273.8 Hz), 123.8 (q, *J* = 3.9 Hz), 116.0, 113.2, 74.4, 53.5, 50.3, 41.7, 39.0, 33.2, 10.9, 9.1, 7.6. ^{19}F NMR (565 MHz, $CDCl_3$) δ -62.53. HRMS (ESI-TOF) m/z: $[M+H]^+$ Calcd for $C_{28}H_{24}ClF_3NO_3S^+$ 546,1112; observed 546.1083.

Synthesis of compound 2g and 3g

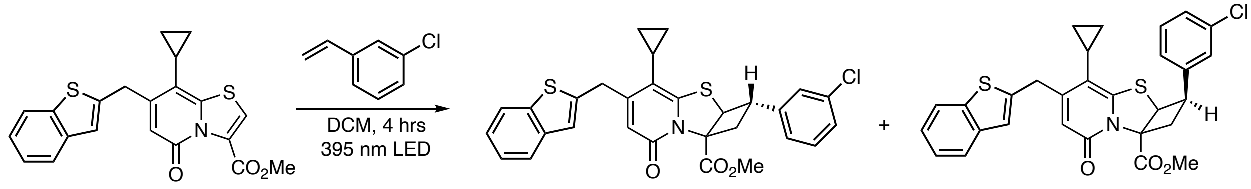

1g **2g** **(Crude: 4:1 dr)** **3g**

Fig. S22. Synthesis of compound 2g and 3g

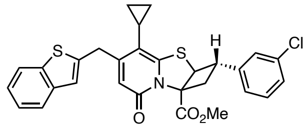

Methyl (2*S*)-5-(benzo[*b*]thiophen-2-ylmethyl)-2-(3-chlorophenyl)-4-cyclopropyl-7-oxo-2,2a-dihydro-7*H*-cyclobuta[4,5]thiazolo[3,2-*a*]pyridine-8a(1*H*)-carboxylate (2g). Prepared by following the general procedure, purified by automated flash column chromatography (25 g SNAP Cartridge) eluting with 0–100% ethyl acetate in heptane. 100 mg of **1g** was converted to 80 mg (59%) of **2g**, isolated as a white solid. IR ($CHCl_3$, cm^{-1}): ν 3006, 2955, 1744, 1655, 1575, 1484, 1435, 1328, 1227, 1117, 1032, 753. ^{1}H NMR (600 MHz, $CDCl_3$) δ 7.77 (d, *J* = 7.9 Hz, 1H), 7.69 (d, *J* = 6.2 Hz 1H), 7.34 – 7.27 (m, 5H), 7.22 (dt, *J* = 6.6, 1.5 Hz, 1H), 7.10 (s, 1H), 6.26 (s, 1H), 4.25 (q, *J* = 16.1 Hz, 2H), 4.17 (d, *J* = 7.4 Hz, 1H), 3.81 (s, 3H), 3.69 (td, *J* = 9.7, 7.3 Hz, 1H), 3.45 (dd, *J* = 13.3, 9.9 Hz, 1H), 3.04 (dd, *J* = 13.3, 9.4 Hz, 1H), 1.59 – 1.63 (m, 1H), 1.04 – 1.00 (m, 1H), 0.97 – 0.92 (m, 1H), 0.77 – 0.71 (m, 2H). ^{13}C NMR (151 MHz, $CDCl_3$) δ 168.3, 161.0, 156.0, 150.1, 142.7, 141.5, 140.0, 139.8, 134.8, 130.3, 127.8, 127.0, 124.9, 124.4, 124.1, 123.2, 122.9, 122.2, 116.0, 114.0, 73.9, 53.5, 52.4, 47.6, 35.2, 34.3, 11.3, 8.1. HRMS (ESI-TOF) m/z: $[M+H]^+$ Calcd for $C_{29}H_{25}ClNO_3S2^+$ 534,0959; observed 534.0940.

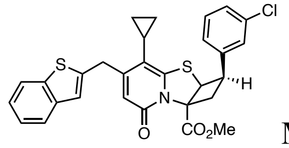

Methyl (2*R*)-5-(benzo[*b*]thiophen-2-ylmethyl)-2-(3-chlorophenyl)-4-cyclopropyl-7-oxo-2,2a-dihydro-7*H*-cyclobuta[4,5]thiazolo[3,2-*a*]pyridine-8a(1*H*)-carboxylate (3g). Prepared by following the general procedure, purified by automated flash column chromatography (25 g SNAP Cartridge) eluting with 0–100% ethyl acetate in heptane. 133 mg of **1g** was converted to 24 mg (18%) of **3g**, isolated as a white solid. IR ($CHCl_3$, cm^{-1}): ν 3003, 2953, 1744, 1654, 1574, 1484, 1435, 1331, 1233, 1133, 1032, 728. ^{1}H NMR (400 MHz, $CDCl_3$) δ 7.77 (d, *J* = 6.9, 1.3 Hz, 1H), 7.69 (d, *J* = 6.7, 1.2 Hz, 1H), 7.35 – 7.27 (m, 3H), 7.21 – 7.15 (m, 2H), 7.06 (d, *J* = 1.0 Hz, 1H), 6.91 (d, *J* = 1.9 Hz, 1H), 6.90 – 6.86 (m, 1H), 6.24 (s, 1H), 4.69 (d, *J* = 8.5 Hz, 1H), 4.22 (q, *J* = 1.9 Hz, 2H), 4.13 – 4.06 (m, 1H), 3.83 (s, 3H), 3.76 – 3.72 (m, 1H), 2.88 (ddd, *J* = 14.0, 6.2, 1.2 Hz, 1H), 1.36 – 1.30 (m, 1H), 0.87 – 0.81 (m, 1H), 0.75 – 0.68 (m, 1H), 0.53 – 0.47 (m, 1H), 0.23 – 0.17 (m, 1H). ^{13}C NMR (100 MHz, $CDCl_3$) δ 168.7, 160.5, 156.0, 150.4, 141.6, 140.2, 140.0, 139.8, 134.4, 129.6, 128.2, 127.5, 126.7, 124.4, 124.0, 123.1, 122.8, 122.2, 115.9, 113.1, 74.4, 53.5, 50.3, 41.7, 34.2, 33.3, 10.8, 8.8, 7.4. HRMS (ESI-TOF) m/z: $[M+H]^+$ Calcd for $C_{29}H_{25}ClNO_3S_2^+$ 534,0959; observed 534.0915.

Synthesis of compound 2h and 3h

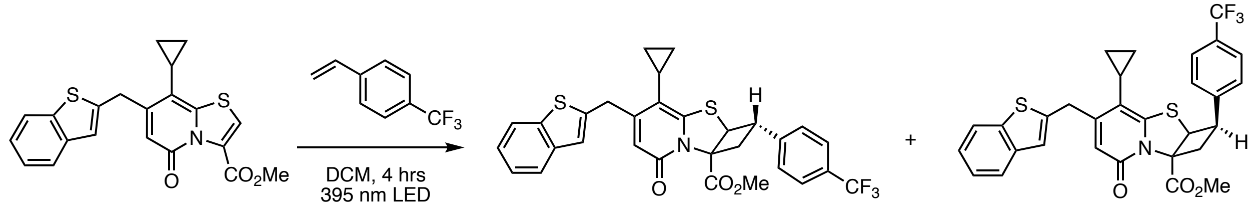

1g **2h** **3h**

(Crude: 4:1 dr)

Fig. S23. Synthesis of compound 2h and 3h

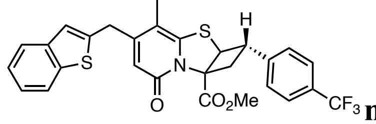

methyl (2*S*)-5-(benzo[*b*]thiophen-2-ylmethyl)-4-cyclopropyl-7-oxo-2-(4-(trifluoromethyl)phenyl)-2,2a-dihydro-7*H*-cyclobuta[4,5]thiazolo[3,2-*a*]pyridine-8a(1*H*)-carboxylate (2h). Prepared by following the general procedure, purified by automated flash column chromatography (10 g SNAP Cartridge) eluting with 0–100% ethyl acetate in heptane. 101 mg of **1g** was converted to 61 mg (42%) of **2h**, isolated as a white solid. IR ($CHCl_3$, cm^{-1}): ν 3006, 2954, 1744, 1657, 1580, 1486, 1434, 1325, 1228, 1121, 1068, 750. ^{1}H NMR (400 MHz, $CDCl_3$) δ 7.68 (d, *J* = 7.8 Hz, 1H), 7.60 (d, *J* = 7.5 Hz, 1H), 7.55 (d, *J* = 8.1 Hz, 2H), 7.36 (d, *J* = 8.1 Hz, 2H), 7.26 – 7.17 (m, 2H), 7.02 (s, 1H), 6.18 (s, 1H), 4.20 (q, *J* = 16.1 Hz, 2H), 4.11 (d, *J* = 7.4 Hz, 1H). 3.72 – 3.67 (m, 4H), 3.40 (dd, *J* = 13.3, 9.9 Hz, 1H), 2.99 (dd, *J* = 12.5, 9.6 Hz, 1H), 1.57 – 1.50 (m, 1H), 0.97 – 0.84 (m, 2H), 0.67 – 0.65 (m, 2H). ^{13}C NMR (100 MHz, $CDCl_3$) δ 168.3, 160.8, 155.9, 149.8, 144.5, 141.4, 139.9, 139.7, 129.9, 129.6, 129.3, 127.0 (2C), 125.8 (q, *J* = 3.7 Hz, 2C), 124.3, 124.05, 124.0 (q, *J* = 272.6 Hz),123.1, 122.8, 122.1, 73.8, 53.4, 52.2, 47.5, 35.1, 34.1, 11.2, 8.0 (2C). ^{19}F NMR (376 MHz, $CDCl_3$) δ -62.50. HRMS (ESI-TOF) m/z: $[M+H]^+$ Calcd for $C_{30}H_{25}F_3NO_3S_2^+$ 568.1222; observed 568.1187.

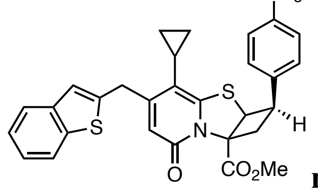

methyl (2*R*)-5-(benzo[*b*]thiophen-2-ylmethyl)-4-cyclopropyl-7-oxo-2-(4-(trifluoromethyl)phenyl)-2,2a-dihydro-7*H*-cyclobuta[4,5]thiazolo[3,2-*a*]pyridine-8a(1*H*)-carboxylate (3h). Prepared by following the general procedure, purified by automated flash column chromatography (10 g SNAP Cartridge) eluting with 0–100% ethyl acetate in heptane. 101 mg of **1g** was converted to 13 mg (9%) of **3h**, isolated as a white solid. IR ($CHCl_3$, cm^{-1}): ν 3003, 2953, 1744, 1655, 1619, 1483, 1459, 1325, 1235, 1119, 1068, 751. ^{1}H NMR (400 MHz, $CDCl_3$) δ 7.60 (d, *J* = 7.8 Hz, 1H), 7.51 (d, *J* = 7.0 Hz, 1H), 7.33 (d, *J* = 8.2 Hz, 2H), 7.18 – 7.10 (m, 2H), 6.93 (d, *J* = 8.5 Hz, 2H), 6.88 (s, 1H), 6.05 (s, 1H), 4.55 (d, *J* = 8.4 Hz, 1H), 4.08 – 3.97 (m, 3H), 3.66 (s, 3H), 3.59 (ddd, *J* = 13.9, 9.9, 1.7 Hz, 1H), 2.78 (dd, *J* = 13.9, 7.5 Hz, 1H), 1.14 – 1.07 (m, 1H), 0.85 – 0.48 (m, 2H), 0.29 – 0.22 (m, 1H), 0.01 – -0.04 (m, 1H). ^{13}C NMR (151 MHz, $CDCl_3$) δ 168.5, 160.3, 155.9, 150.1, 141.9, 141.5, 139.8, 139.6, 129.5 (q, *J* = 32.6 Hz), 128.4 (2C), 125.1 (q, *J* = 3.7 Hz, 2C), 124.3, 124.06, 124.00 (q, *J* = 272.2 Hz), 123.0, 122.6, 122.1, 115.7, 113.0, 74.2, 53.4, 50.2, 41.4, 34.1, 32.9, 10.7, 8.4, 7.5.^{19}F NMR (376 MHz, $CDCl_3$) δ -62.54. HRMS (ESI-TOF) m/z: $[M+H]^+$ Calcd for $C_{30}H_{25}F_3NO_3S_2^+$ 568.1222; observed 568.1173.

Synthesis of compound 2i and 3i

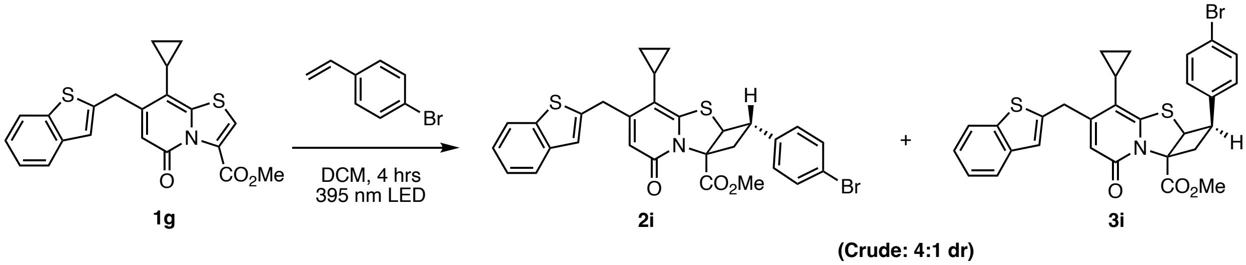

Fig. S24. Synthesis of compound 2i and 3i

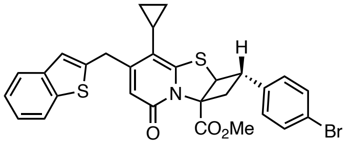

methyl (2*S*)-5-(benzo[*b*]thiophen-2-ylmethyl)-2-(4-bromophenyl)-4-cyclopropyl-7-oxo-2,2a-dihydro-7*H*-cyclobuta[4,5]thiazolo[3,2-*a*]pyridine-8a(1*H*)-carboxylate (2i). Prepared by following the general procedure, purified by automated flash column chromatography (10 g SNAP Cartridge) eluting with 0–100% ethyl acetate in heptane. 95 mg of **1g** was converted to 76 mg (55%) of **2i**, isolated as a white solid. IR ($CHCl_3$, cm^{-1}): ν 3003, 2952, 1743, 1655, 1579, 1487, 1434, 1327, 1225, 1113, 1072, 751. ^1H NMR (400 MHz, CDCl3) δ 7.68 (d, *J* = 8.3 Hz, 1H), 7.60 (d, *J* = 6.8 Hz, 1H), 7.40 (d, *J* = 8.4 Hz, 2H), 7.26 – 7.19 (m, 2H), 7.11 (d, *J* = 8.5 Hz, 2H), 7.01 (s, 1H), 6.16 (s, 1H), 4.19 (q, *J* = 16.2 Hz, 2H), 4.05 (d, *J* = 7.3 Hz, 1H), 3.72 (s, 3H), 3.57 (dd, *J* = 9.7, 7.4 Hz, 1H), 3.35 (dd, *J* = 13.1, 9.9 Hz, 1H), 2.94 (dd, *J* = 12.7, 9.1 Hz, 1H), 1.55 – 1.48 (m, 1H), 0.96 – 0.82 (m, 2H), 0.66 – 0.64 (m, 2H). ^{13}C NMR (100 MHz, $CDCl_3$) δ 168.4, 160.8, 155.8, 149.9, 141.4, 139.9, 139.7, 139.6, 131.9 (2C), 128.4 (2C), 124.3, 124.0, 123.1, 122.8, 122.1, 121.4, 115.9, 113.7, 73.7, 53.3, 52.4, 47.3, 35.2, 34.1, 11.2, 8.0 (2C). HRMS (ESI-TOF) m/z: $[M+H]^+$ Calcd for $C_{29}H_{25}BrNO_3S_2^+$ 578.0454; observed 578.0412.

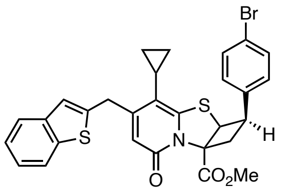

methyl (2*R*)-5-(benzo[*b*]thiophen-2-ylmethyl)-2-(4-bromophenyl)-4-cyclopropyl-7-oxo-2,2a-dihydro-7*H*-cyclobuta[4,5]thiazolo[3,2-*a*]pyridine-8a(1*H*)-carboxylate (3i). Prepared by following the general procedure, purified by automated flash column chromatography (10 g SNAP Cartridge) eluting with 0–100% ethyl acetate in heptane. 95 mg of **1g** was converted to 19 mg (14%) of **3i**, isolated as a white solid. IR ($CHCl_3$, cm^{-1}): ν 3004, 2954, 1743, 1656, 1579, 1485, 1433, 1303, 1233, 1075, 750. ^1H NMR (400 MHz, $CDCl_3$) δ 7.71 (d, *J* = 8.5, 1.3 Hz, 1H), 7.62 (d, *J* = 7.7, 1H), 7.30 – 7.19 (m, 5H), 6.98 (s, 1H), 6.77 (d, *J* = 8.5 Hz, 2H), 6.15 (s, 1H), 4.60 (dt, *J* = 8.4, 1.2 Hz, 1H), 4.13 (q, *J* = 16.0 Hz, 2H), 3.99 (td, *J* = 9.3, 6.5 Hz, 1H), 3.75 (s, 3H), 3.66 (dd, *J* = 13.7, 9.9 Hz, 1H), 2.80 (dd, *J* = 13.9, 6.4 Hz, 1H), 1.27 – 1.21 (m, 1H), 0.79 – 0.66 (m, 2H), 0.44 – 0.38 (m, 1H), 0.24 – 0.17 (m, 1H). ^{13}C NMR (100 MHz, $CDCl_3$) δ 168.6, 160.3, 155.8, 150.4, 141.6, 139.8, 139.7, 136.9, 131.3 (2C), 129.8 (2C), 124.3, 124.0, 123.1, 122.6, 122.2, 121.2, 115.7, 113.0, 74.2, 53.3, 50.2, 41.1, 34.2, 33.1, 10.8, 8.4, 7.6. HRMS (ESI-TOF) m/z: $[M+H]^+$ Calcd for $C_{29}H_{25}BrNO_3S_2^+$ 578.0454; observed 578.0407.

Synthesis of compound 2j and 3j

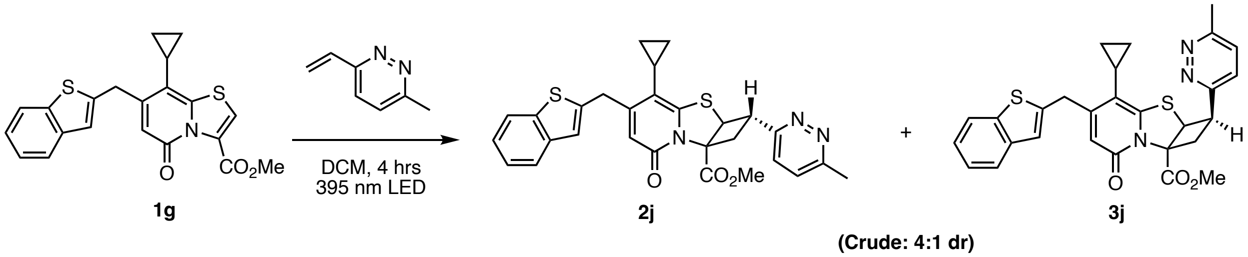

Fig. S25. Synthesis of compound 2j and 3j

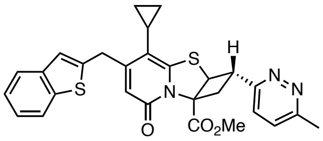

methyl (2*S*)-5-(benzo[*b*]thiophen-2-ylmethyl)-4-cyclopropyl-2-(6-methylpyridazin-3-yl)-7-oxo-2,2a-dihydro-7*H*-cyclobuta[4,5]thiazolo[3,2-*a*]pyridine-8a(1*H*)-carboxylate (2j). Prepared by following the general procedure, purified by automated flash column chromatography (10 g SNAP Cartridge) eluting with 0–100% ethyl acetate in heptane. 102 mg of **1g** was converted to 55 mg (41%) of **2j**, isolated as a white solid. IR ($CHCl_3$, cm^{-1}): ν 3055, 3003, 1743, 1653, 1578, 1485, 1434, 1326, 1227, 1117, 1068, 750. ^1H NMR (400 MHz, $CDCl_3$) δ 7.79 (d, J = 8.0 Hz, 1H), 7.71 (d, J = 7.1 Hz, 1H), 7.37 – 7.27 (m, 4H), 7.12 (s, 1H), 6.28 (s, 1H), 4.91 (d, J = 7.3 Hz, 1H), 4.30 (q, J = 16.1 Hz, 2H), 3.98 (td, J = 9.7, 7.3 Hz, 1H), 3.82 (s, 3H), 3.54 (dd, J = 13.0, 9.7 Hz, 1H), 3.13 (dd, J = 13.0, 10.7 Hz, 1H), 2.75 (s, 3H), 1.66 – 1.59 (m, 1H), 1.05 – 0.93 (m, 2H), 0.78 – 0.74 (m, 2H). ^{13}C NMR (151 MHz, $CDCl_3$) δ 167.9, 160.9, 159.1, 158.8, 155.8, 150.3, 141.5, 139.9, 139.7, 127.2, 125.2, 124.3, 124.0, 123.1, 122.8, 122.1, 115.9, 113.7, 74.0, 53.3, 49.2, 47.2, 34.9, 34.1, 22.1, 11.2, 8.00 (2C). HRMS (ESI-TOF) m/z: $[M+H]^+$ Calcd for $C_{28}H_{26}N_3O_3S_2^+$ 516.1410; observed 516.1382.

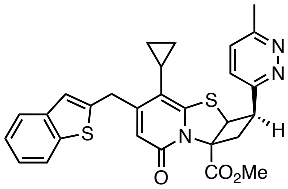

methyl (2*R*)-5-(benzo[*b*]thiophen-2-ylmethyl)-4-cyclopropyl-2-(6-methylpyridazin-3-yl)-7-oxo-2,2a-dihydro-7*H*-cyclobuta[4,5]thiazolo[3,2-*a*]pyridine-8a(1*H*)-carboxylate (3j). Compound **3j** could not be isolated in pure form.

Synthesis of compound 2k and 3k

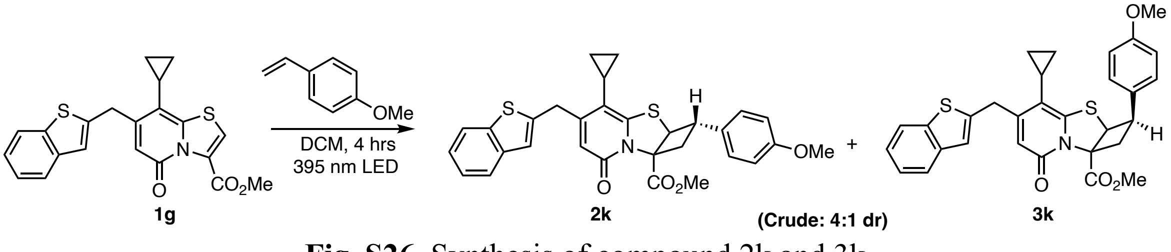

Fig. S26. Synthesis of compound 2k and 3k

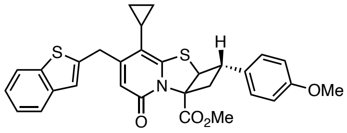

Methyl (2*S*)-5-(benzo[*b*]thiophen-2-ylmethyl)-4-cyclopropyl-2-(4-methoxyphenyl)-7-oxo-2,2a-dihydro-7*H*-cyclobuta[4,5]thiazolo[3,2-*a*]pyridine-8a(1*H*)-carboxylate (2k). Prepared by following the general procedure, purified by automated flash column chromatography (25 g SNAP Cartridge) eluting with 0–100% ethyl acetate in heptane. 100 mg of **1g** was converted to 60 mg (44%) of **2k**, isolated as a white solid. IR ($CHCl_3$, cm^{-1}): ν 3005, 1743, 1655, 1578, 1486, 1436, 1326, 1224, 1119, 1033, 752. ^1H NMR (600 MHz, $CDCl_3$) δ 7.77 (d, *J* = 8.0 Hz, 1H), 7.69 (d, *J* = 7.8 Hz, 1H), 7.35 – 7.31 (m, 1H), 7.30 – 7.25 (m, 4H), 7.10 (s, 1H), 6.92 – 6.90 (m, 2H), 6.25 (s, 1H), 4.31 (q, *J* = 16.0 Hz, 2H), 4.13 (d, *J* = 7.3 Hz, 1H), 3.81 (s, 6H), 3.65 (td, *J* = 9.8, 7.5 Hz, 1H), 3.45 (dd, *J* = 13.2, 9.9 Hz, 1H), 3.02 (ddd, *J* = 13.2, 9.6, 1.1 Hz, 1H), 1.63 – 1.58 (m, 1H), 1.04 – 0.99 (m, 1H), 0.97 – 0.92 (m, 1H), 0.78 – 0.72 (m, 2H). ^{13}C NMR (151 MHz, $CDCl_3$) δ 168.6, 161.0, 159.1, 155.8, 150.4, 141.6, 140.0, 139.8, 132.9, 127.9, 124.4, 124.1, 123.2, 122.9, 122.2, 115.9, 114.3, 113.7, 73.8, 55.4, 53.4, 53.0, 47.5, 35.8, 34.2, 11.3, 8.1, 8.1. HRMS (ESI-TOF) m/z: $[M+H]^+$ Calcd for $C_{30}H_{28}NO_4S2^+$ 530,1454; observed 530.1408.

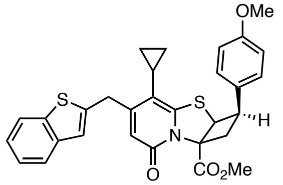

Methyl (2*R*)-5-(benzo[*b*]thiophen-2-ylmethyl)-4-cyclopropyl-2-(4-methoxyphenyl)-7-oxo-2,2a-dihydro-7*H*-cyclobuta[4,5]thiazolo[3,2-*a*]pyridine-8a(1*H*)-carboxylate (3k). Prepared by following the general procedure, purified by automated flash column chromatography (25 g SNAP Cartridge) eluting with 0–100% ethyl acetate in heptane. 100 mg of **1g** was converted to 20 mg (15%) of **3k**, isolated as a white solid. IR ($CHCl_3$, cm^{-1}): ν 3007, 1743, 1654, 1576, 1484, 1436, 1330, 1216, 1105, 1034, 708. ^1H NMR (600 MHz, $CDCl_3$) δ 7.77 (d, *J* = 8.0 Hz, 1H), 7.69 (d, *J* = 7.7 Hz, 1H), 7.35 – 7.27 (m, 3H), 7.06 (s, 1H), 6.88 (d, *J* = 8.7 Hz, 2H), 6.74 (d, *J* = 8.8 Hz, 2H), 6.24 (s, 1H), 4.66 (d, *J* = 8.3 Hz, 1H), 4.21 (q, *J* = 15.9 Hz, 2H), 4.09 – 4.04 (m, 1H), 3.83 (s, 3H), 3.82 – 3.79 (m, 1H), 3.64 (s, 3H), 2.90 – 2.83 (m, 1H), 1.34 – 1.20 (m, 1H), 0.83 – 0.78 (m, 1H), 0.77 – 0.71 (m, 1H), 0.47 – 0.43 (m, 1H), 0.29 – 0.25 (m, 1H). ^{13}C NMR (151 MHz, $CDCl_3$) δ 169.0, 160.5, 158.9, 155.8, 151.1, 142.0, 140.0, 139.8, 130.0, 129.3, 124.4, 124.1, 123.2, 122.6, 122.2, 115.7, 113.7, 112.9, 74.4, 55.3, 53.4, 50.9, 41.2, 34.3, 33.8, 10.9, 8.6, 7.6, 1.1. HRMS (ESI-TOF) m/z: $[M+H]^+$ Calcd for $C_{30}H_{28}NO_4S2^+$ 530,1454; observed 530.1407.

Synthesis of compound 2l and 3l

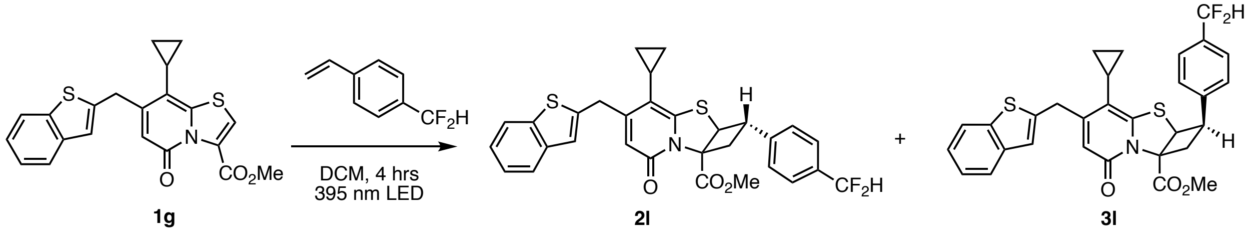

Fig. S27. Synthesis of compound 2l and 3l

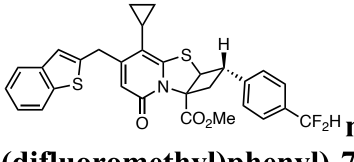
methyl (2*S*)-5-(benzo[*b*]thiophen-2-ylmethyl)-4-cyclopropyl-2-(4-(difluoromethyl)phenyl)-7-oxo-2,2a-dihydro-7*H*-cyclobuta[4,5]thiazolo[3,2-*a*]pyridine-8a(1*H*)-carboxylate (2l). Prepared by following the general procedure, purified by automated flash column chromatography (10 g SNAP Cartridge) eluting with 0–100% ethyl acetate in heptane. 90 mg of **1g** was converted to 49 mg (39%) of **2l**, isolated as a white solid. IR ($CHCl_3$, cm^{-1}): ν 3004, 2954, 1742, 1654, 1578, 1485, 1434, 1302, 1220, 1068, 750. ^1H NMR (600 MHz, $CDCl_3$) δ 7.80 (d, J = 8.0 Hz, 1H), 7.72 (d, J = 7.4 Hz, 1H), 7.50 (dd, J = 58.9, 7.9 Hz, 4H), 7.37 – 7.30 (m, 2H), 7.12 (s, 1H), 6.67 (t, J = 56.4 Hz, 1H), 6.28 (s, 1H), 4.31 (q, J = 16.1 Hz, 2H), 4.21 (d, J = 7.5 Hz, 1H), 3.83 (s, 3H), 3.80 (dd, J = 19.7, 10.4 Hz, 1H), 3.51 (dd, J = 13.3, 9.9 Hz, 1H), 3.09 (dd, J = 13.3, 10.6 Hz, 1H), 1.66 – 1.62 (m, 1H), 1.07 – 0.95 (m, 2H), 0.80 – 0.74 (m, 2H). ^{13}C NMR (151 MHz, $CDCl_3$) δ 168.3, 160.8, 155.8, 149.9, 143.4, 141.4, 139.9, 139.7, 133.6 (t, J = 22.5 Hz), 127.0 (2C), 126.1 (t, J = 6.0 Hz, 2C), 124.3, 124.0, 123.1, 122.8, 122.1, 116.0, 114.4 (t, J = 238.8 Hz), 113.7, 73.8, 53.3, 52.3, 47.6, 35.2, 34.1, 11.2, 8.0 (2C). ^{19}F NMR (565 MHz, $CDCl_3$) δ -110.49 (d, J = 56.7 Hz, 2F). HRMS (ESI-TOF) m/z: $[M+H]^+$ Calcd for $C_{30}H_{26}F_2NO_3S_2^+$ 550.1317; observed 550.1280.

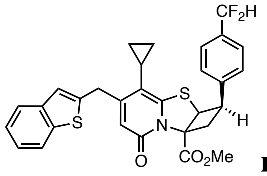
methyl (2*R*)-5-(benzo[*b*]thiophen-2-ylmethyl)-4-cyclopropyl-2-(4-(difluoromethyl)phenyl)-7-oxo-2,2a-dihydro-7*H*-cyclobuta[4,5]thiazolo[3,2-*a*]pyridine-8a(1*H*)-carboxylate (3l). Prepared by following the general procedure, purified by automated flash column chromatography (10 g SNAP Cartridge) eluting with 0–100% ethyl acetate in heptane. 90 mg of **1g** was converted to 8 mg (6%) of **3l**, isolated as a white solid. IR ($CHCl_3$, cm^{-1}): ν 3003, 2954, 1743, 1652, 1576, 1483, 1434, 1302, 1223, 1105, 1024, 750. ^1H NMR (600 MHz, $CDCl_3$) δ 7.57 (d, J = 7.9 Hz, 1H), 7.49 (d, J = 8.7 Hz, 1H), 7.15 – 7.08 (m, 4H), 6.86 – 6.85 (m, 3H), 6.27 (t, J = 56.4 Hz, 1H), 6.03 (s, 1H), 4.51 (d, J = 8.5 Hz, 1H), 4.05 – 3.93 (m, 3H), 3.63 (s, 3H), 3.55 (dd, J = 14.0, 11.5 Hz, 1H), 2.75 (dd, J = 13.9, 6.3 Hz, 1H), 1.09 – 1.04 (m, 1H), 0.62 – 0.51 (m, 2H), 0.24 – 0.21 (m, 1H), 0.02 – -0.03 (m, 1H). ^{13}C NMR (151 MHz, $CDCl_3$) δ 168.6, 160.3, 155.9, 150.4, 141.6, 140.7, 139.8, 139.7, 133.3 (t, J = 22.5 Hz), 128.4 (2C), 125.4 (t, J = 6.0 Hz, 2C), 124.3, 124.0, 123.0, 122.6, 122.1, 115.6, 114.4 (t, J = 238.7 Hz), 113.0, 74.3, 53.3, 50.3, 41.4, 34.1, 32.9, 10.7, 8.4, 7.5. ^{19}F NMR (565 MHz, $CDCl_3$) δ -110.45 (d, J = 5.1 Hz, 1F), -110.55 (d, J = 5.3 Hz, 1F). HRMS (ESI-TOF) m/z: $[M+H]^+$ Calcd for $C_{30}H_{26}F_2NO_3S_2^+$ 550.1317; observed 550.1258.

Synthesis of compound 2m and 3m

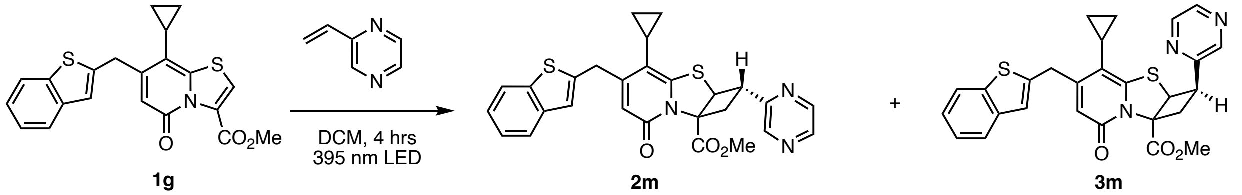

1g 2m 3m

(Crude: 4:1 dr)

Fig. S28. Synthesis of compound 2m and 3m

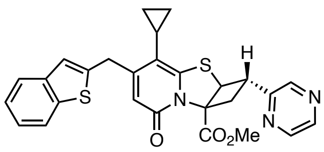

methyl (2*S*)-5-(benzo[*b*]thiophen-2-ylmethyl)-4-cyclopropyl-7-oxo-2-(pyrazin-2-yl)-2,2a-dihydro-7*H*-cyclobuta[4,5]thiazolo[3,2-*a*]pyridine-8a(1*H*)-carboxylate (2m). Prepared by following the general procedure**,** purified by automated flash column chromatography (10 g SNAP Cartridge) eluting with 0–100% ethyl acetate in heptane. 100 mg of **1g** was converted to 75 mg (59%) of **2m**, isolated as a white solid. IR ($CHCl_3$, cm^{-1}): ν 3004, 2954, 1742, 1654, 1578, 1485, 1434, 1302, 1220, 1112, 1026, 750. ^1H NMR (400 MHz, $CDCl_3$) δ 8.83 (dd, *J* = 2.5, 1.5 Hz, 1H), 8.69 (dd, *J* = 9.0, 2.0 Hz, 2H), 7.94 (dd, *J* = 33.1, 8.0 Hz, 2H), 7.56 – 7.47 (m, 3H), 6.47 (s, 1H), 4.88 (d, *J* = 7.2 Hz, 1H), 4.49 (q, *J* = 16.1 Hz, 2H), 4.09 – 4.02 (m, 4H), 3.75 (dd, *J* = 12.9, 9.7 Hz, 1H), 3.25 (ddd, *J* = 13.0, 9.3, 1.0 Hz, 1H), 1.85 – 1.79 (m, 1H), 1.25 – 1.12 (m, 2H), 0.99 – 0.91 (m, 2H). ^{13}C NMR (151 MHz, $CDCl_3$) δ 168.0, 160.9, 155.8, 154.6, 150.0, 144.7, 143.7, 143.6, 141.4, 139.9, 139.7, 124.3, 124.0, 123.1, 122.8, 122.1, 116.0, 113.8, 74.0, 53.3, 49.4, 46.5, 34.5, 34.1, 11.2, 8.0. HRMS (ESI-TOF) m/z: $[M+H]^+$ Calcd for $C_{27}H_{24}N_3O_3S_2^+$ 502.1254; observed 502.1217.

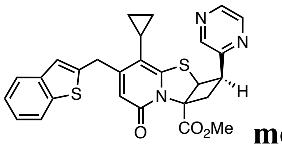

methyl (2*R*)-5-(benzo[*b*]thiophen-2-ylmethyl)-4-cyclopropyl-7-oxo-2-(pyrazin-2-yl)-2,2a-dihydro-7*H*-cyclobuta[4,5]thiazolo[3,2-*a*]pyridine-8a(1*H*)-carboxylate (3m). Compound **3m** could not be isolated in pure form.

Synthesis of compound 2n and 3n

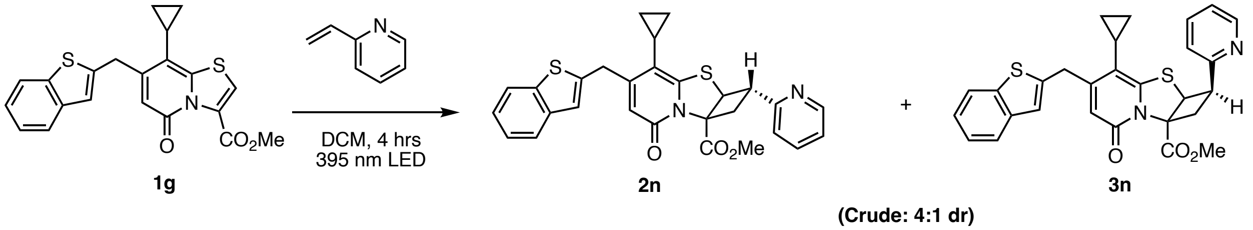

Fig. S29. Synthesis of compound 2n and 3n

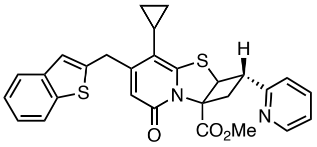

methyl (2*S*)-5-(benzo[*b*]thiophen-2-ylmethyl)-4-cyclopropyl-7-oxo-2-(pyridin-2-yl)-2,2a-dihydro-7*H*-cyclobuta[4,5]thiazolo[3,2-*a*]pyridine-8a(1*H*)-carboxylate (2n). Prepared by following the general procedure**,** purified by automated flash column chromatography (10 g SNAP Cartridge) eluting with 0–100% ethyl acetate in heptane followed by 0-20% methanol in dichloromethane. 80 mg of **1g** was converted to 72 mg (71%) of **2n**, isolated as a white solid. IR ($CHCl_3$, cm^{-1}): ν 3005, 2954, 1743, 1654, 1579, 1485, 1434, 1302, 1225, 1116, 1030, 750. ^1H NMR (400 MHz, $CDCl_3$) δ 8.66 (d, *J* = 4.6 Hz, 1H), 7.80 (d, *J* = 7.8 Hz, 1H), 7.73 – 7.65 (m, 2H), 7.37 – 7.31 (m, 2H), 7.24 – 7.20 (m, 2H), 7.12 (s, 1H), 6.28 (s, 1H), 4.70 (d, *J* = 7.3 Hz, 1H), 4.31 (q, *J* = 16.1 Hz, 2H), 3.88 – 3.81 (m, 3H), 3.54 (dd, *J* = 12.9, 9.8 Hz, 1H), 3.06 (dd, *J* = 11.9, 9.5 Hz, 1H), 1.66 – 1.59 (m, 1H), 1.05 – 0.93 (m, 2H), 0.78 – 0.75 (m, 2H). ^{13}C NMR (151 MHz, $CDCl_3$) δ 168.2, 161.0, 159.1, 155.7, 150.6, 149.8, 141.5, 139.9, 139.7, 136.6,

124.3, 123.9, 123.1, 122.7, 122.3, 122.1, 121.8, 115.8, 113.8, 74.0, 53.2, 49.7, 48.9, 34.7, 34.1, 11.2, 8.0, 7.9. HRMS (ESI-TOF) m/z: $[M+H]^+$ Calcd for $C_{28}H_{25}N_2O_3S_2^+$ 501.1301; observed 501.1275.

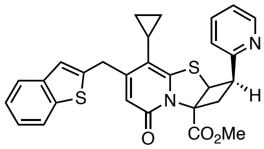
methyl (2*R*)-5-(benzo[*b*]thiophen-2-ylmethyl)-4-cyclopropyl-7-oxo-2-(pyridin-2-yl)-2,2a-dihydro-7*H*-cyclobuta[4,5]thiazolo[3,2-*a*]pyridine-8a(1*H*)-carboxylate (3n). Prepared by following the general procedure**,** purified by automated flash column chromatography (10 g SNAP Cartridge) eluting with 0–100% ethyl acetate in heptane followed by 0-20% methanol in dichloromethane. 80 mg of **1g** was converted to 5 mg (5%) of **3n**, isolated as a white solid. IR ($CHCl_3$, cm^{-1}): ν 3004, 2954, 1743, 1651, 1573, 1484, 1434, 1302, 1222, 1101, 1032, 750. ^1H NMR (400 MHz, $CDCl_3$) δ 8.25 (d, J = 4.4 Hz, 1H), 7.55 (d, J = 7.9 Hz, 1H), 7.46 (d, J = 7.9 Hz, 1H), 7.34 (td, J = 7.7, 1.8 Hz, 1H), 7.14 – 7.07 (m, 2H), 6.89 – 6.80 (m, 3H), 6.00 (s, 1H), 4.53 (d, J = 8.5 Hz, 1H), 4.04 – 3.91 (m, 3H), 3.62 (s, 3H), 3.51 (dd, J = 13.6, 9.7 Hz, 1H), 2.94 (dd, J = 12.9, 6.7 Hz, 1H), 1.10 – 1.03 (m, 1H), 0.61 – 0.47 (m, 2H), 0.29 – 0.22 (m, 1H), 0.04 – -0.02 (m, 1H). ^{13}C NMR (151 MHz, $CDCl_3$) δ 168.9, 160.4, 158.1, 155.4, 150.9, 149.1, 141.8, 139.9, 139.7, 136.1, 124.3, 123.9, 123.0, 122.5, 122.5, 122.1, 121.8, 115.6, 112.4, 73.8, 53.3, 49.8, 42.7, 34.1, 32.5, 10.7, 8.3, 7.5. HRMS (ESI-TOF) m/z: $[M+H]^+$ Calcd for $C_{28}H_{25}N_2O_3S_2^+$ 501.1301; observed 501.1253.

General Procedure for the hydrolysis: Synthesis of TriPcides.

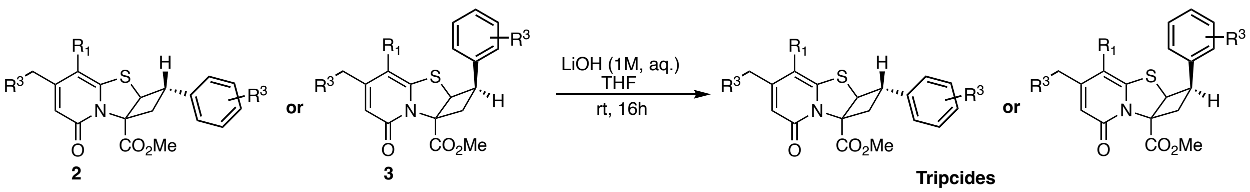

Fig. S30. General Procedure for the hydrolysis: Synthesis of TriPcides

Methyl esters of general structure **2** or **3** (0.24 mmol) was charged into a round bottom flask and added THF (2 mL). LiOH (1M, aq. 0.97 mmol) was added to the reaction mixture and stirred at room temperature for 16 hours. The reaction mixture was neutralized with HCl 1M and extracted with EtOAc (15 mL x 2). Combined organic layers were dried over anhydrous Na_2SO_4 and evaporated under reduced pressure to yield the crude product. The residue was re-dissolved in DMSO (1 mL) and purified with preparative HPLC (H_2O/MeCN + 0.75% HCOOH, 20-100% in 45 min, 100% in 10 min). The pure product was diluted with water (10 mL) and freeze-dried.

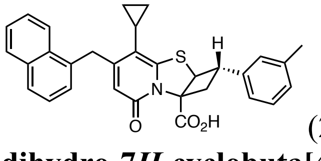
(2*S*)-4-cyclopropyl-5-(naphthalen-1-ylmethyl)-7-oxo-2-(*m*-tolyl)-2,2a-dihydro-7*H*-cyclobuta[4,5]thiazolo[3,2-*a*]pyridine-8a(1*H*)-carboxylic acid (IL262). Prepared

by following the general procedure. 59 mg of **2a** was converted to 37 mg (64%) of **IL262**, isolated as a white solid. IR ($CHCl_3$, cm^{-1}): ν 3008, 2921, 1728, 1628, 1544, 1487, 1426, 1399, 1217, 1194, 755. 1H NMR (600 MHz, $(CD_3)_2SO$) δ 13.33 (s, 1H), 8.00 – 7.97 (m, 1H), 7.96 – 7.92 (m, 1H), 7.90 (d, *J* = 8.3 Hz, 1H), 7.58 – 7.51 (m, 3H), 7.43 (dd, *J* = 7.1, 1.2 Hz, 1H), 7.25 (t, *J* = 7.6 Hz, 1H), 7.15 (s, 1H), 7.13 (d, *J* = 8.1 Hz, 1H), 7.08 (d, *J* = 7.7 Hz, 1H), 5.25 (s, 1H), 4.56 (d, *J* = 7.5 Hz, 1H), 4.50 (s, 2H), 3.64 (td, *J* = 9.8, 7.5 Hz, 1H), 3.05 (dd, *J* = 12.7, 10.0 Hz, 1H), 2.72(dd, *J* = 12.6, 9.9 Hz, 1H), 2.30 (s, 3H), 1.82 – 1.77 (m, 1H), 0.99 – 0.93 (m, 2H), 0.87 – 0.83 (m, 1H), 0.77 – 0.73 (m, 1H). 13C NMR (151 MHz, $(CD_3)_2SO$) δ 168.9, 159.5, 157.1, 149.9, 141.0, 137.7, 134.4, 133.4, 131.6, 128.6, 128.5, 127.7, 127.6, 127.3, 127.3, 126.4, 125.8, 125.7, 124.1, 123.7, 113.8, 112.7, 73.1, 51.0, 46.9, 35.3, 35.1, 21.0, 10.8, 7.8, 7.5. HRMS (ESI-TOF) m/z: $[M+H]^+$ Calcd for $C_{31}H_{28}NO_3S^+$ 494,1784; observed 494.1794.

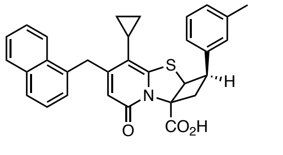

(2*R*)-4-cyclopropyl-5-(naphthalen-1-ylmethyl)-7-oxo-2-(*m*-tolyl)-2,2a-dihydro-7*H*-cyclobuta[4,5]thiazolo[3,2-*a*]pyridine-8a(1*H*)-carboxylic acid (IL263). Prepared by following the general procedure. 21 mg of **3a** was converted to 9 mg (44%) of **IL263**, isolated as a white solid. IR ($CHCl_3$, cm^{-1}): ν 3009, 2933, 1727, 1654, 1597, 1495, 1441, 1216, 1004, 753. ^1H NMR (600 MHz, $(CD_3)_2SO$) δ 8.01 – 7.96 (m, 1H), 7.89 (dd, *J* = 9.4, 6.7 Hz, 2H), 7.57 – 7.50 (m, 3H), 7.37 (d, *J* = 8.1 Hz, 1H), 7.19 (t, *J* = 7.6 Hz, 1H), 7.09 (d, *J* = 7.6 Hz, 1H), 6.78 (d, *J* = 7.9 Hz, 1H), 6.73 (s, 1H), 5.30 (s, 1H), 4.94 (d, *J* = 8.7 Hz, 1H), 4.42 (q, *J* = 17.4 Hz, 2H), 4.05 (td, *J* = 9.8, 6.0 Hz, 1H), 3.42 (ddd, *J* = 13.7, 9.9, 1.6 Hz, 1H), 2.58 (dd, *J* = 14.6, 6.1 Hz, 1H), 1.50– 1.46 (m, 1H), 0.84 – 0.80 (m, 1H), 0.67 – 0.61 (m, 1H), 0.45– 0.41 (m, 1H), 0.23 – 0.17 (m, 1H). ^{13}C NMR (151 MHz, $(CD_3)_2SO$) δ 169.2, 159.0, 156.9, 150.5, 138.5, 137.0, 134.5, 133.4, 131.5, 128.7, 128.4, 127.8, 127.5, 127.3 (2C), 125.8, 125.7, 125.4, 123.9, 113.6, 111.5, 73.9, 49.8, 41.0, 35.2, 32.2, 21.1, 10.3, 8.5, 6.8. HRMS (ESI-TOF) m/z: $[M+H]^+$ Calcd for $C_{31}H_{28}NO_3S^+$ 494,1784; observed 494.1778.

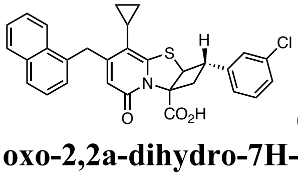

(2S)-2-(3-chlorophenyl)-4-cyclopropyl-5-(naphthalen-1-ylmethyl)-7-oxo-2,2a-dihydro-7H-cyclobuta[4,5]thiazolo[3,2-a]pyridine-8a(1H)-carboxylic acid (NQA8). Prepared by following the general procedure. 80 mg of **2b** was converted to 43 mg (55%) of **NQA8**, isolated as a white solid. IR (KBr, cm^{-1}): ν 3468, 3077, 3041, 3005, 1924, 1727, 1624, 1544, 1493, 1426, 1256, 1234, 198, 779, 754^1H NMR (600 MHz, $(CD_3)_2SO$) δ 8.00 – 7.97 (m, 1H), 7.93 (d, *J* = 7.4 Hz, 1H), 7.90 (d, *J* = 8.2 Hz, 1H), 7.55 (ddd, *J* = 6.5, 5.2, 3.3 Hz, 2H), 7.52 (t, *J* = 6.3 Hz, 1H), 7.42 (t, *J* = 5.3 Hz, 2H), 7.39 (d, *J* = 7.8 Hz, 1H), 7.33 (t, *J* = 9.3 Hz, 2H), 5.26 (s, 1H), 4.68 (d, *J* = 7.6 Hz, 1H), 4.50 (s, 2H), 3.78 – 3.71 (m, 1H), 3.03 (dd, *J* = 12.7, 10.0 Hz, 1H), 2.75 (dt, *J* = 18.5, 9.2 Hz, 1H), 1.83 – 1.75 (m, 1H), 1.00 – 0.92 (m, 2H), 0.88 – 0.83 (m, 1H), 0.78 – 0.72 (m, 1H). ^{13}C NMR (151 MHz, $(CD_3)_2SO$) δ 168.8, 159.6, 157.3, 149.8, 143.7, 134.4, 133.5, 133.3, 131.6, 130.5, 128.7, 127.8, 127.4, 127.0, 126.7, 126.4, 125.7, 125.9, 125.5, 124.1, 113.9, 112.7, 73.0, 50.6, 46.3, 35.3, 10.8, 7.9, 7.5. HRMS (ESI-TOF) m/z: $[M+H]^+$ Calcd for $C_{30}H_{25}ClNO_3S^+$ 514,1238; observed 514.1226.

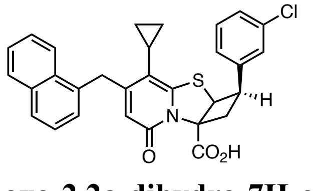

(2R)-2-(3-chlorophenyl)-4-cyclopropyl-5-(naphthalen-1-ylmethyl)-7-oxo-2,2a-dihydro-7H-cyclobuta[4,5]thiazolo[3,2-a]pyridine-8a(1H)-carboxylic acid (NQA9). Prepared by following the general procedure. 40 mg of **3b** was converted to 15 mg (38%) of **NQA9**, isolated as a white solid. IR (KBr, cm^{-1}): ν 3439, 3062, 3003, 1737, 1654, 1629, 1485, 1424, 1217, 1189, 790, 779^{1}H NMR [400 MHz, $(CD_3)_2SO$]: δ 7.98 (dd, *J* = 6.2, 3.3 Hz, 1H), 7.89 (dd, *J* = 9.0, 3.7 Hz, 2H), 7.54 (dt, *J* = 7.4, 3.9 Hz, 3H), 7.38 – 7.32 (m, 3H), 6.98 (dd, *J* = 5.7, 2.5 Hz, 1H), 6.94 (s, 1H), 5.29 (s, 1H), 4.95 (d, *J* = 8.7 Hz, 1H), 4.41 (q, *J* = 17.4 Hz, 2H), 4.11 (td, *J* = 9.3, 6.1 Hz, 1H), 3.43 (dd, *J* = 13.1, 10.1 Hz, 1H), 2.61 (dd, *J* = 13.8, 5.7 Hz, 1H), 1.53 – 1.44 (m, 1H), 0.88 – 0.78 (m, 1H), 0.70 – 0.60 (m, 1H), 0.50 – 0.41 (m, 1H), 0.22 – 0.14 (m, 1H).^{13}C NMR [100 MHz, $(CD_3)_2SO$]: δ 169.1, 159.0, 157.0, 150.0, 141.3, 134.4, 133.5, 132.9, 131.6, 129.8, 128.7, 127.6, 127.3, 126.8, 126.4, 125.8, 125.7, 123.9, 113.8, 111.7, 74.1, 49.7, 40.9, 35.3, 31.9, 10.4, 8.6, 6.8. HRMS (ESI-TOF) m/z: $[M+H]^+$ Calcd for $C_{30}H_{25}ClNO_3S^+$ 514,1238; observed 514.1223.

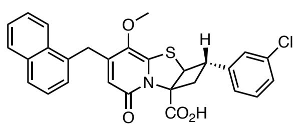

(2*S*)-2-(3-chlorophenyl)-4-methoxy-5-(naphthalen-1-ylmethyl)-7-oxo-2,2a-dihydro-7*H*-cyclobuta[4,5]thiazolo[3,2-*a*]pyridine-8a(1*H*)-carboxylic acid (IL305). Prepared by following the general procedure. 10 mg of **2c** was converted to 5 mg (48%) of **IL305**, isolated as a white solid. IR ($CHCl_3$, cm^{-1}): ν 3009, 2933, 1727, 1654, 1597, 1495, 1441, 1216, 1004, 780, 753. ^{1}H NMR (600 MHz, $(CD_3)_2SO$) δ 13.51 (s, 1H), 7.99 (td, *J* = 7.9, 1.4 Hz, 2H), 7.90 (d, *J* = 8.1 Hz, 1H), 7.59 – 7.51 (m, 3H), 7.48 (dd, *J* = 7.1, 1.5 Hz, 1H), 7.44 – 7.39 (m, 2H), 7.36 – 7.31 (m, 2H), 5.41 (s, 1H), 4.79 (d, *J* = 7.9 Hz, 1H), 4.35 (d, *J* = 2.8 Hz, 2H), 3.85 – 3.80 (m, 1H), 3.78 (s, 3H), 3.02 (dd, *J* = 12.7, 9.9 Hz, 1H), 2.80 (dd, *J* = 12.3, 10.2 Hz, 1H). ^{13}C NMR (151 MHz, $(CD_3)_2SO$) δ 168.5, 158.6, 151.6, 143.4, 141.9, 135.7, 133.9, 133.4, 133.2, 131.5, 130.4, 128.6, 127.8, 127.4, 127.0, 126.7, 126.4, 125.8, 125.7, 125.5, 123.9, 113.8, 73.8, 60.6, 51.6, 46.2, 35.0, 31.8. HRMS (ESI-TOF) m/z: $[M+H]^+$ Calcd for $C_{28}H_{23}ClNO_4S^+$ 504,1031; observed 504.1007.

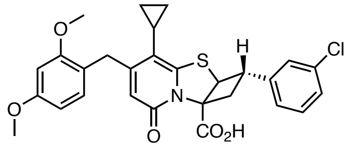

(2*S*)-2-(3-chlorophenyl)-4-cyclopropyl-5-(2,4-dimethoxybenzyl)-7-oxo-2,2a-dihydro-7*H*-cyclobuta[4,5]thiazolo[3,2-*a*]pyridine-8a(1*H*)-carboxylic acid (PS1961). Prepared by following the general procedure. 50 mg of **2d** was converted to 30 mg (61%) of **PS1961**, isolated as a white solid. ^{1}H NMR (400 MHz, $(CD_3)_2SO$) δ 13.38 (s, 1H), 7.44 – 7.38 (m, 2H), 7.33 (ddt, *J* = 7.2, 5.8, 1.5 Hz, 2H), 7.05 (d, *J* = 8.3 Hz, 1H), 6.61 (d, *J* = 2.4 Hz, 1H), 6.53 (dd, *J* = 8.3, 2.4 Hz, 1H), 5.47 (s, 1H), 4.65 (d, *J* = 7.5 Hz, 1H), 3.89 (s, 2H), 3.77 (s, 6H), 3.72 (dd, *J* = 10.0, 7.5 Hz, 1H), 3.05 (dd, *J* = 12.7, 9.8 Hz, 1H), 2.78 (dd, *J* = 12.6, 9.3 Hz, 1H), 1.66 – 1.59 (m, 1H), 0.96 – 0.88 (m, 2H), 0.76 – 0.69 (m, 1H), 0.66 – 0.60 (m, 1H). ^{13}C NMR (100 MHz, $(CD_3)_2SO$) δ 168.9, 159.7, 159.6, 158.0, 157.6, 149.5, 143.7, 133.2, 131.1, 130.4,

126.9, 126.7, 125.5, 118.1, 113.3, 112.7, 104.7, 98.4, 73.0, 55.4, 55.1, 50.6, 46.3, 35.3, 31.8, 10.7, 7.8, 7.5. HRMS (ESI-TOF) m/z: $[M+H]^+$ Calcd for $C_{28}H_{27}ClNO_5S^+$ 524,1293; observed 524.1210.

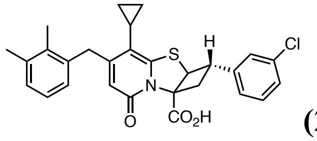

(2*S*)-2-(3-chlorophenyl)-4-cyclopropyl-5-(2,3-dimethylbenzyl)-7-oxo-2,2a-dihydro-7*H*-cyclobuta[4,5]thiazolo[3,2-*a*]pyridine-8a(1*H*)-carboxylic acid (PS1965). Prepared by following the general procedure. 90 mg of **2e** was converted to 36 mg (41%) of **PS1965**, isolated as a white solid. IR ($CHCl_3$, cm^{-1}): ν 3008, 2920, 1728, 1626, 1598, 1489, 1427, 1228, 1216, 1184, 1032, 752. ^{1}H NMR (600 MHz, $(CD_3)_2SO$) δ 7.43 – 7.38 (m, 2H), 7.35 – 7.31 (m, 2H), 7.12 – 7.06 (m, 2H), 7.00 (d, *J* = 7.3 Hz, 1H), 5.31 (s, 1H), 4.66 (d, *J* = 7.6 Hz, 1H), 4.03 (s, 2H), 3.74 (td, *J* = 9.7, 7.5 Hz, 1H), 3.05 (dd, *J* = 12.8, 9.9 Hz, 1H), 2.78 (dd, *J* = 12.9, 9.7 Hz, 1H), 2.50 (s, 4H), 2.28 (s, 3H), 2.09 (s, 3H), 1.72 – 1.67 (m, 1H), 0.98 – 0.92(m, 2H), 0.78 – 0.75 (m, 1H), 0.68 – 0.65 (m, 1H). ^{13}C NMR (151 MHz, $(CD_3)_2SO$) δ 168.9, 159.6, 157.2, 149.7, 143.7, 136.6, 136.3, 134.9, 133.2, 130.4, 128.4, 128.0, 127.0, 126.7, 125.5 (2C), 113.3, 112.6, 73.0, 50.6, 46.3, 40.0, 36.5, 35.3, 20.3, 15.1, 10.7, 7.8, 7.4. HRMS (ESI-TOF) m/z: $[M+H]^+$ Calcd for $C_{28}H_{27}ClNO_3S^+$ 492,1395; observed 492.1376.

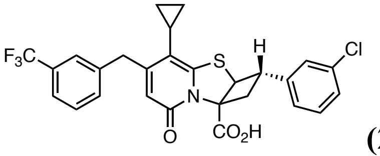

(2*S*)-2-(3-chlorophenyl)-4-cyclopropyl-7-oxo-5-(3-(trifluoromethyl)benzyl)-2,2a-dihydro-7*H*-cyclobuta[4,5]thiazolo[3,2-*a*]pyridine-8a(1*H*)-carboxylic acid (PS1970). Prepared by following the general procedure. 44 mg of **2f** was converted to 36 mg (84%) of **PS1970**, isolated as a white solid. IR ($CHCl_3$, cm^{-1}): ν 3006, 2924, 1726, 1655, 1597, 1485, 1424, 1330, 1213, 1123, 1033, 751. ^{1}H NMR (600 MHz, $(CD_3)_2SO$) δ 7.68 (s, 1H), 7.66 – 7.64 (m, 1H), 7.63 – 7.59 (m, 2H), 7.43 – 7.38 (m, 2H), 7.35 – 7.31 (m, 2H), 5.67 (s, 1H), 4.66 (d, *J* = 7.6 Hz, 1H), 3.72 (td, dd, *J* = 9.6, 7.8 Hz, 1H), 3.06 (dd, *J* = 12.8, 9.9 Hz, 1H), 2.80 (dd, *J* = 12.8, 10.0 Hz, 1H), 1.54 – 1.50 (m, 1H), 0.97 – 0.89 (m, 2H), 0.76 – 0.73 (m, 1H), 0.66 – 0.62 (m, 1H). ^{13}C NMR (151 MHz, $(CD_3)_2SO$) δ 168.8, 159.5, 156.7, 150.4, 143.6, 139.9, 133.5, 133.2, 130.4, 129.6, 129.2 (q, *J* = 31.6 Hz), 126.6, 125.8 (q, *J* = 3.6 Hz), 125.5, 125.1, 123.3 (d, *J* = 4.4 Hz), 121.5, 114.3, 112.4, 73.2, 50.6, 46.3, 37.7, 35.3, 10.8, 8.0, 7.7. ^{19}F NMR (565 MHz, DMSO) δ -62.53 (3F) HRMS (ESI-TOF) m/z: $[M+H]^+$ Calcd for $C_{27}H_{22}ClF_3NO_3S^+$ 532,0956; observed 532.0922.

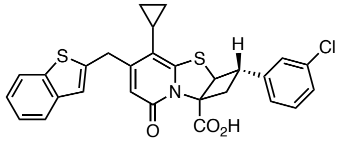

(2*S*)-5-(benzo[*b*]thiophen-2-ylmethyl)-2-(3-chlorophenyl)-4-cyclopropyl-7-oxo-2,2a-dihydro-7*H*-cyclobuta[4,5]thiazolo[3,2-*a*]pyridine-8a(1*H*)-carboxylic acid (PS1962). Prepared by following the general procedure. 130 mg of **2g** was converted to 57 mg (45%) of **PS1962**, isolated as a white solid. IR ($CHCl_3$, cm^{-1}): ν 3059, 3008, 1726, 1626, 1598, 1486, 1425, 1328, 1215, 1188, 1116, 750. ^{1}H NMR (600 MHz, $(CD_3)_2SO$) δ 13.42 (s, 1H), 7.91 (d, *J* = 7.9 Hz, 1H), 7.79 (d, *J* = 7.7 Hz, 1H), 7.44 – 7.28 (m, 7H), 6.03 (s, 1H), 4.68 (d, *J* = 7.5 Hz, 1H), 4.26 (q, *J* = 16.1 Hz, 2H), 3.72 (d, *J* = 9.8, 7.5 Hz, 1H), 3.08 (dd, *J* = 12.8, 9.9 Hz, 1H), 2.82 (dd, *J* = 12.4, 9.2 Hz, 1H), 1.61–1.57 (m, 1H), 1.01 – 0.89 (m, 2H), 0.77 – 0.70 (m, 1H), 0.67 – 0.60 (m, 1H). ^{13}C NMR (151 MHz, $(CD_3)_2SO$) δ 168.8, 159.6, 155.7, 150.6, 143.6, 142.1, 139.6, 139.0, 133.2, 130.4, 127.0, 126.7, 125.5, 124.4, 123.9, 123.1, 122.9, 122.3, 114.4, 112.4, 73.1, 50.6, 46.3, 35.3,

33.2, 10.8, 7.9, 7.6. HRMS (ESI-TOF) m/z: $[M+H]^+$ Calcd for $C_{28}H_{23}ClNO_3S2^+$ 520,0802; observed 520.0795.

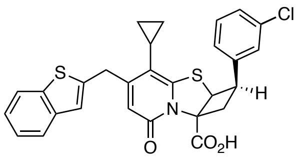

(2*R*)-5-(benzo[*b*]thiophen-2-ylmethyl)-2-(3-chlorophenyl)-4-cyclopropyl-7-oxo-2,2a-dihydro-7*H*-cyclobuta[4,5]thiazolo[3,2-*a*]pyridine-8a(1*H*)-carboxylic acid (ps1963). Prepared by following the general procedure. 42 mg of **3g** was converted to 10 mg (24%) of **PS1963**, isolated as a white solid. IR ($CHCl_3$, cm^{-1}): ν 3060, 3007, 1727, 1649, 1571, 1485, 1425, 1217, 1192, 1103, 752. ^{1}H NMR (400 MHz, $(CD_3)_2SO$) δ 7.90 (d, *J* = 7.8 Hz, 1H), 7.77 (d, *J* = 7.7 Hz, 1H), 7.41 – 7.19 (m, 5H), 6.96 (s, 2H), 6.04 (s, 1H), 4.96 (d, *J* = 8.6 Hz, 1H), 4.25 (s, 1H), 4.12 (td, *J* = 9.6, 6.0 Hz, 1H), 3.47 (dd, *J* = 13.9, 9.7 Hz, 1H), 2.66 (dd, *J* = 13.8, 6.0 Hz, 1H), 1.30 – 1.25 (m, 1H), 0.84 – 0.79 (m, 1H), 0.66 – 0.62 (m, 1H), 0.38 – 0.34 (m, 1H), 0.16 – 0.01 (m, 1H). ^{13}C NMR (100 MHz, $(CD_3)_2SO$) δ 169.0, 159.1, 155.5, 150.7, 142.2, 141.1, 139.6, 139.0, 132.9, 129.7, 127.8, 127.0, 126.6, 124.3, 123.9, 123.1, 122.6, 122.2, 114.4, 111.4, 74.1, 49.6, 40.9, 33.2, 32.1, 10.3, 8.5, 6.9. HRMS (ESI-TOF) m/z: $[M+H]^+$ Calcd for $C_{28}H_{23}ClNO_3S_2^+$ 520,0802; observed 520.0769.

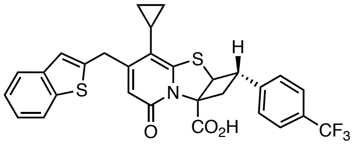

(2*S*)-5-(benzo[*b*]thiophen-2-ylmethyl)-4-cyclopropyl-7-oxo-2-(4-(trifluoromethyl)phenyl)-2,2a-dihydro-7*H*-cyclobuta[4,5]thiazolo[3,2-*a*]pyridine-8a(1*H*)-carboxylic acid (SS1040B). Prepared by following the general procedure. 65 mg of **2h** was converted to 49 mg (77%) of **SS1040B**, isolated as a white solid. IR ($CHCl_3$, cm^{-1}): ν 3583, 3346, 2936, 2883, 1726, 1630, 1487, 1426, 1213, 1093, 1037, 747. ^{1}H NMR (600 MHz, $CDCl_3$) δ 7.80 (d, *J* = 8.0 Hz, 1H), 7.73 (d, *J* = 7.3 Hz, 1H), 7.54 (dd, *J* = 125.6, 8.2 Hz, 4H), 7.38 – 7.32 (m, 2H), 7.14 (s, 1H), 6.45 (s, 1H), 4.76 (d, *J* = 8.0 Hz, 1H), 4.35 (q, *J* = 16.2 Hz, 2H), 3.79 (q, *J* = 9.2 Hz, 1H), 3.31 (dd, *J* = 13.3, 9.5 Hz, 1H), 3.10 (dd, *J* = 13.4, 9.8 Hz, 1H), 1.71 – 1.67 (m, 1H), 1.15 – 1.10 (m, 1H), 1.01 – 0.97 (m, 1H), 0.79 – 0.70 (m, 2H). ^{13}C NMR (151 MHz, $CDCl_3$) δ 168.5, 163.1, 157.8, 151.5, 143.9, 140.5, 139.8, 139.7, 129.9 (q, *J* = 3.7 Hz), 127.0 (2C), 125.9 (q, *J* = 3.8 Hz, 2C), 124.5, 124.2, 123.9 (q, *J* = 272.2 Hz), 123.2, 123.1, 122.2, 117.4, 115.5, 76.1, 49.9, 46.9, 37.2, 34.0, 11.3, 8.3, 7.7. ^{19}F NMR (565 MHz, $CDCl_3$) δ -62.58. HRMS (ESI-TOF) m/z: $[M+H]^+$ Calcd for $C_{29}H_{23}F_3NO_3S_2^+$ 554.1066; observed 554.1035.

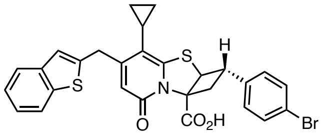

(2*S*)-5-(benzo[*b*]thiophen-2-ylmethyl)-2-(4-bromophenyl)-4-cyclopropyl-7-oxo-2,2a-dihydro-7*H*-cyclobuta[4,5]thiazolo[3,2-*a*]pyridine-8a(1*H*)-carboxylic acid (SS1045B). Prepared by following the general procedure. 68 mg of **2i** was converted to 44 mg (66%) of **SS1045B**, isolated as a white solid. IR ($CHCl_3$, cm^{-1}): ν 3583, 2980, 1728, 1620, 1563, 1485, 1325, 1214, 1165, 1123, 1068, 748. ^{1}H NMR (600 MHz, $(CD_3)_2SO$) δ 7.91 (d, *J* = 7.4 Hz, 1H), 7.80 (d, *J* = 7.0 Hz, 1H), 7.56 (d, *J* = 8.5 Hz, 1H), 7.37 – 7.30 (m, 6H), 6.03 (s, 1H), 4.61 (d, *J* = 7.6 Hz, 1H), 4.33 (q, *J* = 16.1 Hz, 2H), 3.71 (td, *J* = 9.7, 7.5 Hz, 1H), 3.08 (dd, *J* = 12.8, 9.9 Hz, 1H), 2.82 (dd, *J* = 12.1, 9.8 Hz, 1H), 1.76 – 1.62 – 1.57 (m, 1H), 0.99 – 0.92 (m, 2H), 0.75 – 0.63 (m, 2H). ^{13}C NMR (151 MHz, $(CD_3)_2SO$) δ 169.3, 160.1, 156.1, 151.0, 142.6, 141.0, 140.1, 139.5, 131.8 (2C), 129.5 (2C), 124.8, 124.4, 123.6, 123.4, 122.8, 120.5, 114.9,

112.8, 73.7, 51.3, 46.7, 35.7, 33.7, 11.2, 8.3, 8.1. HRMS (ESI-TOF) m/z: $[M+H]^+$ Calcd for $C_{28}H_{23}BrNO_3S_2^+$ 564.0297; observed 564.0282.

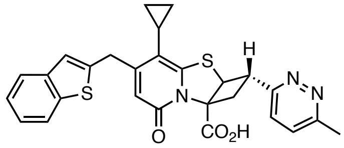

(2*S*)-5-(benzo[*b*]thiophen-2-ylmethyl)-4-cyclopropyl-2-(6-methylpyridazin-3-yl)-7-oxo-2,2a-dihydro-7*H*-cyclobuta[4,5]thiazolo[3,2-*a*]pyridine-8a(1*H*)-carboxylic acid (SS1022B). Prepared by following the general procedure. 35 mg of **2j** was converted to 14 mg (41%) of **SS1022B**, isolated as a white solid. IR ($CHCl_3$, cm^{-1}): ν 3004, 1723, 1650, 1485, 1428, 1293, 1213, 1166, 1115, 1032, 749. ^1H NMR (600 MHz, $CDCl_3$) δ 7.78 (d, *J* = 7.9 Hz, 1H), 7.71 (d, *J* = 7.4 Hz, 1H), 7.36 – 7.29 (m, 4H), 7.11 (s, 1H), 6.40 (s, 1H), 5.09 (d, *J* = 7.5 Hz, 1H), 4.32 (q, *J* = 16.1 Hz, 2H), 4.01 (td, *J* = 9.5, 7.6 Hz, 1H), 3.42 (dd, *J* = 13.2, 9.6 Hz, 1H), 3.26 (dd, *J* = 13.2, 9.6 Hz, 1H), 2.75 (s, 3H), 1.65 – 1.61 (m, 1H), 1.07 – 1.03 (m, 1H), 0.97 – 0.92 (m, 1H), 0.78 – 0.69 (m, 2H). ^{13}C NMR (151 MHz, $CDCl_3$) δ 168.6, 162.3, 159.2, 159.0, 157.0, 151.4, 141.0, 139.8, 139.7, 127.8, 125.6, 124.3, 124.0, 123.1, 122.9, 122.1, 116.0, 115.6, 75.6, 48.6, 46.9, 35.9, 34.0, 21.8, 11.3, 8.2, 7.7. HRMS (ESI-TOF) m/z: $[M+H]^+$ Calcd for $C_{27}H_{24}N_3O_3S_2^+$ 502.1254; observed 502.1230.

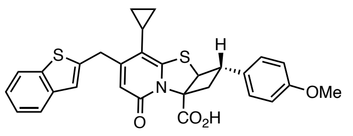

(8a*R*)-5-(benzo[*b*]thiophen-2-ylmethyl)-4-cyclopropyl-2-(4-methoxyphenyl)-7-oxo-2,2a-dihydro-7*H*-cyclobuta[4,5]thiazolo[3,2-*a*]pyridine-8a(1*H*)-carboxylic acid (PS2840). Prepared by following the general procedure. 60 mg of **2k** was converted to 30 mg (51%) of **PS2840**, isolated as a white solid. IR ($CHCl_3$, cm^{-1}): ν 3005, 2933, 1727, 1625, 1545, 1514, 1485, 1425, 1213, 1032, 749. ^1H NMR (400 MHz, $(CD_3)_2SO$) δ 7.91 (dd, *J* = 7.9, 1.2 Hz, 1H), 7.82 –n 7.77 (dd, *J* = 7.9, 1.2 Hz, 1H), 7.41 – 7.22 (m, 5H), 6.98 – 6.86 (m, 2H), 6.02 (s, 1H), 4.51 (d, *J* = 7.5 Hz, 1H), 4.33 (q, *J* = 16.4 Hz, 2H), 3.74 (s, 3H), 3.66 (dd, *J* = 9.6, 7.5 Hz, 1H), 3.07 (dd, *J* = 12.7, 9.9 Hz, 1H), 2.78 (dd, *J* = 12.7, 9.3 Hz, 1H), 1.62–1.55 (m, 1H), 1.01 – 0.88 (m, 2H), 0.77 – 0.69 (m, 1H), 0.68 – 0.60 (m, 1H). ^{13}C NMR (100 MHz, $(CD_3)_2SO$) δ 168.9, 159.7, 158.3, 155.6, 150.7, 142.1, 139.6, 139.0, 133.1, 127.8, 124.4, 123.9, 123.1, 122.9, 114.3, 113.9, 112.3, 73.2, 55.1, 51.4, 46.4, 35.7, 33.2, 10.8, 7.8, 7.7. HRMS (ESI-TOF) m/z: $[M+H]^+$ Calcd for $C_{29}H_{26}NO_4S_2^+$ 516,1298; observed 516.1235.

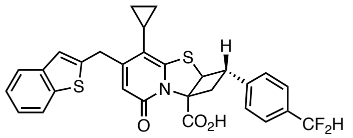

(2*S*)-5-(benzo[*b*]thiophen-2-ylmethyl)-4-cyclopropyl-2-(4-(difluoromethyl)phenyl)-7-oxo-2,2a-dihydro-7*H*-cyclobuta[4,5]thiazolo[3,2-*a*]pyridine-8a(1*H*)-carboxylic acid (SS1238B). Prepared by following the general procedure. 58 mg of **2l** was converted to 24 mg (42%) of **SS1238B**, isolated as a white solid. IR ($CHCl_3$, cm^{-1}): ν 2925, 1721, 1655, 1579, 1544, 1536, 1486, 1296, 1219, 1071, 749. ^1H NMR (600 MHz, $CDCl_3$) δ 7.80 (d, *J* = 7.9 Hz, 1H), 7.73 (d, *J* = 7.8 Hz, 1H), 7.53 (d, *J* = 7.8 Hz, 2H), 7.41 (d, *J* = 7.9 Hz, 2H), 7.35 (dtd, *J* = 22.7, 7.2, 1.2 Hz, 2H), 7.13 (s, 1H), 6.66 (t, *J* = 56.4 Hz, 1H), 6.44 (s, 1H), 4.77 (d, *J* = 8.0 Hz, 1H), 4.35 (q, *J* = 16.2 Hz, 2H), 3.76 (q, *J* = 9.1 Hz, 1H), 3.31 (dd, *J* = 13.4, 9.5 Hz, 1H), 3.09 (dd, *J* = 13.4, 9.8 Hz, 1H) 1.71 – 1.66 (m, 1H), 1.14 – 1.10 (m, 1H), 1.01 – 0.96 (m, 1H), 0.79 – 0.71 (m, 2H). ^{13}C NMR (151 MHz, $CDCl_3$) δ 168.6, 163.1, 157.8, 151.6, 142.7, 140.5,

139.8, 139.7, 133.8 (t, J = 22.2 Hz, 1C), 127.0 (2C), 126.2 (t, J = 6.0 Hz, 2C), 124.5, 124.2, 123.2, 123.1, 122.2, 117.5, 115.4, 114.3 (t, J = 238.7 Hz), 76.2, 49.9, 46.9, 37.4, 34.0, 11.3, 8.3, 7.7. ^{19}F NMR (376 MHz, DMSO) δ -108.98 (2F). HRMS (ESI-TOF) m/z: $[M+H]^+$ Calcd for $C_{29}H_{24}F_2NO_3S_2^+$ 536.1160; observed 536.1135.

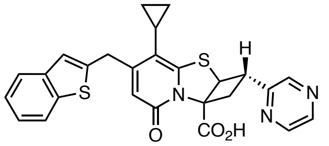

(2*S*)-5-(benzo[*b*]thiophen-2-ylmethyl)-4-cyclopropyl-7-oxo-2-(pyrazin-2-yl)-2,2a-dihydro-7*H*-cyclobuta[4,5]thiazolo[3,2-*a*]pyridine-8a(1*H*)-carboxylic acid (SS998). Prepared by following the general procedure. 24 mg of **2m** was converted to 13 mg (53%) of **SS998**, isolated as a white solid. IR ($CHCl_3$, cm^{-1}): ν 3054, 3004, 1725, 1652, 1575, 1485, 1425, 1233, 1214, 1142, 1031, 749. ^1H NMR (600 MHz, $CDCl_3$) δ 8.65 (s, 1H), 8.52 (d, J = 2.5 Hz, 1H), 8.49 (d, J = 1.5 Hz, 1H), 7.80 (d, J = 7.9 Hz, 1H), 7.73 (d, J = 7.9 Hz, 1H), 7.38 – 7.31 (m, 2H), 7.13 (s, 1H), 6.43 (s, 1H), 5.06 (d, J = 7.6 Hz, 1H), 4.35 (q, J = 16.2 Hz, 2H), 3.85 (q, J = 8.8 Hz, 1H), 3.33 – 3.23 (m, 2H), 1.70 – 1.64 (m, 1H), 1.13 – 1.09 (m, 1H), 1.00 – 0.93 (m, 1H), 0.79 – 0.68 (m, 2H). ^{13}C NMR (151 MHz, $CDCl_3$) δ 168.3, 162.9, 157.6, 154.1, 151.4, 144.8, 143.7, 143.5, 140.6, 139.8, 139.7, 124.4, 124.2, 123.2, 123.1, 122.2, 117.1, 115.6, 76.2, 48.1, 45.9, 35.7, 34.0, 11.3, 8.3, 7.6. HRMS (ESI-TOF) m/z: $[M+H]^+$ Calcd for $C_{26}H_{22}N_3O_3S_2^+$ 488.1097; observed 488.1064.

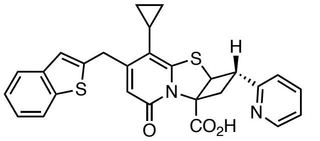

(2*S*)-5-(benzo[*b*]thiophen-2-ylmethyl)-4-cyclopropyl-7-oxo-2-(pyridin-2-yl)-2,2a-dihydro-7*H*-cyclobuta[4,5]thiazolo[3,2-*a*]pyridine-8a(1*H*)-carboxylic acid (SS991B). Prepared by following the general procedure. 57 mg of **2n** was converted to 17 mg (31%) of **SS991B**, isolated as a white solid. IR ($CHCl_3$, cm^{-1}): ν 3058, 3006, 1722, 1649, 1590, 1484, 1433, 1294, 1213, 1166, 1115, 749. ^1H NMR (600 MHz, $(CD_3)_2SO$) δ 8.63 – 8.61 (m, 1H), 7.92 (dd, J = 7.9, 1.1 Hz, 1H), 7.80 (dd, J = 8.0, 1.1 Hz, 1H), 7.75 (td, J = 7.6, 1.8 Hz, 1H), 7.38 – 7.29 (m, 5H), 6.03 (s, 1H), 4.70 (d, J = 7.4 Hz, 1H), 4.34 (q, J = 16.1 Hz, 2H), 3.82 (td, J = 9.6, 7.4 Hz, 1H), 3.29 (dd, J = 12.5, 9.8 Hz, 1H), 2.75 (dd, J = 13.5, 9.4 Hz, 1H), 1.62 – 1.58 (m, 1H), 1.00 – 0.91 (m, 2H), 0.76 – 0.72 (m, 1H), 0.68 – 0.63 (m, 1H). ^{13}C NMR (151 MHz, $(CD_3)_2SO$) δ 169.2, 160.2, 159.5, 156.1, 151.2, 149.9, 142.6, 140.1, 139.5, 137.2, 124.8, 124.4, 123.6, 123.4, 122.8 (2C), 114.9, 112.9, 74.1, 49.9, 48.6, 34.4, 33.7, 11.3, 8.3, 8.1. HRMS (ESI-TOF) m/z: $[M+H]^+$ Calcd for $C_{26}H_{22}N_3O_3S_2^+$ 487.1145; observed 487.1115.

Determination of diastereomer's configuration by NMR spectroscopy

The NMR studies were performed on compound IL262 and IL263 which represent a diastereomeric pair. The 3D structures for both diastereomers are shown in **Figure S15**. The configuration on the stereocenters was established by careful analysis of NOE interaction between the H^1, H^2, H^{3a} and H^{3b} protons. As shown in NOESY spectrum of compound IL262, strong cross-peaks H^2–H^{3b} and two cross peaks of medium strength H^1–H^2 and H^1–H^{3a} confirmed *trans* configuration between H^1 and H^2. This configuration was further supported by strong cross-peaks H^1–H-aromatic and H^{3a}–H-aromatic. On the other hand, for other diastereomer IL263, two strong

cross peak H[1]–H[2] and H[2]–H[3a] revealed cis configuration between H[1] and H[2]. A cross peak of medium strength H[2]–H[3b] established *trans* configuration between H[2] and H[3b]. Further, a strong cross peak H[3b]–H-aromatic confirmed a cis configuration between H[3b] and the phenyl ring.

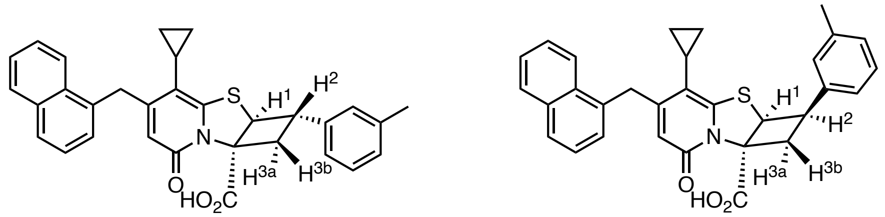

IL262 **IL263**

Fig. S31. Structures of IL262 and IL263 which represent a pair of diastereomers

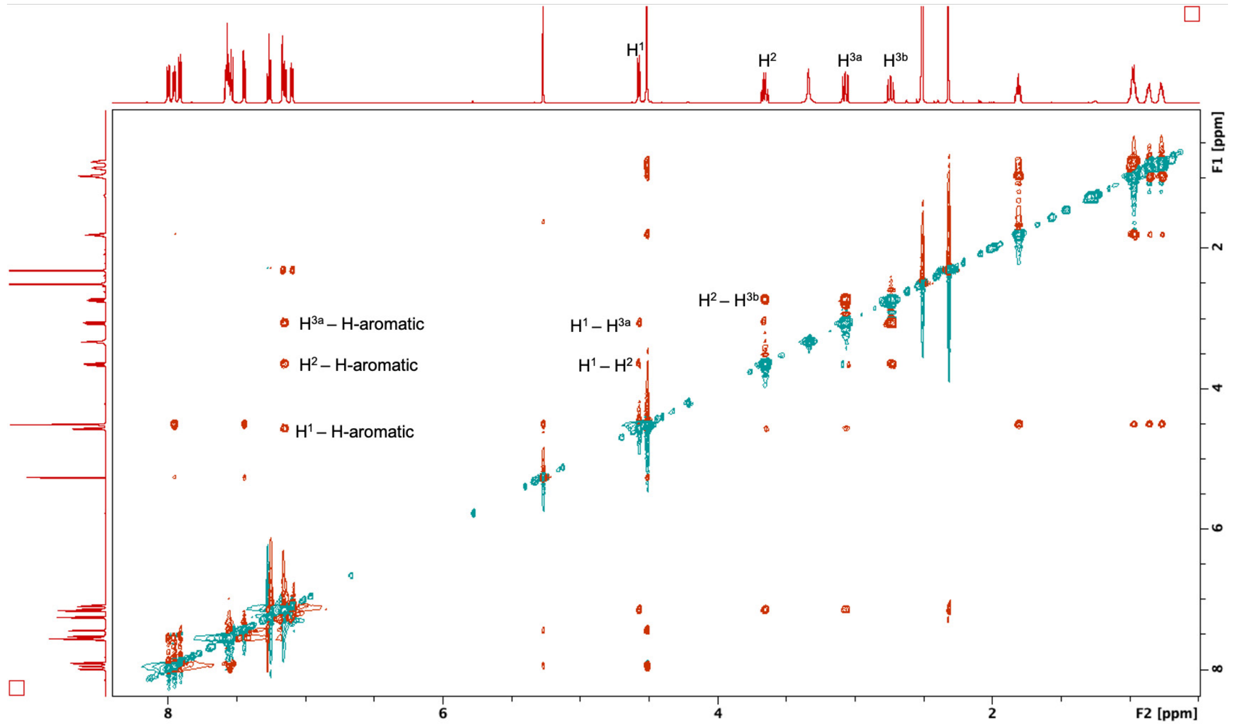

Fig. S32. NOESY spectrum of IL262 in DMSO-d6 at 298 K

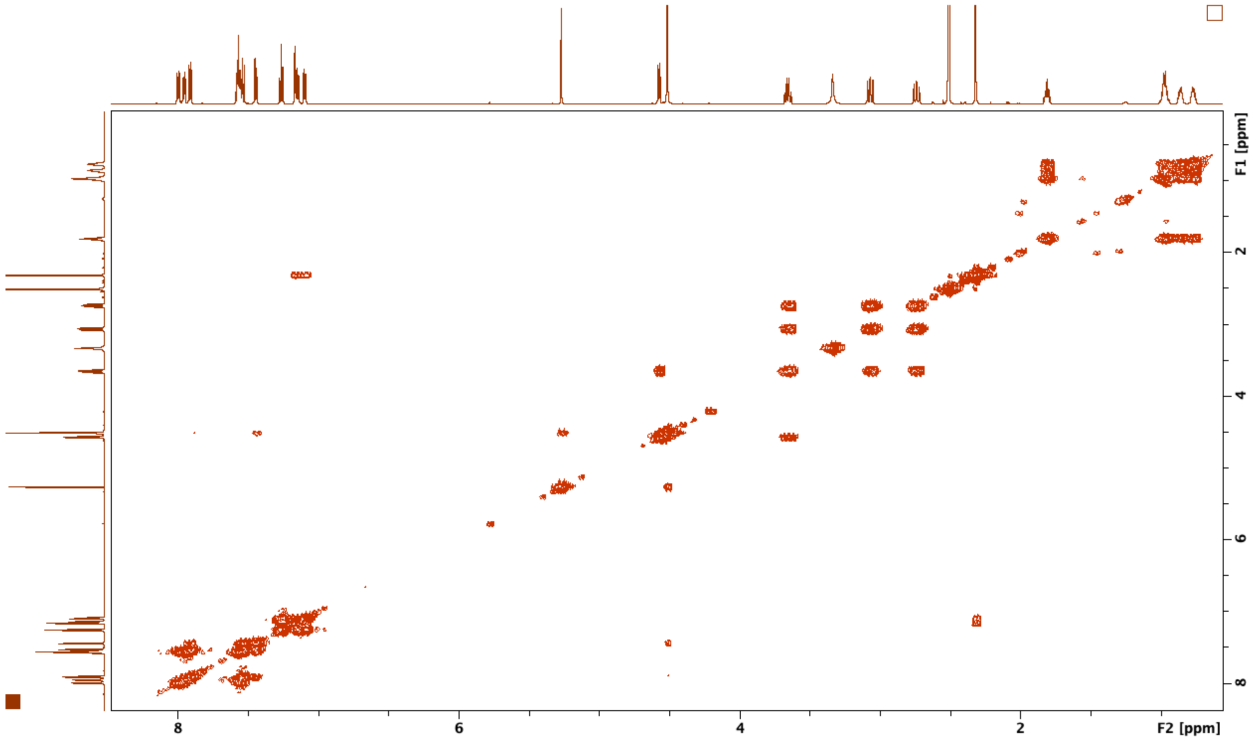

Fig. S33. COSY spectrum of IL262 in DMSO-d6 at 298 K

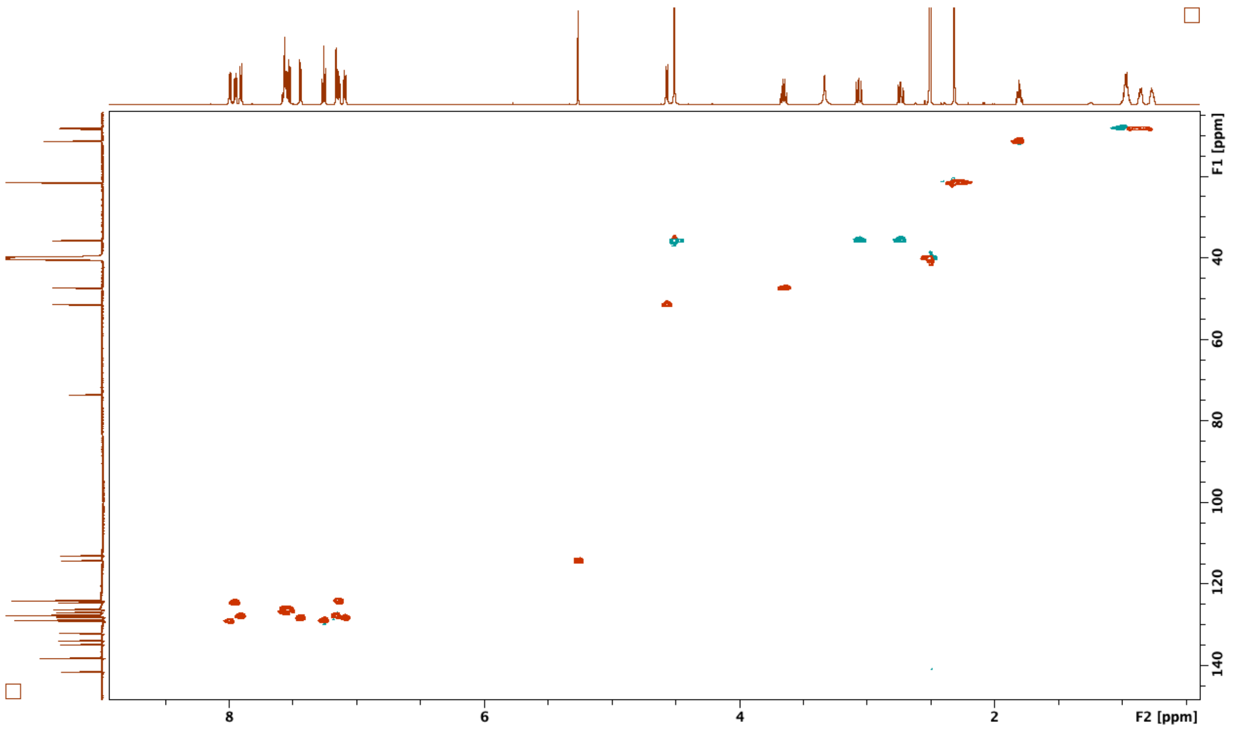

Fig. S34. HSQC spectrum of IL262 in DMSO-d6 at 298 K

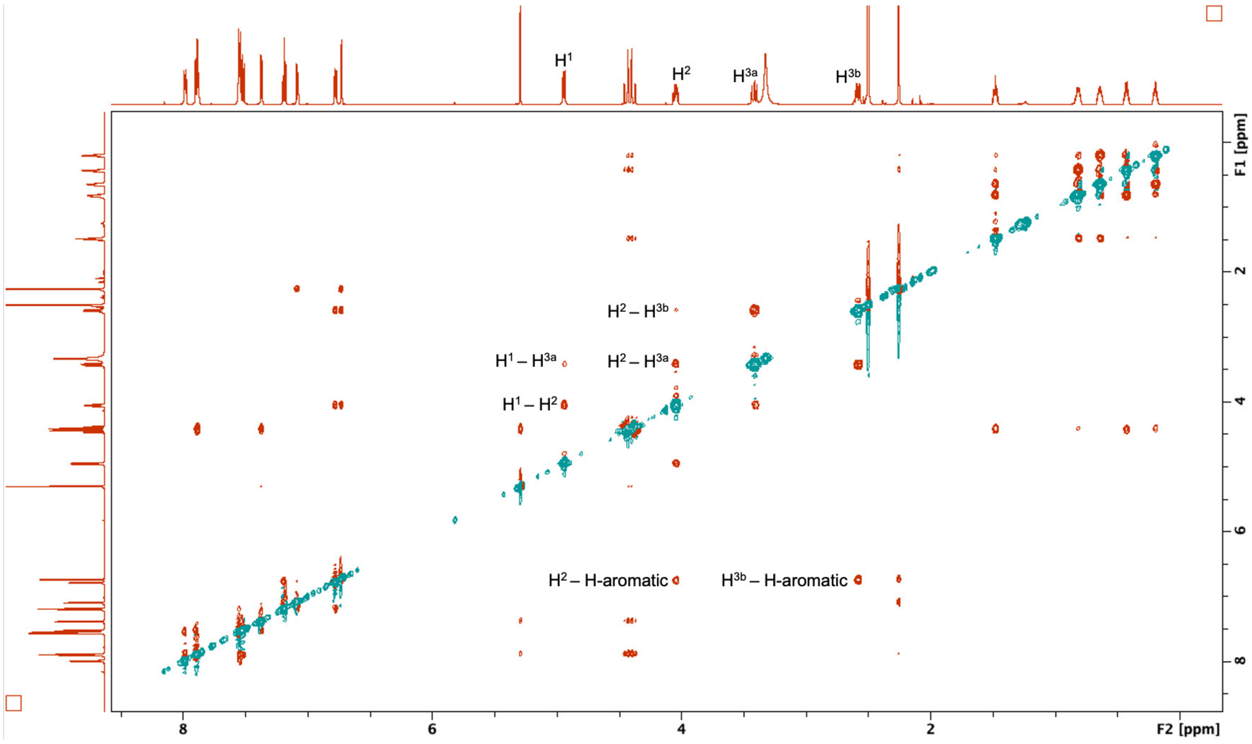

Fig. S35. NOESY spectrum of IL263 in DMSO-d6 at 298 K

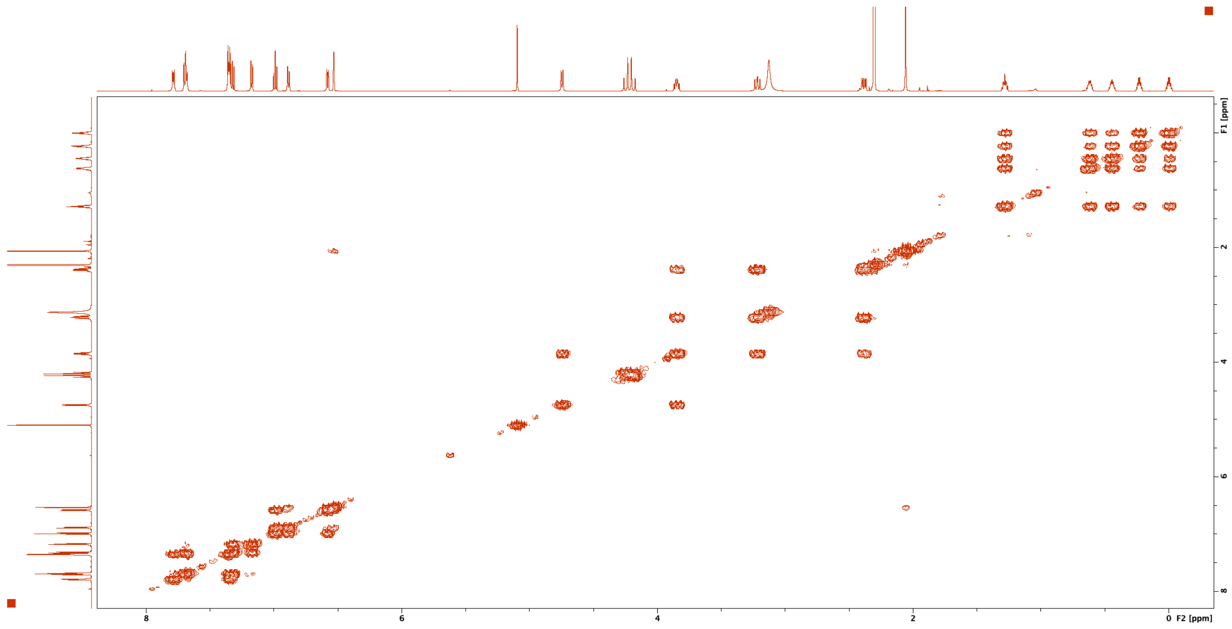

Fig. S36. COSY spectrum of IL263 in DMSO-d6 at 298 K

Compound **1d**, ^{1}H-NMR (600 MHz) and ^{13}C-NMR (151 MHz) ($CDCl_3$):

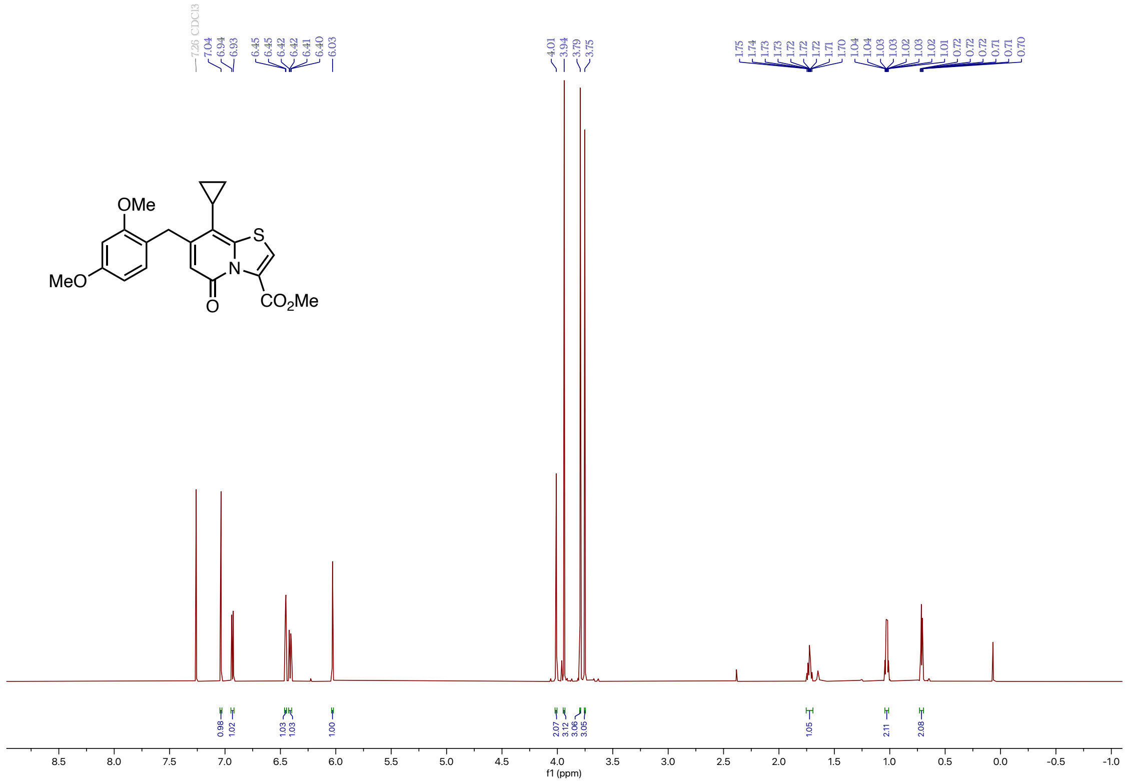

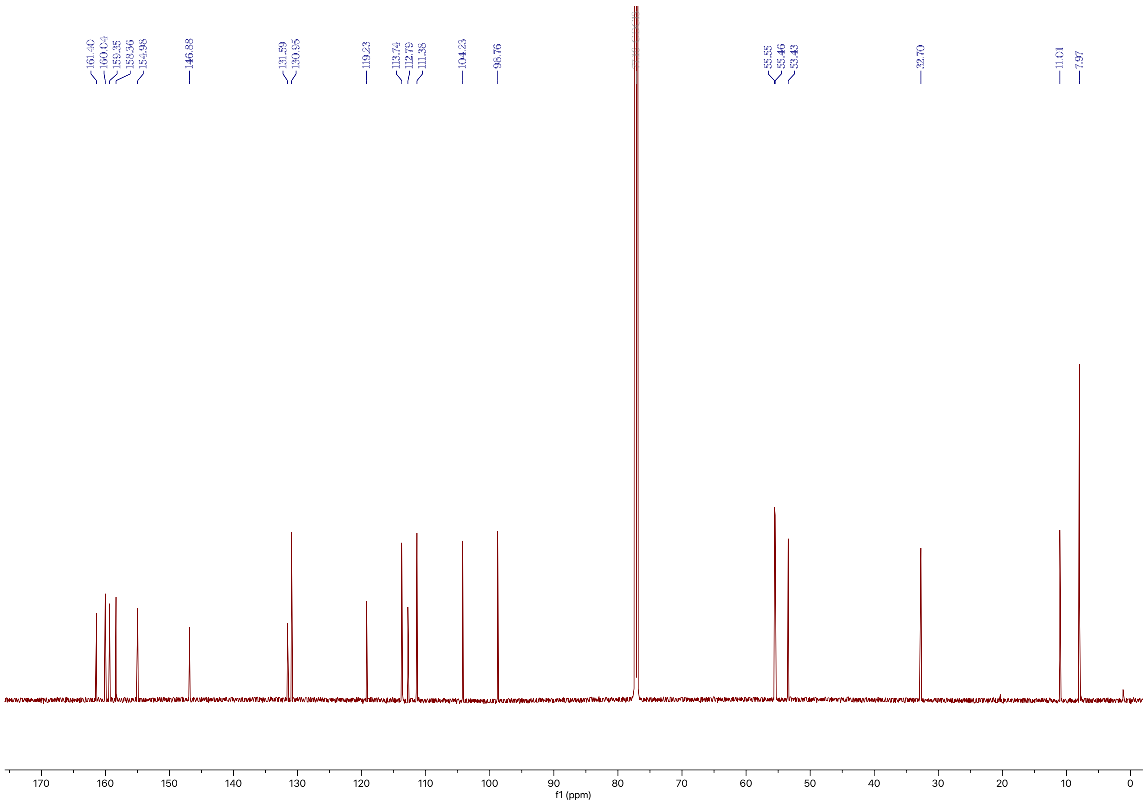

Compound **1e**, ^{1}H-NMR (600 MHz) and ^{13}C-NMR (151 MHz) ($CDCl_3$):

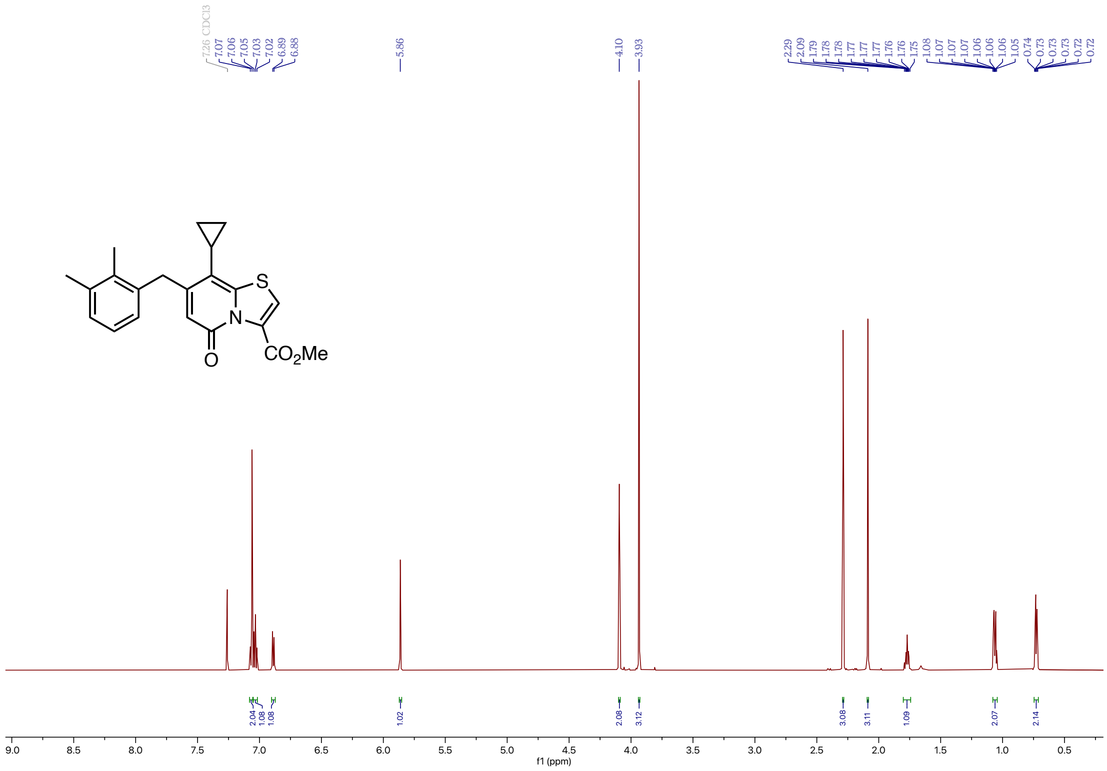

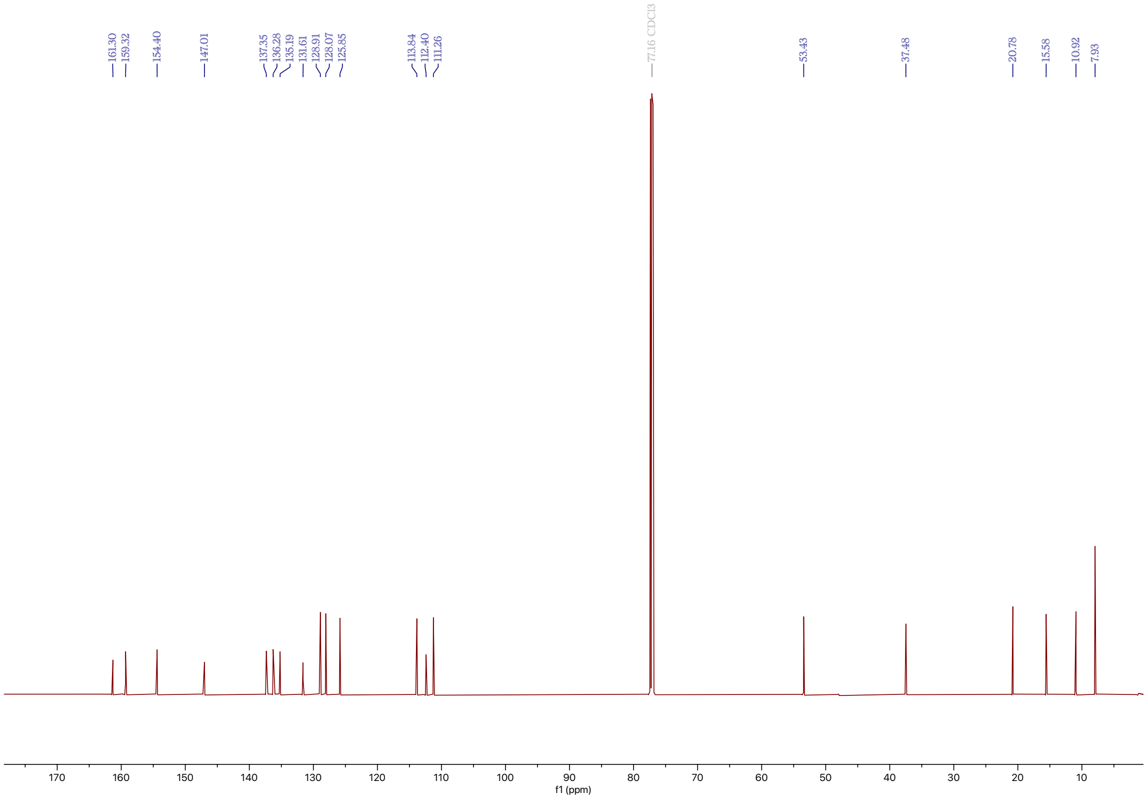

Compound **1f**, ^{1}H-NMR (600 MHz) and ^{13}C-NMR (151 MHz) ($CDCl_3$):

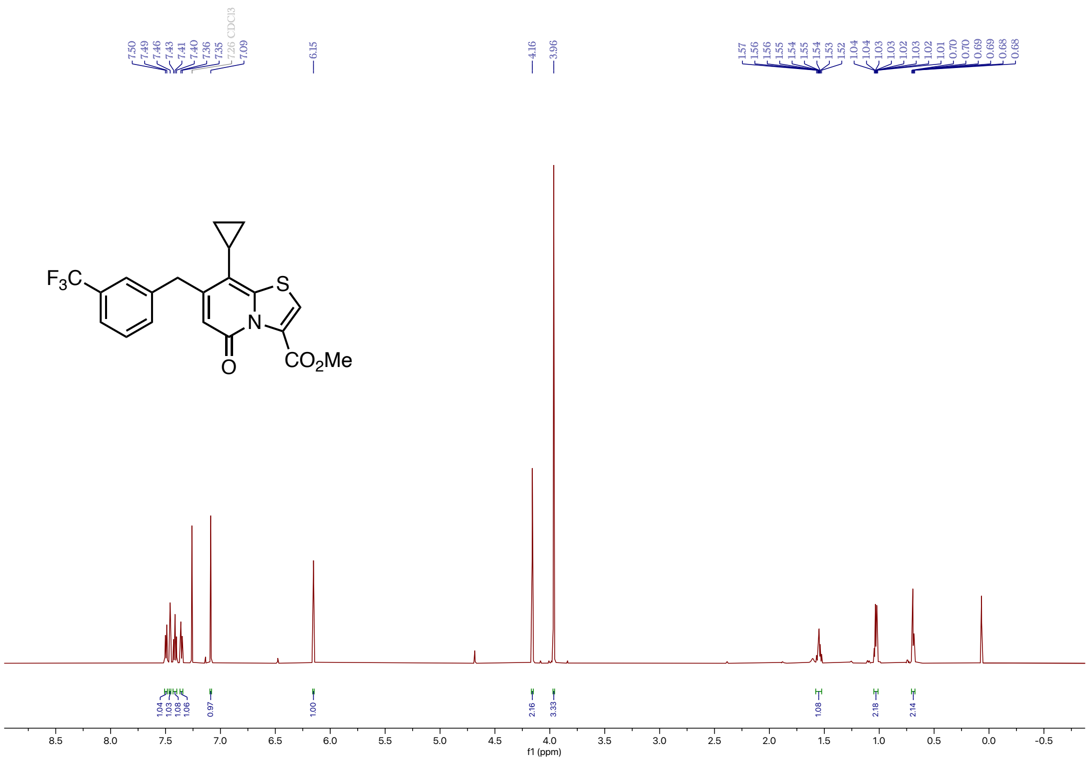

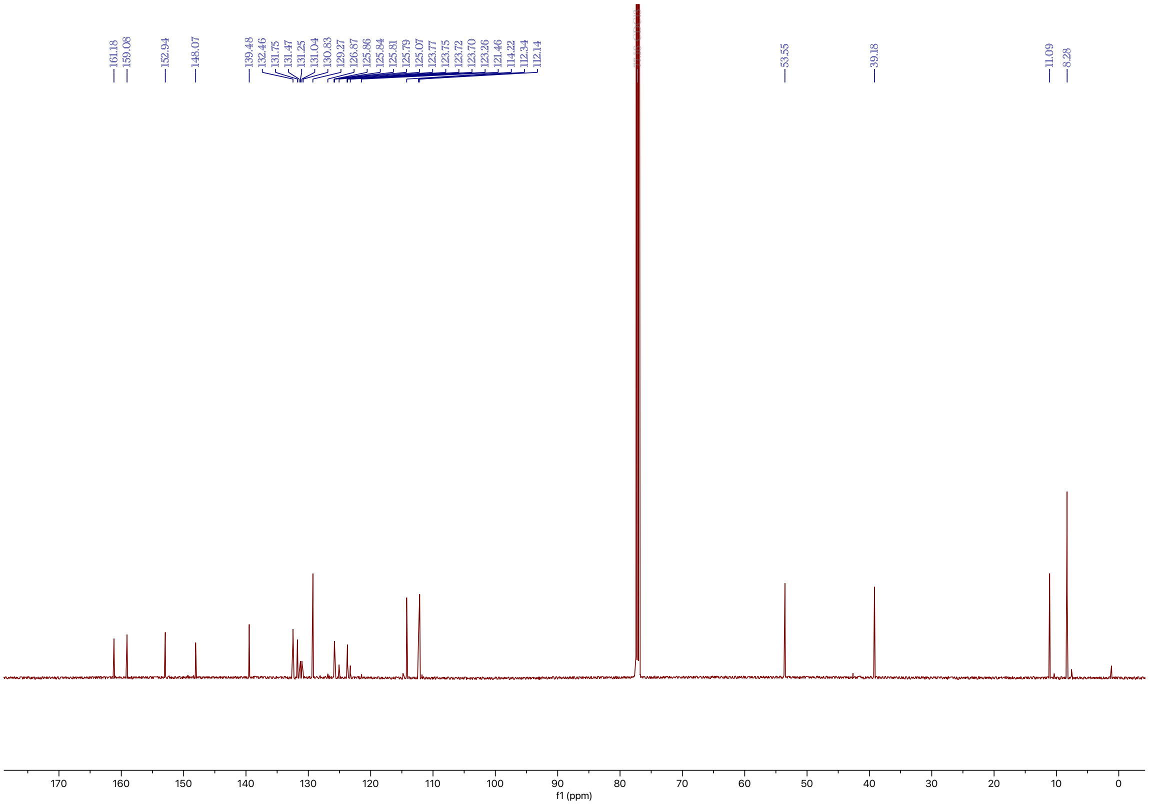

Compound **1f**, ^{19}F NMR (565 MHz) ($CDCl_3$):

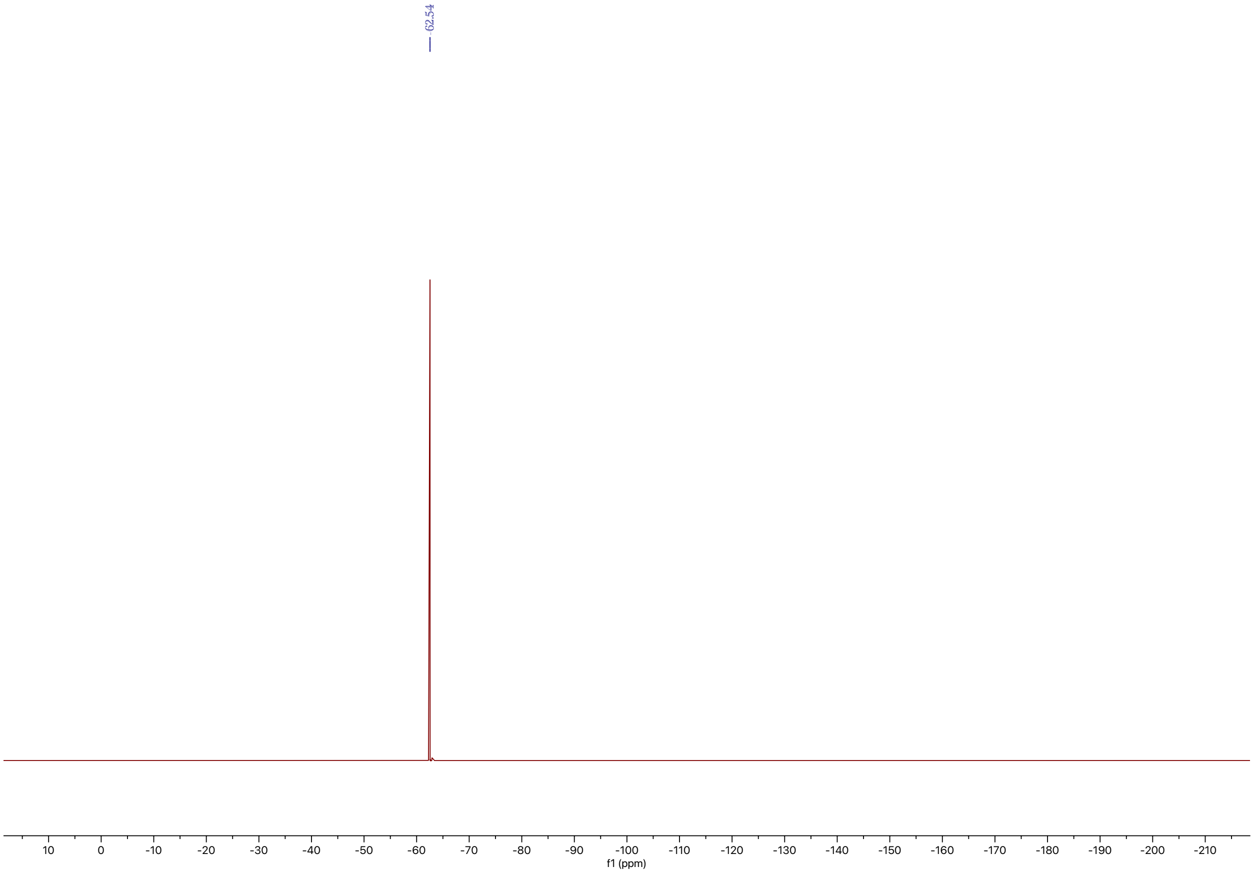

Compound **1g**, ^{1}H-NMR (400 MHz) and ^{13}C-NMR (100 MHz) ($CDCl_3$):

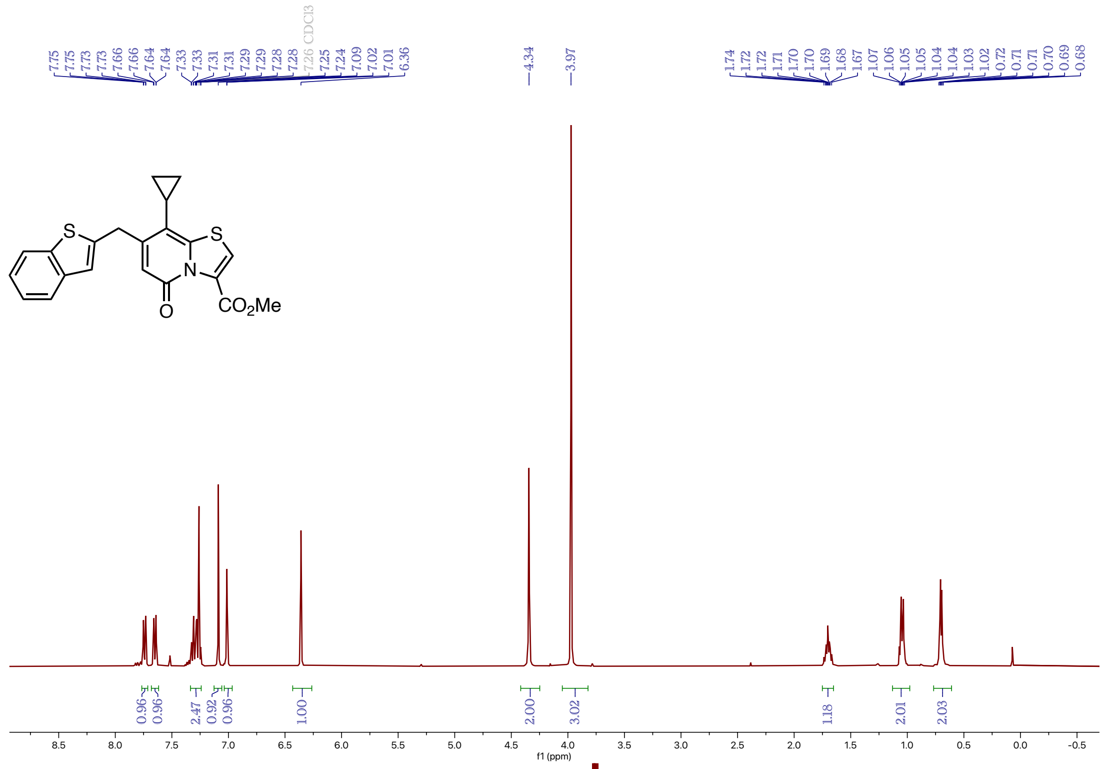

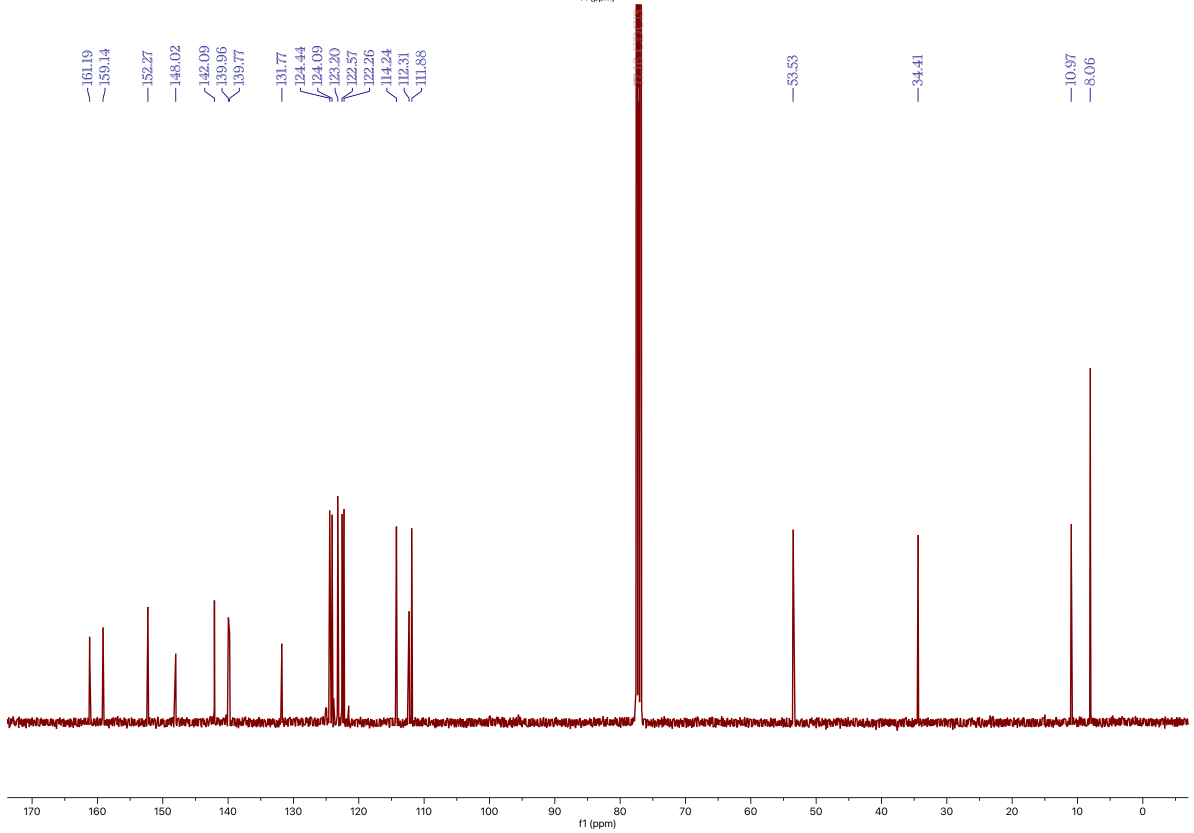

Compound **2a**, ^{1}H-NMR (600 MHz) and ^{13}C-NMR (151 MHz) ($CDCl_3$):

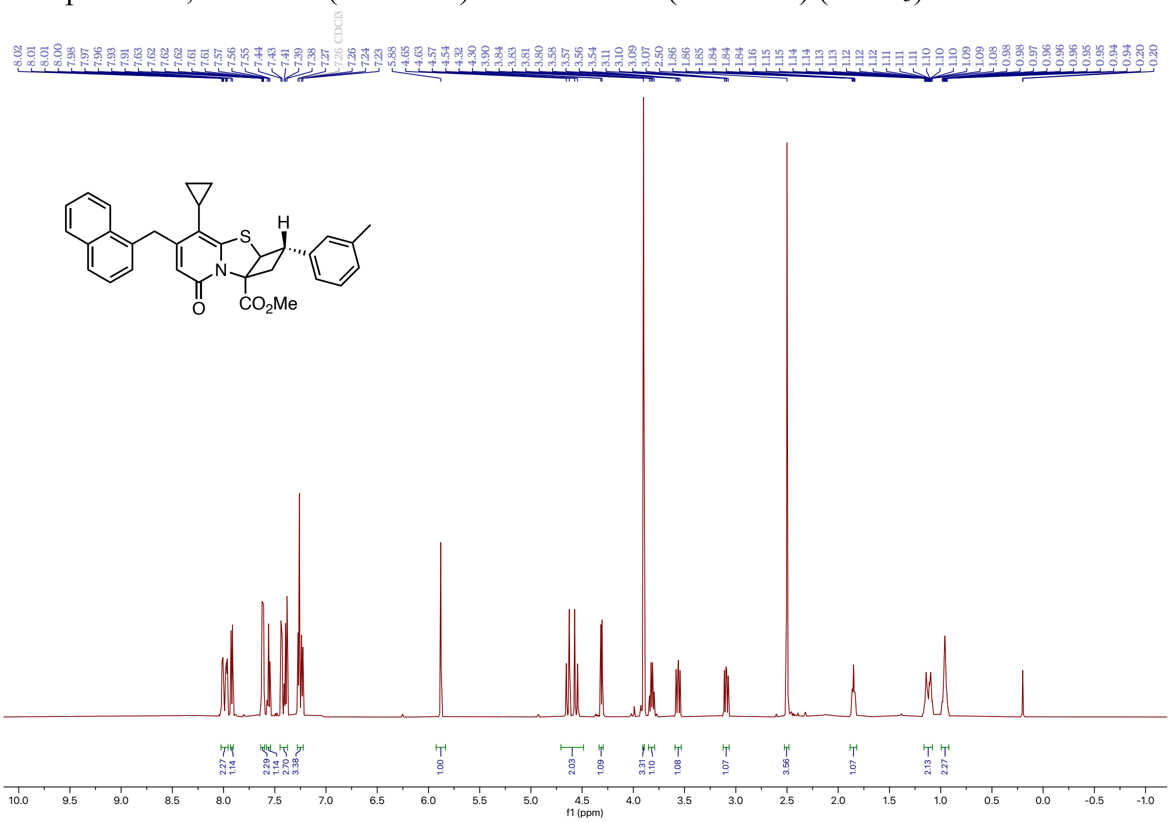

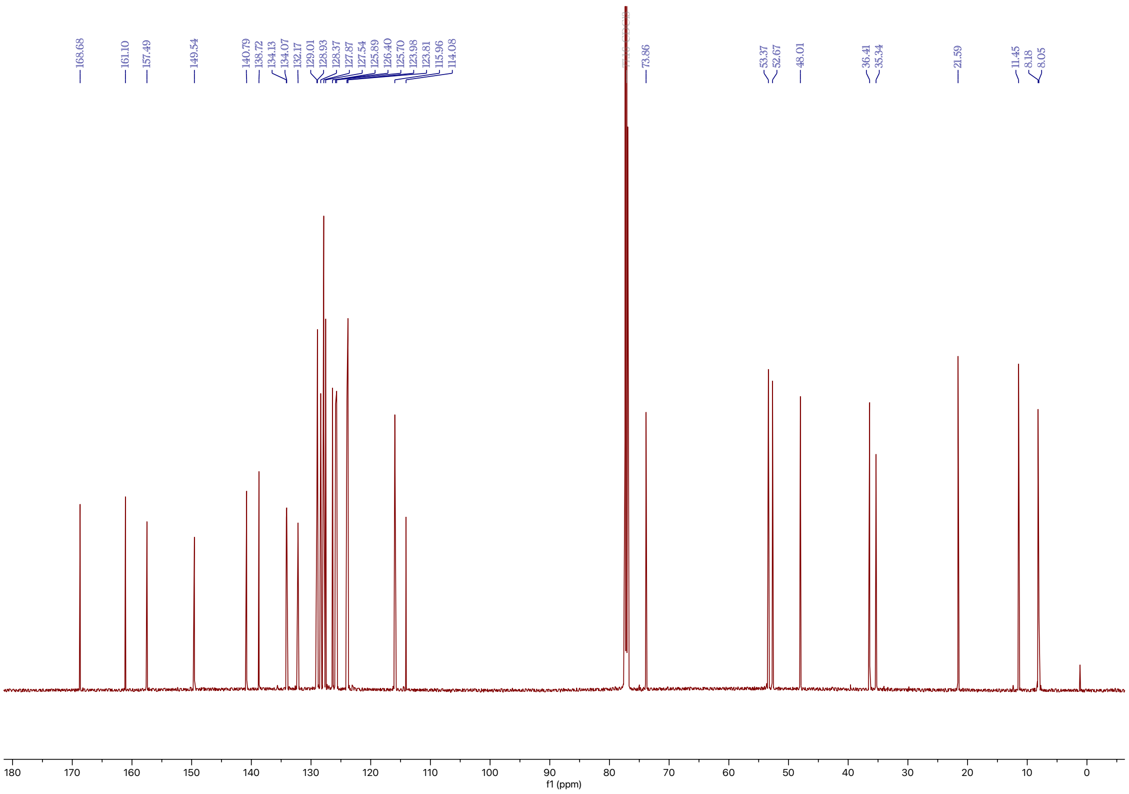

Compound **3a**, ^{1}H-NMR (600 MHz) and ^{13}C-NMR (151 MHz) ($CDCl_3$):

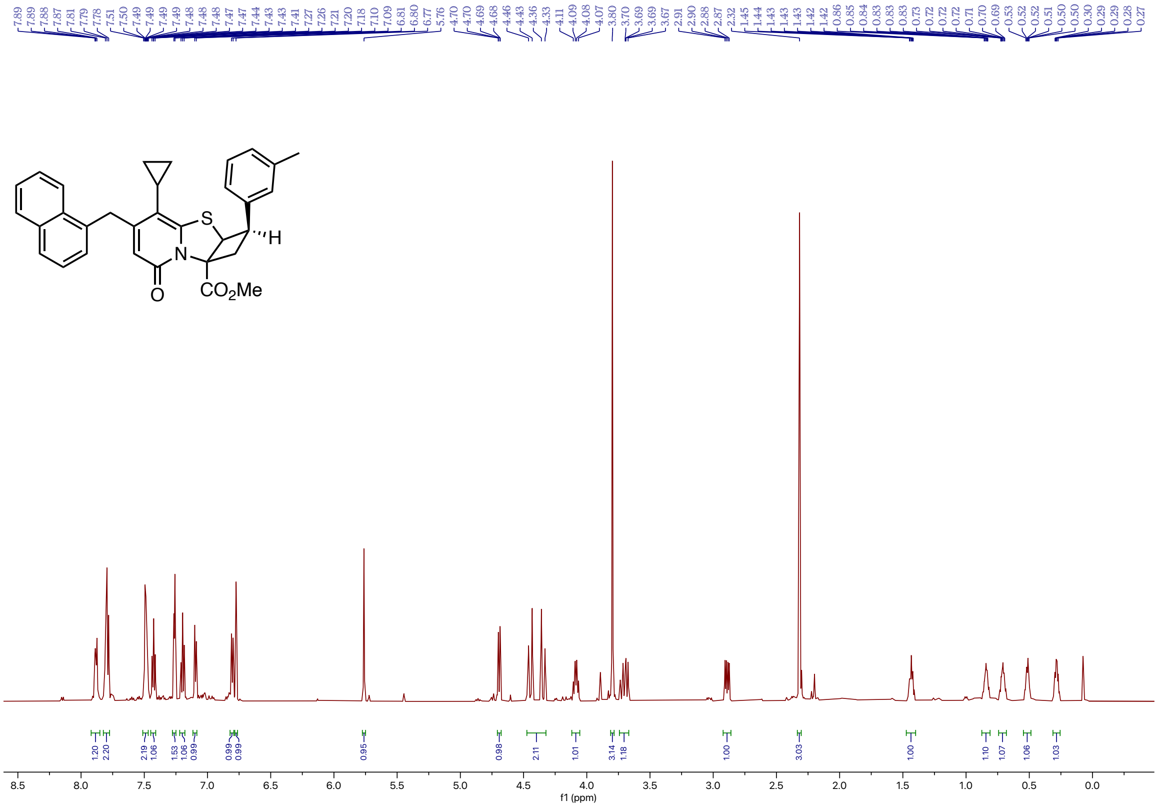

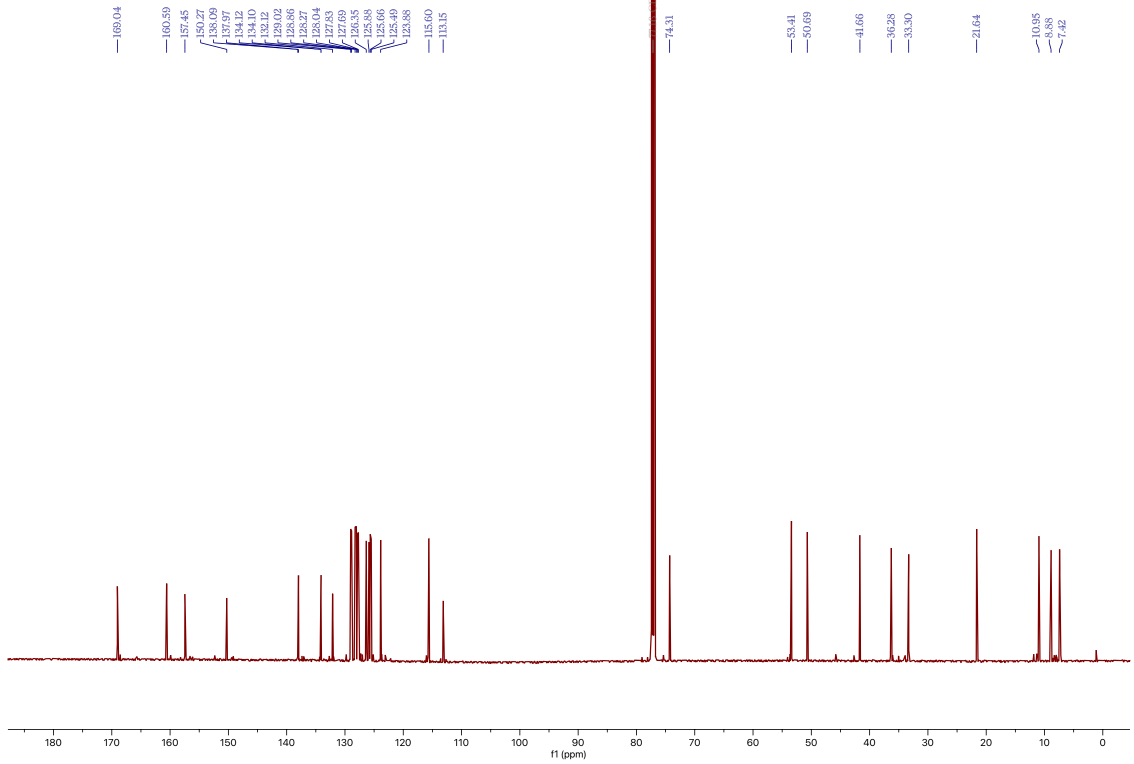

Compound **2b**, ^{1}H-NMR (400 MHz) and ^{13}C-NMR (100 MHz) ($CDCl_3$):

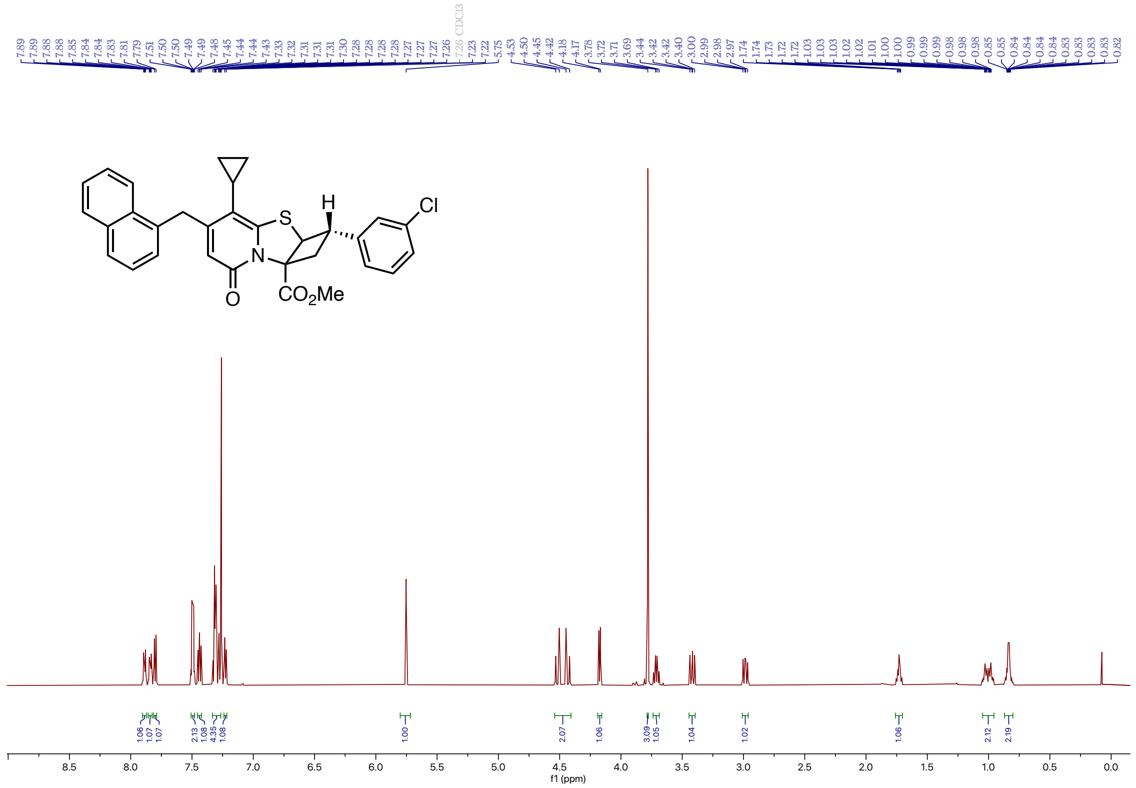

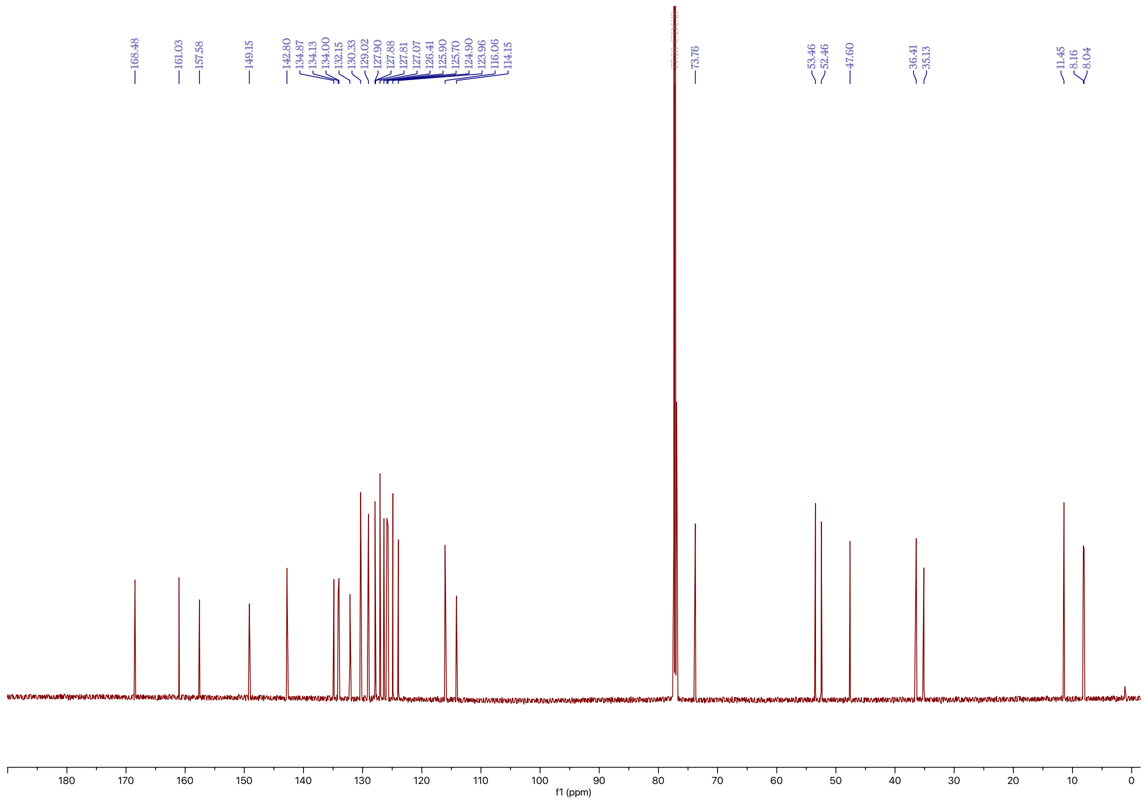

Compound **3b**, ^{1}H-NMR (600 MHz) and ^{13}C-NMR (151 MHz) ($CDCl_3$):

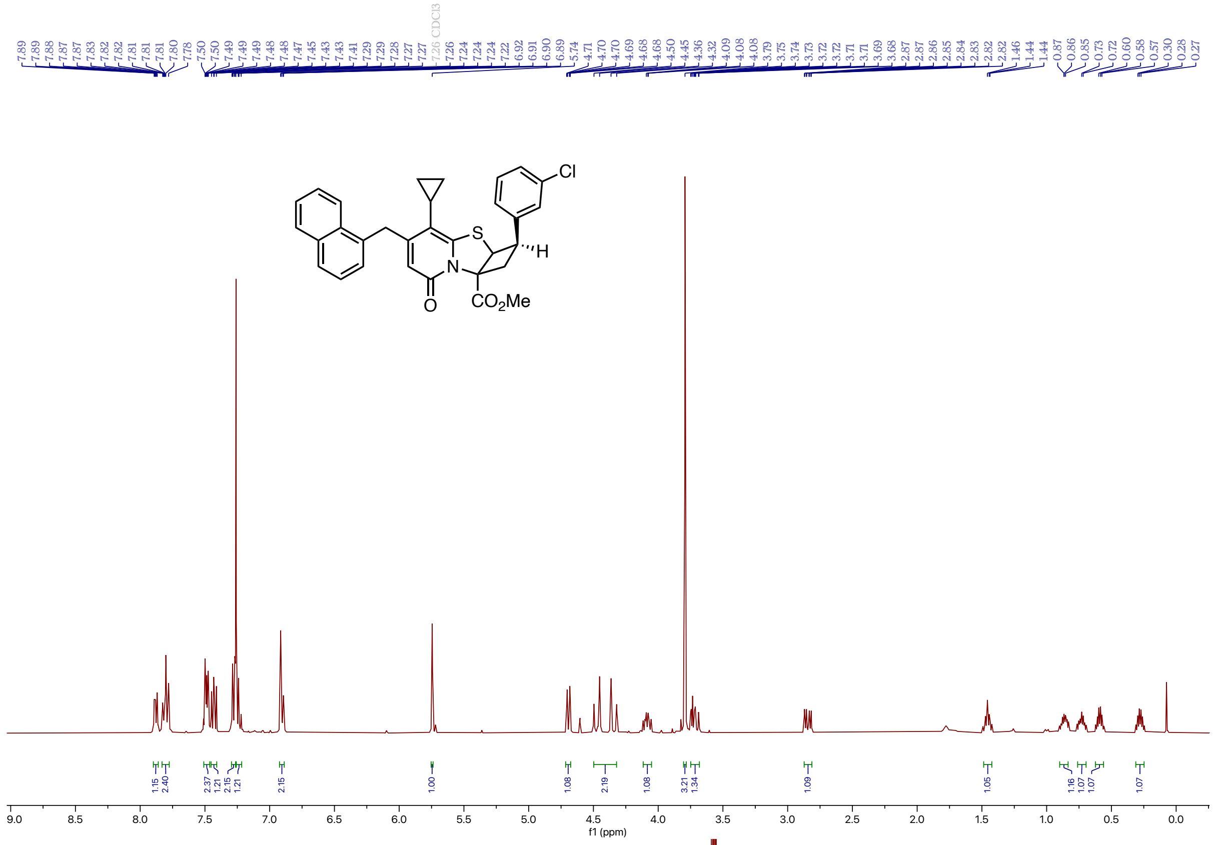

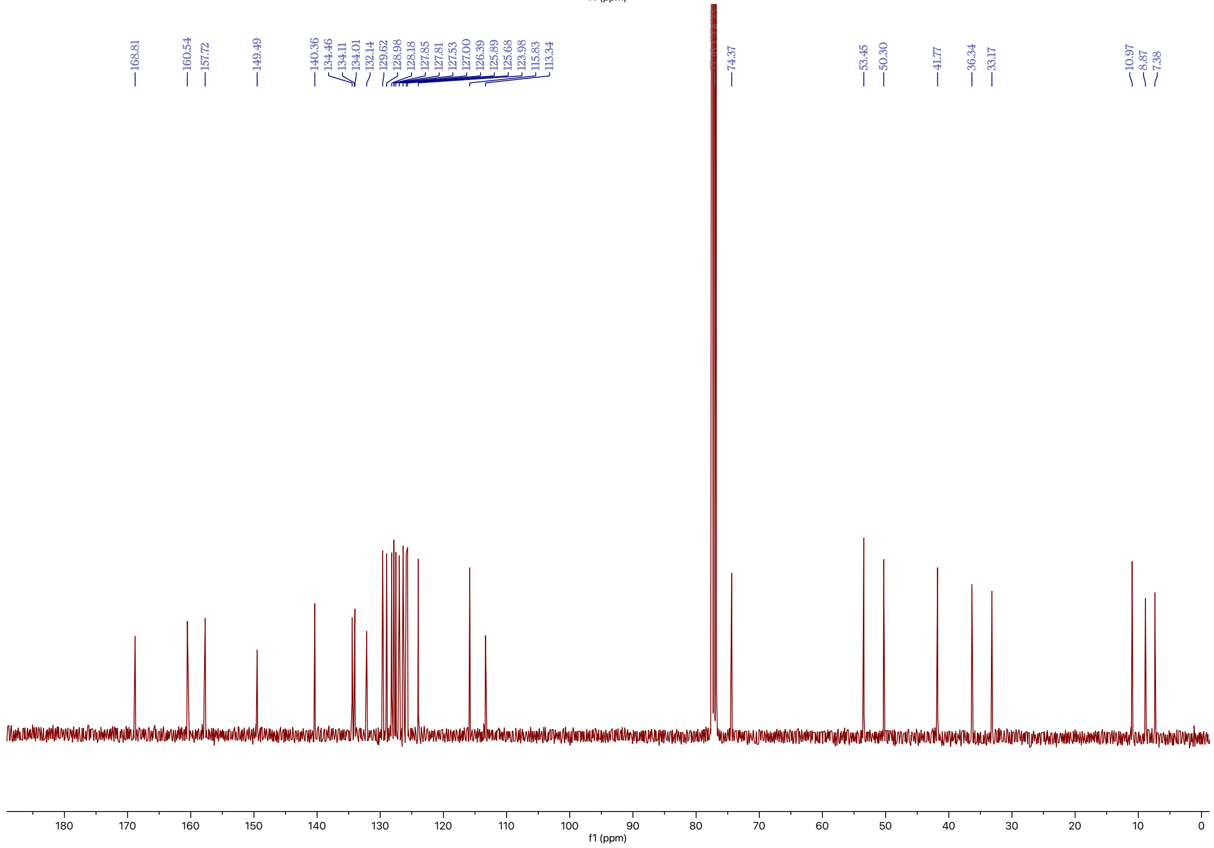

Compound **2c**, ^{1}H-NMR (400 MHz) and ^{13}C-NMR (100 MHz) ($CDCl_3$):

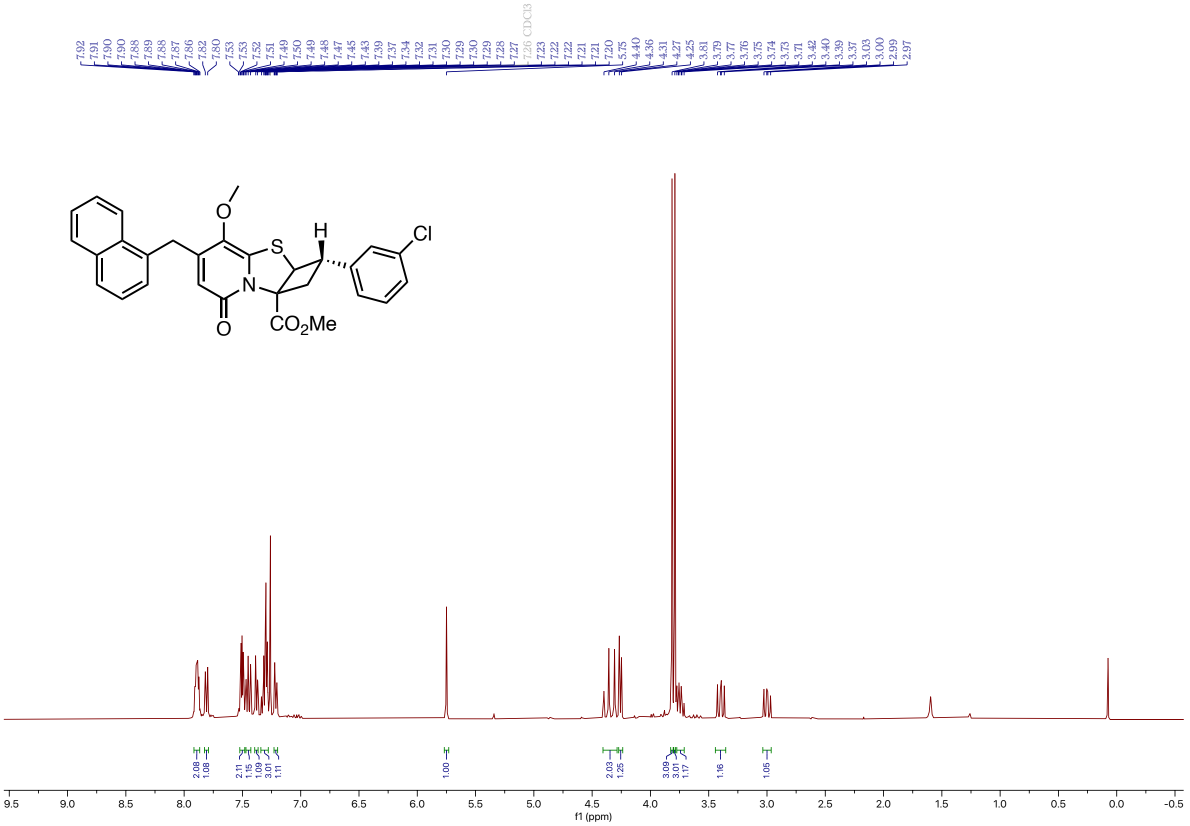

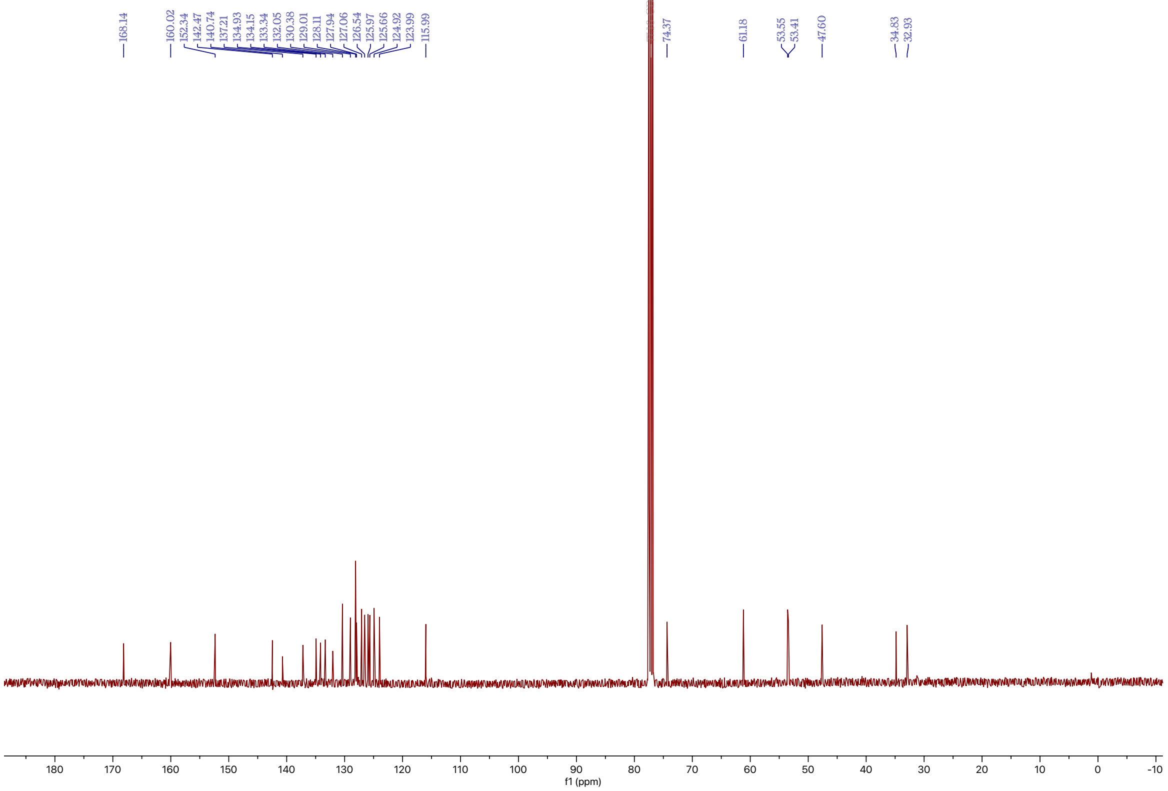

Compound **2d**, ^{1}H-NMR (600 MHz) and ^{13}C-NMR (151 MHz) ($CDCl_3$):

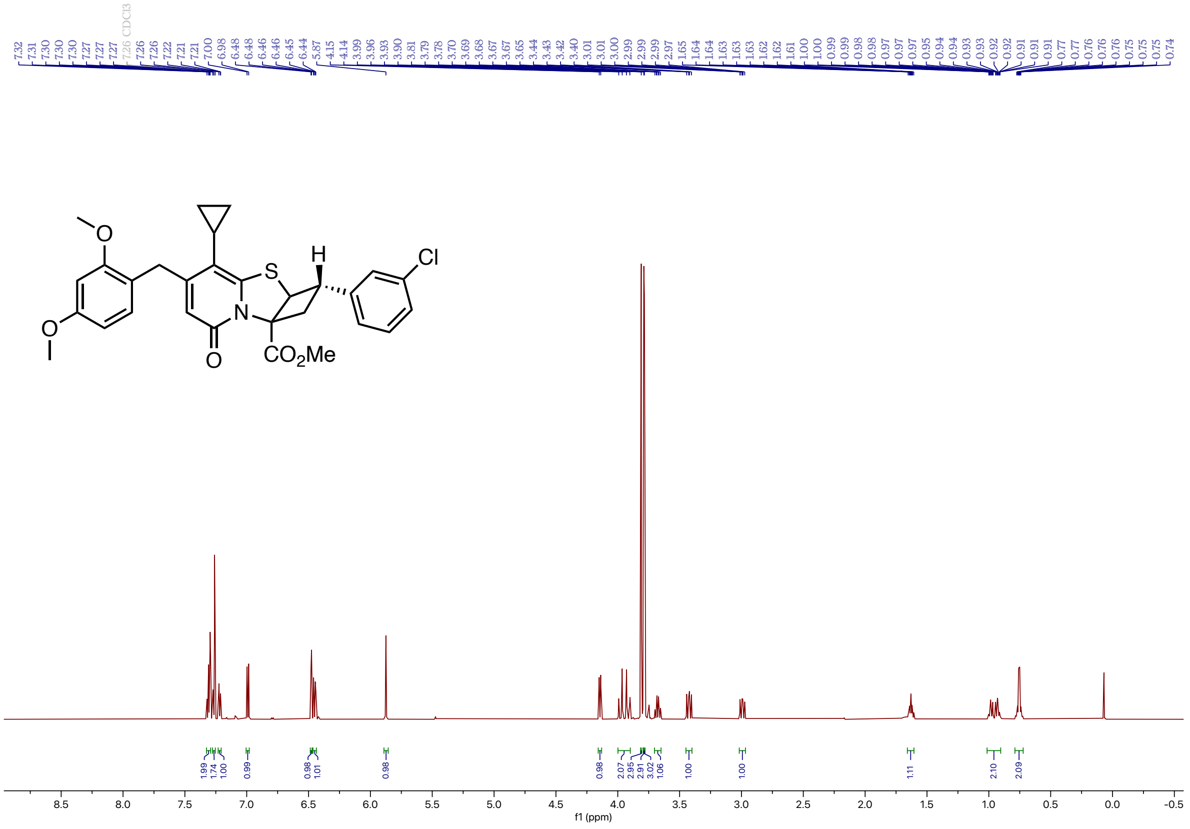

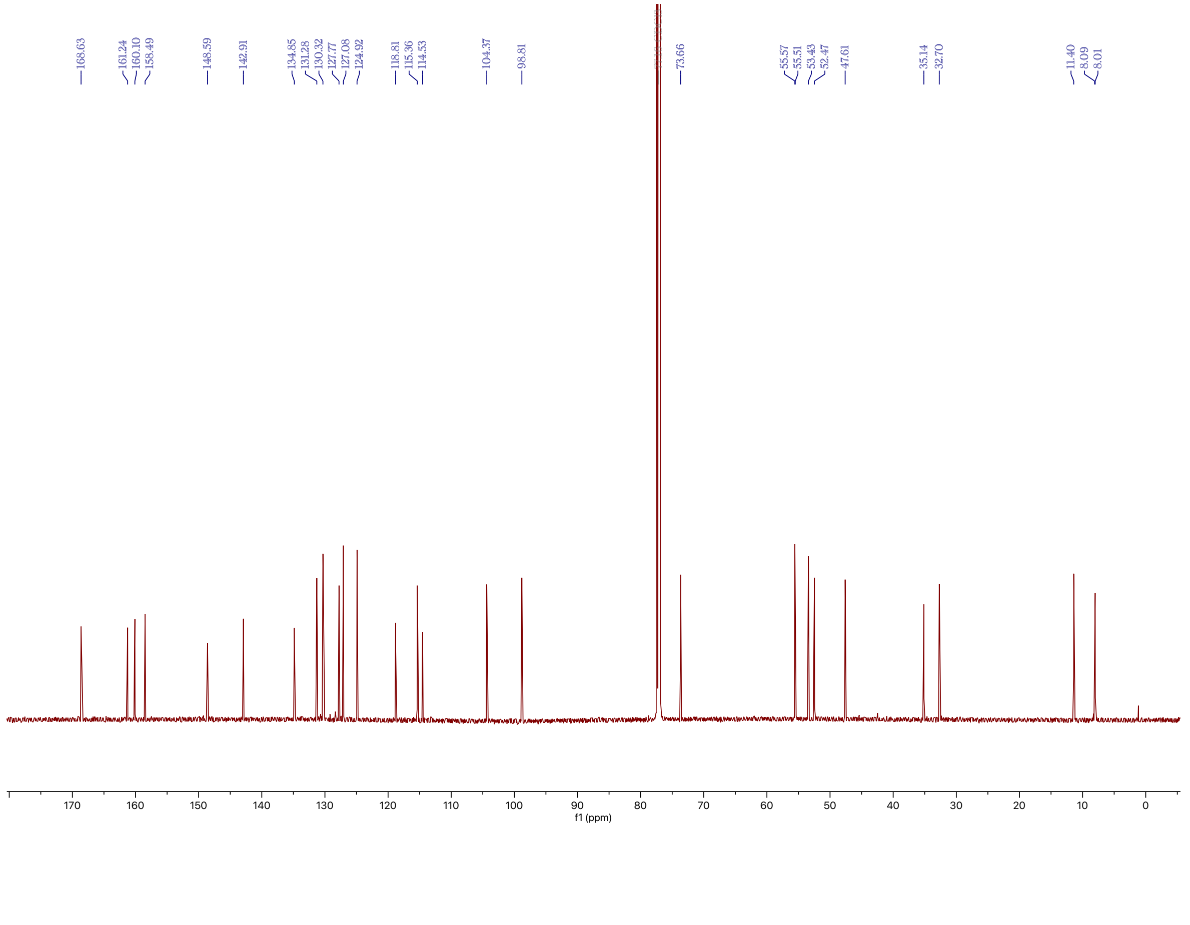

Compound **3d**, ^{1}H-NMR (600 MHz) and ^{13}C-NMR (151 MHz) ($CDCl_3$):

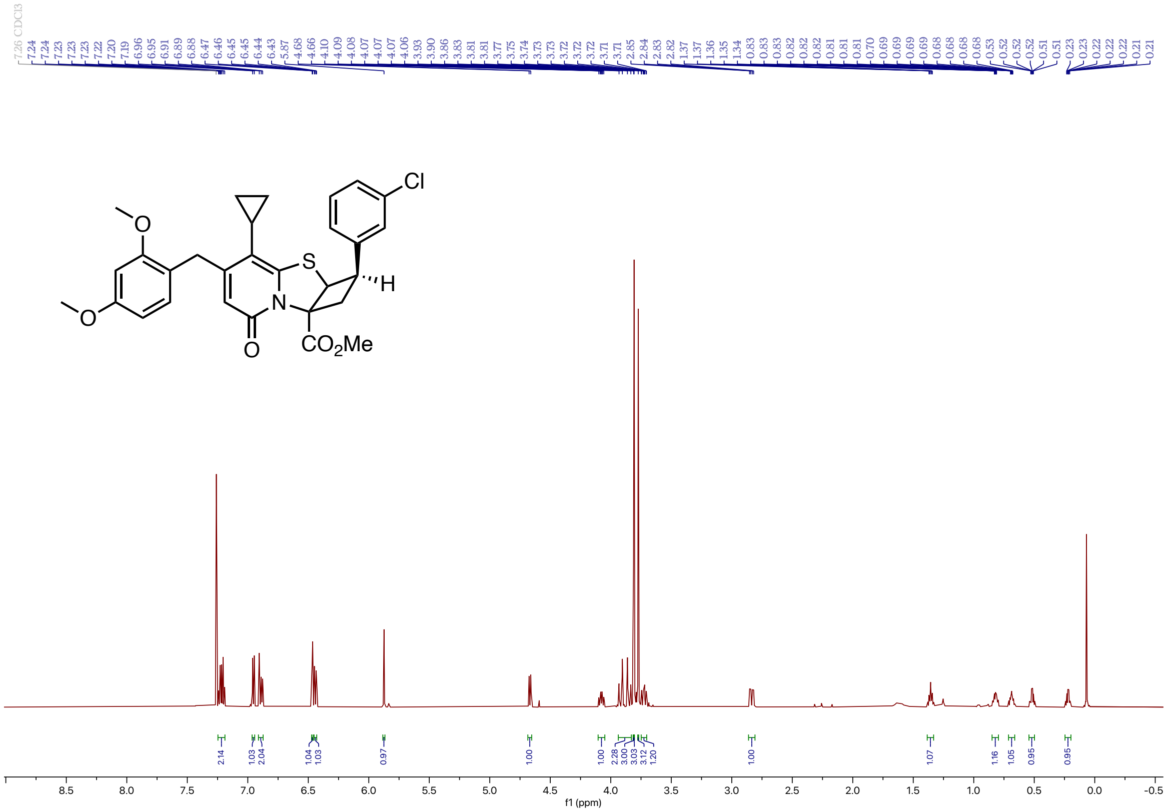

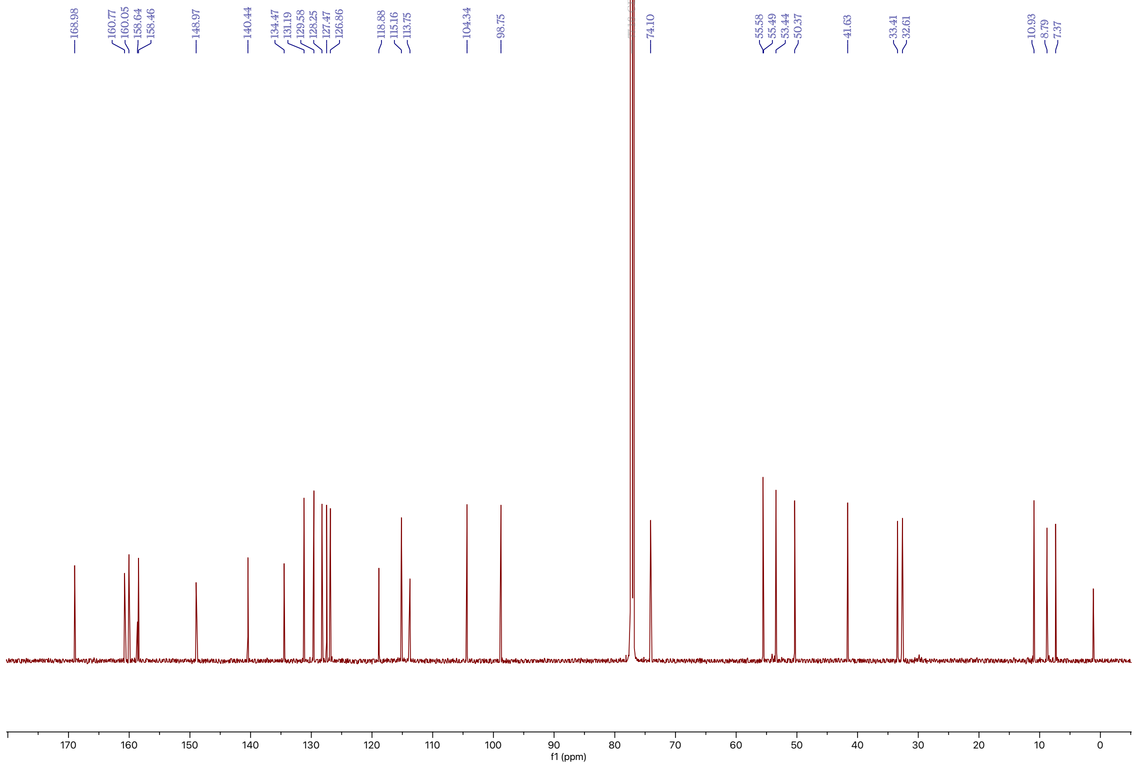

Compound **2e**, ^{1}H-NMR (600 MHz) and ^{13}C-NMR (151 MHz) ($CDCl_3$):

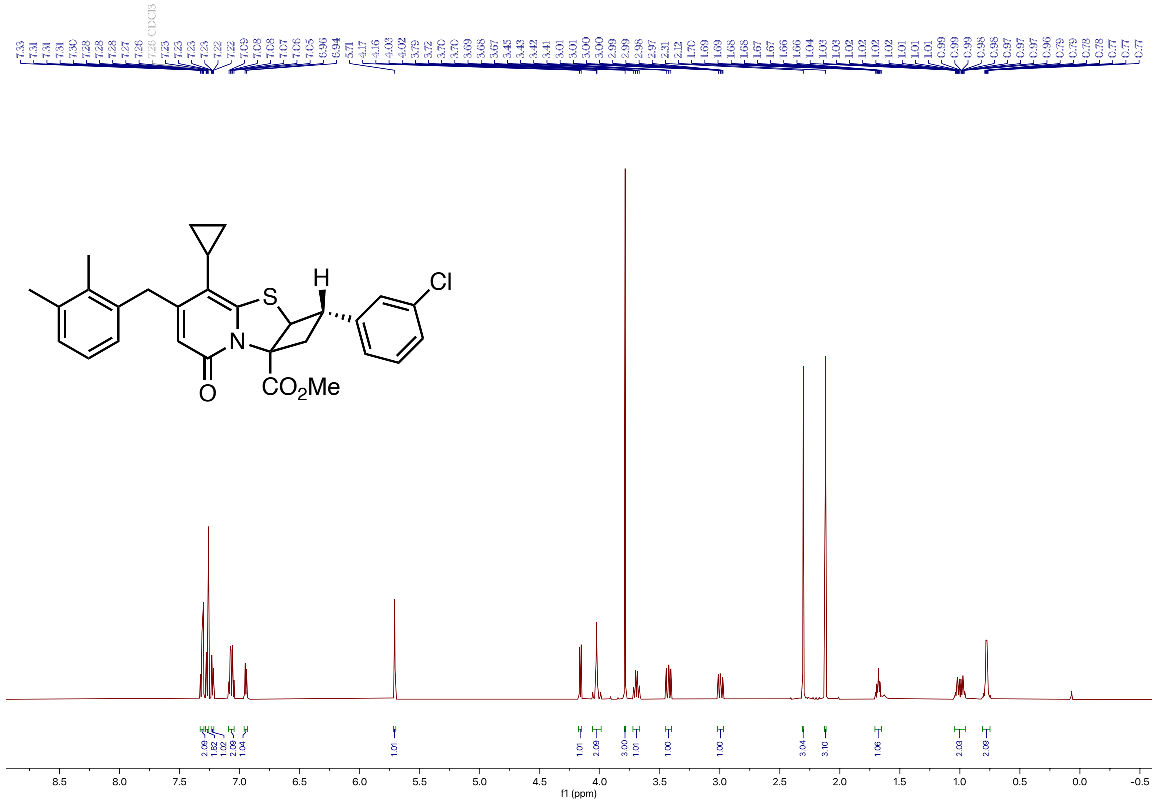

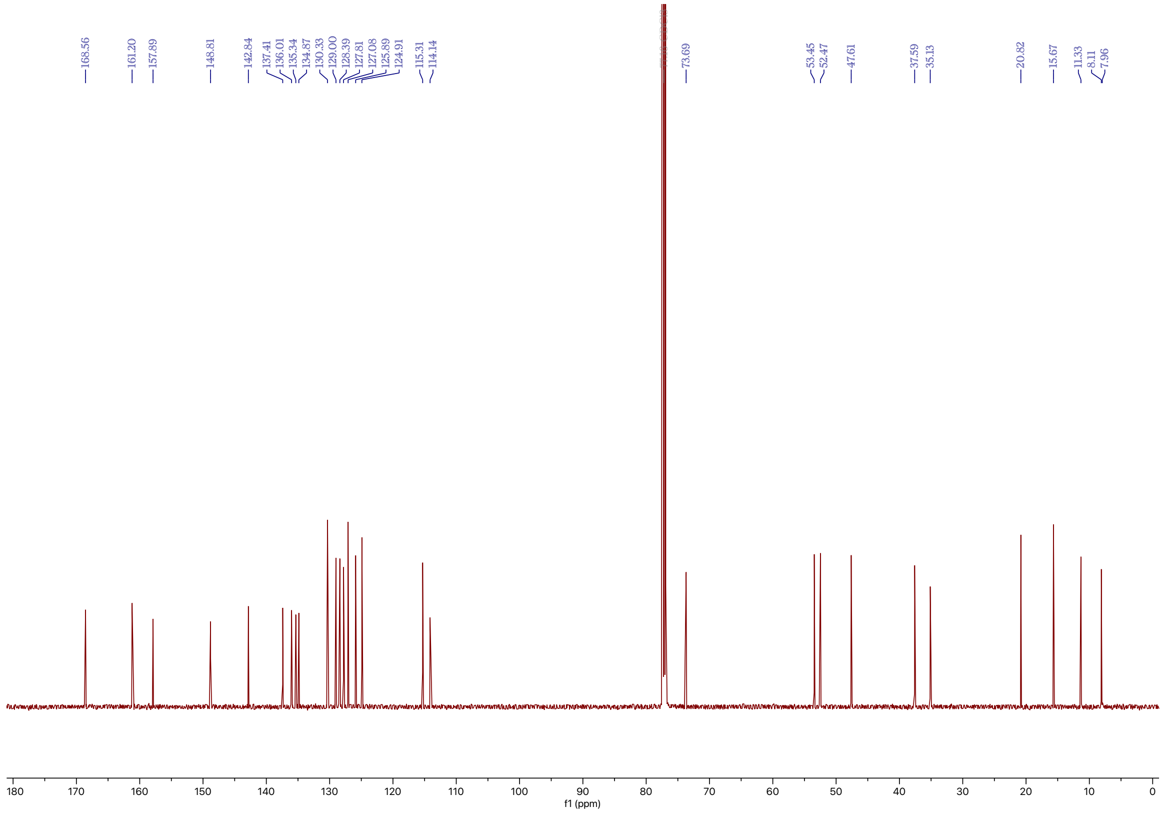

Compound **3e**, ^{1}H-NMR (600 MHz) and ^{13}C-NMR (151 MHz) ($CDCl_3$):

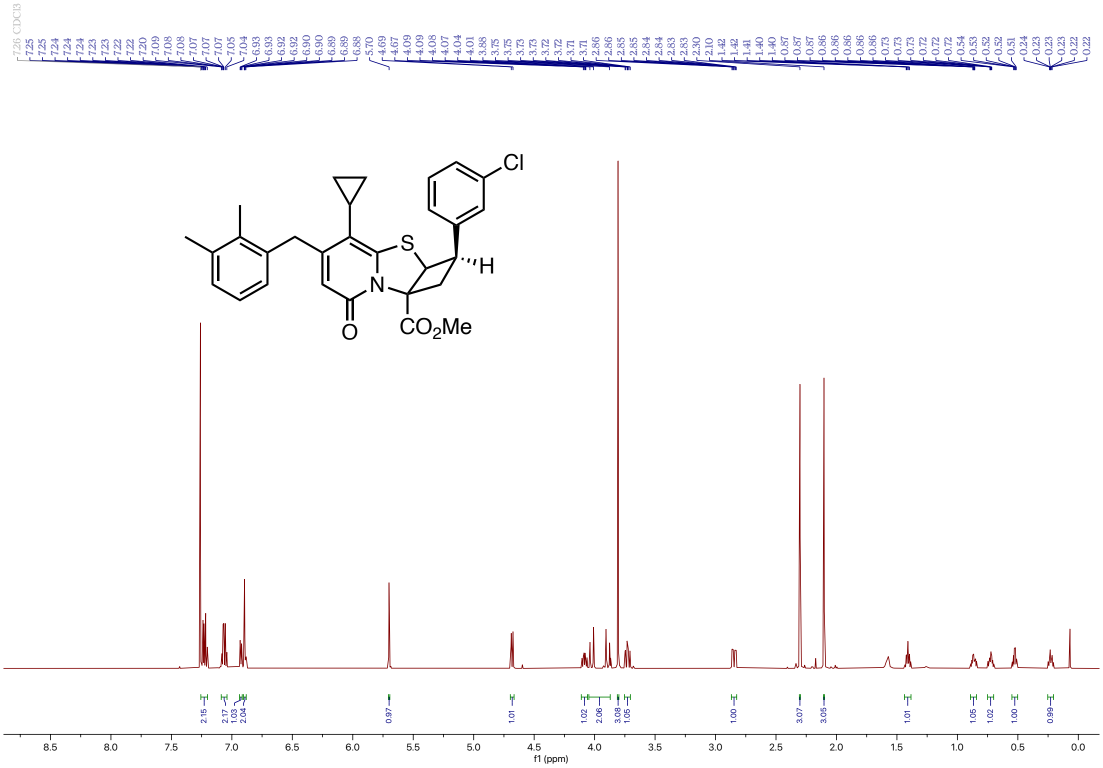

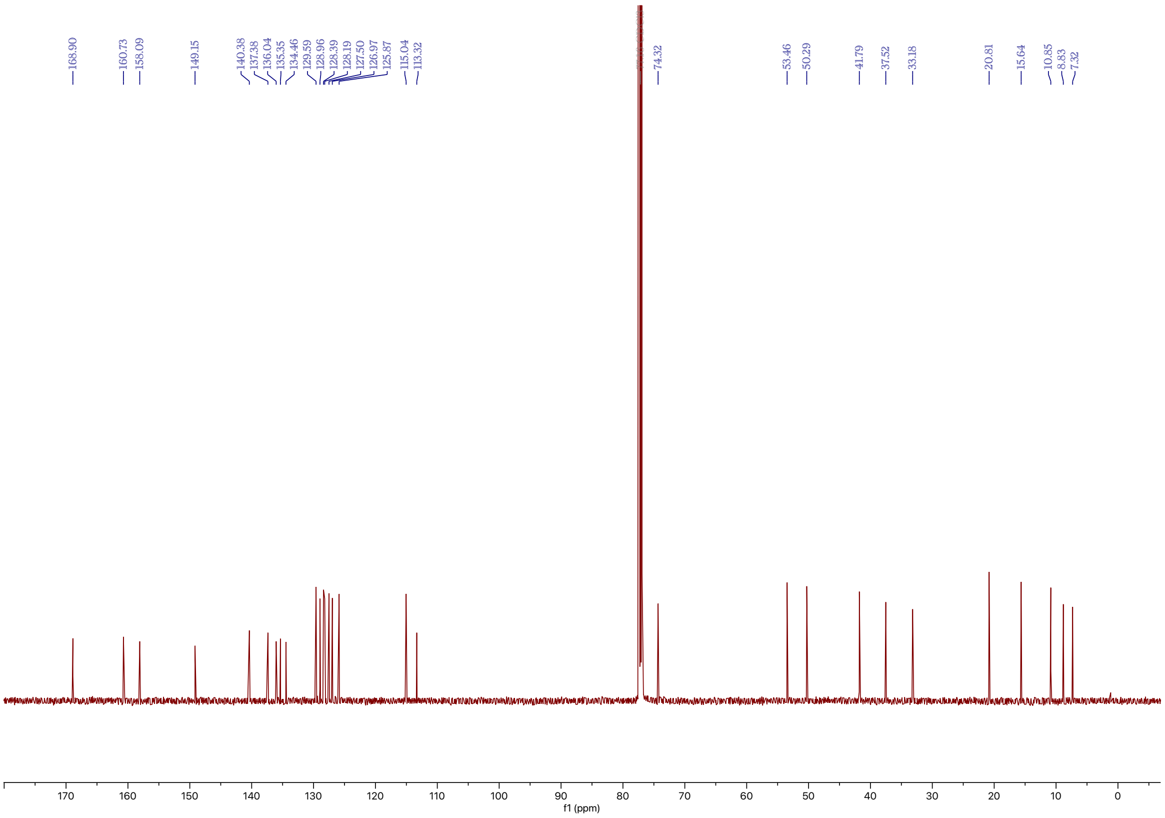

Compound **2f**, ^{1}H-NMR (600 MHz) and ^{13}C-NMR (151 MHz) ($CDCl_3$):

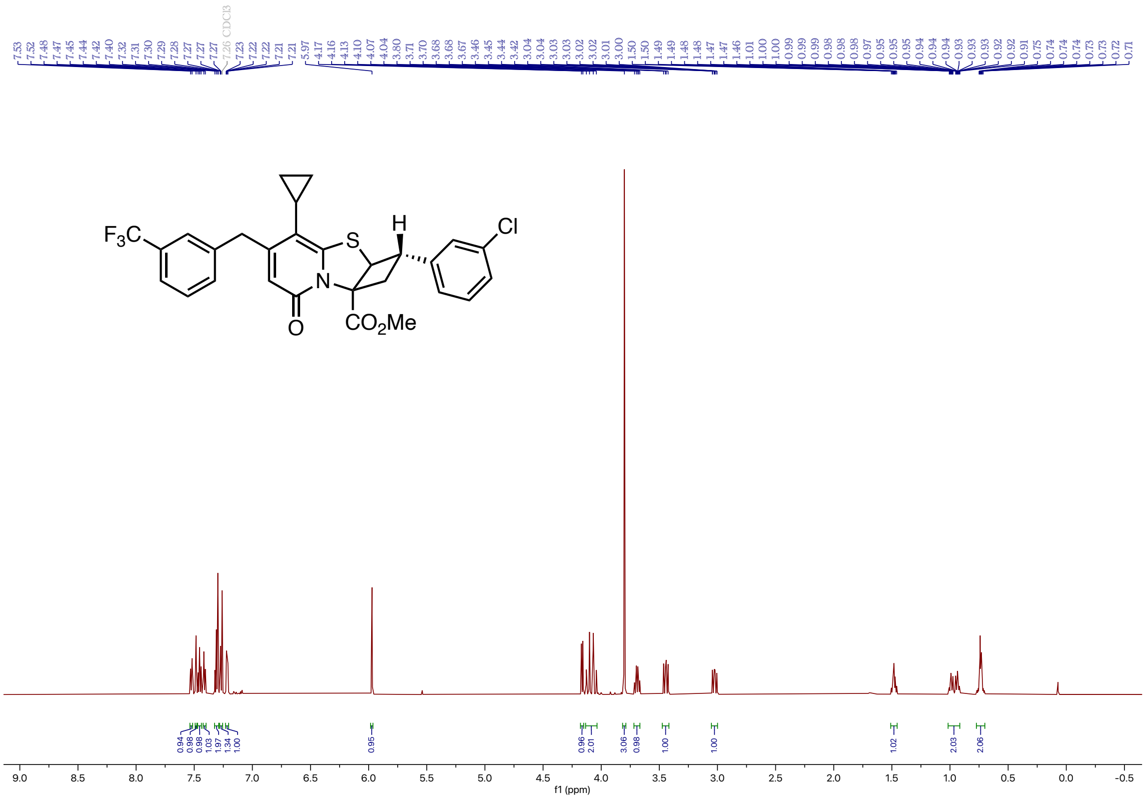

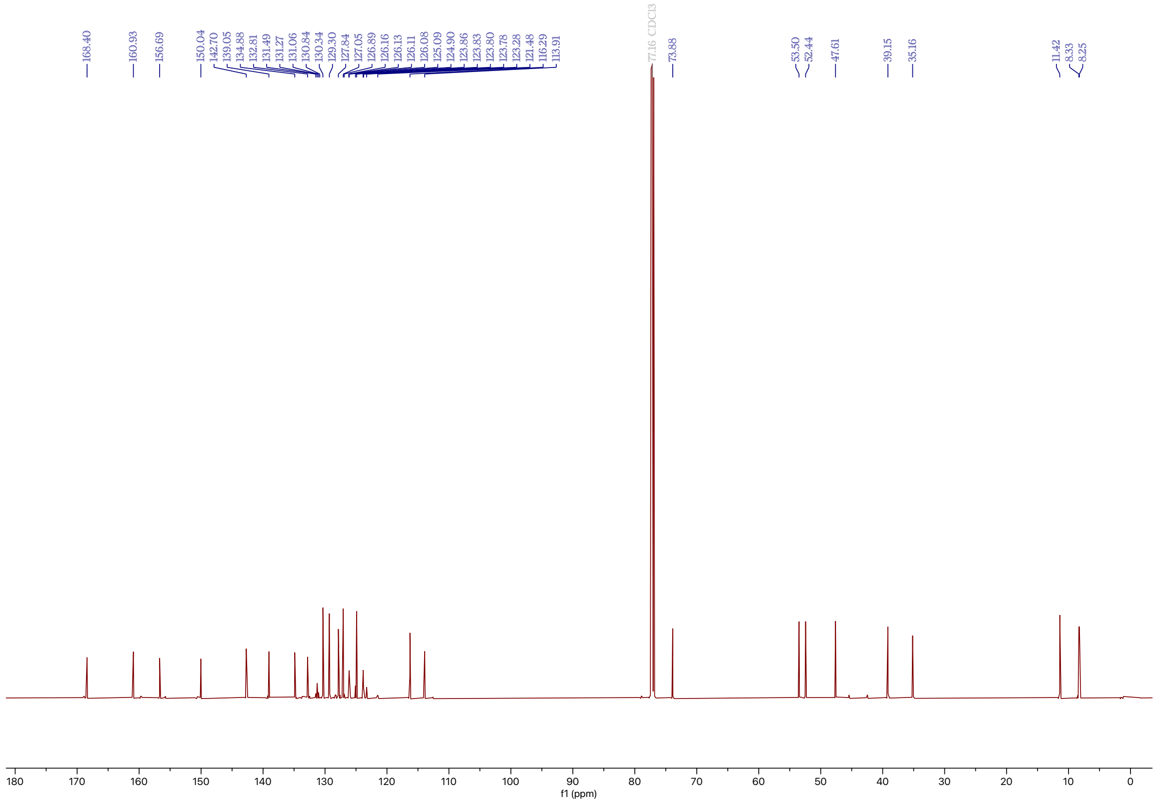

Compound **2f**, ^{19}F-NMR (565 MHz) ($CDCl_3$):

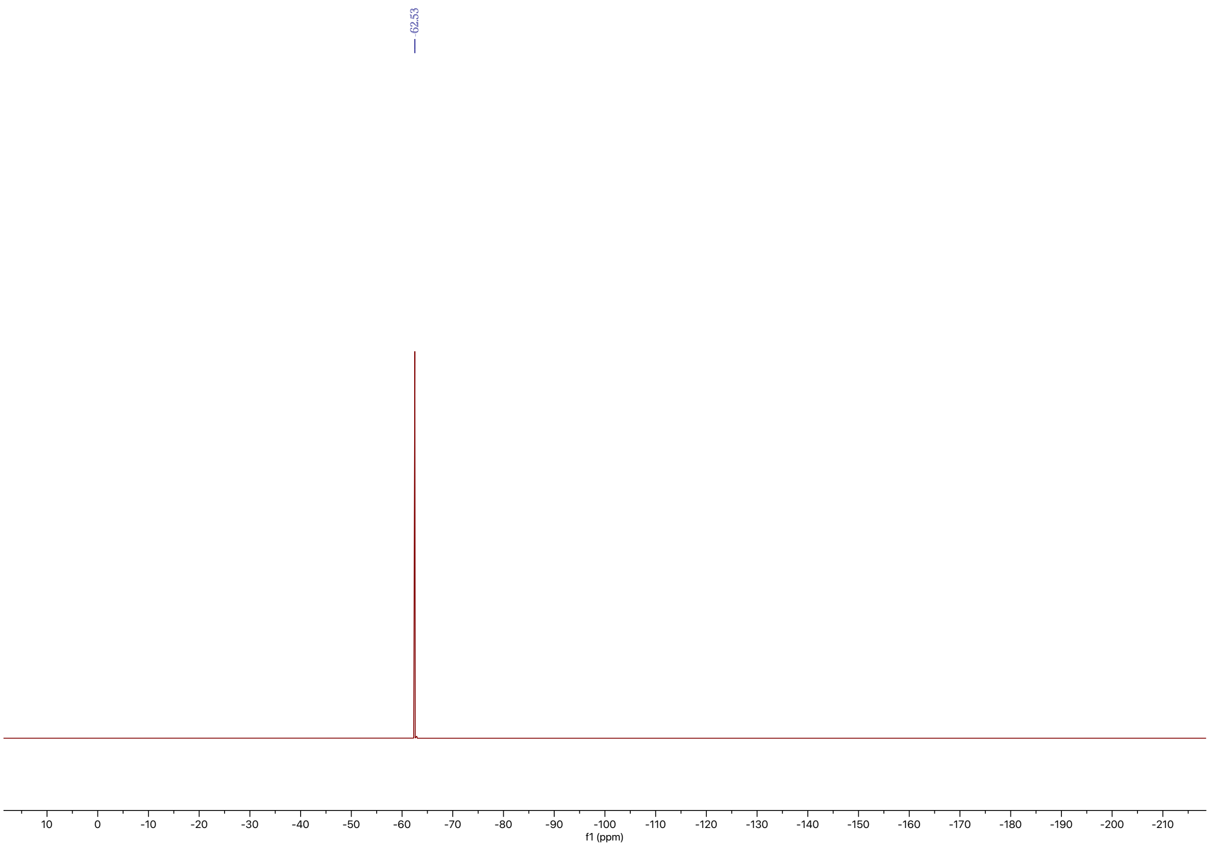

Compound **3f**, ^{1}H-NMR (600 MHz) and ^{13}C-NMR (151 MHz) ($CDCl_3$):

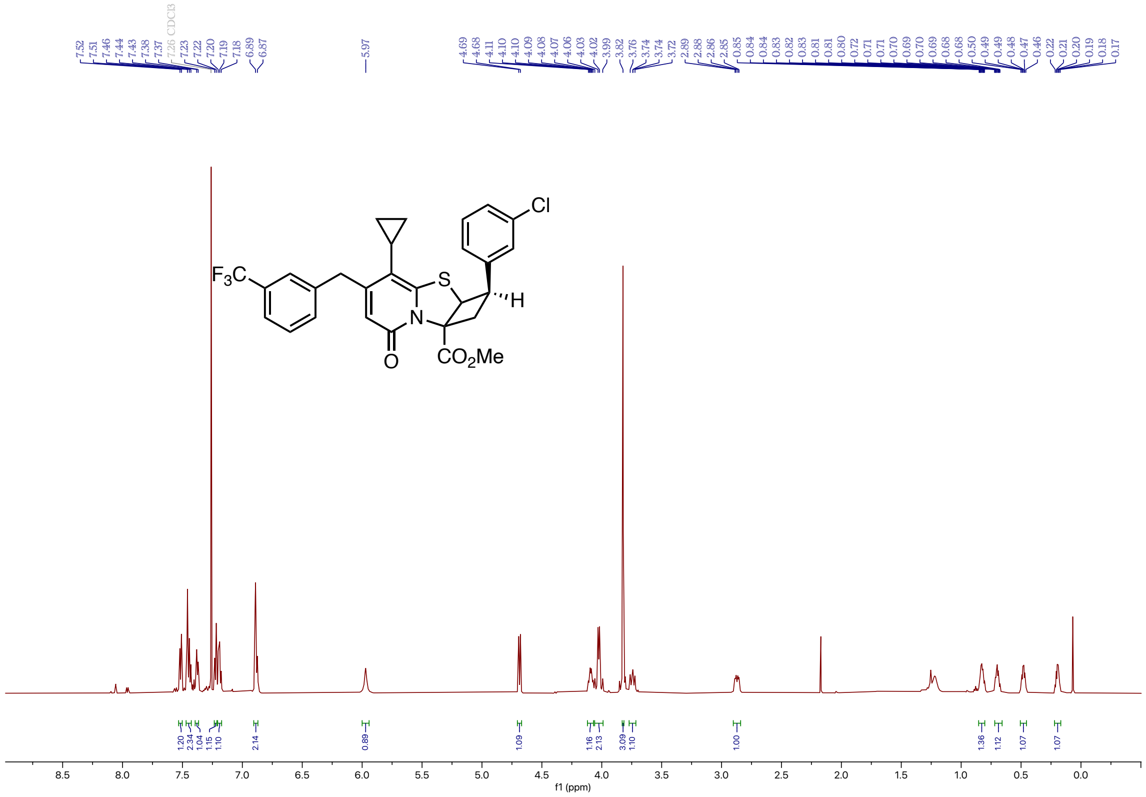

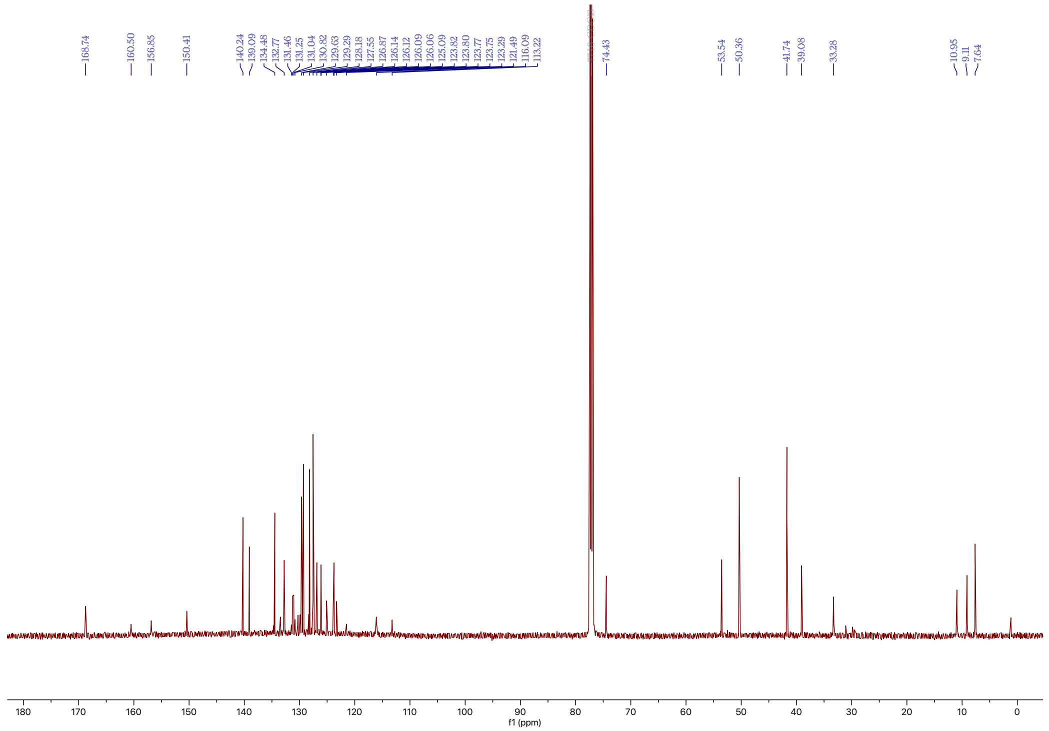

Compound **3f**, ^{19}F-NMR (565 MHz) ($CDCl_3$):

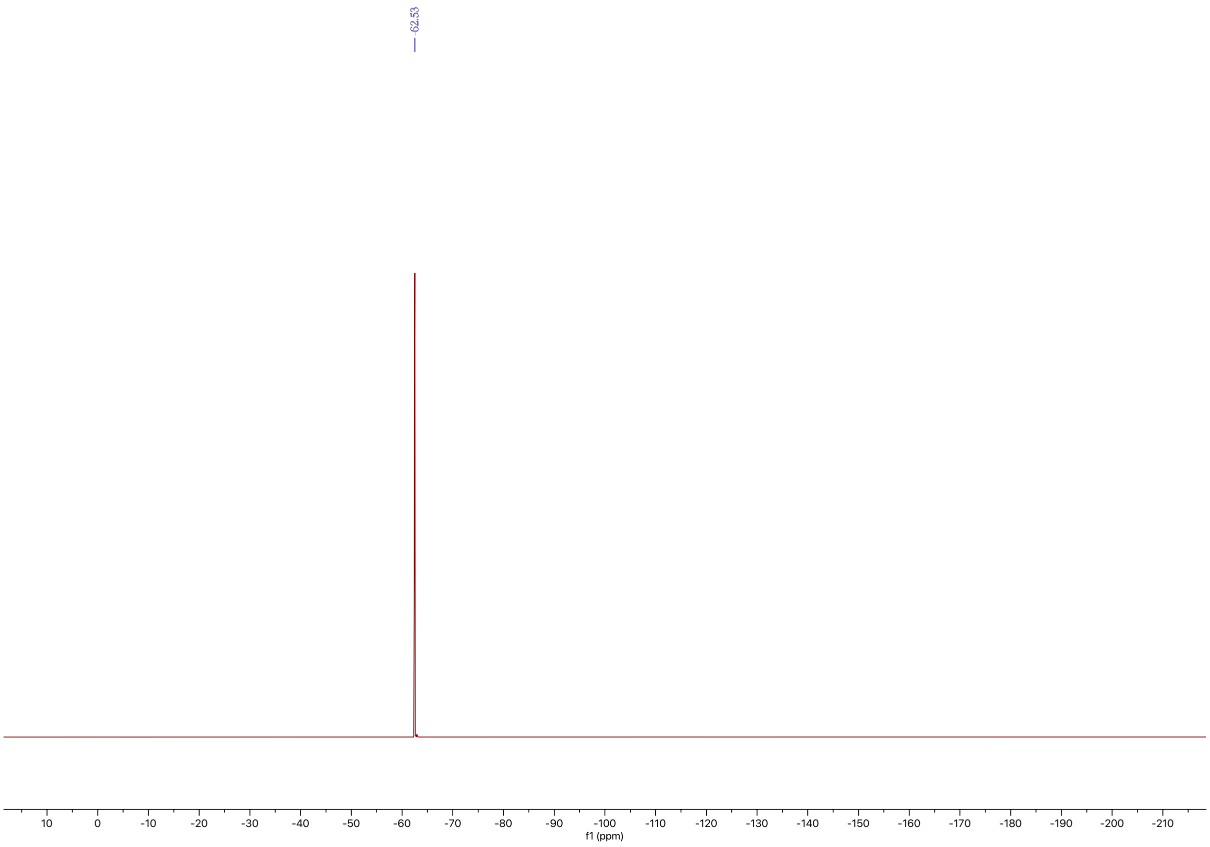

Compound **2g**, ^{1}H-NMR (600 MHz) and ^{13}C-NMR (150 MHz) ($CDCl_3$):

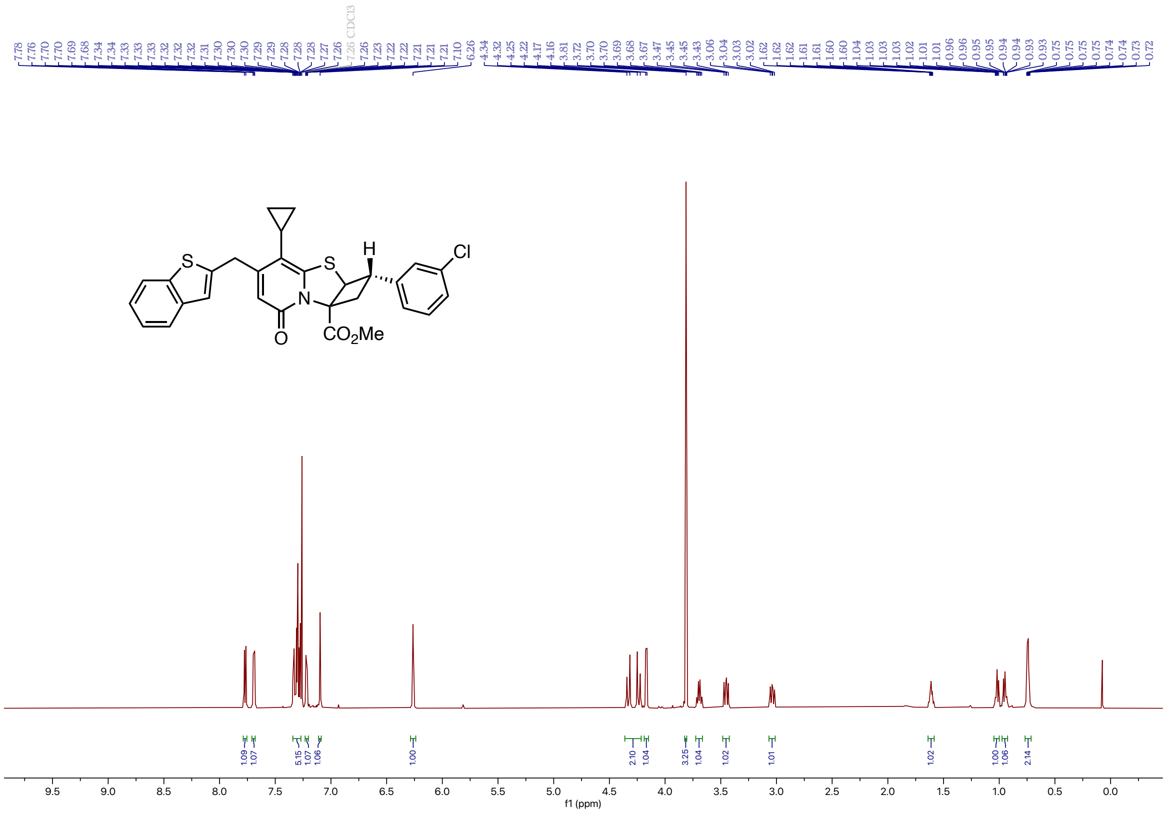

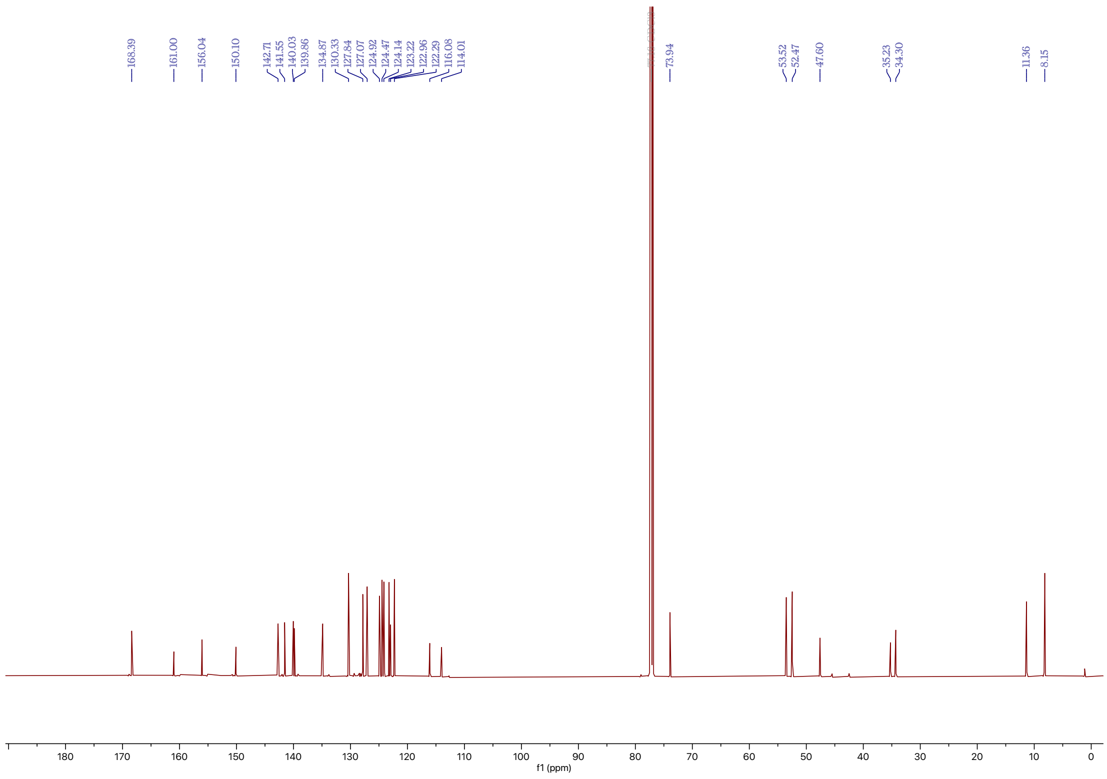

Compound **3g**, ^{1}H-NMR (400 MHz) and ^{13}C-NMR (100 MHz) ($CDCl_3$):

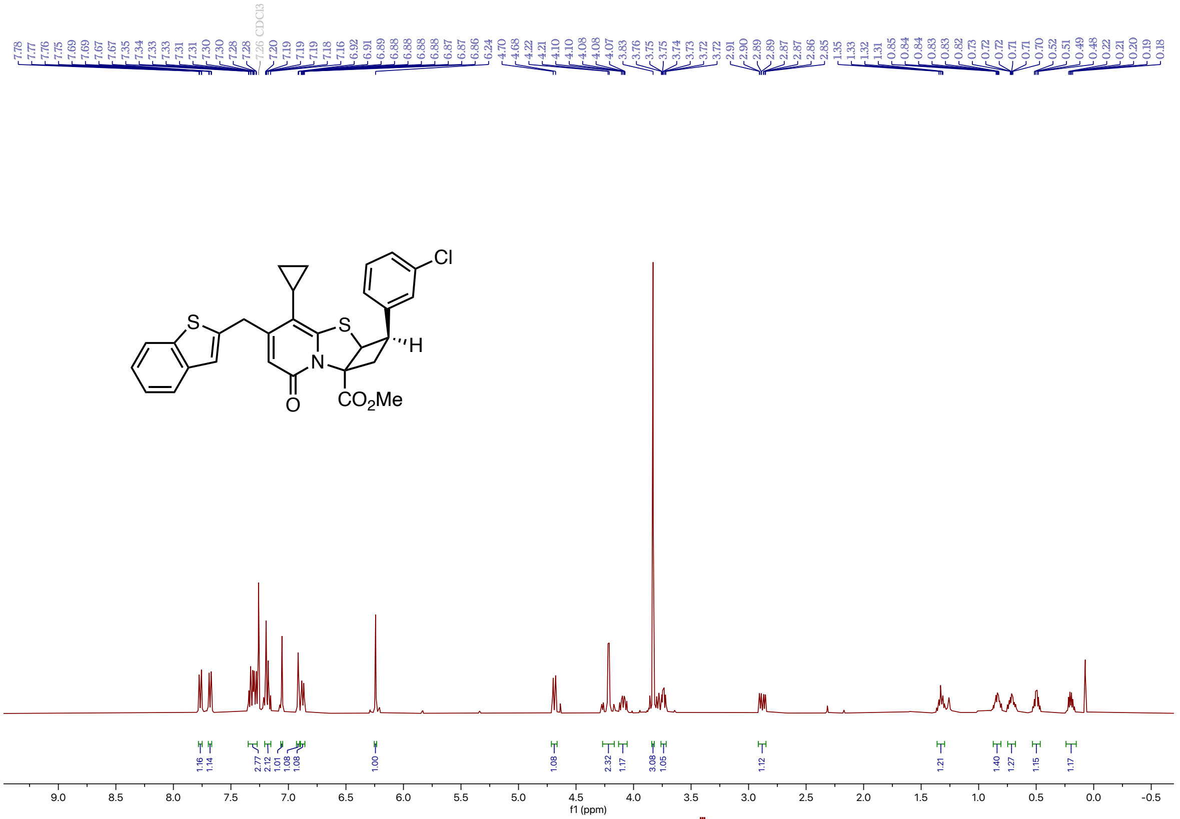

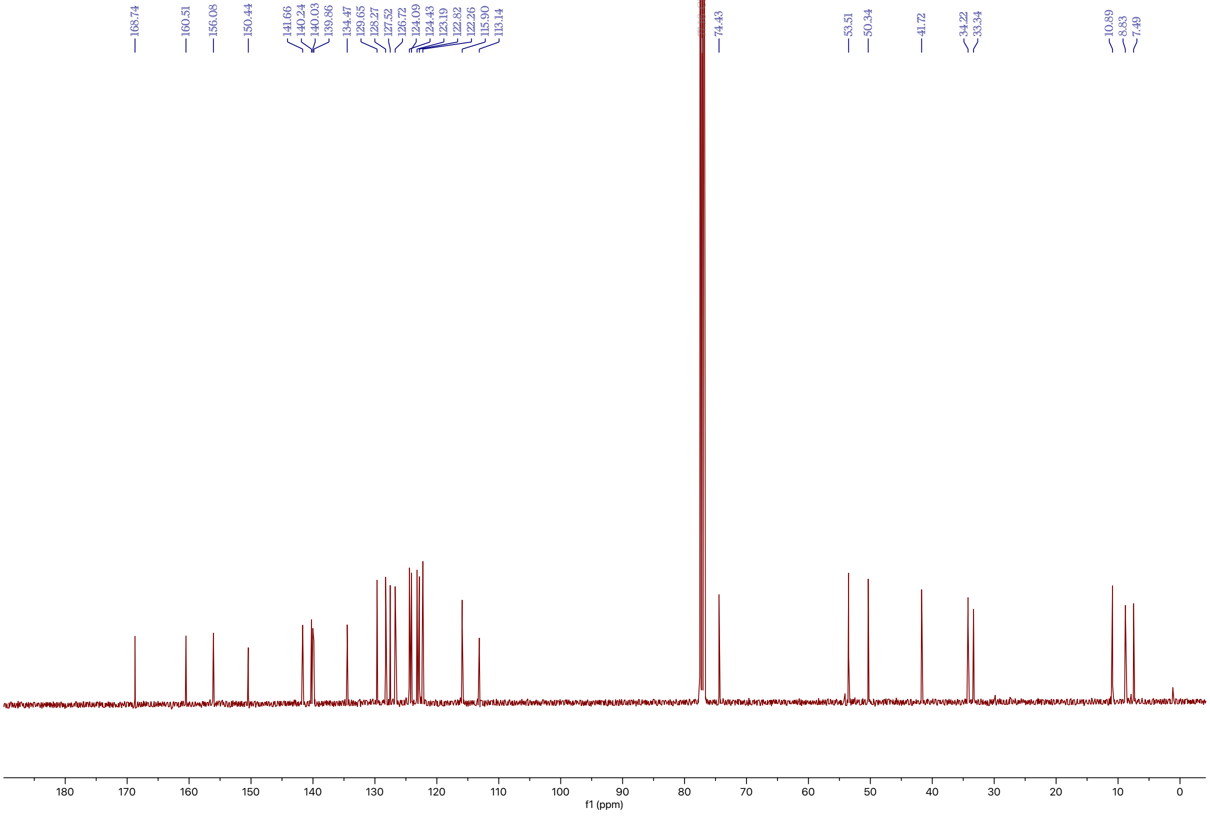

Compound **2h**, ^{1}H-NMR (400 MHz), ^{13}C-NMR (100 MHz) and ^{19}F NMR (376 MHz) ($CDCl_3$):

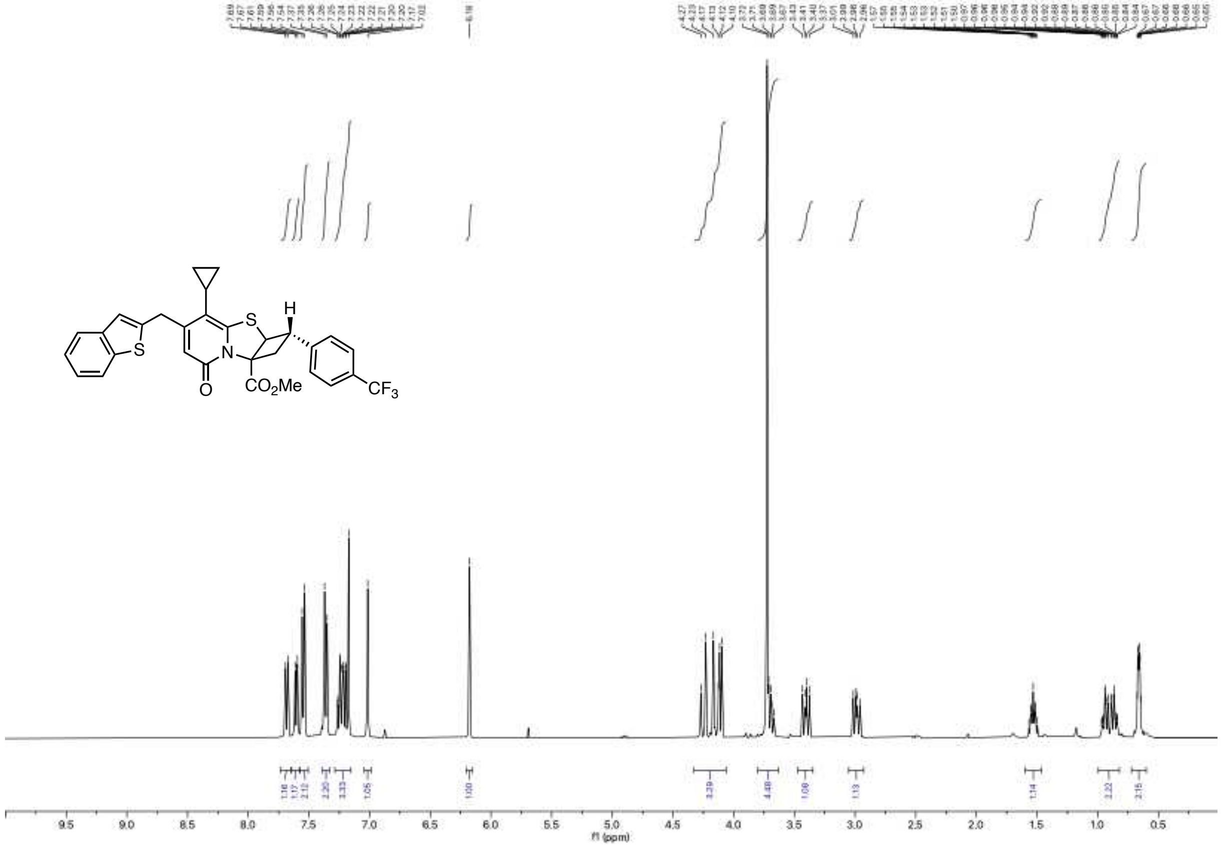

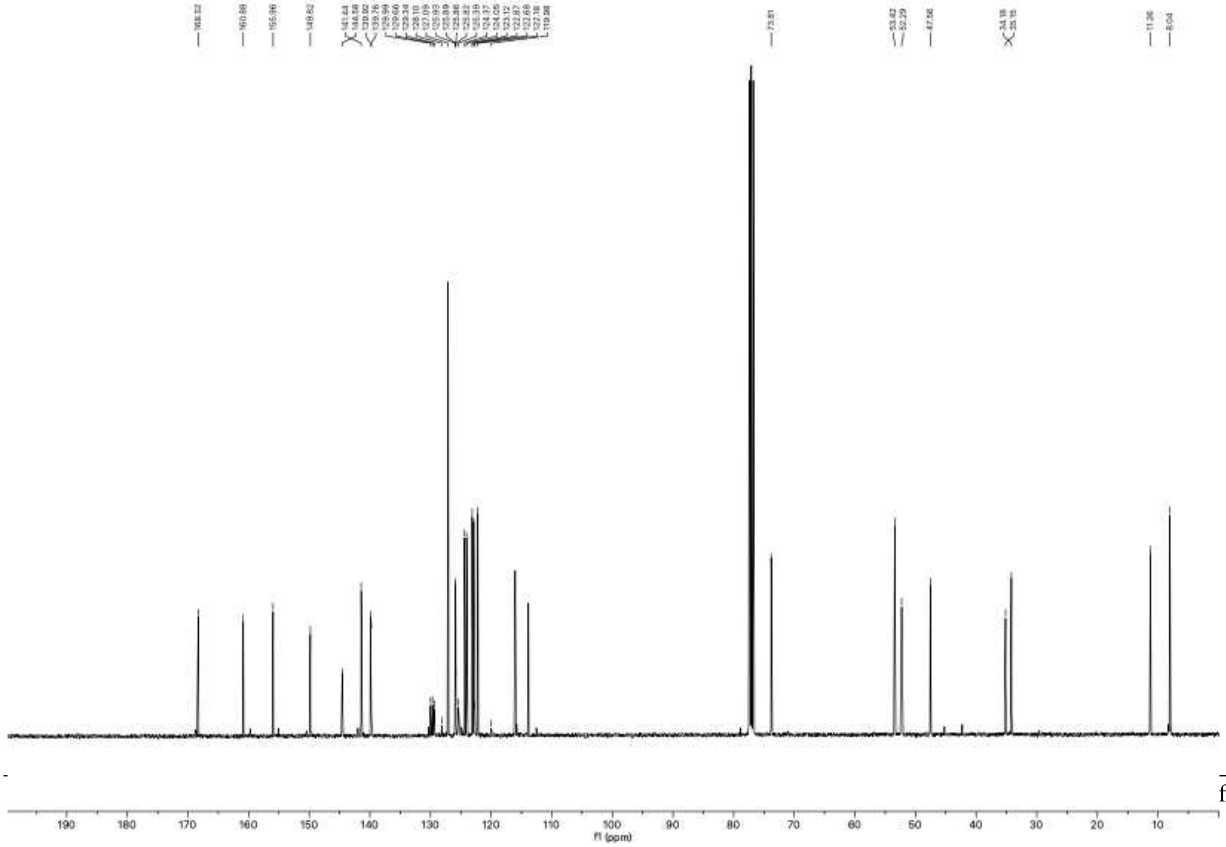

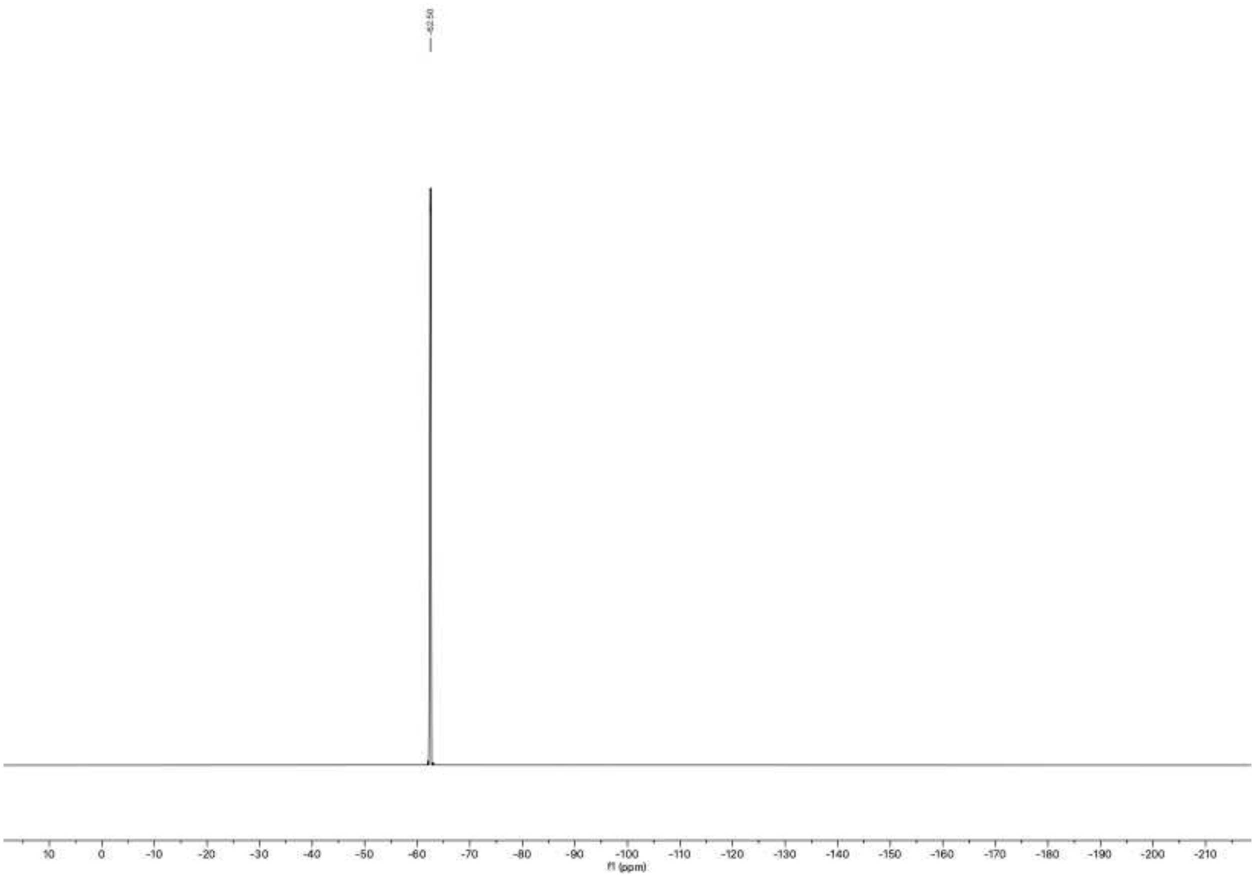
-62.50
10 0 -10 -20 -30 -40 -50 -60 -70 -80 -90 -100 -110 -120 -130 -140 -150 -160 -170 -180 -190 -200 -210
f1 (ppm)

Compound **3h**, ^{1}H-NMR (400 MHz), ^{13}C-NMR (151 MHz) and ^{19}F NMR (376 MHz) ($CDCl_3$):

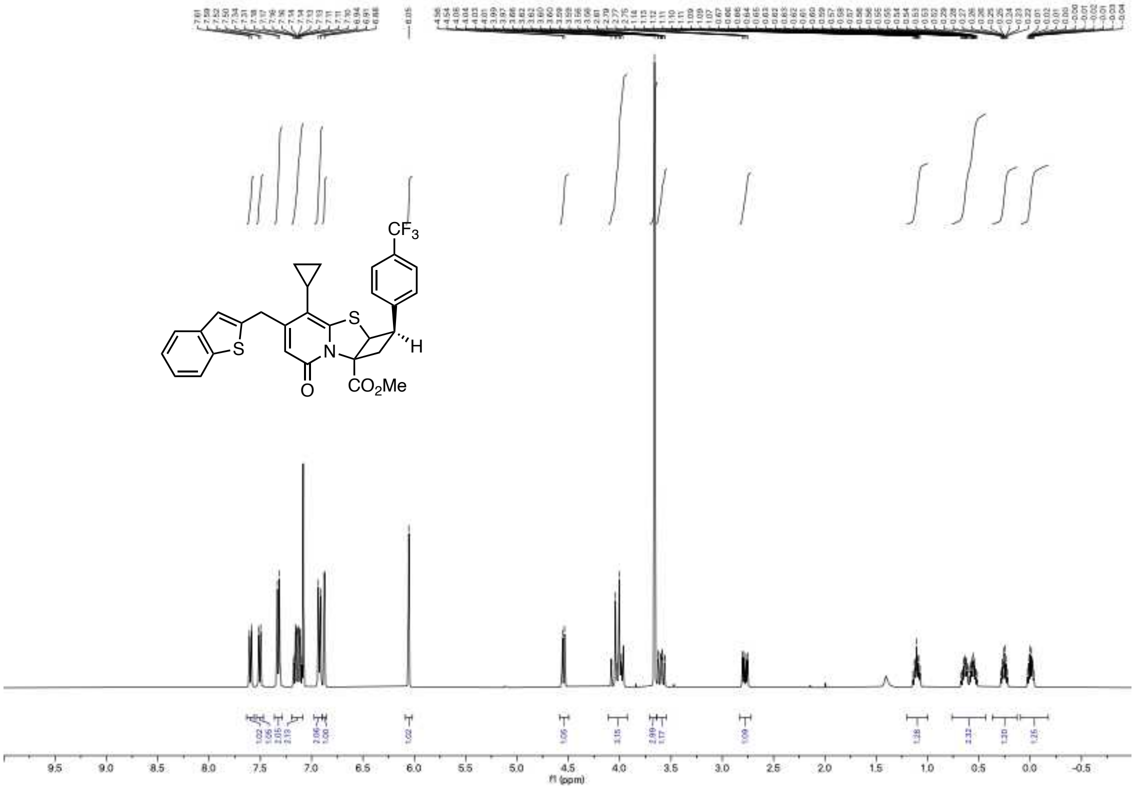

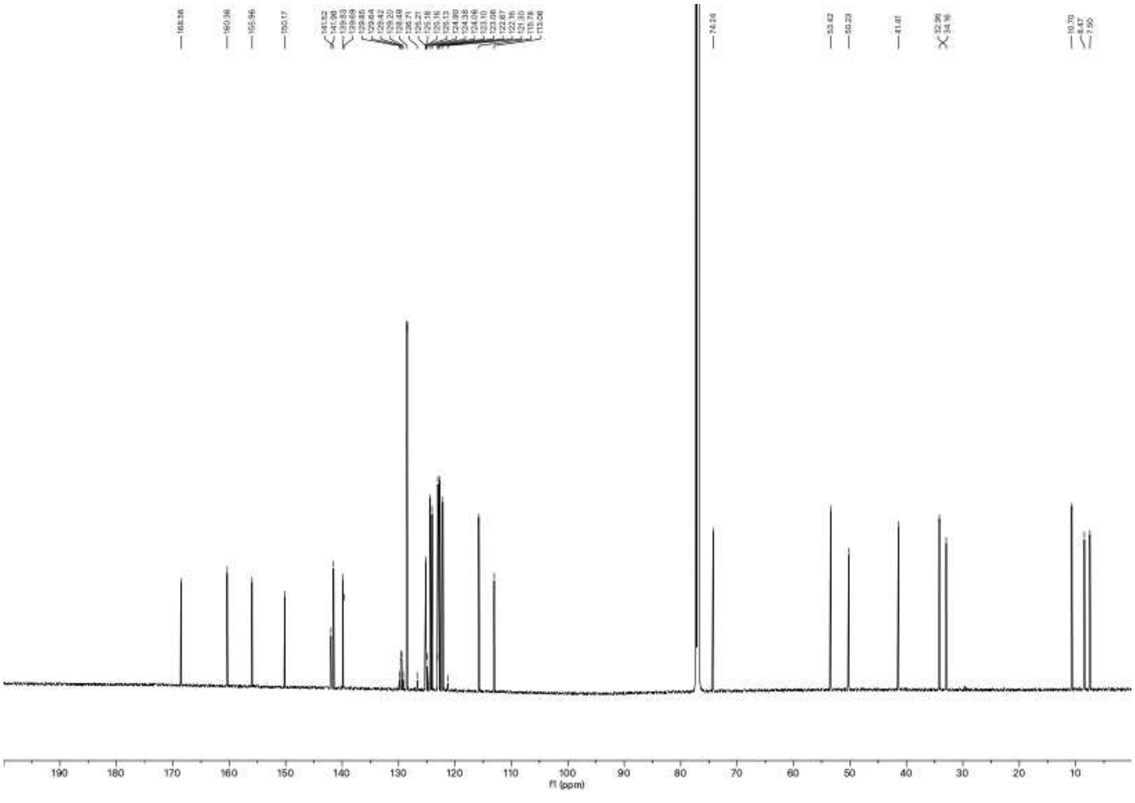

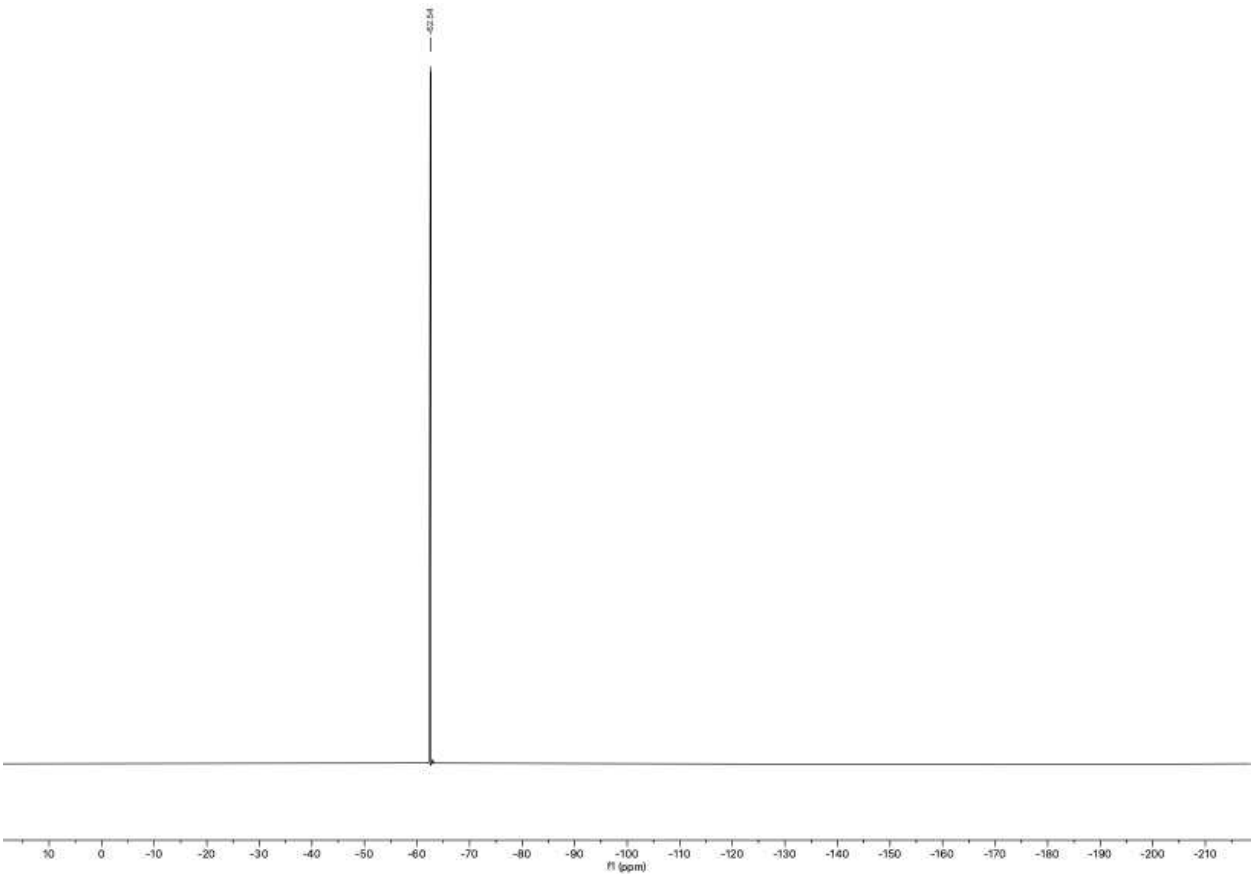
-62.54
10 0 -10 -20 -30 -40 -50 -60 -70 -80 -90 -100 -110 -120 -130 -140 -150 -160 -170 -180 -190 -200 -210
f1 (ppm)

Compound **2i**, ^{1}H-NMR (400 MHz) and ^{13}C-NMR (100 MHz) ($CDCl_3$):

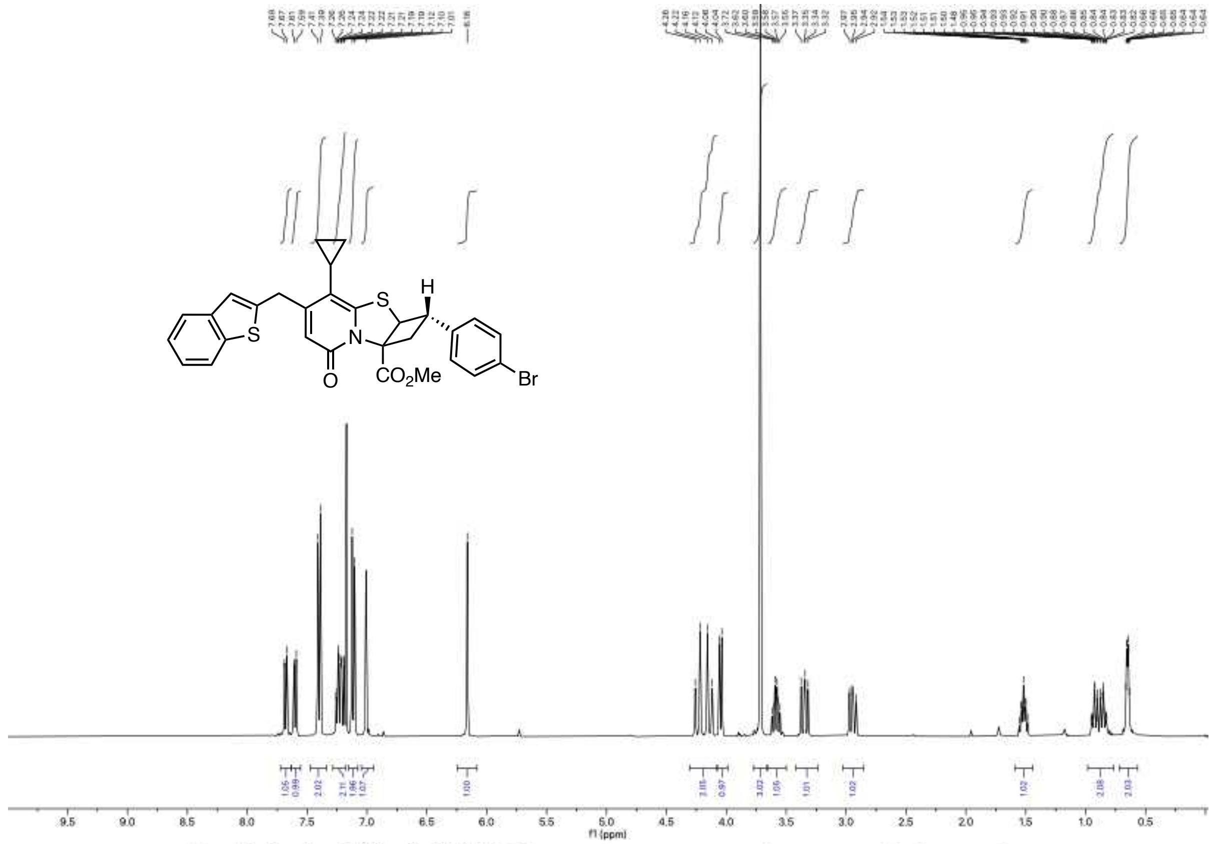

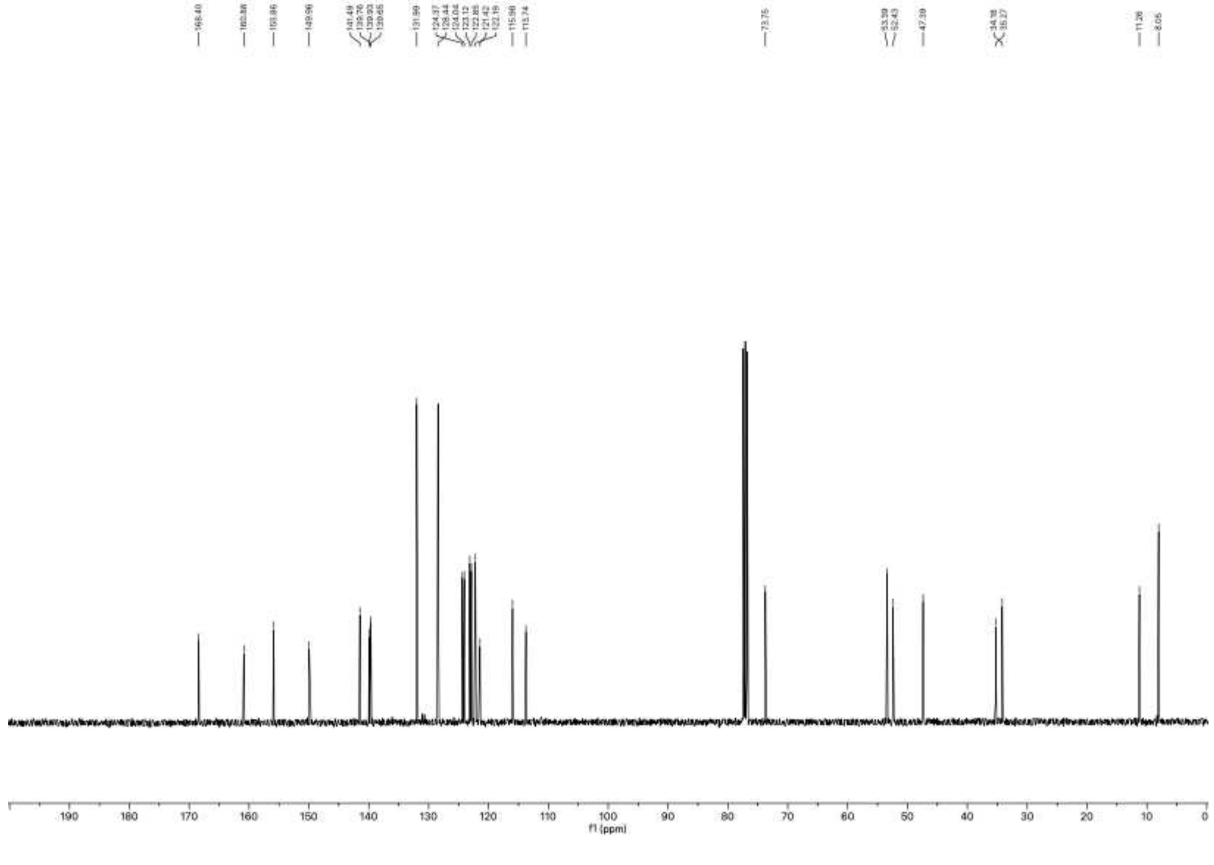

Compound **3i**, ^{1}H-NMR (400 MHz) and ^{13}C-NMR (100 MHz) ($CDCl_3$):

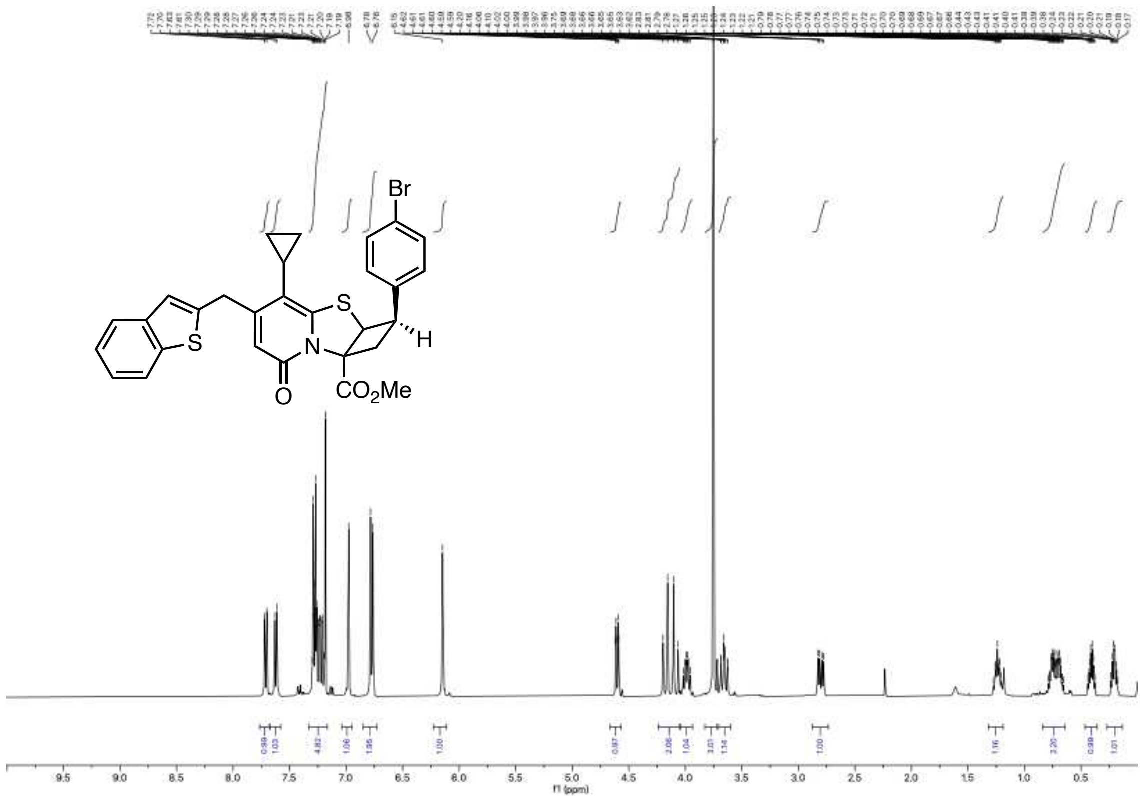

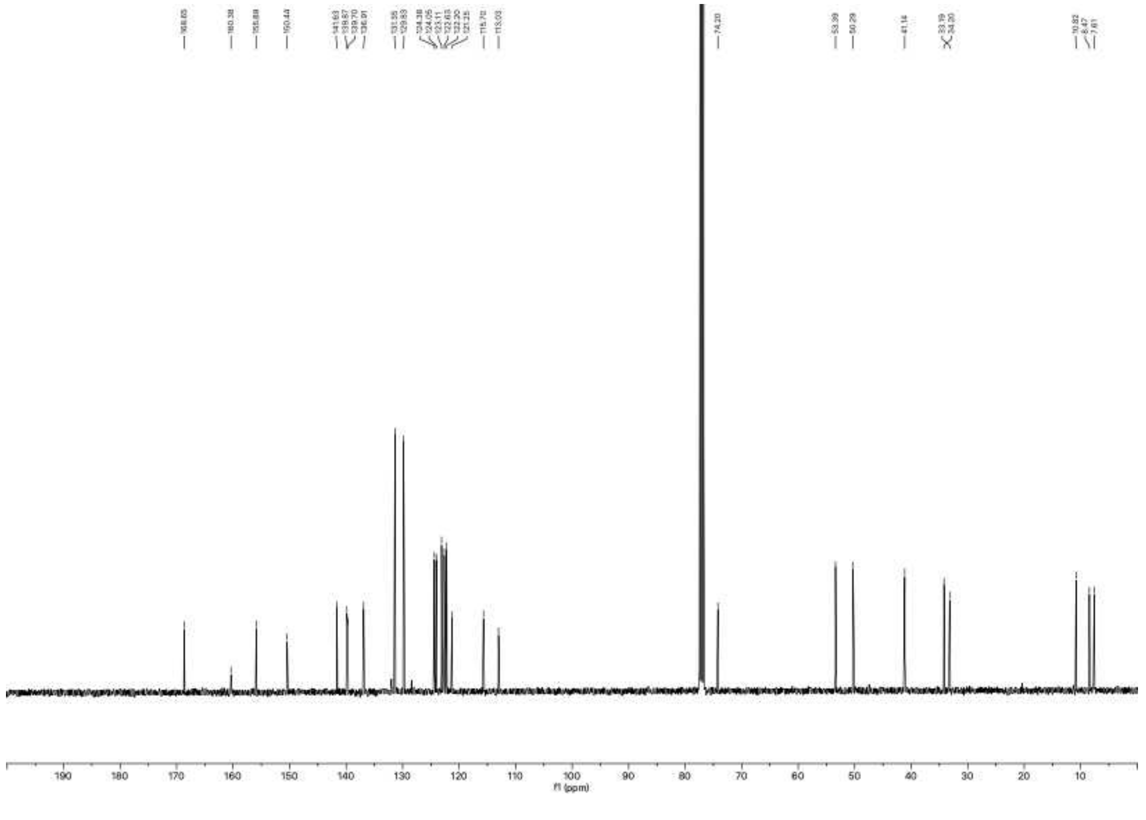

Compound **2j**, ^{1}H-NMR (400 MHz) and ^{13}C-NMR (151 MHz) ($CDCl_3$):

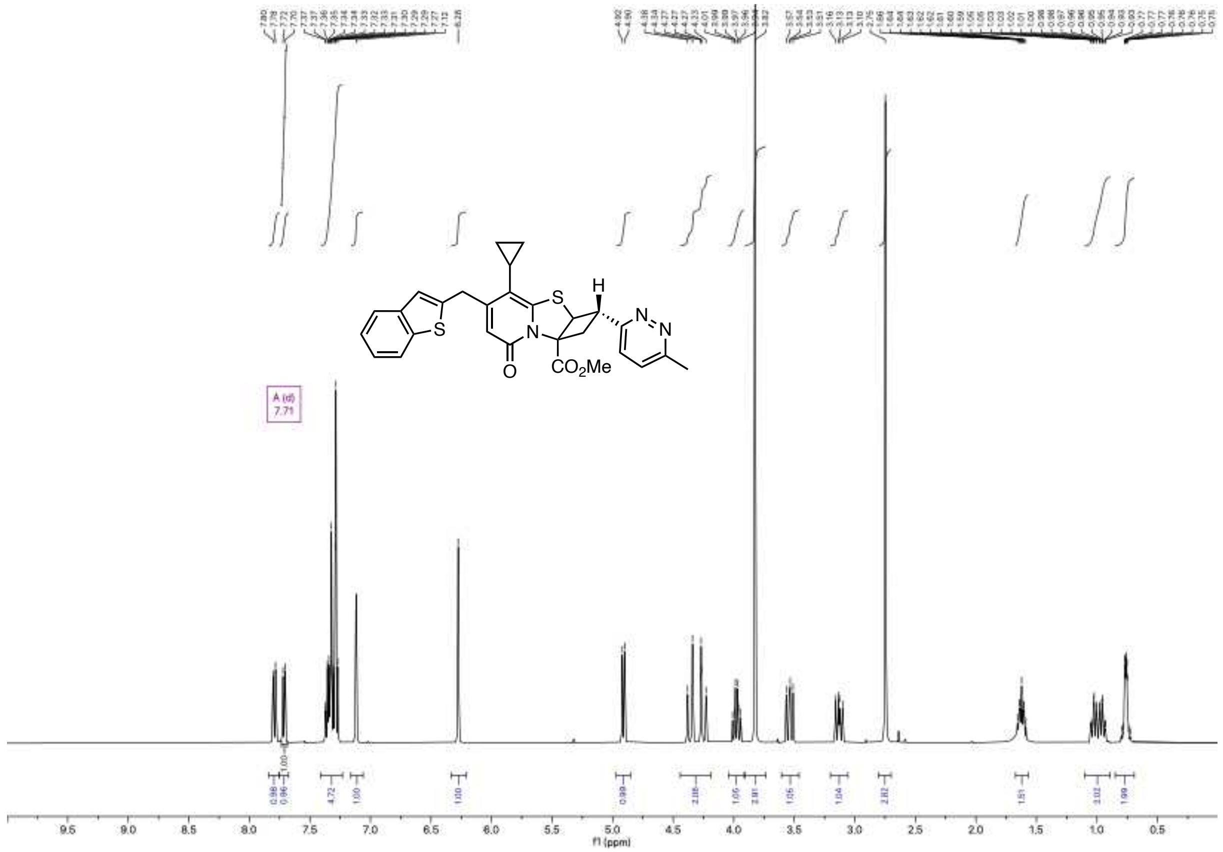

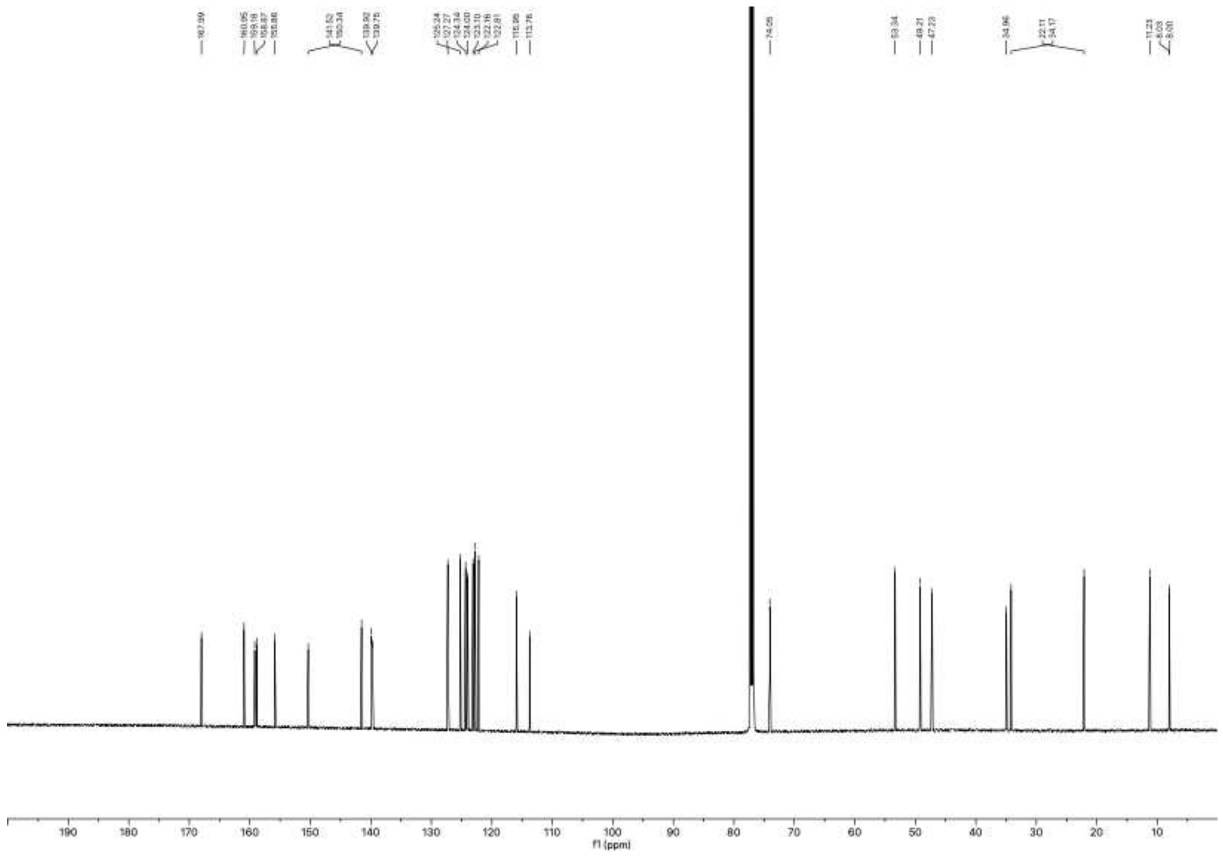

Compound **2k**, ^{1}H-NMR (600 MHz) and ^{13}C-NMR (150 MHz) ($CDCl_3$):

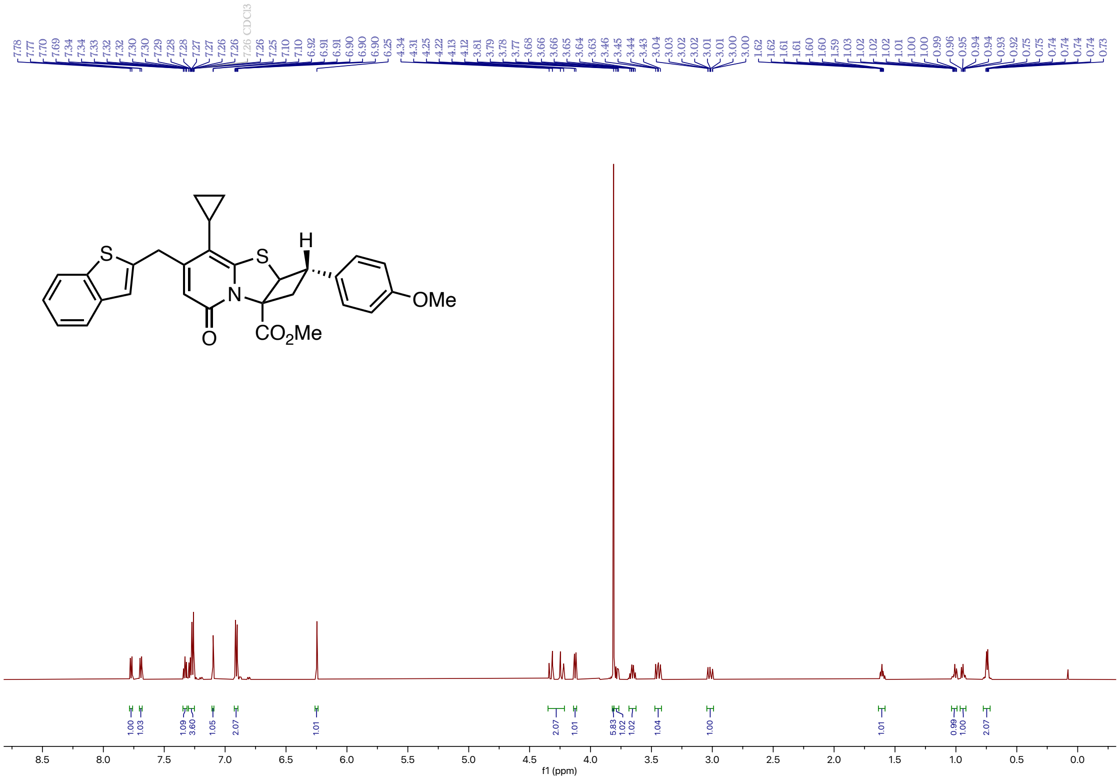

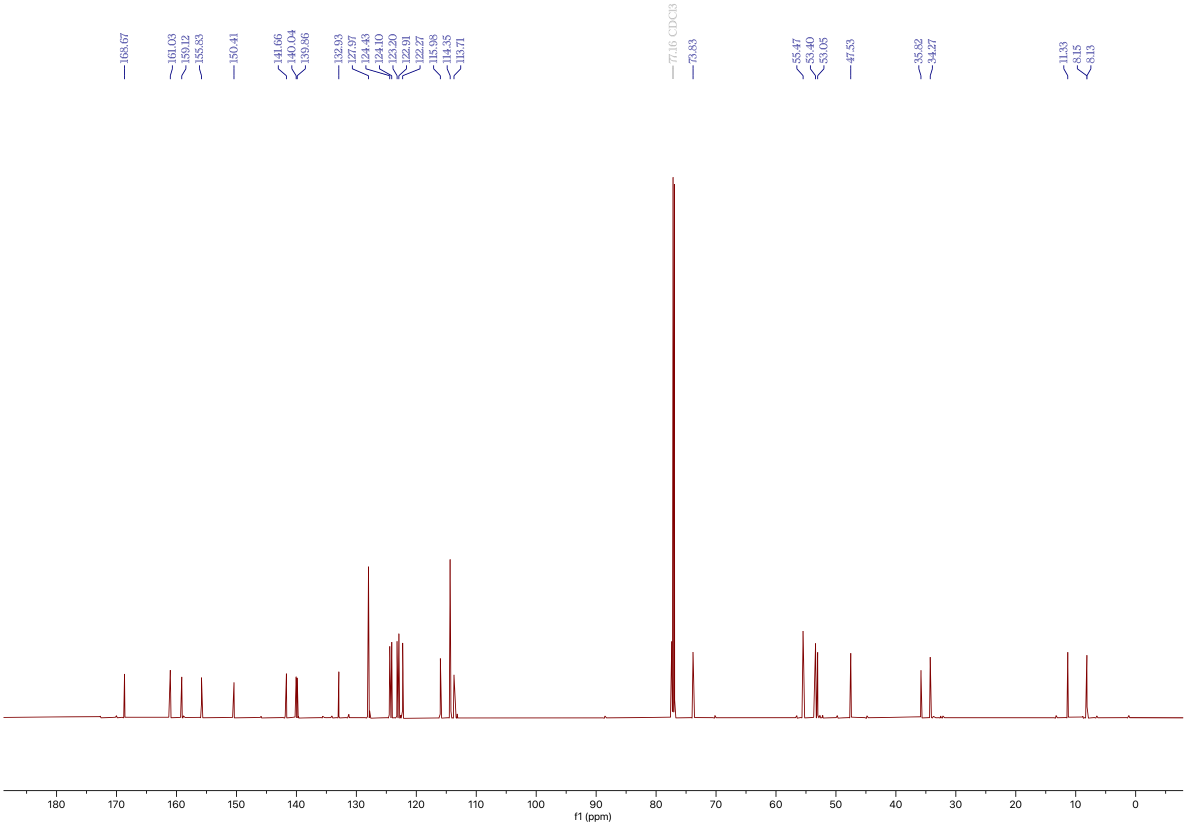

Compound **3k**, ^{1}H-NMR (600 MHz) and ^{13}C-NMR (150 MHz) ($CDCl_3$):

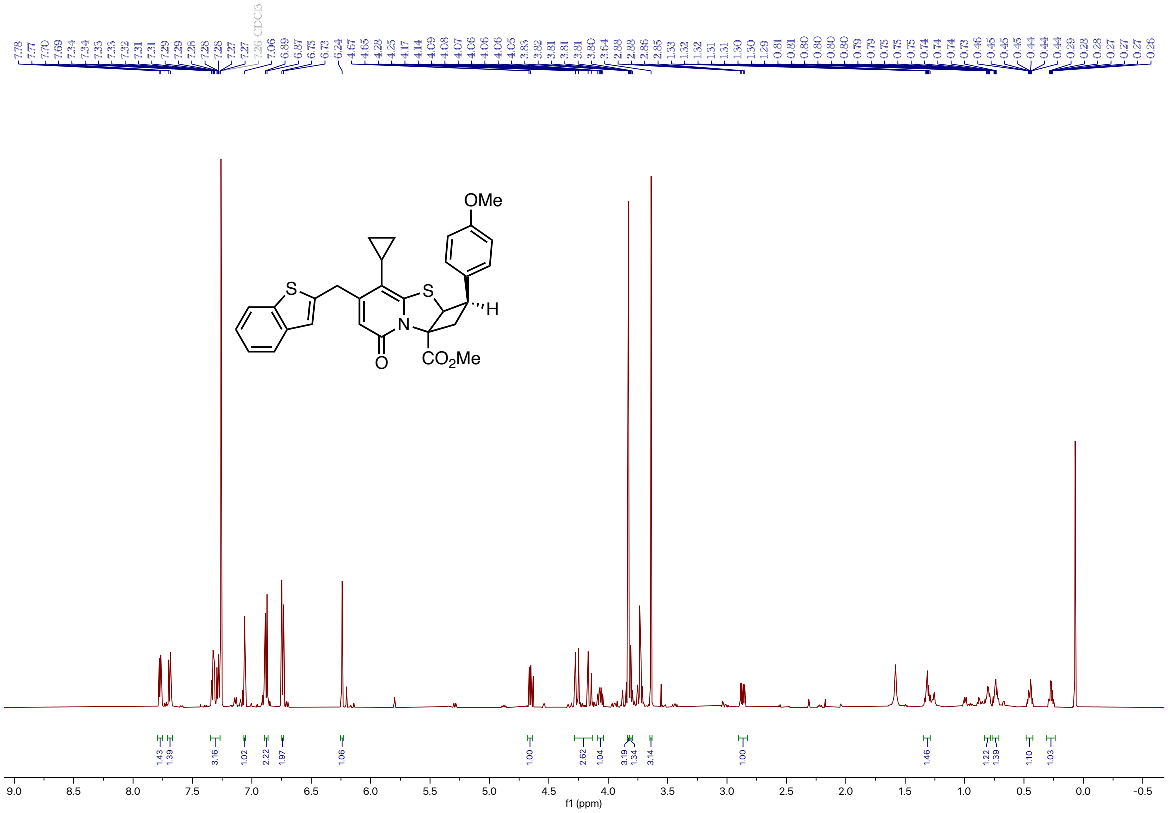

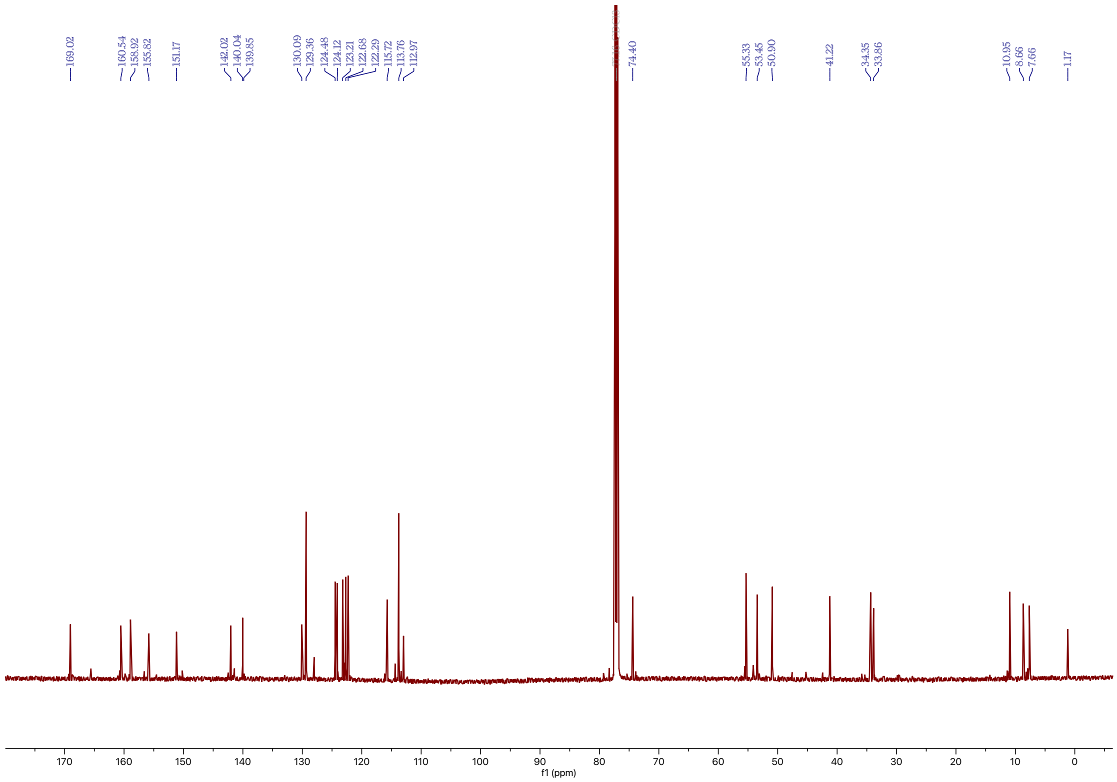

Compound **2l**, ^{1}H-NMR (600 MHz), ^{13}C-NMR (151 MHz) and ^{19}F NMR (565 MHz) ($CDCl_3$):

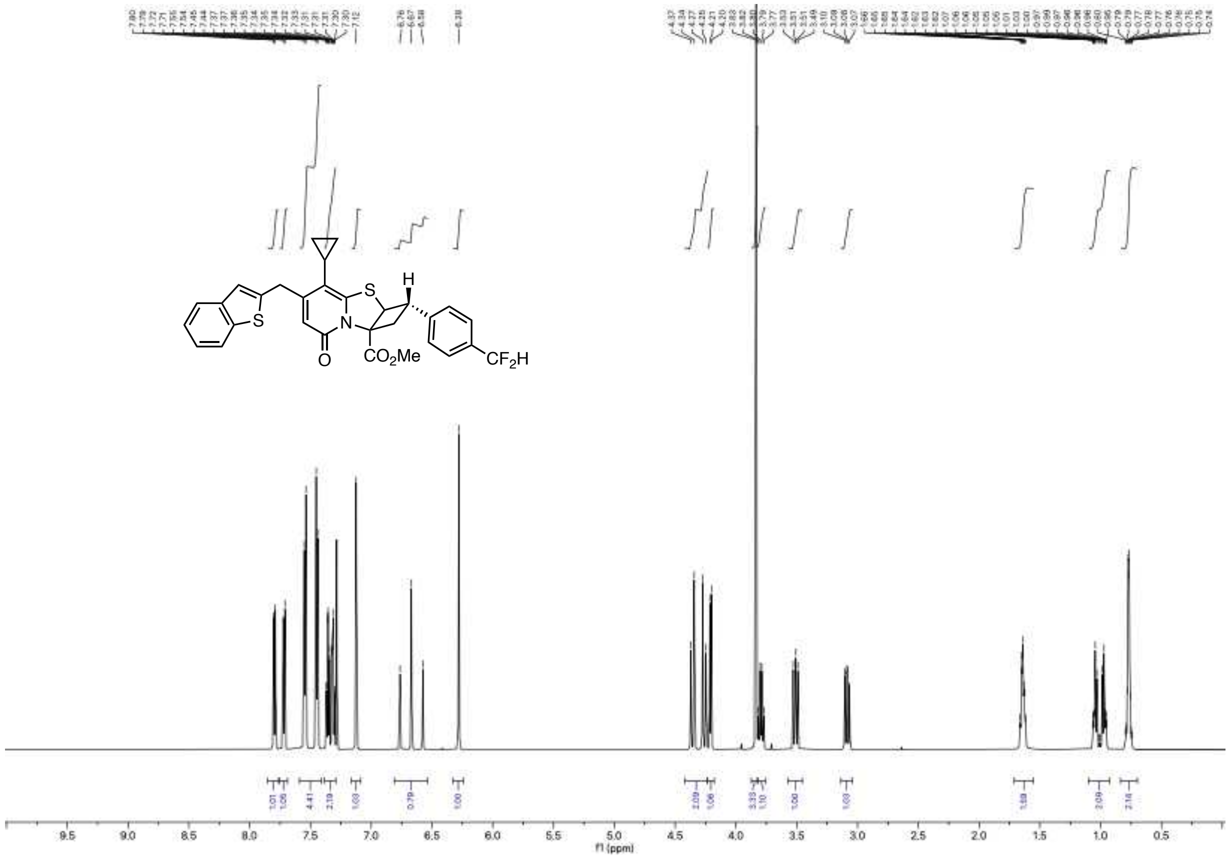

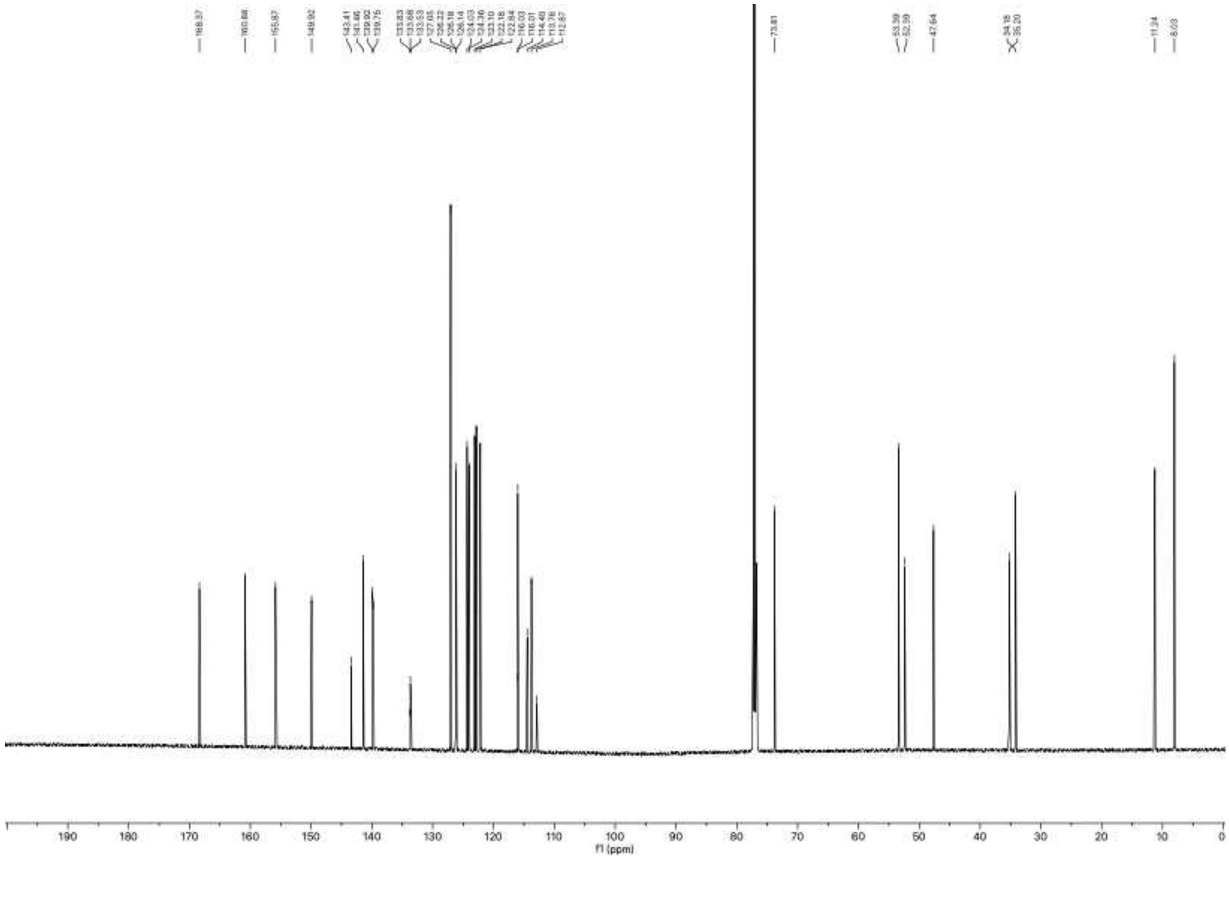

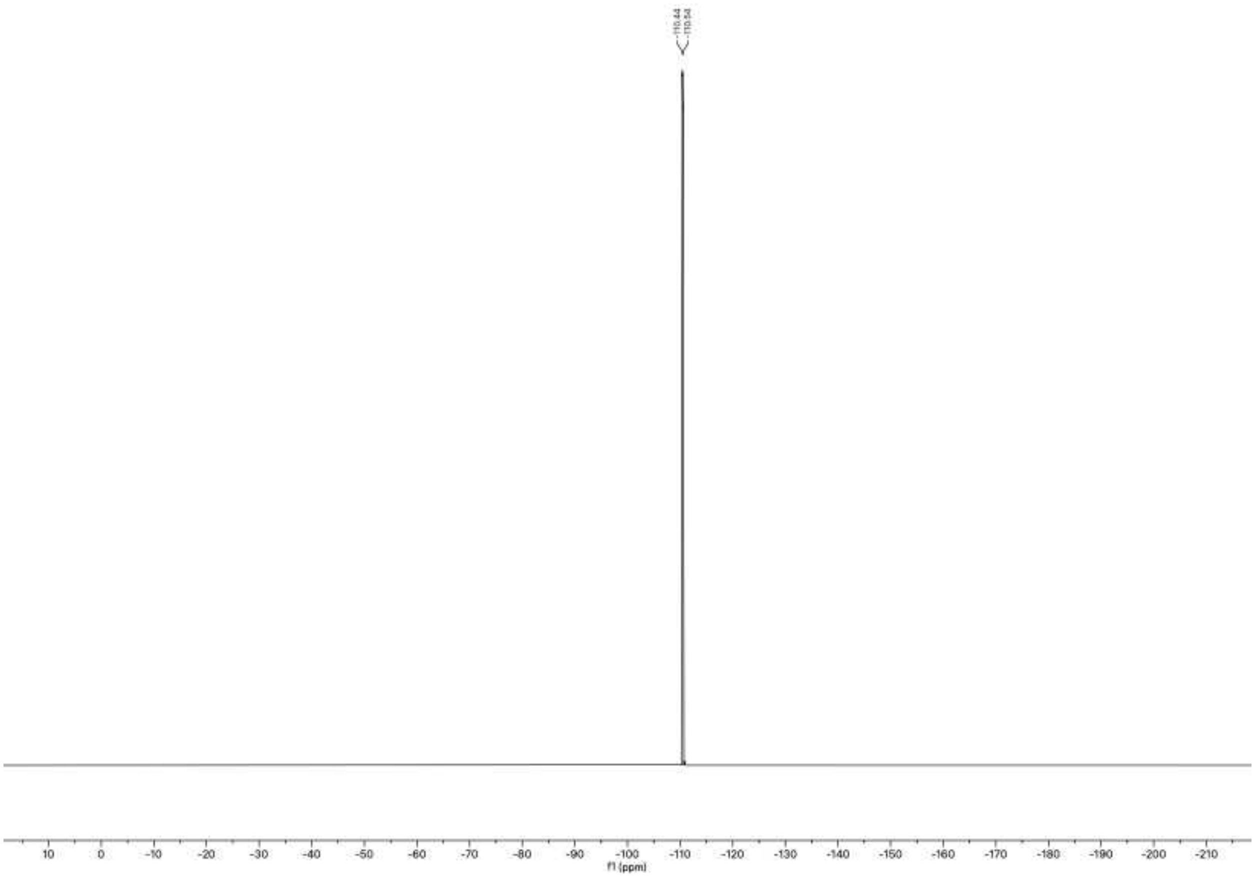
-110.44
-110.54
10
0
-10
-20
-30
-40
-50
-60
-70
-80
-90
-100
-110
-120
-130
-140
-150
-160
-170
-180
-190
-200
-210
f1 (ppm)

Compound **3l**, ^{1}H-NMR (600 MHz), ^{13}C-NMR (151 MHz) and ^{19}F NMR (565 MHz) ($CDCl_3$):

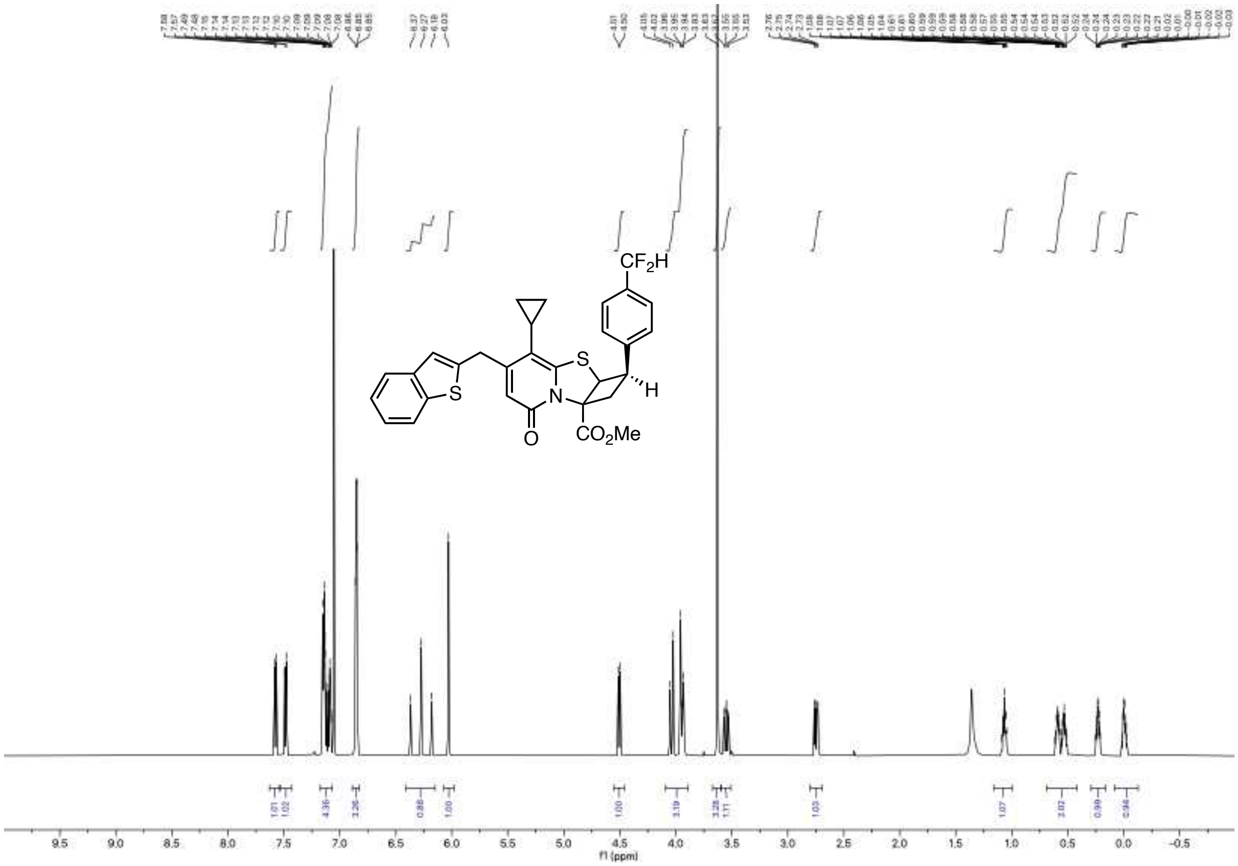

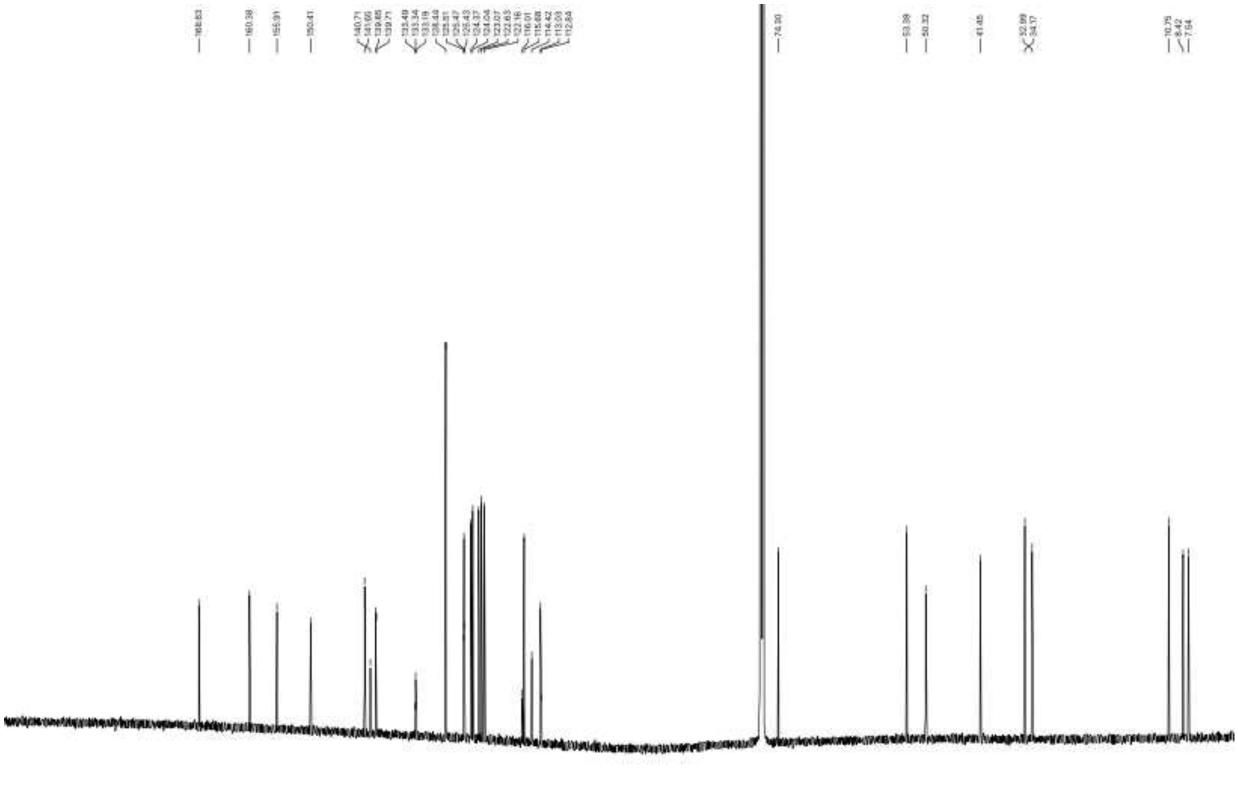

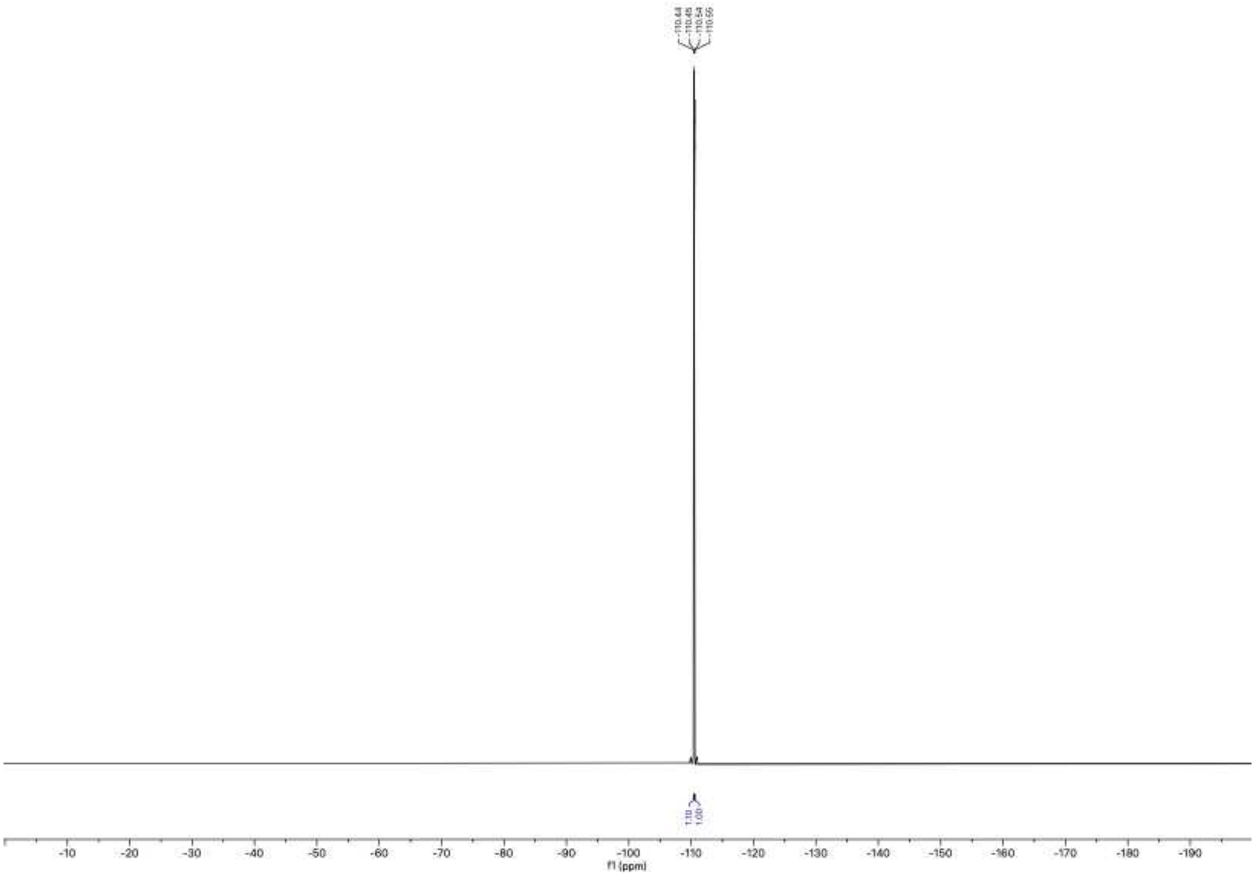

-110.44
-110.45
-110.54
-110.55
1.10
1.00
-10
-20
-30
-40
-50
-60
-70
-80
-90
-100
-110
-120
-130
-140
-150
-160
-170
-180
-190
f1 (ppm)

Compound **2m**, ^{1}H-NMR (400 MHz) and ^{13}C-NMR (151 MHz) ($CDCl_3$):

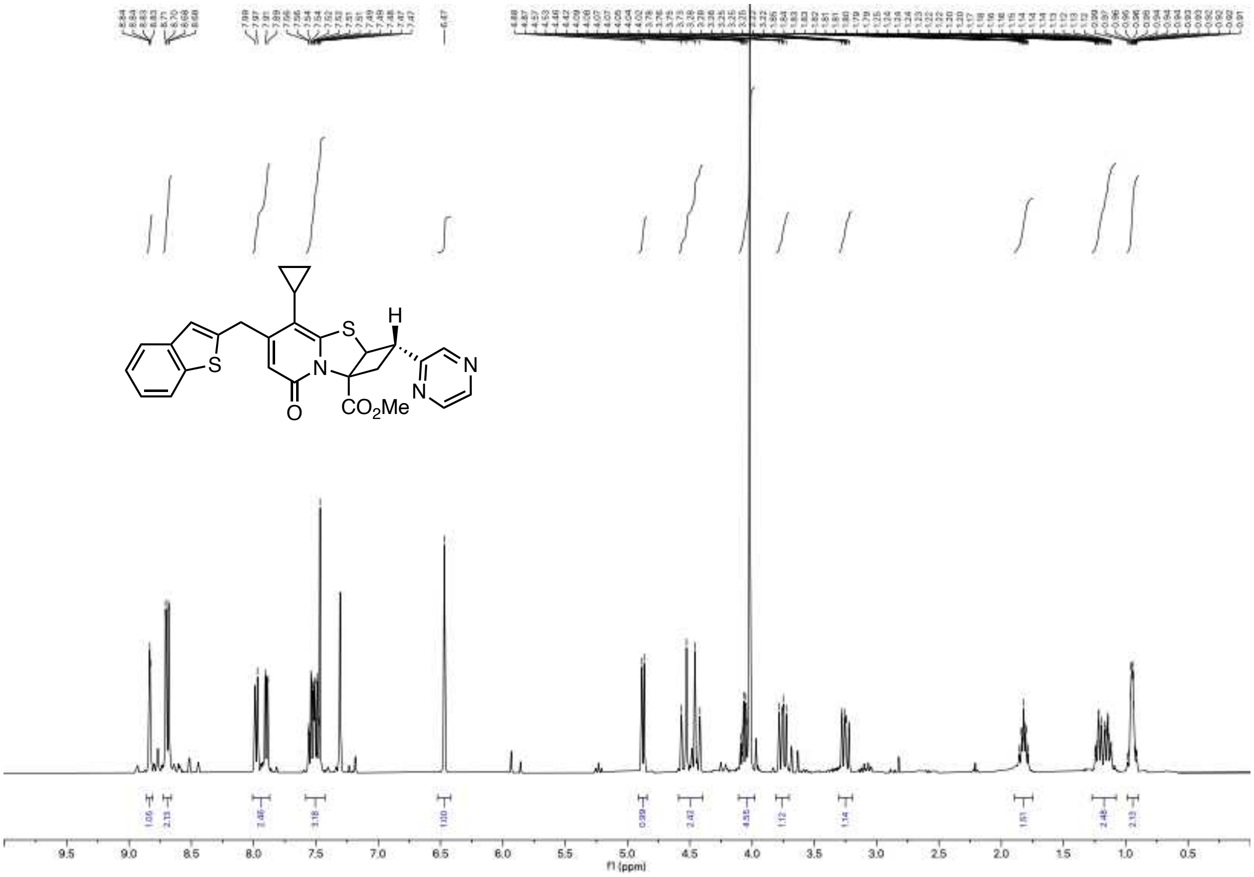

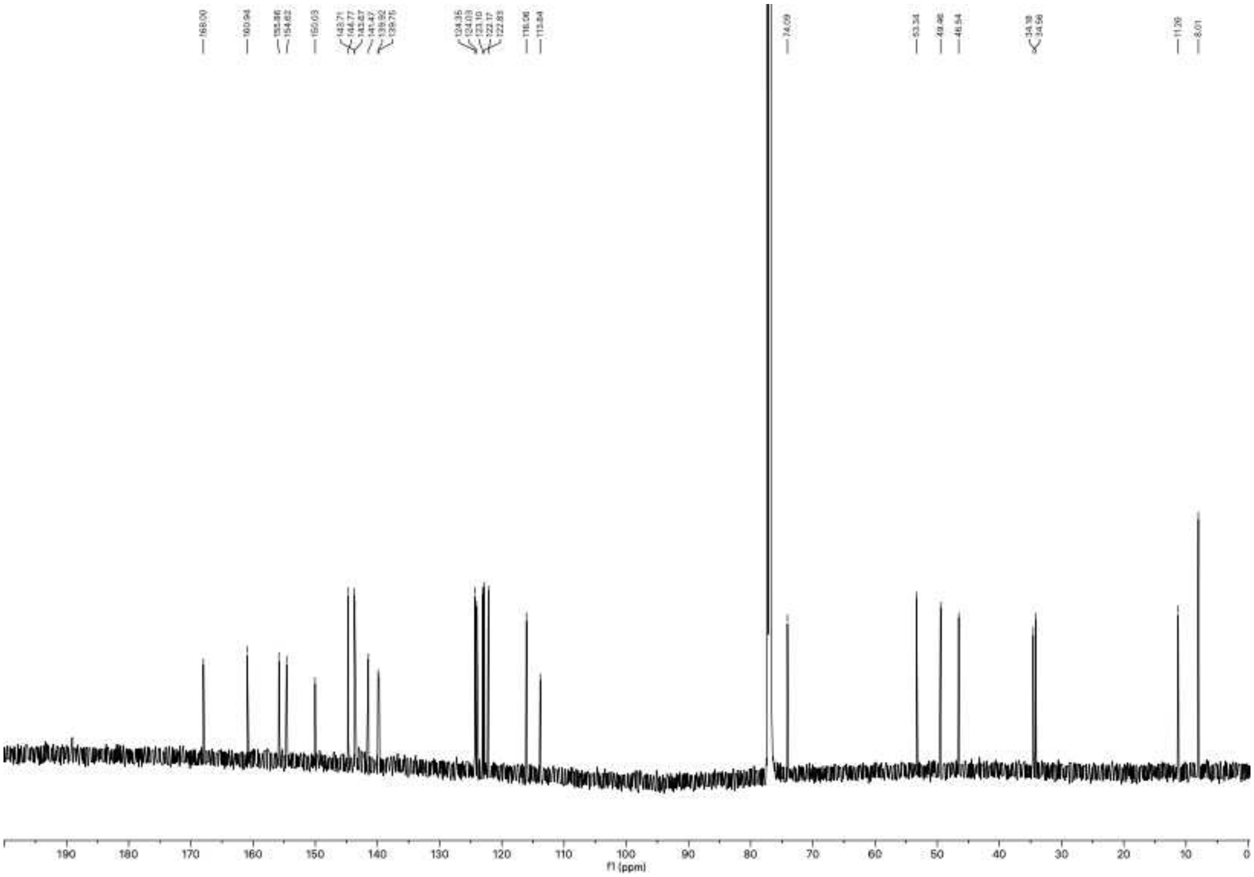

Compound **2n**, ^{1}H-NMR (400 MHz) and ^{13}C-NMR (151 MHz) ($CDCl_3$):

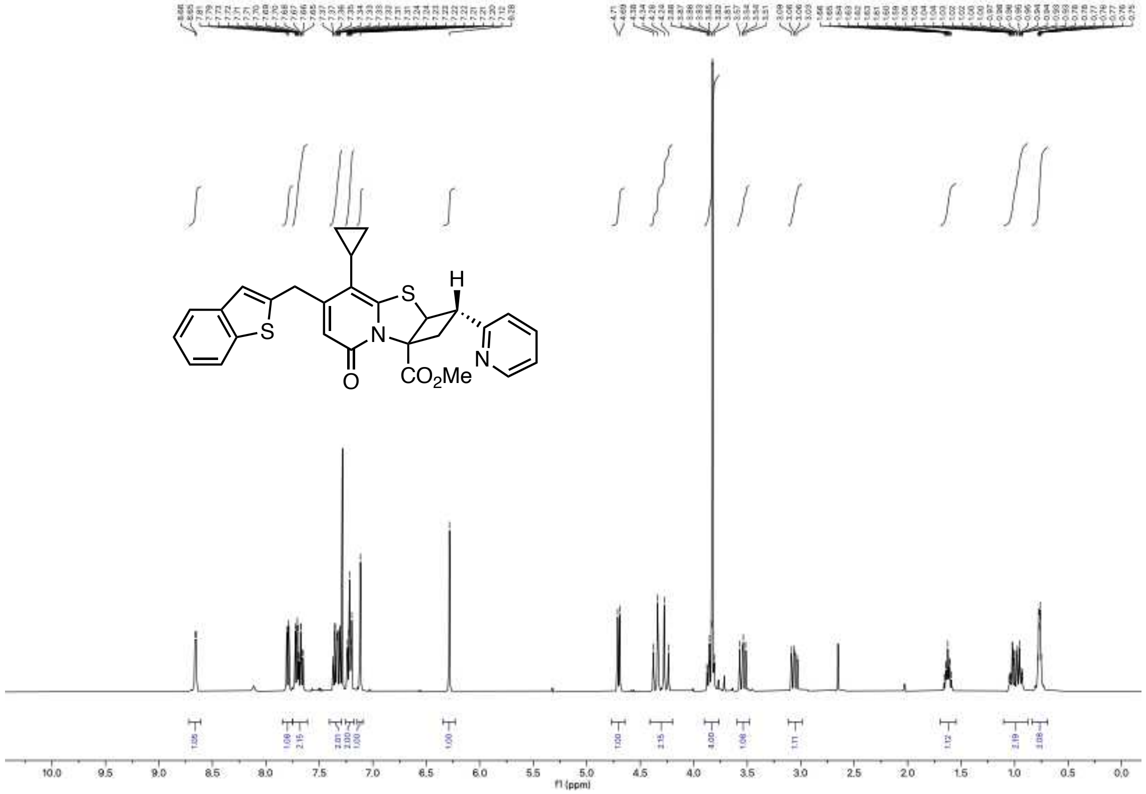

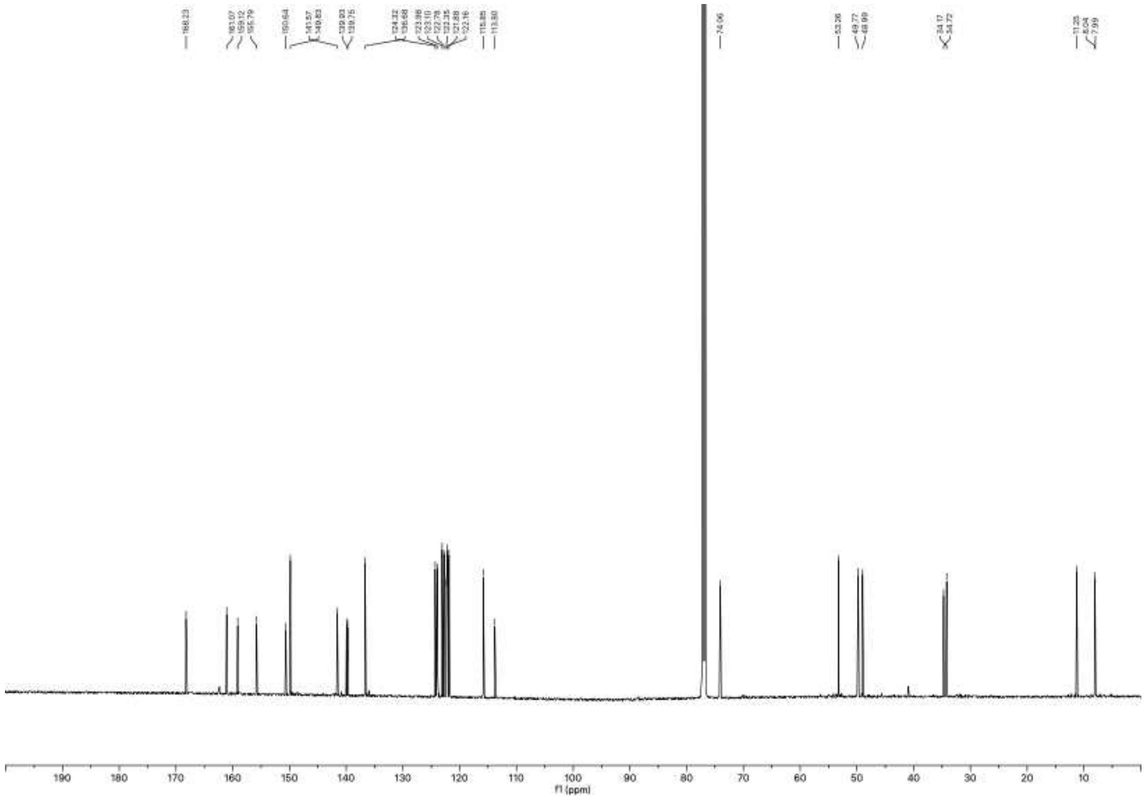

Compound **3n**, ^{1}H-NMR (400 MHz) and ^{13}C-NMR (151 MHz) ($CDCl_3$):

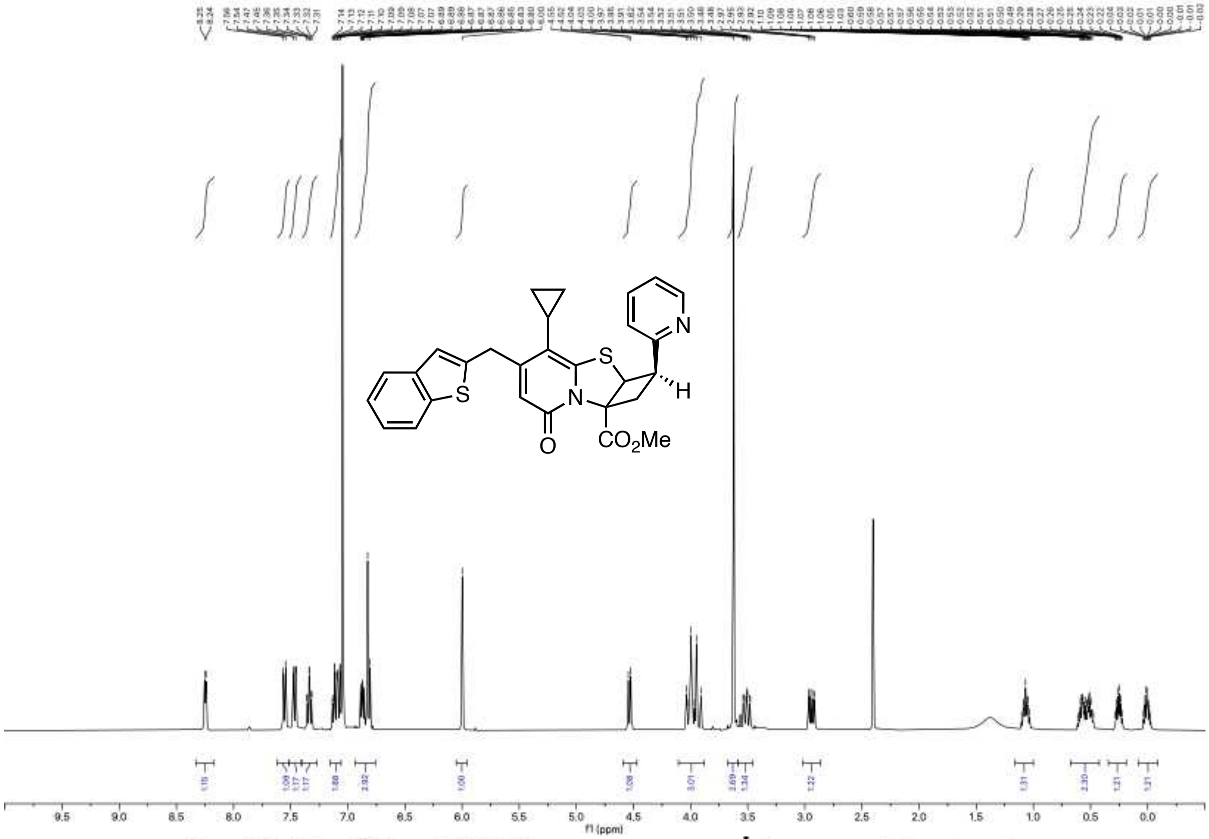

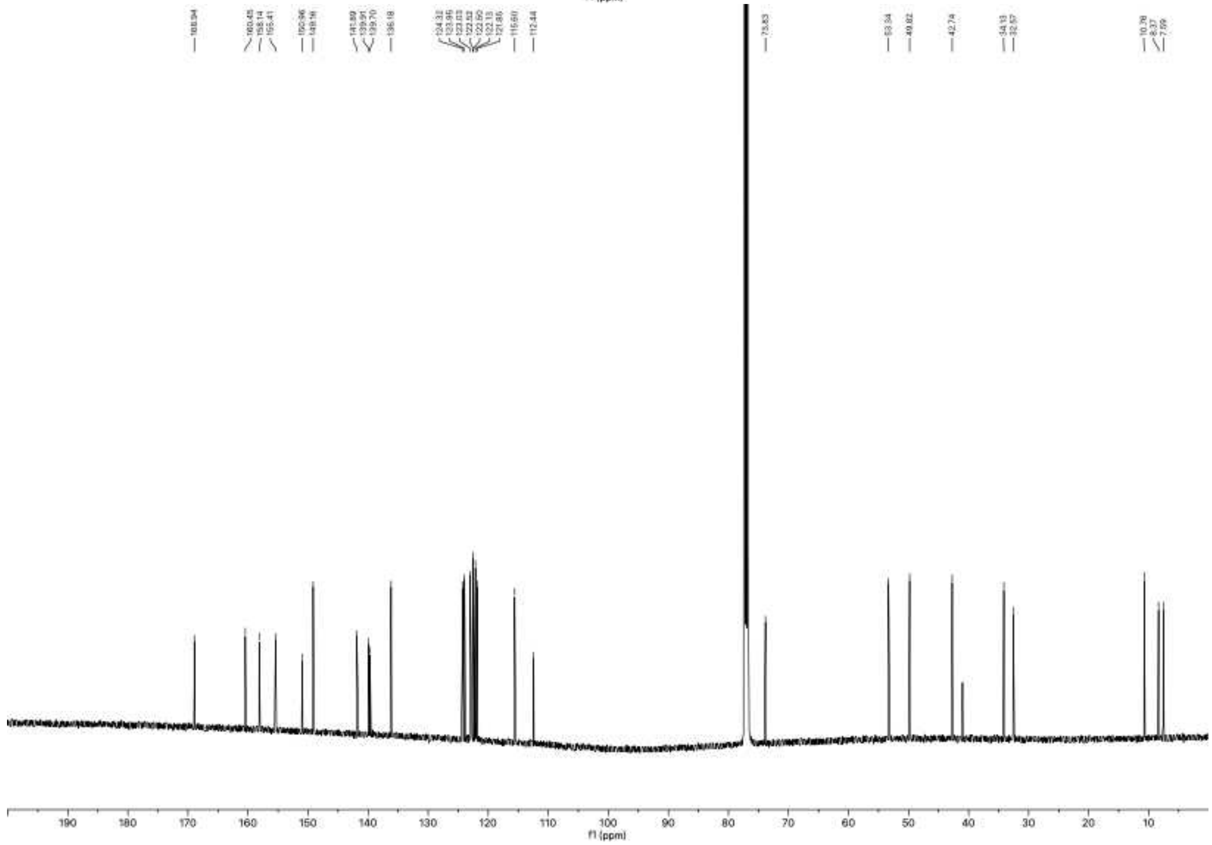

Compound **IL262**, ^{1}H-NMR (600 MHz) and ^{13}C-NMR (151 MHz) ($(CD_3)_2SO$):

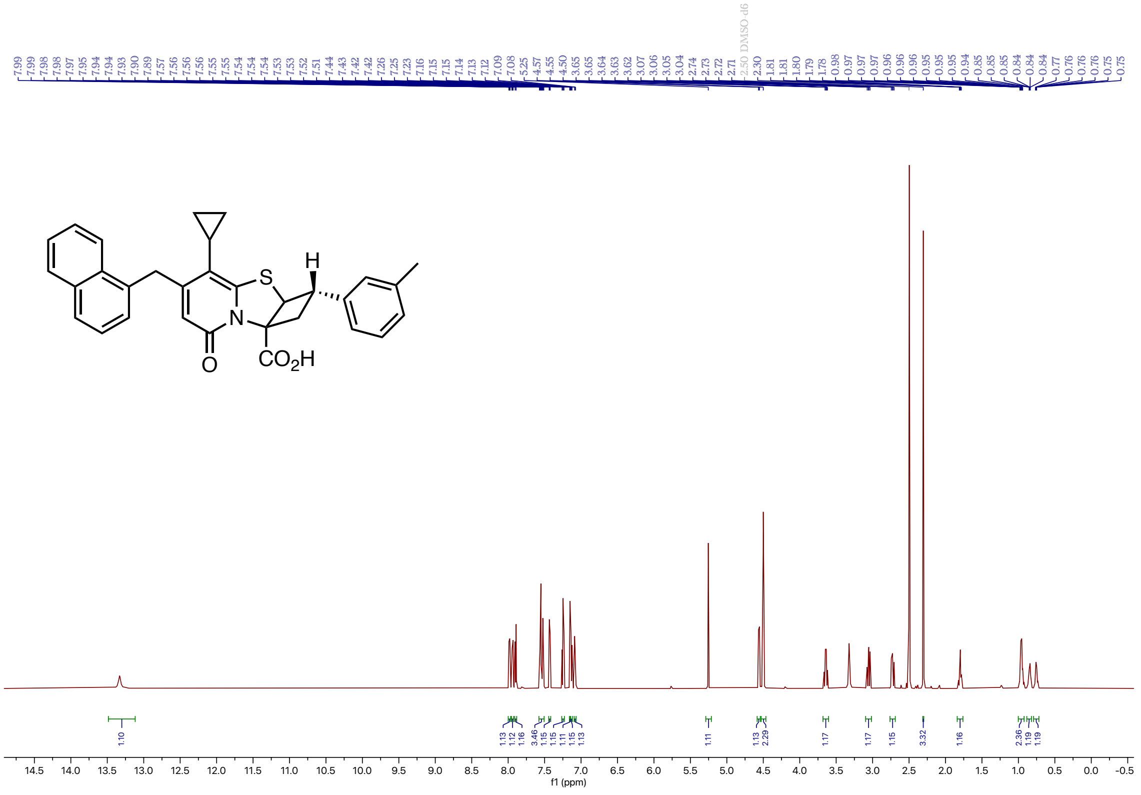

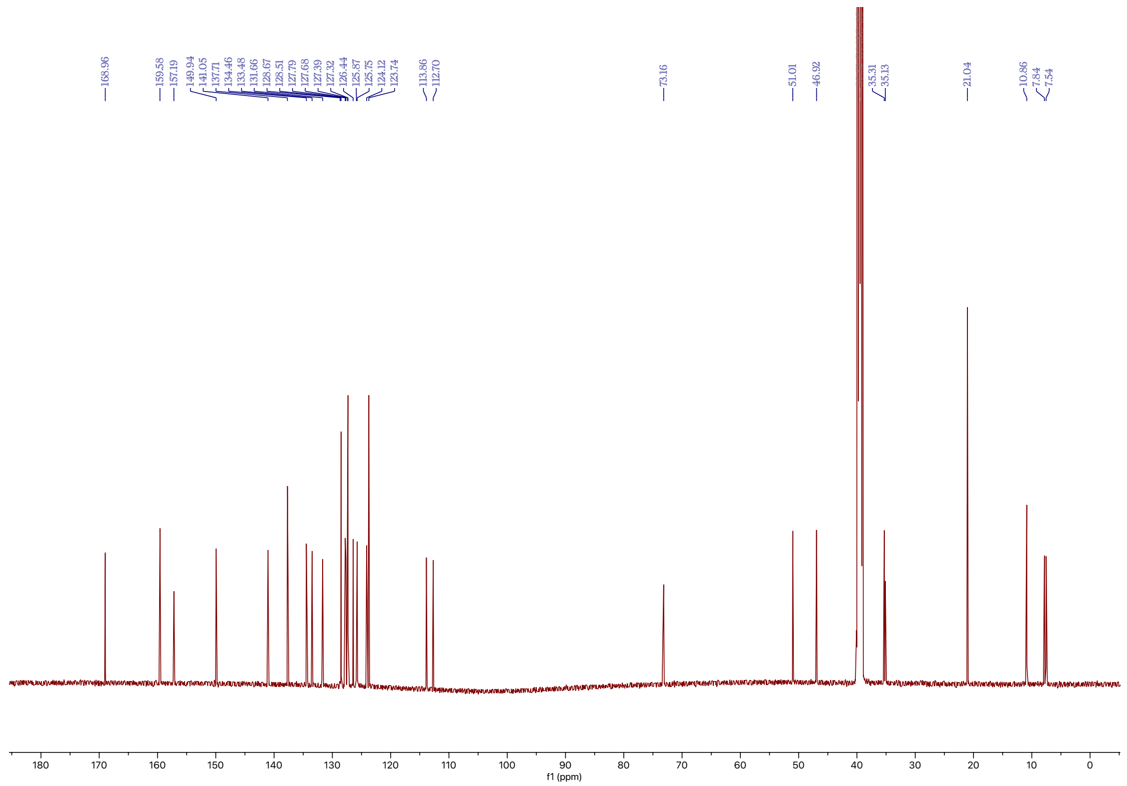

Compound **IL263**, ^{1}H-NMR (600 MHz) and ^{13}C-NMR (151 MHz) (($CD_3)_2SO$):

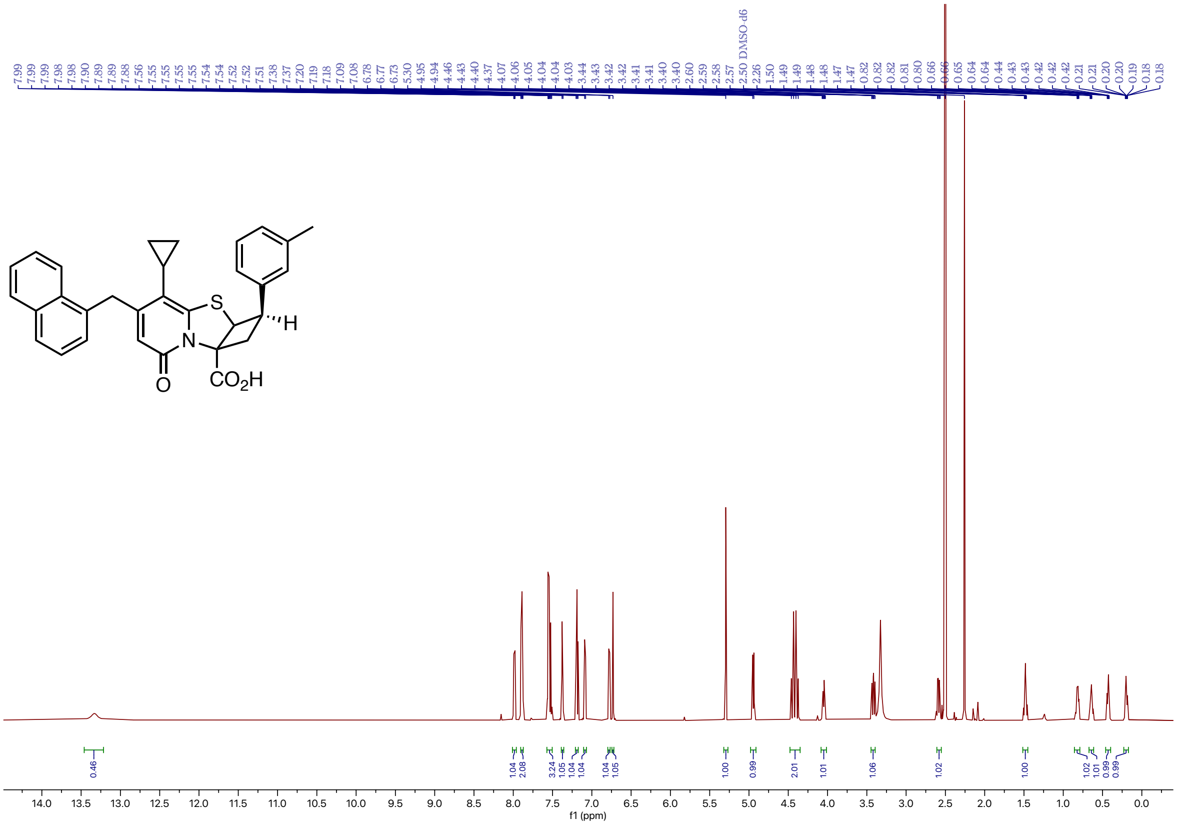

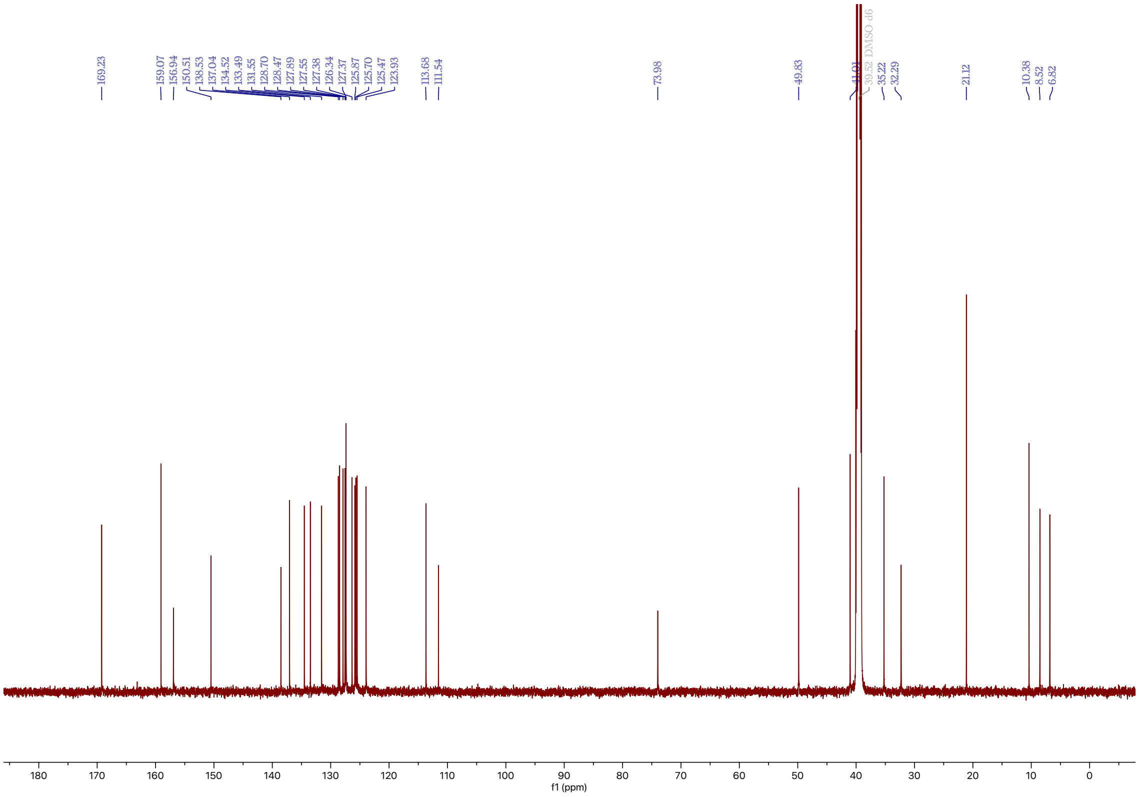

Compound **NQA8**, ^{1}H-NMR (600 MHz) and ^{13}C-NMR (151 MHz) ((CD_3)$_2$SO):

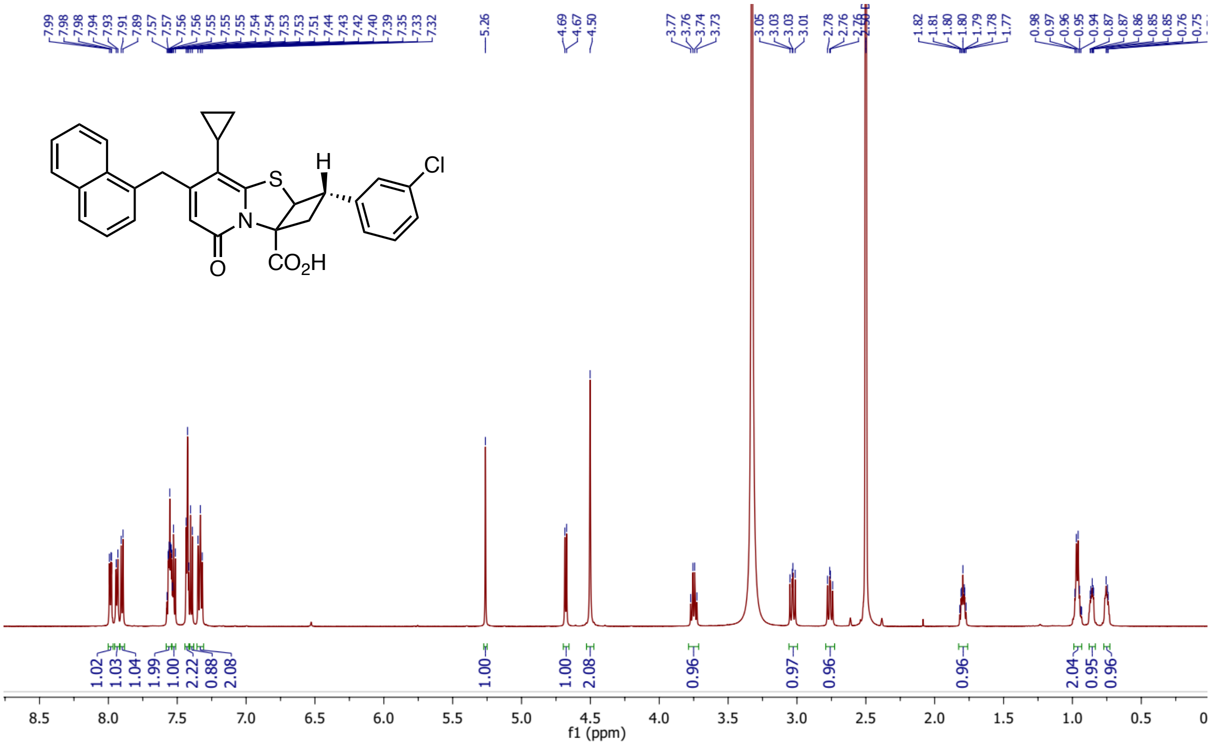

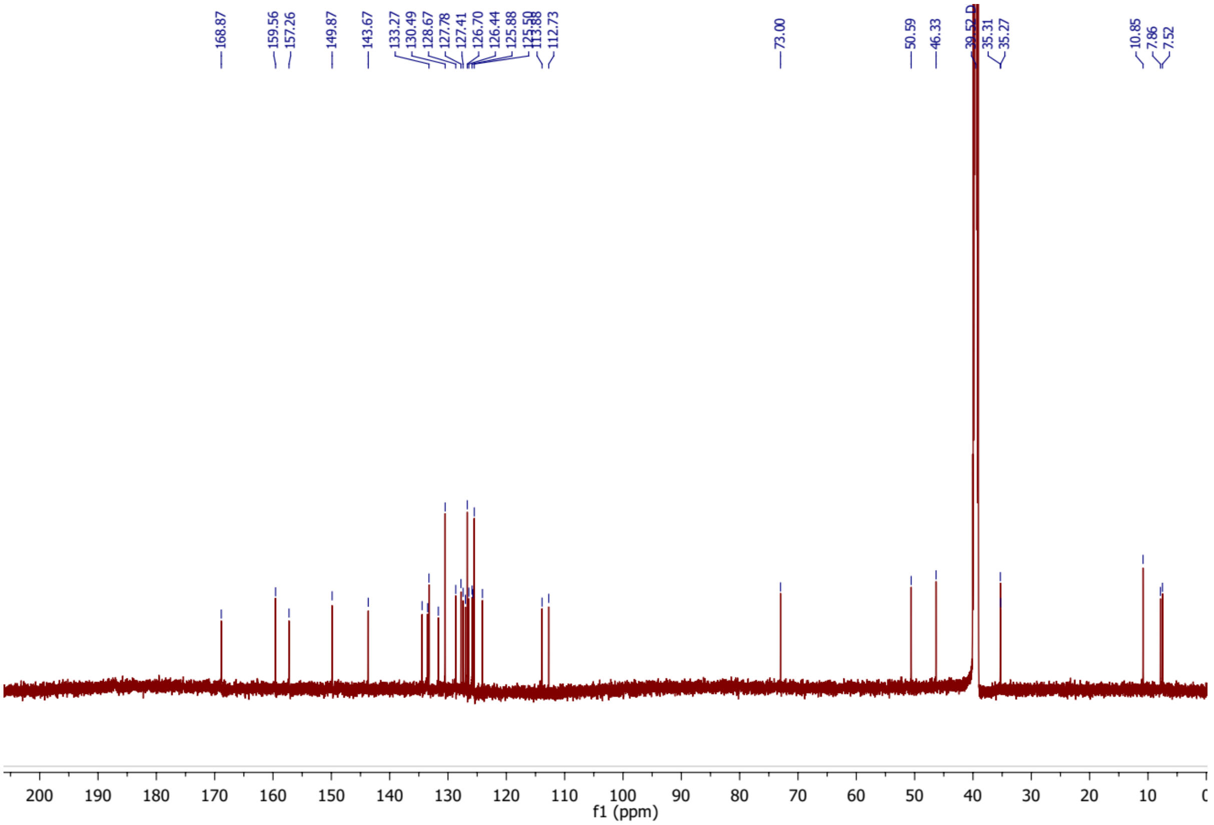

Compound **NQA9**, ^{1}H-NMR (600 MHz) and ^{13}C-NMR (151 MHz) ($(CD_3)_2SO$):

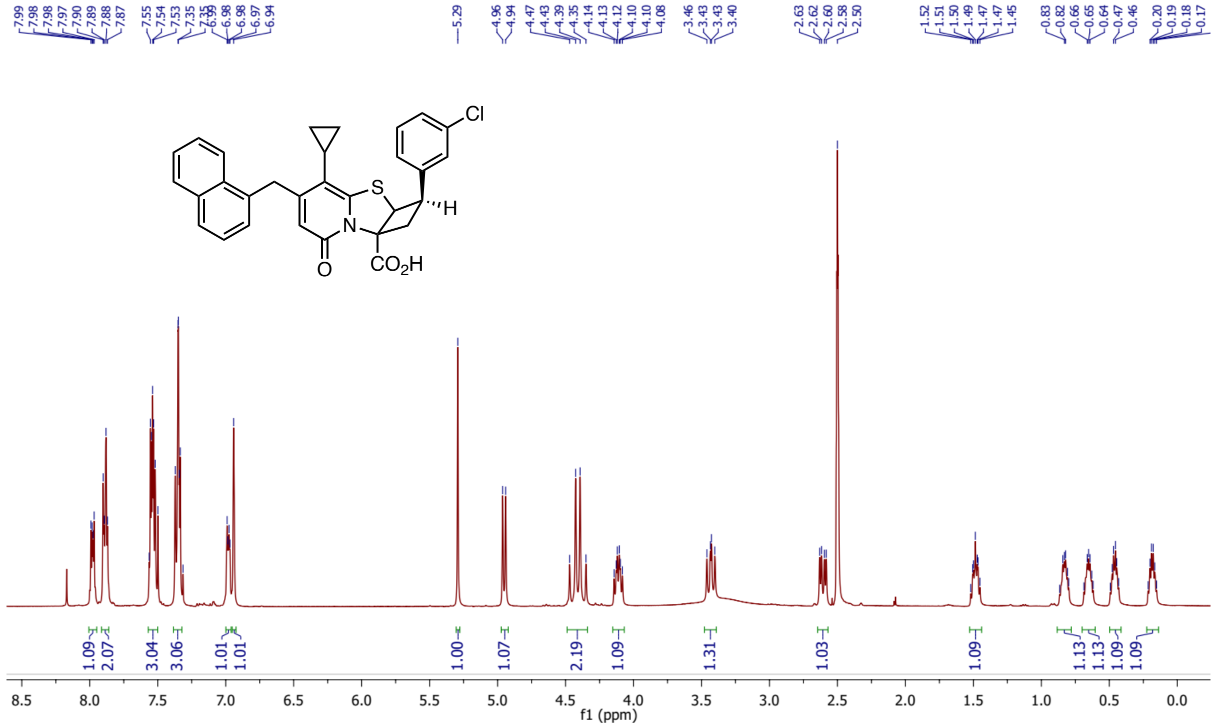

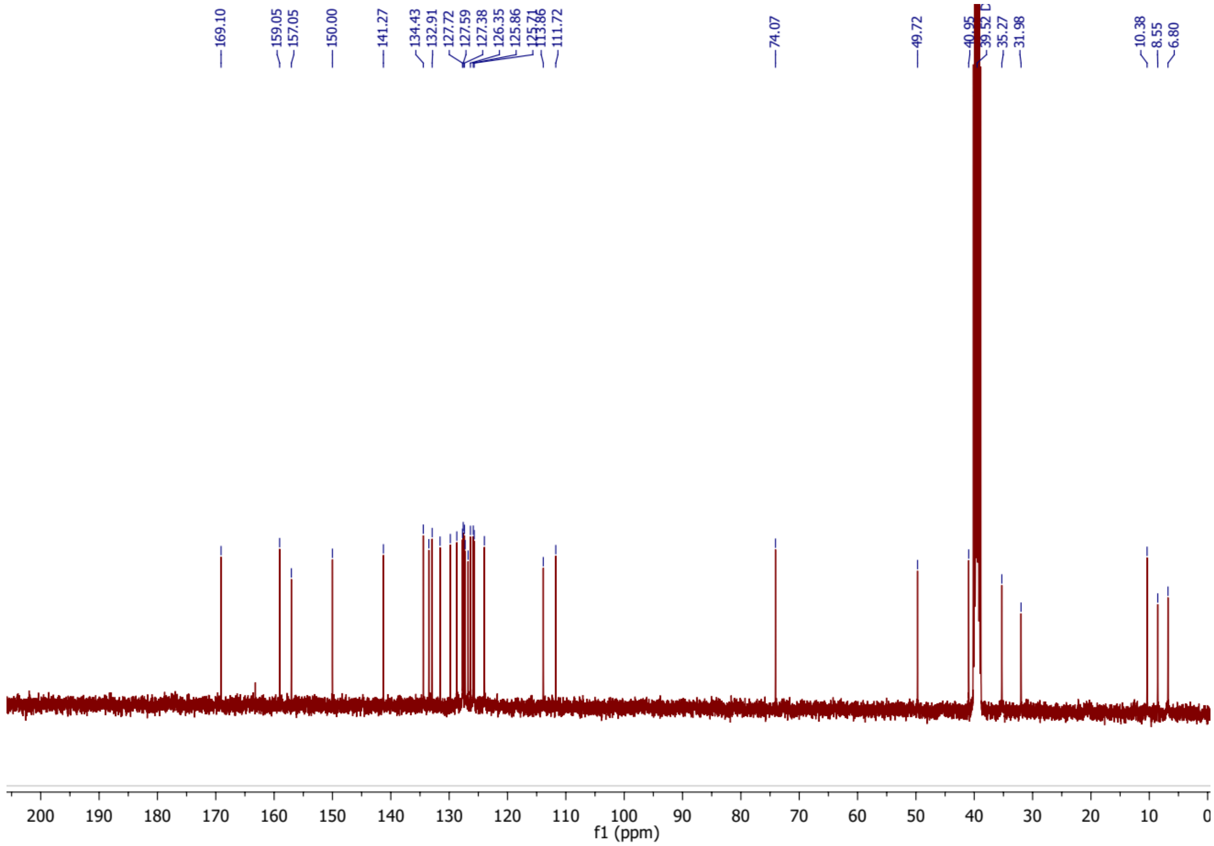

Compound **IL2305**, ^{1}H-NMR (600 MHz) and ^{13}C-NMR (151 MHz) ($(CD_3)_2SO$):

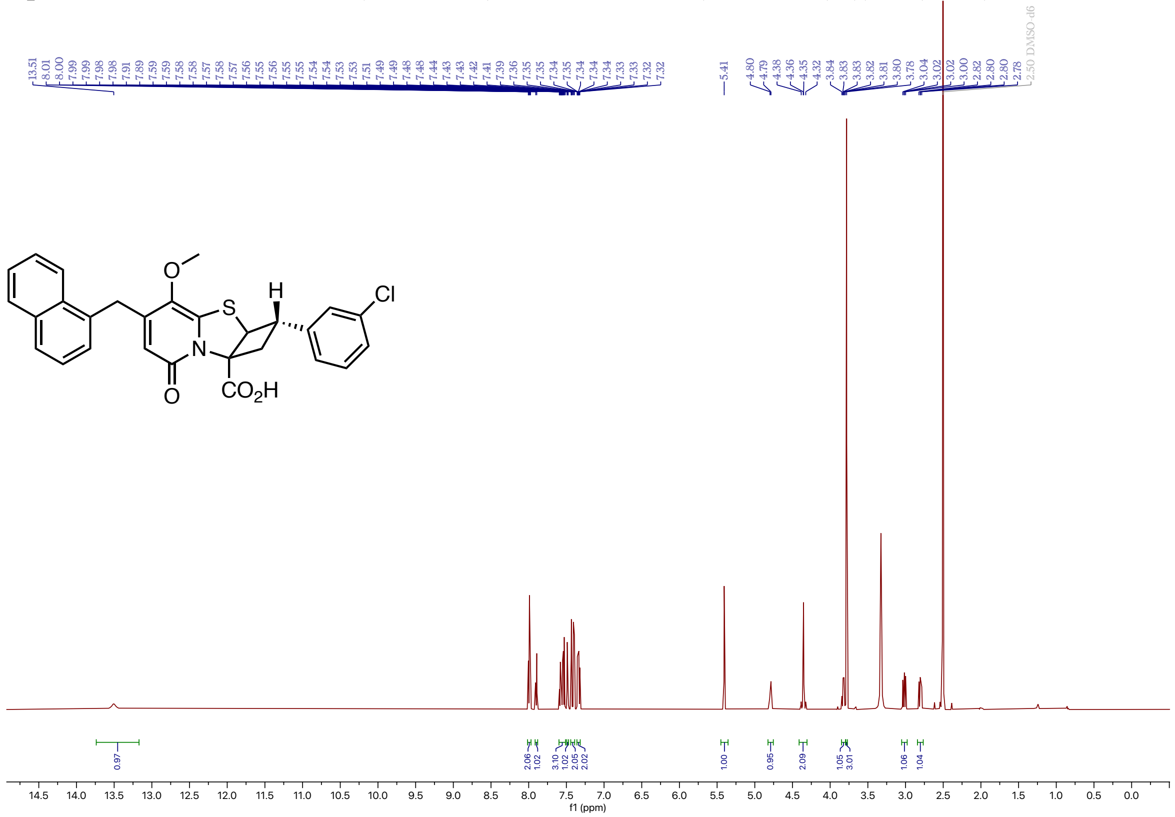

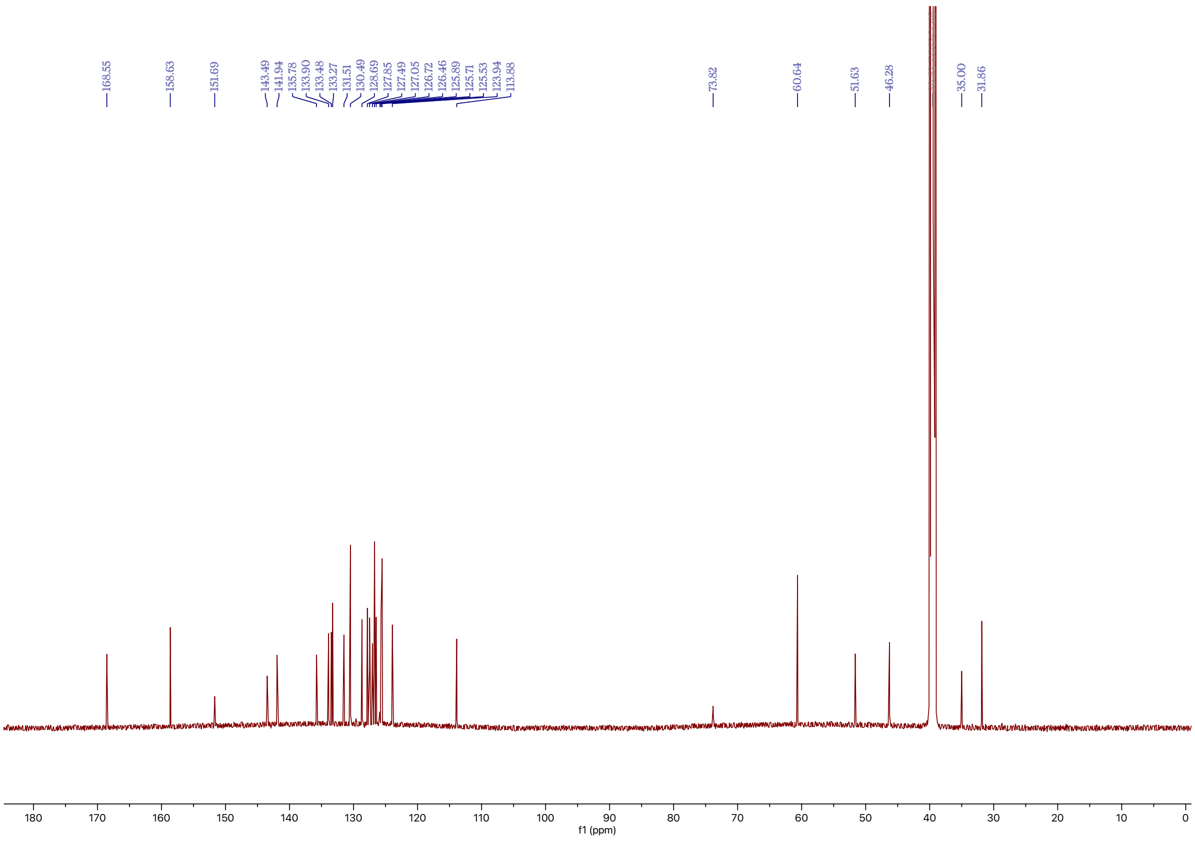

Compound **PS1961**, ^{1}H-NMR (600 MHz) and ^{13}C-NMR (151 MHz) ($(CD_3)_2SO$):

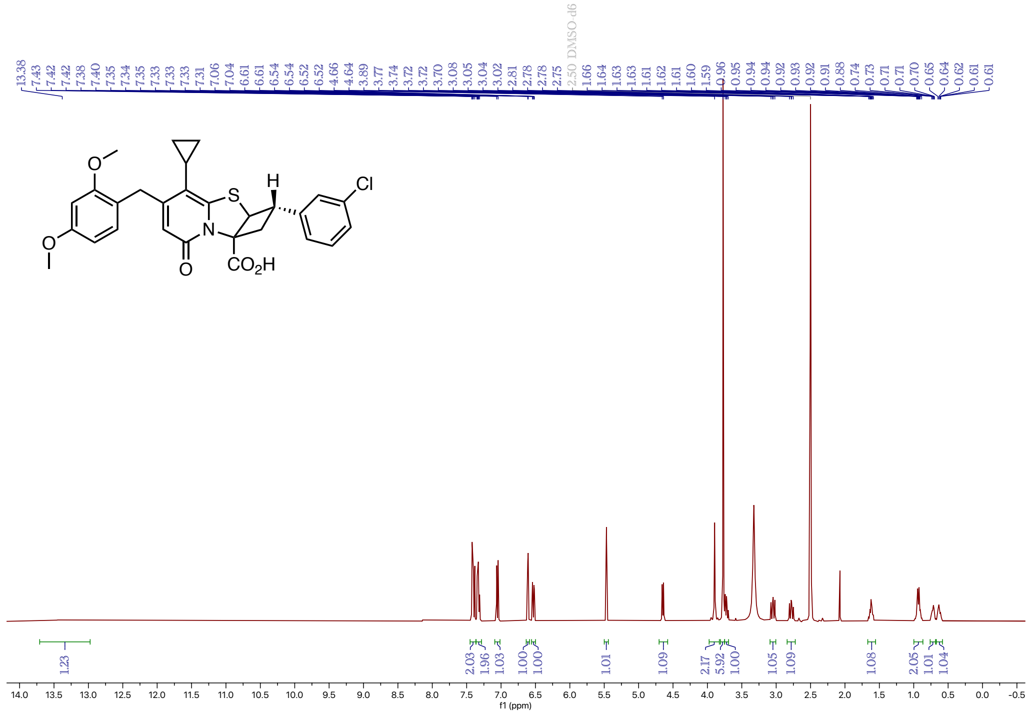

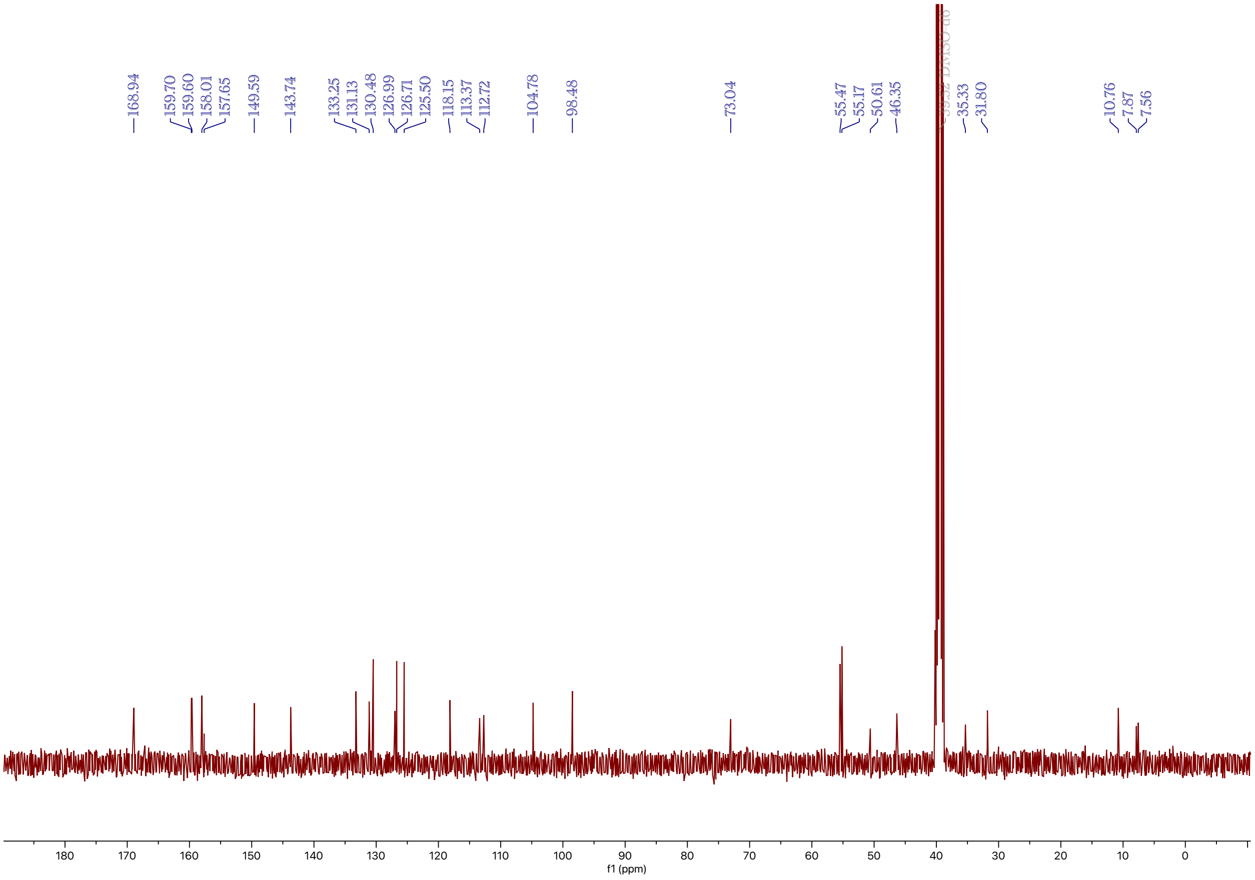

Compound **PS1965**, ^{1}H-NMR (600 MHz) and ^{13}C-NMR (151 MHz) ($(CD_3)_2SO$):

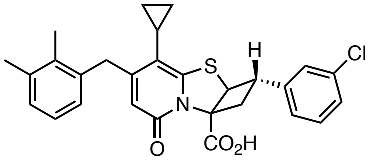

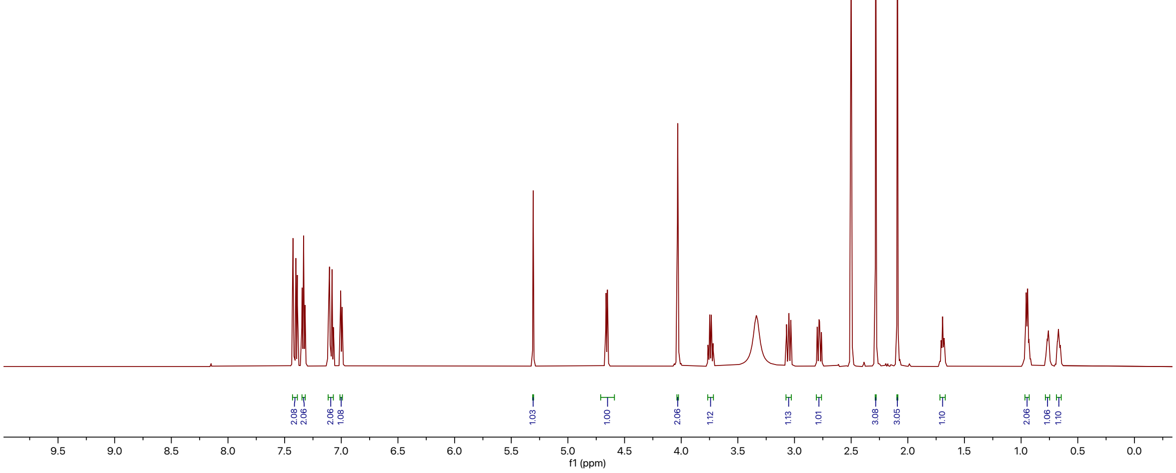

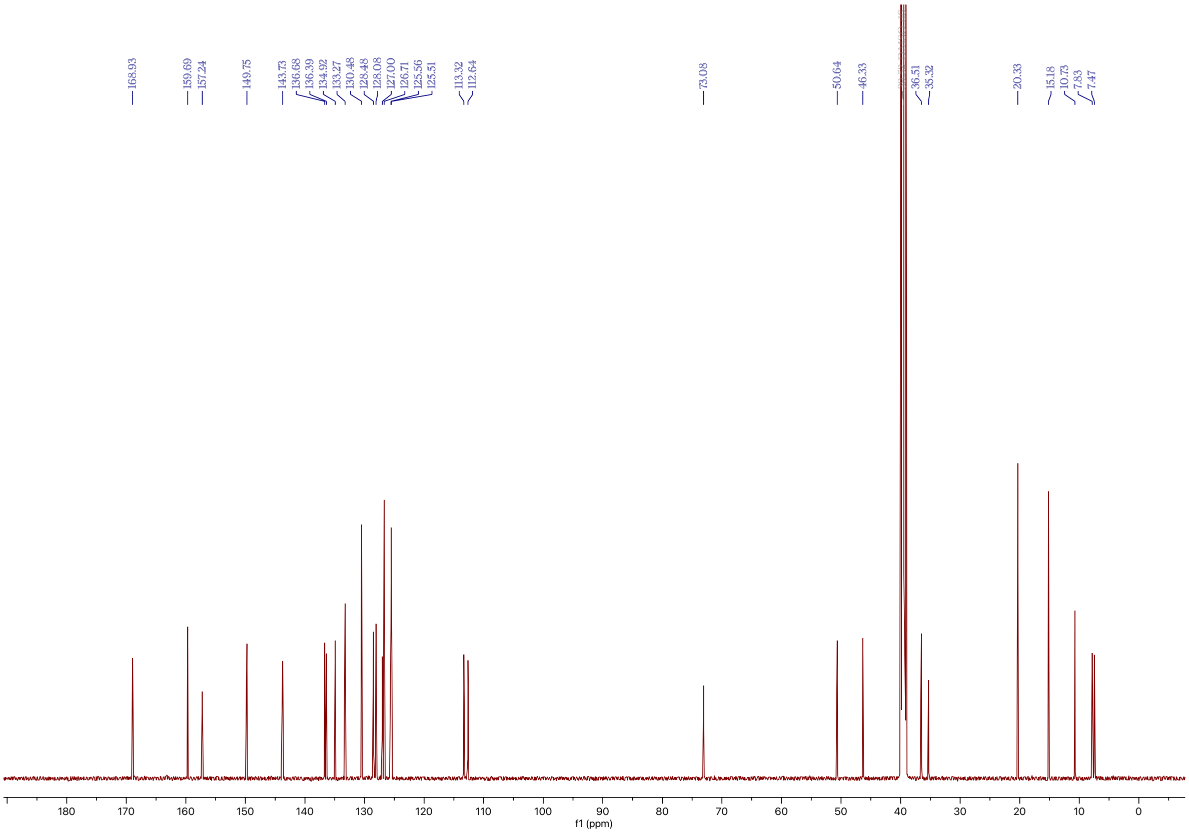

Compound **PS1970**, ^{1}H-NMR (600 MHz) and ^{13}C-NMR (151 MHz) ($(CD_3)_2SO$):

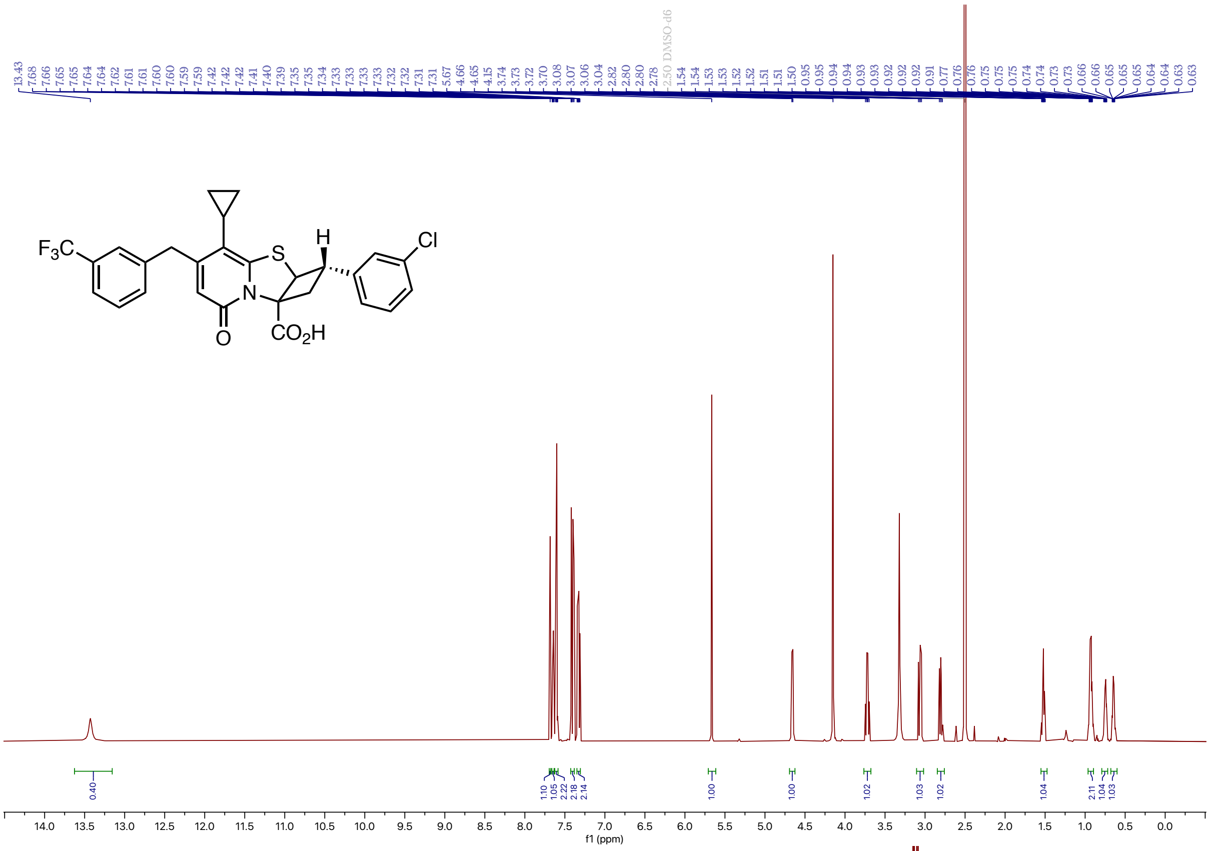

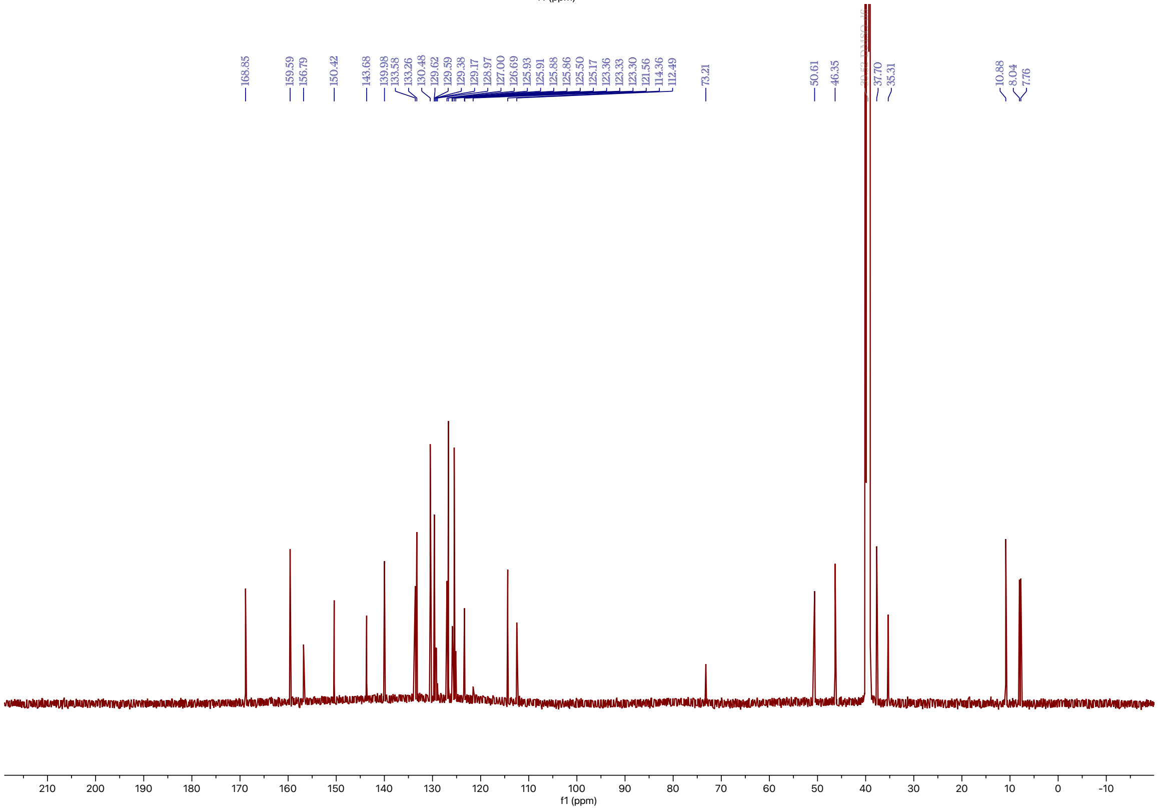

Compound **PS1970**, ^{19}F-NMR (565 MHz) ((CD_3)$_2$SO):

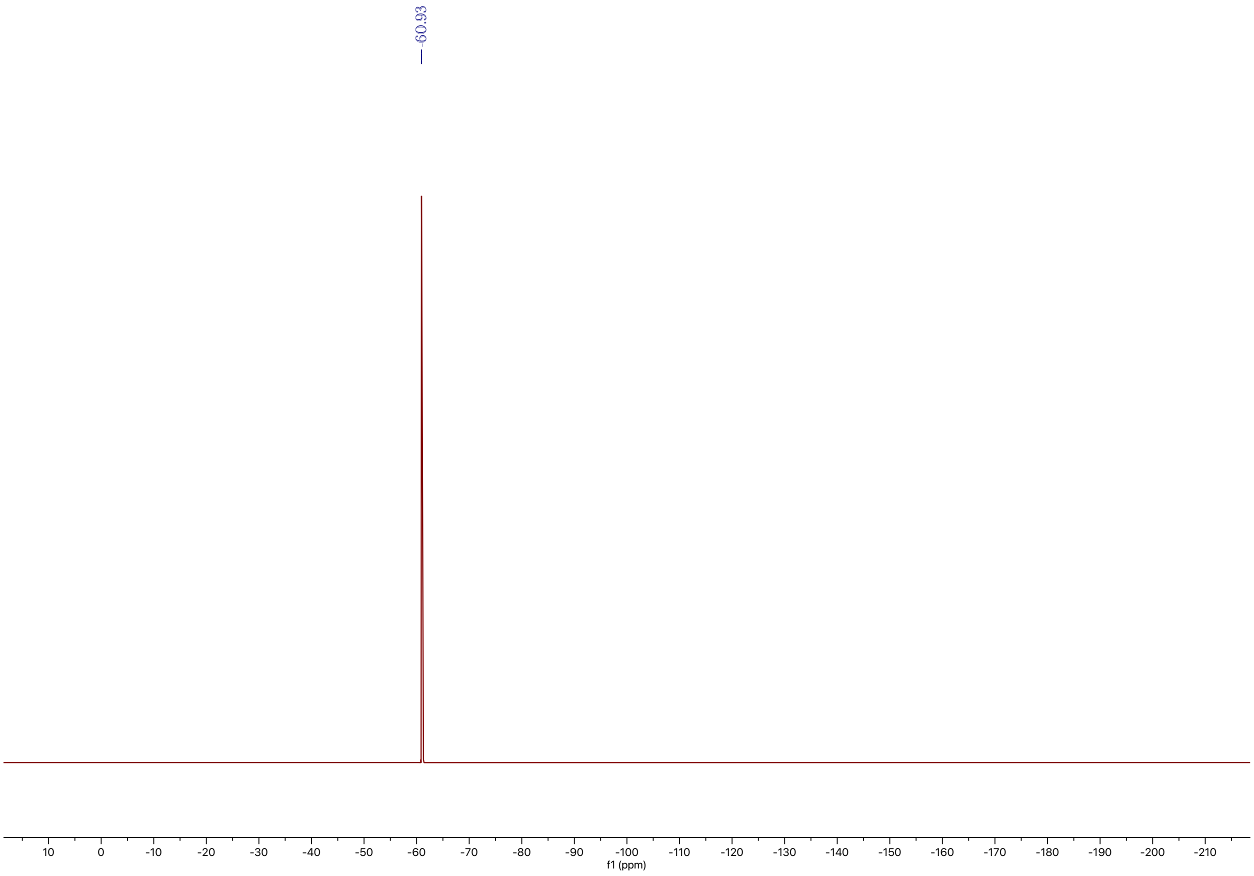

Compound **PS1962**, ^{1}H-NMR (600 MHz) and ^{13}C-NMR (151 MHz) ($(CD_3)_2SO$):

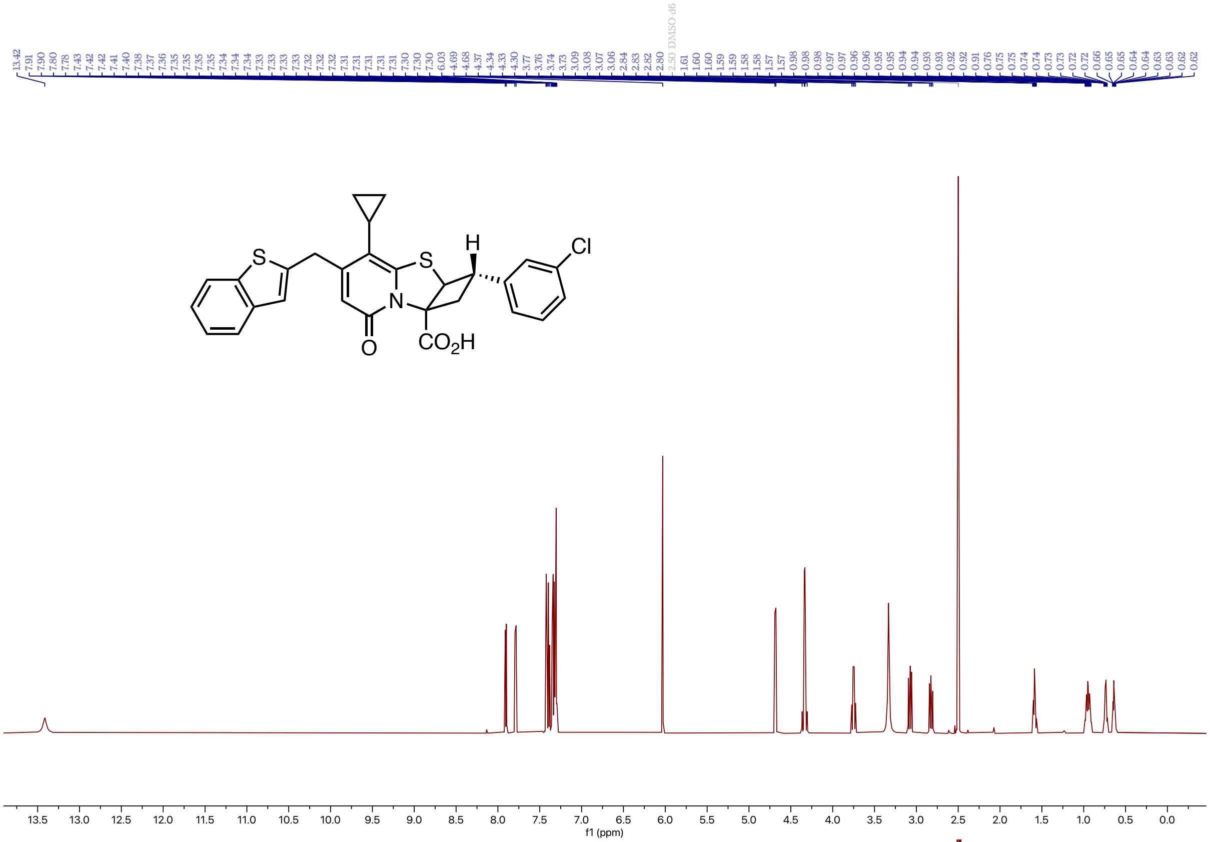

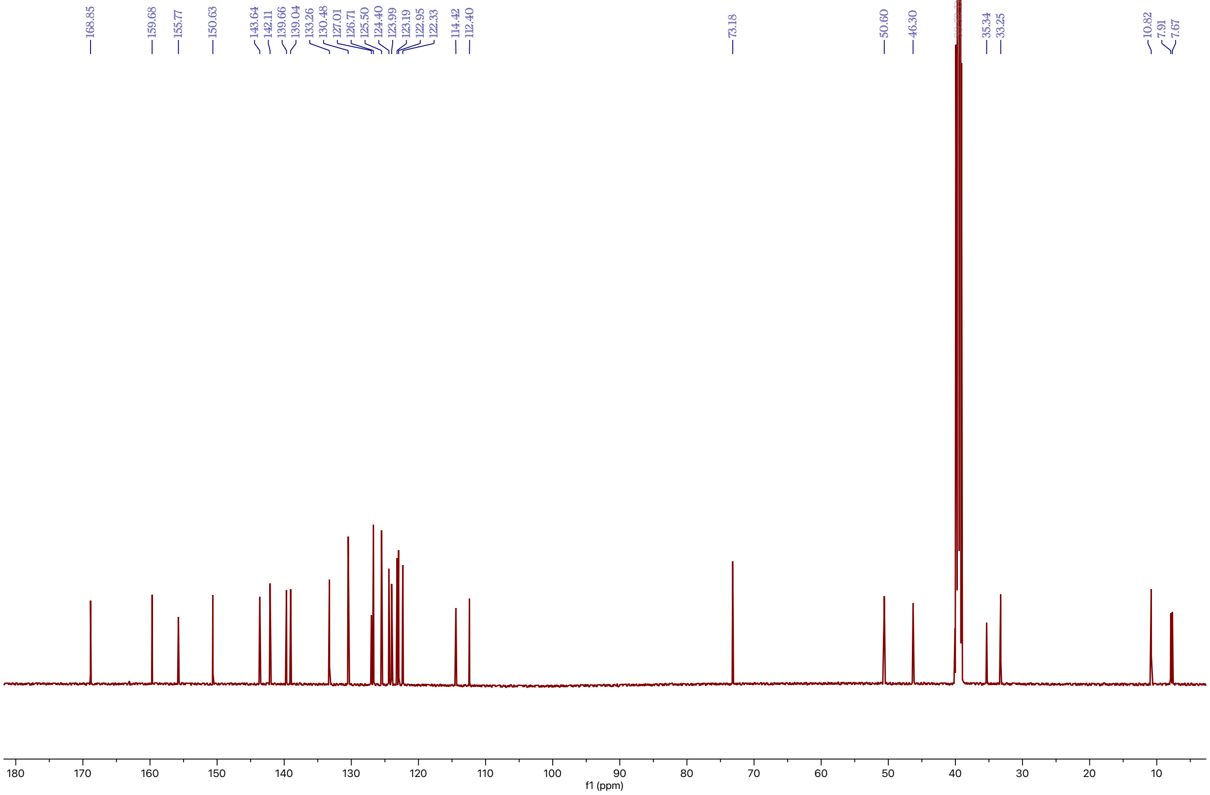

Compound **PS1963**, ^{1}H-NMR (600 MHz) and ^{13}C-NMR (151 MHz) ($(CD_3)_2SO$):

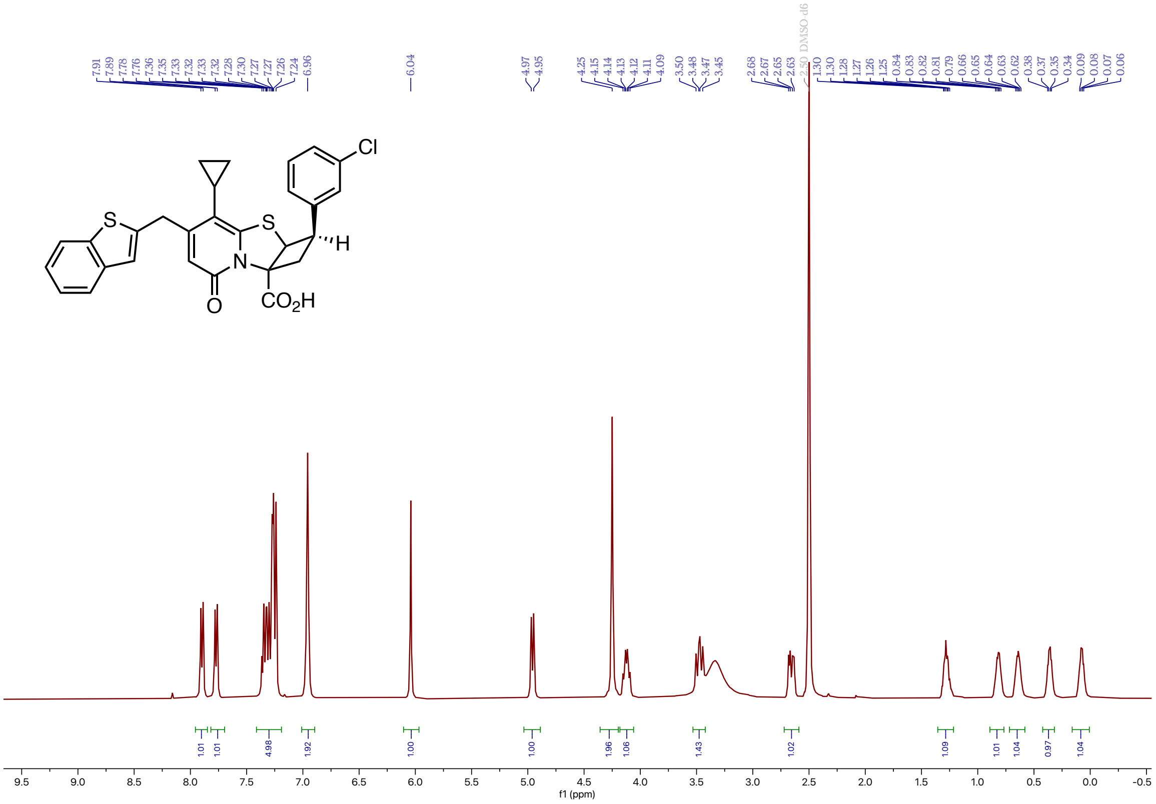

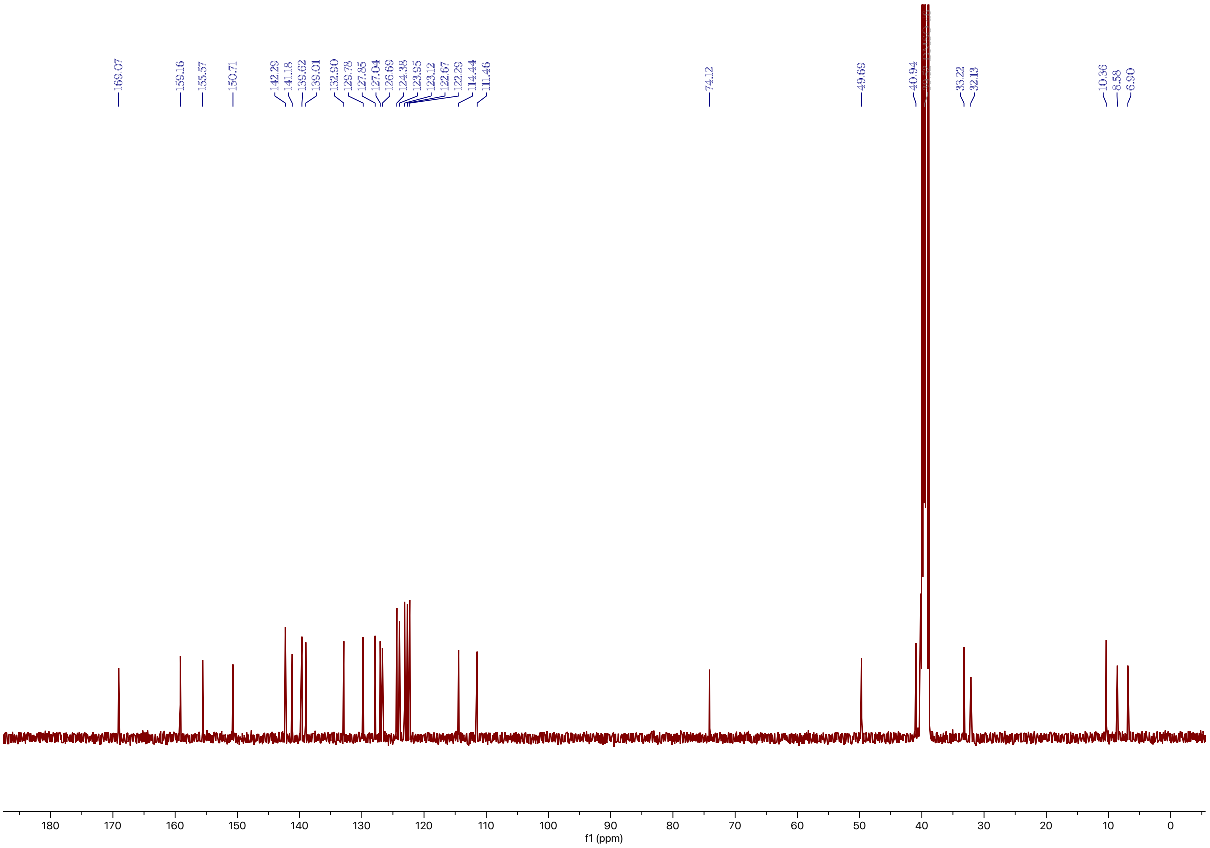

Compound **SS1040B**, ^{1}H-NMR (600 MHz), ^{13}C-NMR (151 MHz) and ^{19}F NMR (565 MHz) $CDCl_3$):

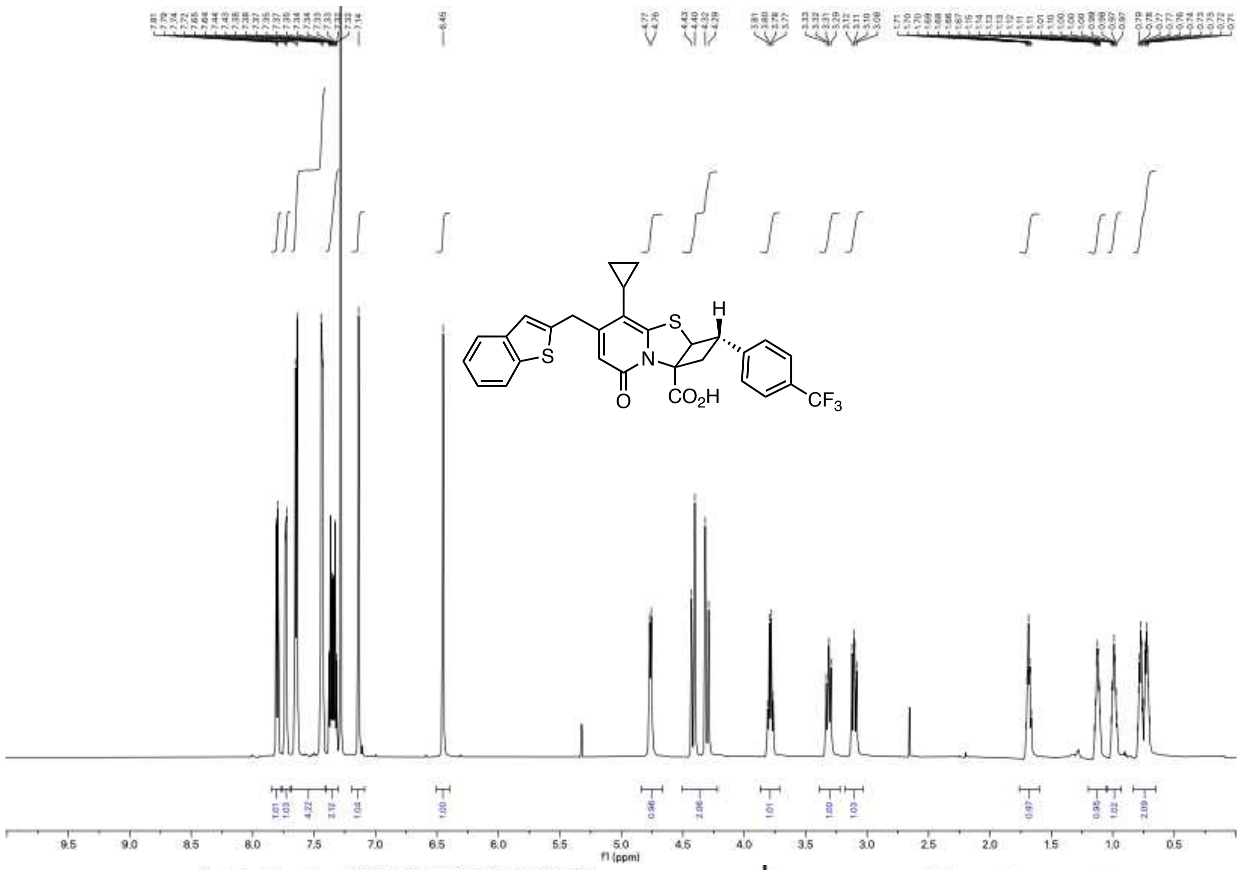

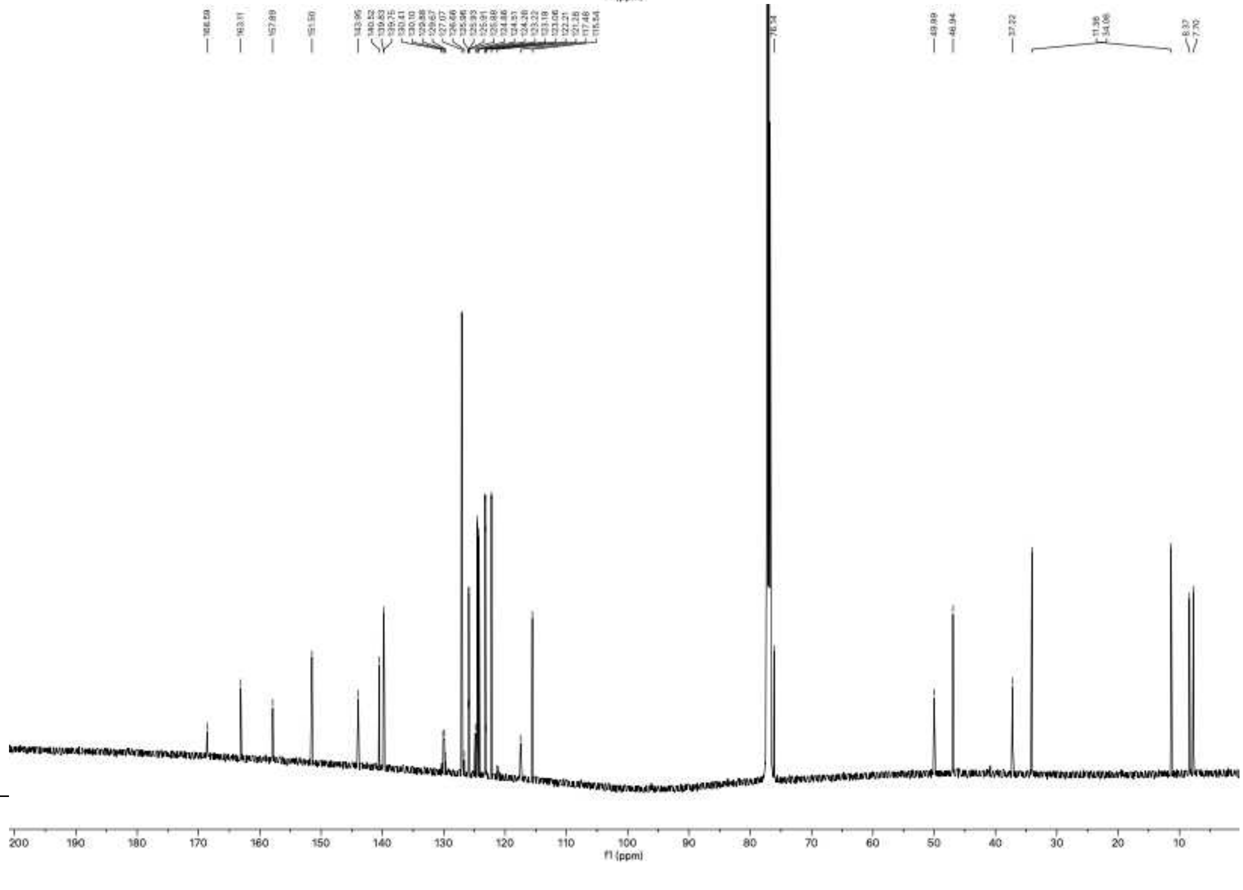

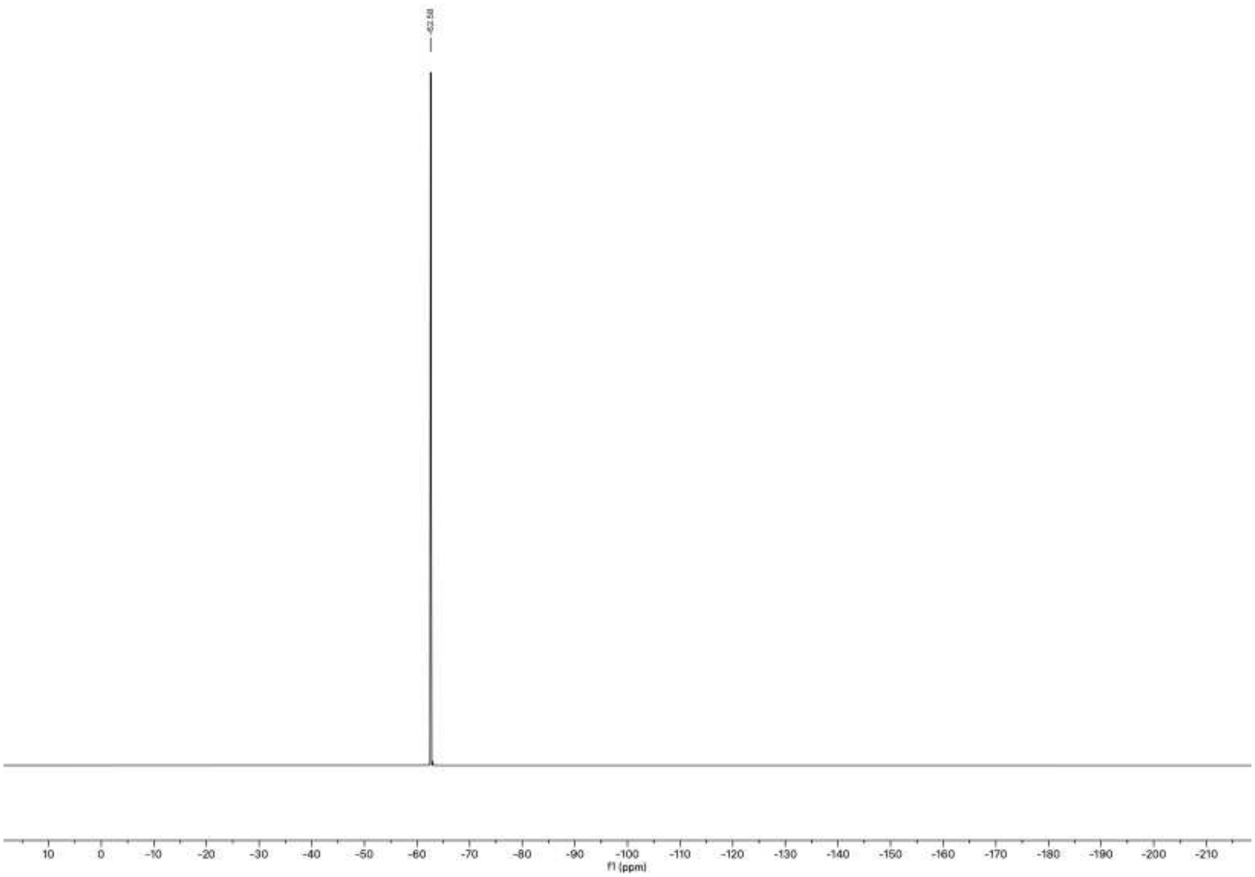

-62.58
10
0
-10
-20
-30
-40
-50
-60
-70
-80
-90
-100
-110
-120
-130
-140
-150
-160
-170
-180
-190
-200
-210
f1 (ppm)

Compound **SS1045B**, ^{1}H-NMR (600 MHz) and ^{13}C-NMR (151 MHz) (($(CD_3)_2SO$):

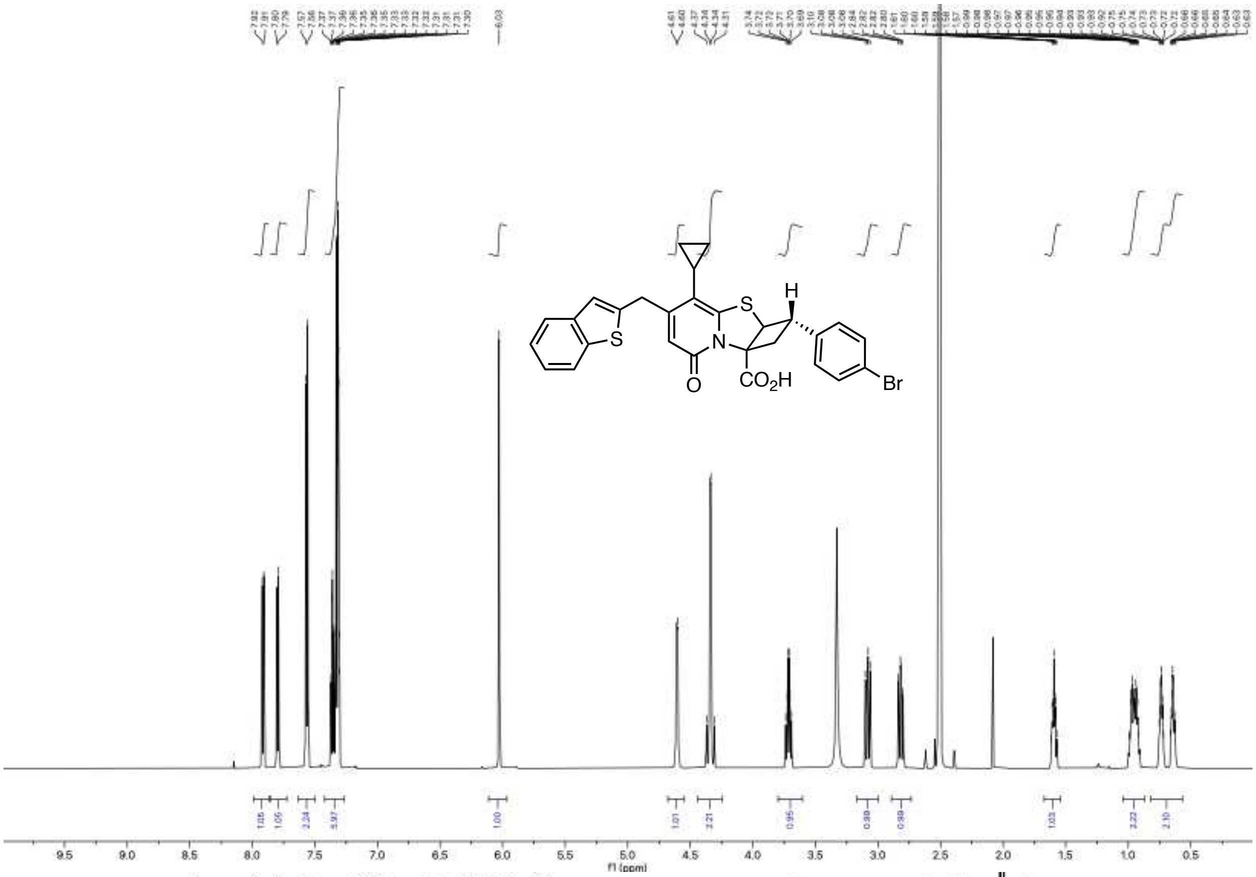

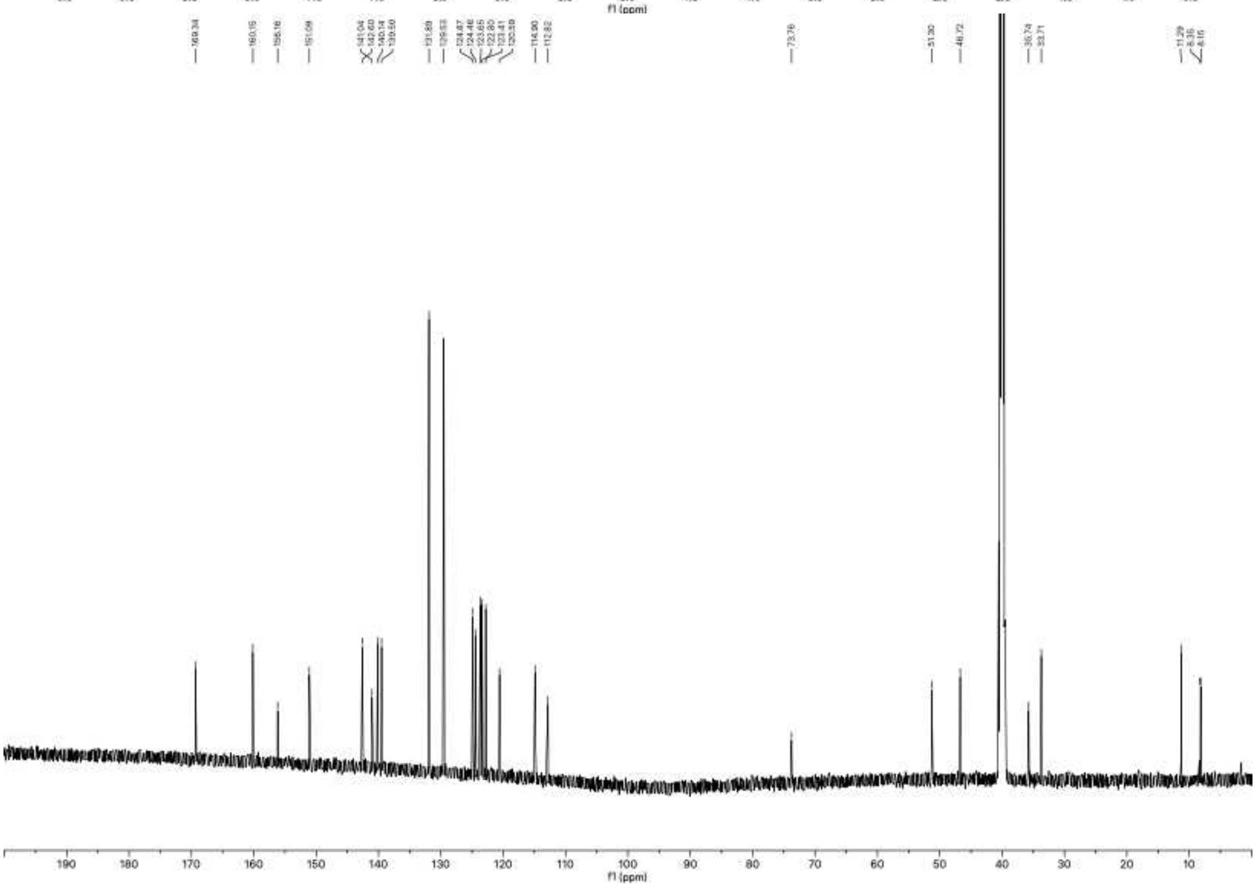

Compound **SS1022B**, ^{1}H-NMR (600 MHz) and ^{13}C-NMR (151 MHz) ($CDCl_3$):

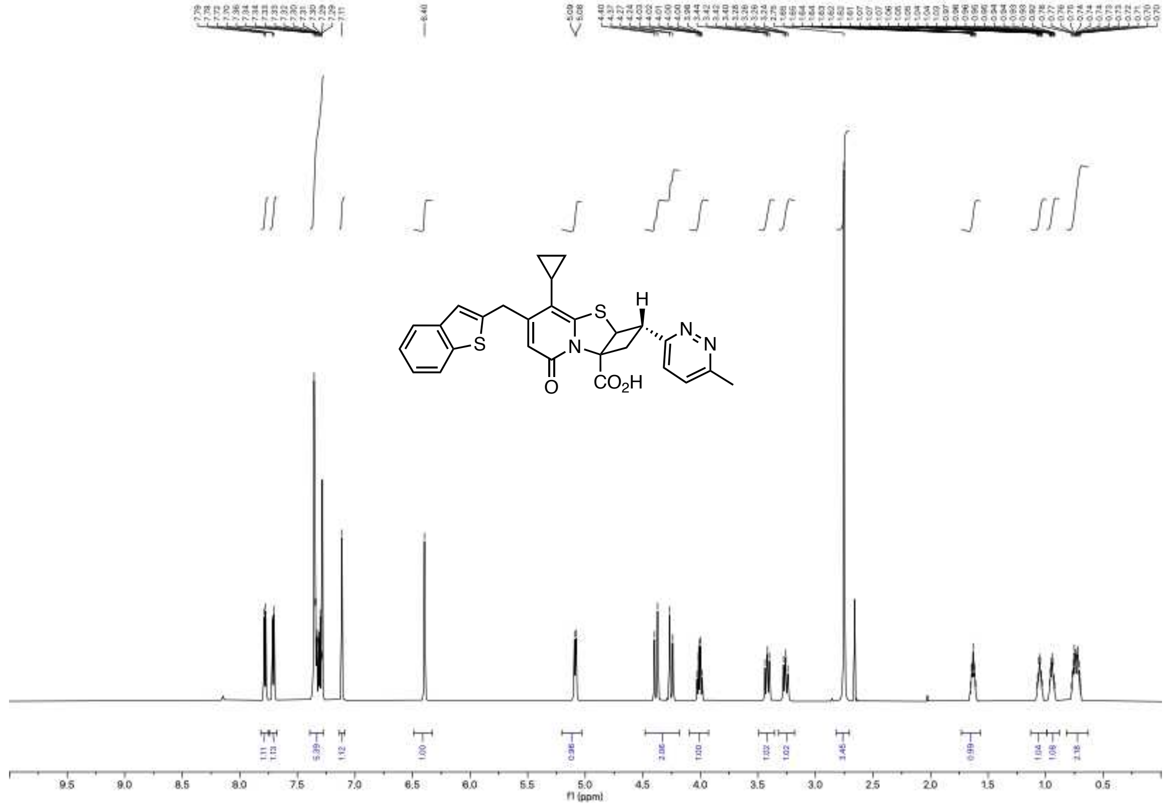

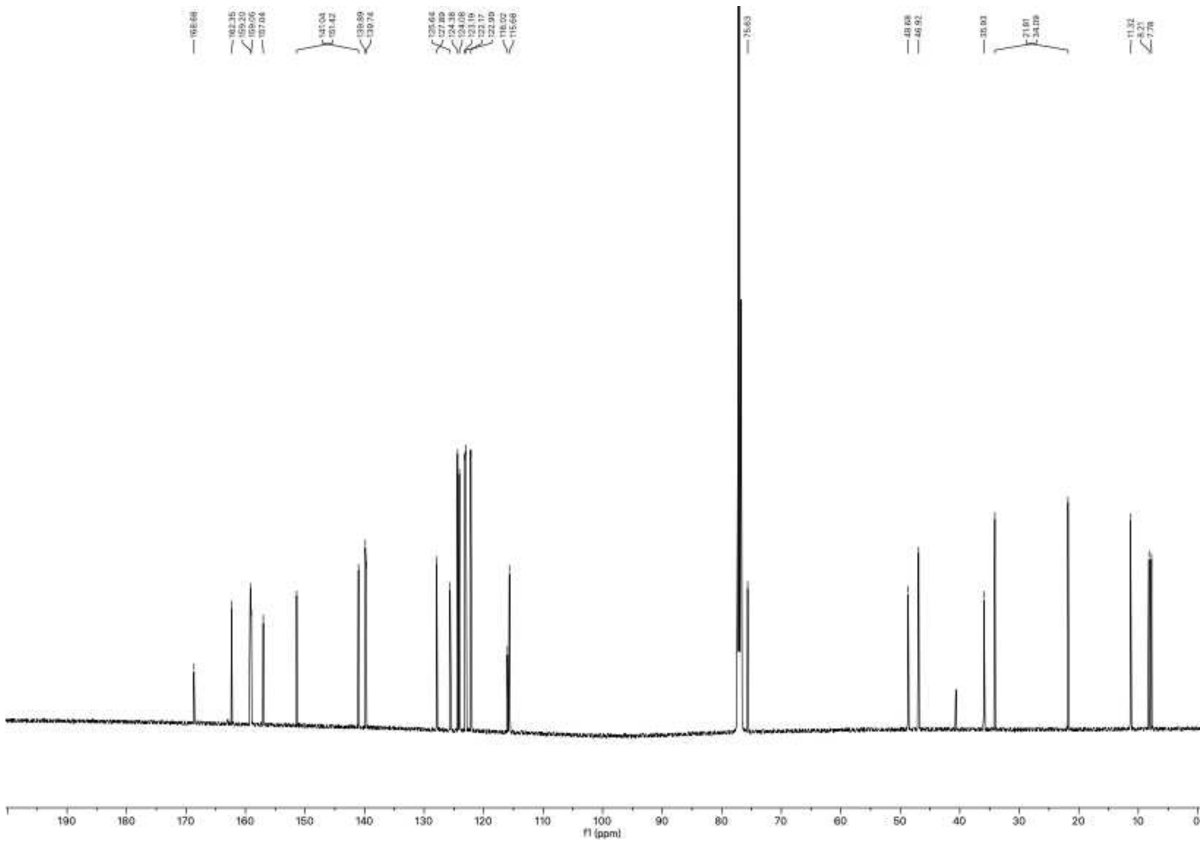

Compound **PS2840**, ^{1}H-NMR (600 MHz) and ^{13}C-NMR (151 MHz) ($(CD_3)_2SO$):

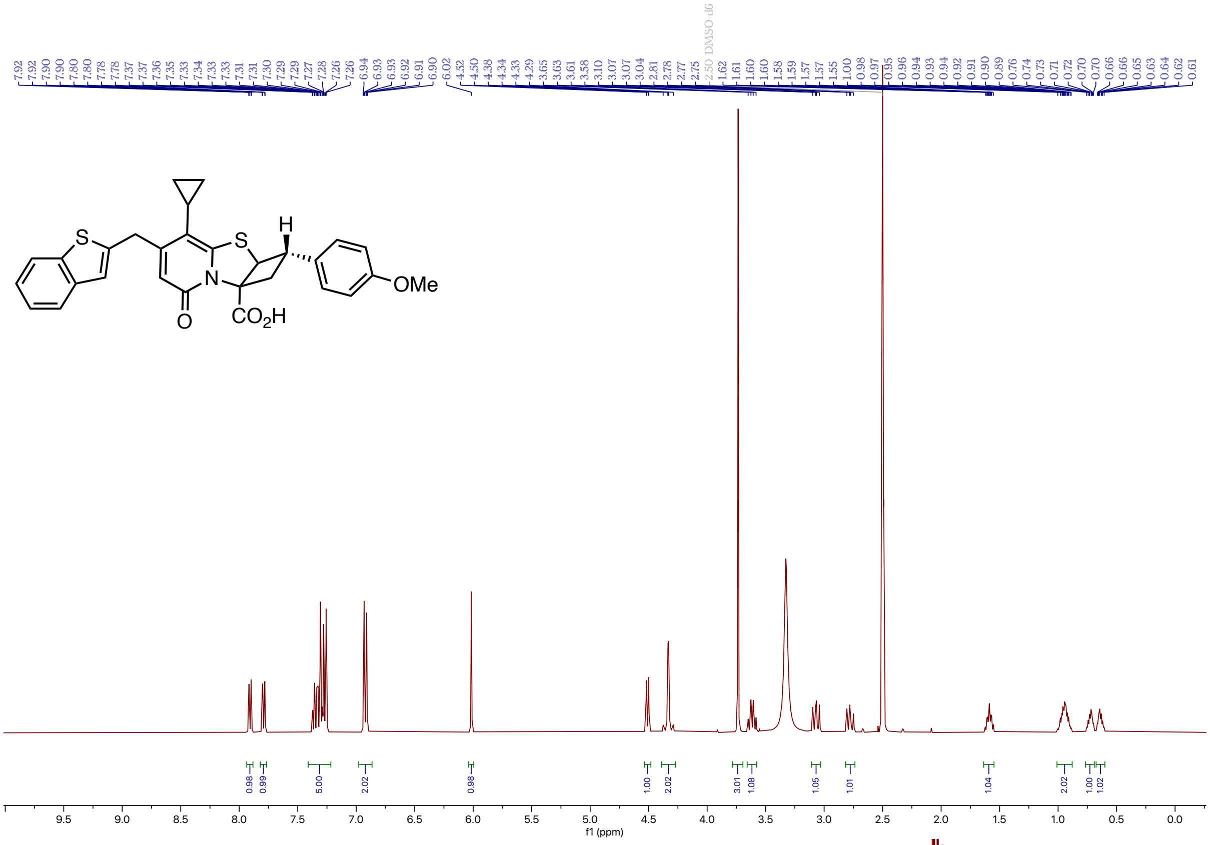

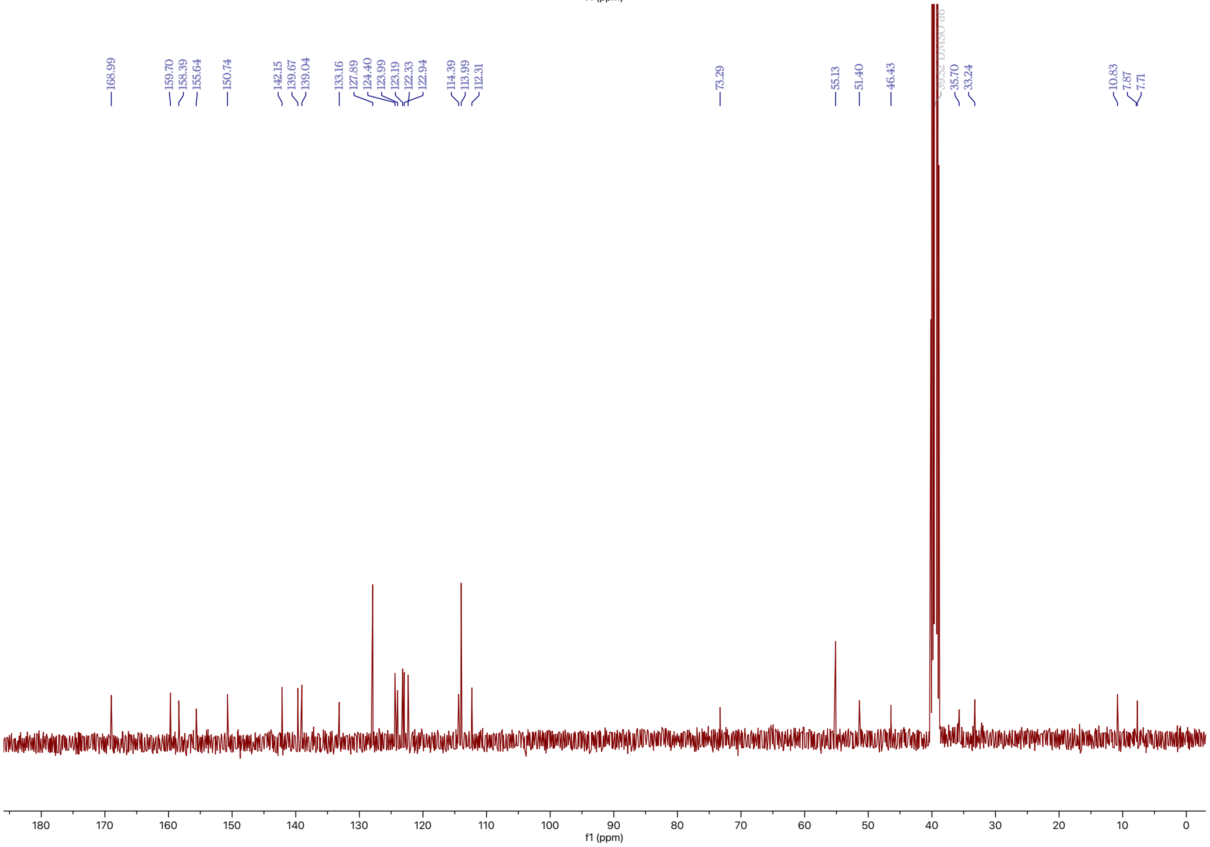

Compound **SS1238B**, ^{1}H-NMR (600 MHz) ($CDCl_3$), ^{13}C-NMR (151 MHz) ($CDCl_3$) and ^{19}F NMR (376 MHz) ($(CD_3)_2SO$):

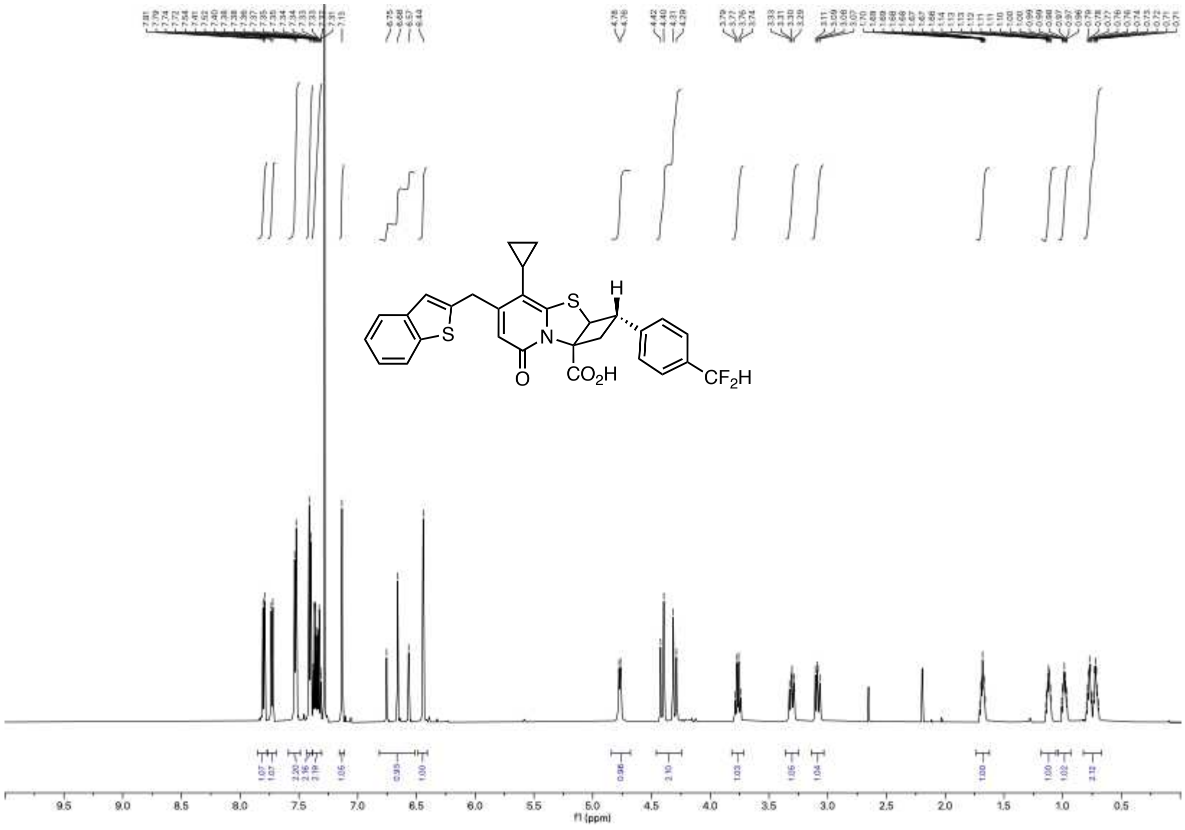

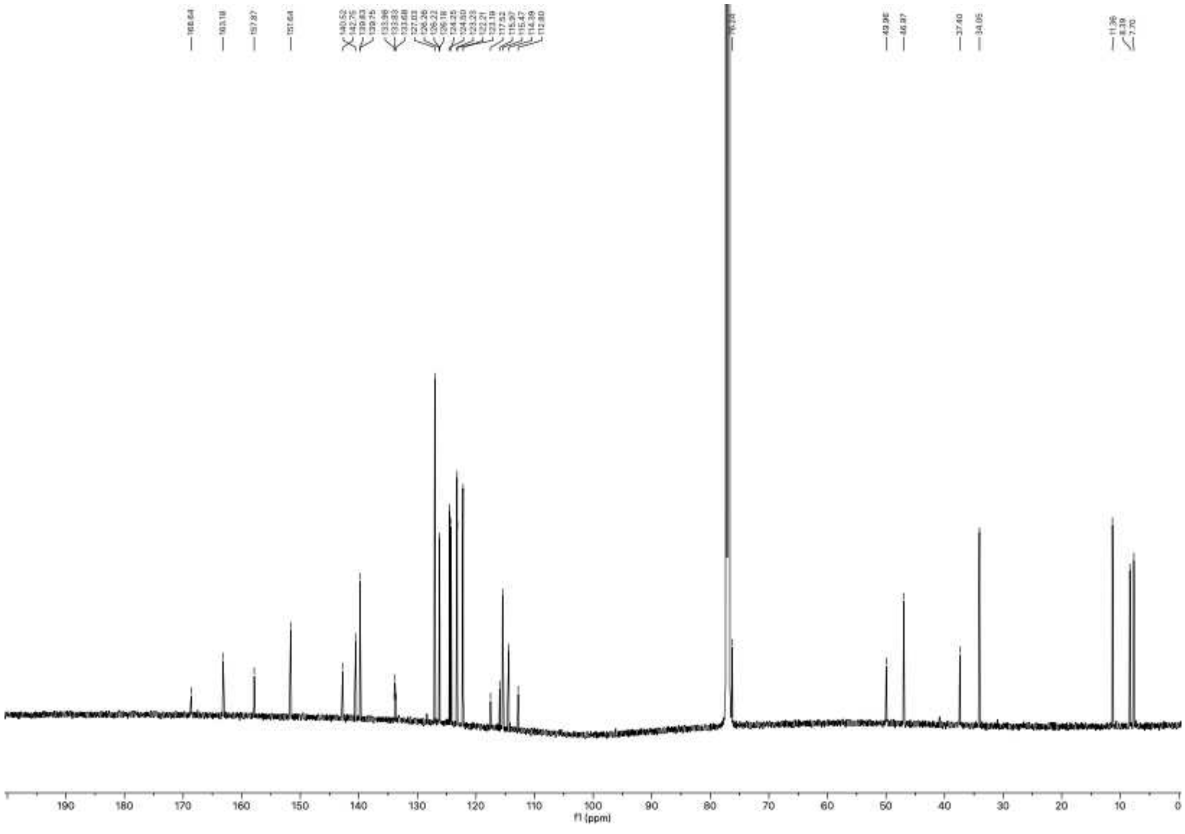

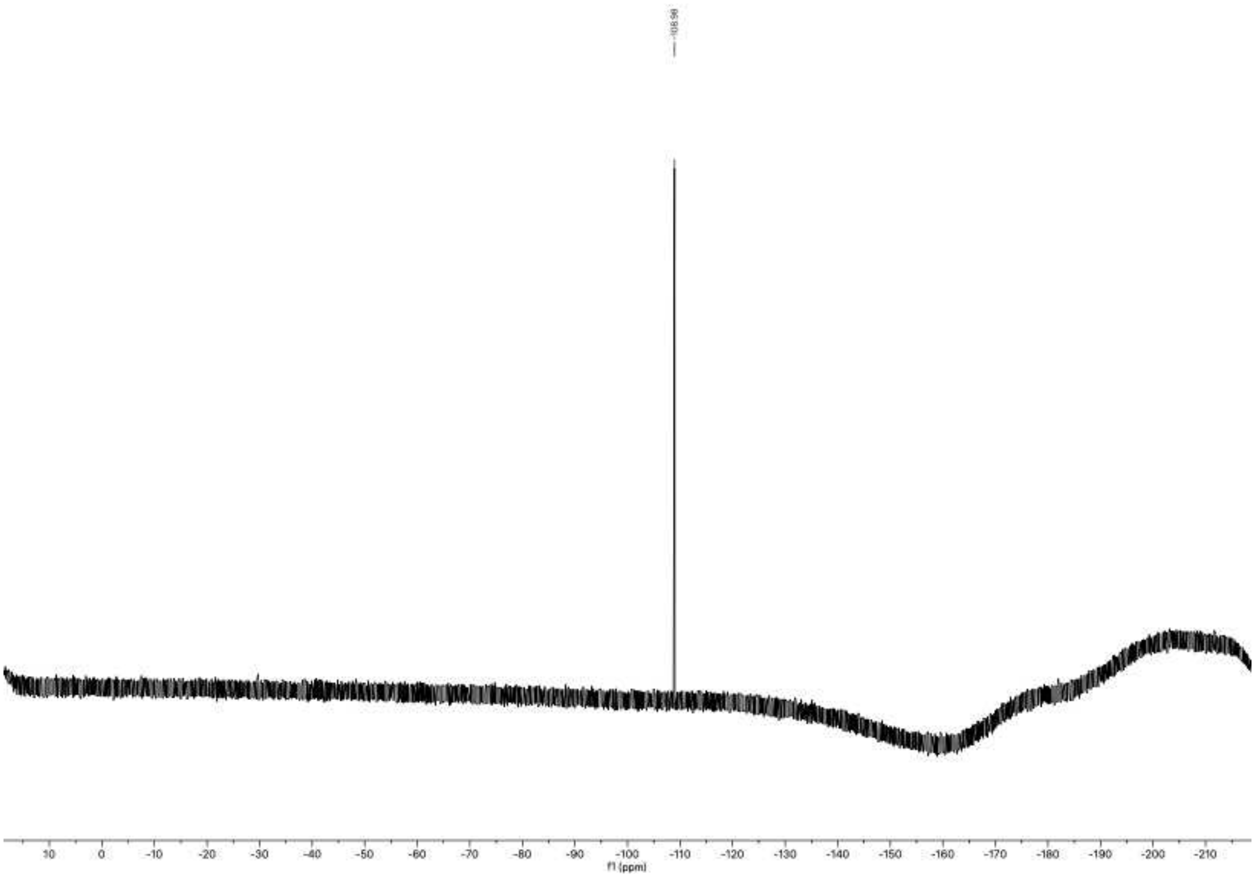
-108.98
10 0 -10 -20 -30 -40 -50 -60 -70 -80 -90 -100 -110 -120 -130 -140 -150 -160 -170 -180 -190 -200 -210
f1 (ppm)

Compound **SS998**, ^{1}H-NMR (600 MHz) and ^{13}C-NMR (151 MHz) ($CDCl_3$):

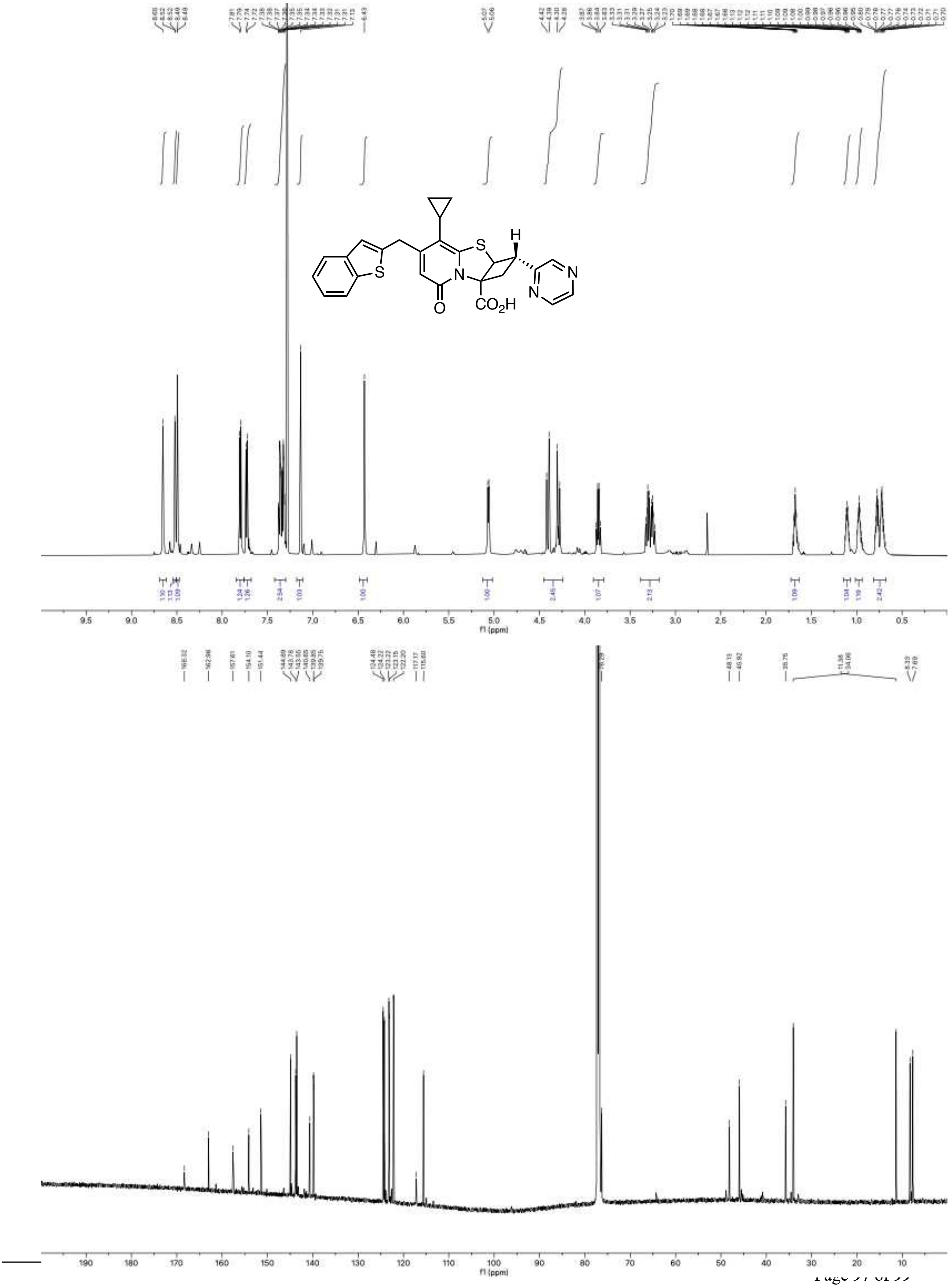

Compound **SS991B**, ^{1}H-NMR (600 MHz) and ^{13}C-NMR (151 MHz) (($(CD_3)_2SO$):

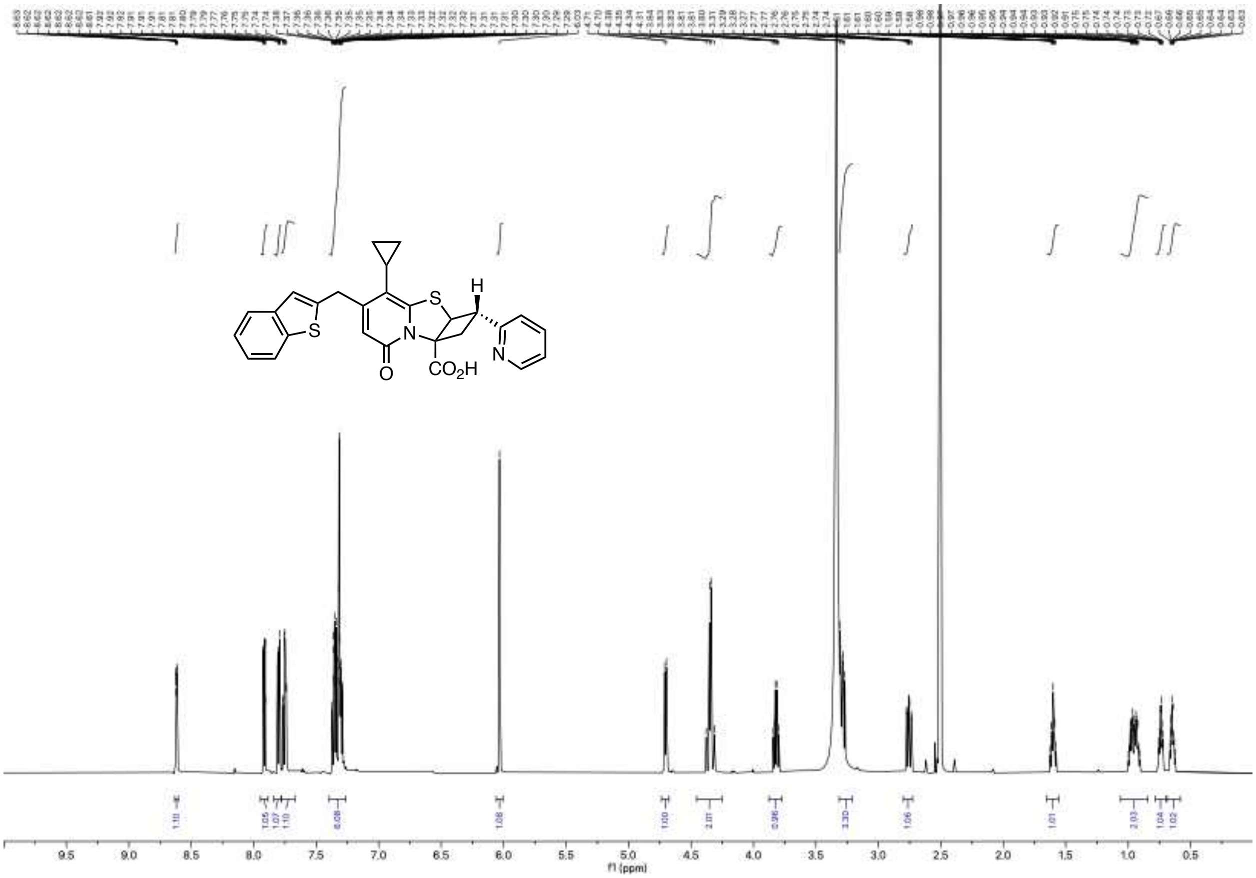

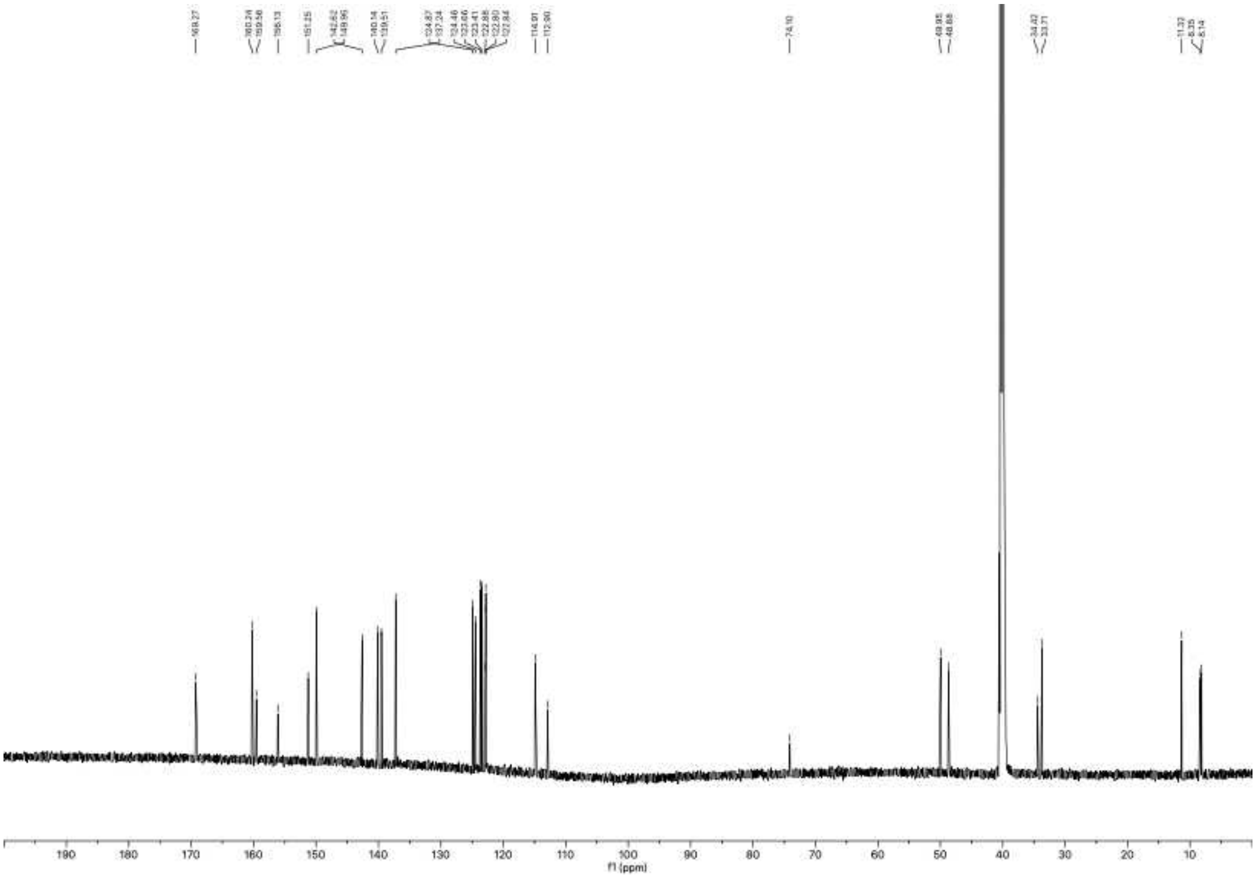

Data S1. (separate file)

Summary of TNseq-hits
